# Quality of life measures in Parkinson’s disease: a systematic literature review of patient-reported outcomes measures (PROMs) and their psychometric properties

**DOI:** 10.1007/s00415-025-13348-x

**Published:** 2025-08-28

**Authors:** Alberto de la Cuadra-Grande, Javier Rejas, Miguel Ángel Casado, Manuel Monroy, Miguel Ruiz

**Affiliations:** 1 Department of Social Psychology and Methodology, School of Psychology, Universidad Autónoma de Madrid, Madrid, Spain; 2 Pharmacoeconomics & Outcomes Research Iberia (PORIB), Paseo Joaquín Rodrigo 4-Letter I, 28224 Madrid, Spain; 3 EACCOS Research Group, School of Psychology, Universidad Autónoma de Madrid, Madrid, Spain; 4 Fundación PORIB, Paseo Joaquín Rodrigo 4-Letter I, 28224 Madrid, Spain

**Keywords:** Parkinson’s disease, Patient-reported outcomes measures, PROMs, Psychometric properties, Quality of life, Systematic literature review

## Abstract

**Background:**

Quality-of-life (QoL) measures are key for monitoring health of patients with Parkinson’s disease (PD).

**Objective:**

This systematic review aimed to gather evidence on the psychometric properties of available patient-reported outcomes measures (PROMs) for assessing QoL in people with PD (PwPD).

**Methods:**

A search of PROMs was conducted in PubMed (MEDLINE), Embase, Scopus, Web of Science, PSICDOC, and ‘gray literature’ (April 2024, PROSPERO-ID: CRD42024526458). After screening and data extraction, in a two-phase procedure conducted by independent reviewers, a critical assessment of feasibility, validity (content, structural, known-group, and criterion) and reliability (internal consistency, test–retest, and measurement error) was conducted based on the COSMIN criteria for good psychometric properties.

**Results:**

The search identified 83 eligible studies from which 29 PROMs were gathered (15 PD-specific and 14 generic/unspecific PROMs validated for PD). All PROMs proved their feasibility and included common dimensions between them, suggesting adequate content validity. Among the 29 PROMs, 17 reported data on structural validity (58.6%), 20 on known-group validity (69.0%), 25 on criterion validity (86.2%), 23 on internal consistency (79.3%), 11 on test–retest validity (37.9%), and 4 on measurement error (13.8%). According to the COSMIN criteria, 6 PROMs have the potential to be the most suitable QoL measure for PwPD: PDQ-39, PDQ-8, PDQL, PDQUALIF, PIMS, and Neuro-QOL.

**Conclusions:**

Several PROMs are feasible, valid, and reliable for measuring QoL in PwPD. However, further research ensuring their psychometric properties and cross-cultural adaptations are needed to recommend their use.

**Supplementary Information:**

The online version contains supplementary material available at 10.1007/s00415-025-13348-x.

## Introduction

Parkinson’s disease (PD) is the second most common neurodegenerative disease worldwide [1]. Although the etiology of the disease remains unknown, it is commonly accepted that the depletion of dopamine levels due to the loss of dopaminergic neurons, together with the involvement of other neural pathways, yields to a wide variety of symptoms [2, 3]. Motor symptoms include stiffness, freezing of gait, and postural instability or tremors, among others. Moreover, PD is also associated with nonmotor symptoms, such as neuro-psychiatric affections (cognitive impairment, dementia, depression, etc.), altered autonomic nervous system activity (constipation, diaphoresis, dysphagia, etc.), sleep disturbances, and sensorial disorders [2, 3].

In addition to the stigma caused by the disease [4, 5], all these PD-related symptoms have a remarkable impact on patients’ quality of life (QoL) and activities of daily living (ADL) [5–8]. Since there is still no curative treatment for the disease, the available therapeutic alternatives aim to improve or maintain patients’ QoL by reducing both motor and nonmotor disturbances [9].

However, QoL is a complex, multidimensional, and subjective concept that can be defined according to different approaches [10]. From a medical perspective, QOL is measured by the presence, frequency, and/or severity of symptoms as reported by the patient, and is referred to as health-related QoL (HRQoL). In this approach, better HRQoL implies a lack of pathology perceived by patients [10]. Nonetheless, social sciences consider a wider point of view, using another construct for QoL, which is named subjective well-being (SWB). In contrast to HRQoL, SWB measures the patient-reported satisfaction with life, expectations for the future, and a sense of fulfillment in life, both as a whole and by its domains [10].

Both HRQoL and SWB rely on patients’ self-reported perceptions [10], which are measured by instruments called patient-reported outcomes measures (PROMs), which are standardized and validated questionnaires measuring the perceptions of individuals regarding their health status and/or QoL [11]. These PROMs can be generic for multiple health conditions or even for a healthy population [10, 11]. However, since generic tools may not be sensitive to changes specific to a particular disease, this has favored the development of condition-specific PROMs [10, 11].

In the case of PD, several QoL measures have been developed since the 1990s; and thus, reviews of QoL measures in people with PD (PwPD) are currently available [12–21]. Nonetheless, given that PROMs are clinical tools, their feasibility, validity, and reliability must be proven by conducting psychometric studies [22]. Moreover, none of the previously mentioned reviews summarized and assessed critically their psychometric properties. For both reasons, the present study consisted of a systematic literature review (SLR) identifying all specific and generic PROMs for measuring QoL in patients with PD, which aimed to provide an in-depth description and critical evaluation of their content, psychometric properties, and adequacy with respect to the needs of patients with PD.

## Methods

### Systematic literature review

The SLR was conducted based on the Preferred Reporting Items in Systematic Literature Reviews and Meta-Analyses (PRISMA) [23] and COnsensus-based Standards for the selection of health Measurement INstruments (COSMIN) [24] guidelines.

An SLR protocol was first designed and registered in PROSPERO (CRD42024526458), including a wide investigation question: “What instruments or surveys are currently available for measuring patient-reported outcomes and experience?”. Additionally, a PICO (Population–Intervention–Comparator–Outcome) question was defined. The population included patients with PD, and the intervention included the administration of patient-reported measures (PRMs). No restrictions were established for comparators, outcomes, and dates. The inclusion and exclusion criteria are presented in Online Resource 1. The protocol was reviewed and validated by a specialist in documentation and literature review.

The search was conducted on 2nd April 2024, which included the terms “Parkinson’s Disease”, “Patient-Reported Outcomes Measure (PROM)”, “Patient-Reported Experience Measure (PREM)”, “Instrument”, “Tool”, “Questionnaire/Survey”, “Development”, “Design”, “Validation”, and “Psychometrics”. All the terms were combined via Boolean operators (OR/AND/NOT). The search was conducted in PubMed (MEDLINE), Embase, Scopus, Web of Science (WoS), and PSICODOC. The search also included a review of PRMs published in the ‘gray literature’, which included scientific congresses, scientific society publications, doctorate theses, and other databases. Online Resource 1 includes all the details regarding the inclusion and exclusion criteria, search strategies in every single database, and the search strategy for retrieving ‘gray literature’.

Previous SLRs describing PROMs in PwPD, which resulted from the present search, were collected. The reference sections of these studies were reviewed to gather additional psychometric studies that could not be identified.

Among all the references resulting from the search strategy, duplicates were removed using the software RefWorks®. A two-phase procedure was then followed by first reviewing the title and abstract. The full texts of the screened references were subsequently assessed to determine the eligibility of the studies. Data extraction from eligible studies was conducted using a structured extraction matrix. Two independent reviewers participated in the process of screening and data extraction. Discrepancies were resolved by sharing with the whole study group and reaching complete agreement.

For this study, data extracted for both generic and PD-specific PROMs measuring QoL were exclusively considered (including those designed for other diseases but currently validated for their use in PD).

### Critical assessment of QoL measures

A description of QoL measures was synthesized from the eligible references. When available, additional practical considerations concerning the PROMs were collected. These aspects included score range, ceiling and floor effects (percentages of patients obtaining the maximum and minimum scores, respectively), amount of missing data, minimal important (MIC) or detectable (MDC) changes, and administration time.

Regarding the psychometric properties, the critical appraisal of validity and reliability was performed based on the COSMIN Risk of Bias Tool [25–27].

Validity measures the adequacy of the questionnaire in measuring the construct for which it was designed [28]. Demonstrating the validity of a PROM requires deciding whether its content and items are appropriate in relation to the construct to be measured, which refers to content validity [28].

In addition, structural validity measures whether the instrument’s scores are an adequate reflection of the construct’s dimensionality [28]. The analysis of this property can be framed in classical test theory (CTT) or item response theory (IRT). The CTT assumes that the score in a test is determined by its hypothetical true score, which is the actual state of the unobservable variable of interest, and a random error produced by the influence of other variables [29]. In this framework, exploratory factor analysis (EFA) and confirmatory factor analysis (CFA) are the main methods used to determine the dimensionality of a PROM [29]. Factor analysis aims to detect latent patterns in the scores of the questionnaire, and, thus, reveals the domains assessed in the PROM [30]. While EFA is used when the domains of the instrument are unknown, CFA can be performed when there is previous evidence on its dimensionality arrangement [30]. In contrast, IRT develops mathematical models for measuring the probability of a response in the PROM’s items explained by the quantitative attributes of the respondents and the additional characteristics of the items [31, 32]. Rasch models are widely used for assessing structural validity of PROMs when they are unidimensional [33].

Under the assumption that the PROM is valid, other properties can be examined. The known-group validity implies the ability of the PROM’s scores to identify differences between groups of respondents with known differential characteristics in advance [28]. Methods for assessing this property are based on the traditional hypothesis testing, which aims to determine differences in the PROM’s scores between groups that are expected to differ according to clinical or sociodemographic evidence (e.g., severity of disease, age, etc.) [28].

Similarly, criterion validity measures the relationship between the PROM’s scores and an external assessment of the patient (clinical screening or biological indicators) [28]. This relationship is usually assessed by determining Pearson or Spearman correlations between the scores of the studied PROM and the Gold Standard [28].

Reliability refers to the degree to which the instrument’s scores remain constant when several measurements are repeated under similar conditions [28]. The internal consistency represents one of the properties that determines whether an instrument is accurate enough (or error free). This property measures the interrelatedness among the theoretically related PROM’s items (parallel items) [28]. Although several measures are available, including item–total or item–domain correlations, the most commonly used estimate for this property is Cronbach’s alpha, which is a measure of the average inter–relatedness of items included in the dimension [34].

The time stability of the questionnaire scores is also an indicator of reliability [28]. To assess this property, the test–retest correlation is the preferred method, which can be measured via intraclass correlation coefficient (ICC) of two subsequent administrations of the PROM after a relatively short period of time, ensuring that the respondent has not experienced substantial changes impacting the scores [35–37].

Finally, reliability can also be defined as the proportion of the total variance in scores that is produced by ‘true’ differences between patients. Thus, the measurement error (ME) can also be examined, which provides the amount of systematic and random error in a patient’s score that is not due to actual changes in the measured construct [28].

Two additional psychometric properties should be evaluated according to the COSMIN guidelines [25–27], but were dismissed for this study. First, responsiveness, which refers to the ability of the PROM to detect changes over time, was discarded as the SLR was constrained to cross-sectional studies. Cross-cultural validity, also called measurement invariance, indicates whether translated or culturally adapted items of the PROM perform similarly to the original version. This second property was excluded, since none of the eligible studies presented results in this regard.

As mentioned previously, all these properties were critically reviewed via the COSMIN Risk of Bias Tool (Online Resource 2) [25–27]. However, the content validity assessment is mainly qualitative. Thus, inductive content analysis techniques were used to organize information into previously known categories [38, 39]. The established categories corresponded to the defined domains of the currently available QoL measures for PwPD, which were also classified as HRQoL (ADL, cognition, symptoms of PD, and treatment for PD), SWB (facing PD and emotional disturbances), or miscellaneous (psychological disturbances, financial condition, work, and social and family life). The inductive content analysis was performed as follows [38, 39]: 1) extraction of data (items) from PROMs; 2) selection of key words; 3) codification, by determining key words; 4) categorization, by grouping codes in categories providing related information; and 5) integration categories, codes, and cites in conceptual models.

Finally, the formulation of recommendations on the usage of the available PROMs represents the last step when conducting a SLR of these tools according to the COSMIN guidelines [24]. Thus, after summarizing the extracted data, an evidence-based recommendation was established [24]: (A) “PROMs that have potential to be recommended as the most suitable PROM for the construct and population of interest”; (B) “PROMs that may have the potential to be recommended, but further validation studies are needed”; or (C) “PROMs that should not be recommended”.

## Results

### Systematic literature review results

**Fig. 1:**
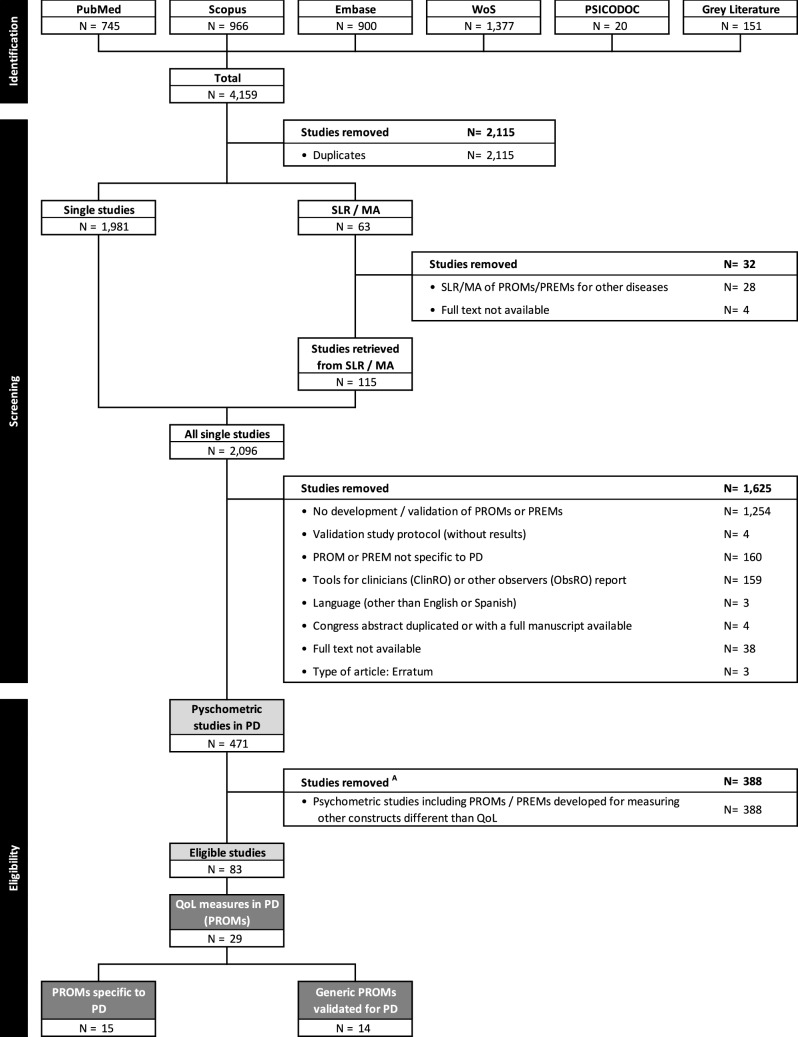
PRISMA flowchart of the screening and selection process. The list of abbreviations is provided in Online Resource 11. ^A^ Studies removed for this study’s purposes

The search retrieved 4159 references from all the databases and ‘gray literature’ (Fig. 1). A total of 2115 duplicates were discarded. Among the remaining 2044 studies, 63 consisted of previous SLRs, which provided 115 additional psychometric studies evaluating PROMs in PwPD. Thus, 2,096 studies were ultimately screened. After removing 1,625 studies, the remaining 471 studies were eligible according to the SLR objectives. The present study focused exclusively on QoL; thus, only 83 studies were selected [40–122]. The studies, participants’ clinical and sociodemographic characteristics, and their scores on the PROMs are described in Online Resource 3.

From the eligible studies, a total of 29 different QoL measures were identified (Table 1). Of those, 15 were specific for PD, and 14 consisted of unspecific or generic instruments. All these PROMs are described in Online Resource 4, which includes a summary of their evidence on feasibility.

**Table 1:** Synthesis of evidence for currently available PROMs for measuring QoL in PwPD

PROMs ^A^	Number of studies	[References]	COSMIN evidence-based recommendation ^B^	Number of studies evaluating the property/COSMIN RoB ^C^/COSMIN good property ^D^					
				Validity			Reliability		
				Structural	Know-group	Criterion	Internal consistency	ICC (test–retest)	ME
Specific PROMs for PD									
Bela-P-K	3	[40–42]	(B)	–	2/VG/(–)	2/VG/(+)	3/VG/(+)	1/VG/(?)	–
Indo-PDQOL	1	[43]	(B)	1/D/(?)	–	1/VG/(?)	1/VG/(+)	–	–
OFFELIA	2	[44, 45] ^E^	(B)	2/VG/(?)	2/VG/(–)	–	1/VG/(+)	–	–
PDQ-39	42	[46–87] ^E^	(A)	15/VG/(+)	31/VG/(+)	20/VG/(+)	37/VG/(+)	16/VG/(+)	2/VG/(?)
PDQ-8	16	[46, 57, 62, 71, 75, 76, 84, 86–94]	(A)	7/VG/(+)	4/VG/(+)	14/VG/(+)	10/VG/(+)	5/VG/(+)	1/VG/(?)
PDQ-DAT	1	[97]	(B)	1/VG/(?)	–	–	1/VG/(+)	–	–
PDQL	7	[65, 69, 81, 98–101]	(A)	–	4/VG/(?)	7/VG/(+)	7/VG/(+)	4/VG/(+)	1/VG/(?)
PDQoL7	1	[96]	(B)	1/VG/(?)	–	1/VG/(+)	1/VG/(+)	–	–
PDQUALIF	1	[102]	(A)	1/VG/(?)	1/VG/(+)	1/VG/(+)	1/VG/(+)	1/VG/(+)	–
PIMS	3	[69, 103, 104]	(A)	2/VG/(?)	3/VG/(+)	2/VG/(+)	3/VG/(+)	3/VG/(+)	–
QLPD	1	[105]	(B)	1/VG/(?)	1/VG/(+)	1/VG/(+)	1/VG/(+)	–	–
QLSM-DBS	2	[106, 107]	(B)	1/VG/(?)	–	2/VG/(+)	1/VG/(?)	1/VG/(+)	–
QLSM-MD	2	[106, 107]	(B)	1/VG/(?)	–	2/VG/(+)	1/VG/(+)	1/VG/(+)	–
QoLQ-PwP	1	[108]	(B)	1/VG/(?)	–	1/VG/(+)	1/VG/(+)	–	–
QOLSQ	1	[109]	(B)	–	1/VG/(+)	–	1/VG/(+)	1/VG/(+)	–
Unspecific PROMs validated for PD									
15D	3	[61, 110, 111]	(B)	–	3/VG/(+)	3/VG/(+)	–	–	–
EQ-5D-3L	4	[52, 95, 112, 113]	(B)	–	3/VG/(+)	4/VG/(+)	–	–	–
EQ-5D-5L	2	[93, 110]	(B)	–	1/VG/(+)	2/VG/(+)	1/VG/(+)	–	–
EQ-VAS	3	[52, 93, 112]	(B)	–	3/VG/(+)	3/VG/(+)	–	–	–
McGill QOL	1	[84]	(B)	1/VG/(?)	1/VG/(–)	1/VG/(+)	1/VG/(+)	–	–
Neuro-QOL	2	[114, 115] ^E^	(A)	–	2/VG/(+)	1/VG/(+)	1/VG/(+)	1/VG/(+)	1/VG/(?)
PGI	2	[116, 117] ^E^	(B)	–	–	1/VG/(+)	–	–	–
PROMIS-29	1	[84]	(B)	1/VG/(?)	1/VG/(+)	1/VG/(+)	1/VG/(+)	–	–
QOL-AD	1	[84]	(B)	1/VG/(?)	1/VG/(–)	1/VG/(+)	1/VG/(+)	–	–
SF-12	1	[118]	(B)	1/VG/(?)	–	–	–	–	–
SF-36	3	[52, 119, 120]	(B)	1/VG/(+)	1/VG/(+)	1/VG/(+)	2/VG/(+)	1/VG/(+)	–
SF-6D	2	[110, 111]	(B)	–	2/VG/(+)	2/VG/(+)	–	–	–
WHO-5	1	[121]	(B)	–	–	1/VG/(+)	1/VG/(+)	–	–
WHOQOL-BREF	1	[122]	(B)	–	1/VG/(?)	1/VG/(+)	1/VG/(+)	–	–

^A^List of abbreviations is provided in Online Resource 11

^B^COSMIN recommendations for the use of PROMs are as follows [24]: (A) “PROMs that have potential to be recommended as the most suitable PROM for the construct and population of interest”; (B) “PROMs that may have the potential to be recommended, but further validation studies are needed”; or (C) “PROMs that should not be recommended”

^C^COSMIN RoB can be defined as [24]: “Very good” (VG), “Good” (G), “Doubtful” (D), and “Inadequate” (I)

^D^COSMIN definition of a good property are as follows (Online Resource 2) [24]: (+), (–), (±) and (?)

^E^Several studies included the same participants but provided complementary results

### Review of psychometric properties

In the critical appraisal of content validity (Table 2), only the Patient Generated Index (PGI) included all the categories identified in the content analysis. The health-related quality-of-life instrument for Hindi speaking Parkinson’s disease patients (Indo-PDQOL) and Quality of Life in Parkinson’s Disease (QLPD) included 9 out of 10 categories; followed by the Off Episode Quality of Life Impact Scale (OFFELIA) and Neuro Quality of Life (Neuro-QOL), which included 7 categories; and both the Parkinson’s Disease Questionnaire 39-items (PDQ-39) and the 8-items (PDQ-8), which included 6 categories. The remaining PROMs included 5 or fewer domains.

**Table 2:** Assessment of the content validity of PROMs for measuring QoL in PwPD

PROMs ^A^ (Domains included/total domains)		Inclusion of the domain in the PROM									
		HRQoL				SWB		Miscellaneous			
		ADL	Cognition	Symptoms of PD	Treatment for PD	Facing PD	Emotional disturbances	Psychological disturbances	Financial condition	Work	Social and family life
**Specific PROMs for PD**	**Bela-P-K** (4/10)		X			X	X			X	
	**Indo-PDQOL** (9/10)	X	X	X	X	X	X	X	X		X
	**OFFELIA** (7/10)	X		X		X	X	X		X	X
	**PDQ-39** (6/10)	X	X	X			X	X			X
	**PDQ-8** (6/10)	X	X	X			X	X			X
	**PDQ-DAT** (5/10)	X	X	X	X		X				
	**PDQL** (7/10)	X	X	X		X	X	X			X
	**PDQoL7** (5/10)	X	X	X				X			X
	**PDQUALIF** (6/10)	X		X		X	X	X			X
	**PIMS** (4/10)					X			X	X	X
	**QLPD** (9/10)	X	X	X	X	X	X	X	X		X
	**QLSM-DBS** (2/10)			X	X						
	**QLSM-MD** (5/10)	X	X	X		X					X
	**QoLQ-PwP** (0/10)										
	**QOLSQ** (4/10)			X			X	X			X
**Unspecific PROMs validated for PD**	**15D** (3/10)	X					X			X	
	**EQ-5D-3L** (1/10)						X				
	**EQ-5D-5L** (1/10)						X				
	**EQ-VAS** (0/10)										
	**McGill QOL** (3/10)						X	X			X
	**Neuro-QOL** (7/10)	X	X	X		X	X	X			X
	**PGI** (10/10)	X ^B^	X ^B^	X ^B^	X ^B^	X ^B^	X ^B^	X ^B^	X ^B^	X ^B^	X ^B^
	**PROMIS-29** (3/10)			X				X			X
	**QOL-AD** (5/10)	X	X				X		X		X
	**SF-12** (4/10)	X						X		X	X
	**SF-36** (5/10)	X		X				X		X	X
	**SF-6D** (5/10)	X		X				X		X	X
	**WHO-5** (1/10)						X				
	**WHOQOL-BREF** (7/10)	X	X		X		X	X		X	X

^A^ List of abbreviations is provided in Online Resource 11

^B^ The PGI includes all dimensions as its design requires patients to determine the five areas of health that most impact their QoL. The PROM also allows patients to assess the impact of non-health areas on their QoL

Concerning additional validity indicators (Table 3), among the 29 PROMs identified, evidence for structural validity was available for 17 (58.6%, Online Resource 5), 20 had data on known-group validity (69.0%, Online Resource 6), and 25 had studies on criterion validity (86.2%, Online Resource 7). Complete evidence for validity, including content, structural, known-group, and criterion validity, was available for 9 PROMs exclusively (31.0%): PDQ-39, PDQ-8, Parkinson’s Disease Quality of Life Scale (PDQUALIF), Parkinson’s Impact Scale (PIMS), QLPD, McGill QoL, Patient-Reported Outcomes Measurement Information System 29-items (PROMIS-29), QoL – Alzheimer’s Disease (QOL-AD), and Short Form 36-items (SF-36).

**Table 3:** Validity of PROMs for measuring QoL in PwPD

PROMs ^A^	Structural validity (Online Resource 5)		Know-Group Validity (Online Resource 6) [group differences detected]	Criterion validity (Online Resource 7) [correlated as hypothesized with other measures]
	CTT [EFA/CFA results]	IRT [Rasch model results]		
Specific PROMs for PD				
Bela-P-K	–	–	None of the groups studied (H&YS)	SIP, COOP/WONCA, Loneliness Scale, PDQ-39
Indo-PDQOL	EFA identified 12 factors (estimates not available)	–	–	SF-36, PDQ-39, socioeconomic status, dose of levodopa
OFFELIA	EFA identified 2 domains: 1) Functioning (13 items; r = 0.42–0.89; eigenvalue = 10.28; variance explained = 88%); and 2) Emotional wellbeing (5 items; r = 0.54–0.86; eigenvalue = 1.41; variance explained = 12%). Factor loadings were > 0.4 for all items	Some evidence of DIF by sex and location with no relevant magnitude (ΔR^2^ < 0.035). The best fitting model identified two domains: 1) Functioning (13 items); and 2) Emotional wellbeing (5 items). The item “Employment” had mixed evidence for its inclusion	Unpredictability of the Off episodes, duration of the Off episodes (PDQ-8 performed better for those groups of 2 h and > 4 h) and time since PD onset	–
PDQ-39	EFA identified 8 domains (total variance explained = 45.3–67.0%; eigenvalue = 3.6–4.89): 1) Mobility (loadings = 0.74–0.81); 2) ADL (loadings = 0.75–0.81); 3) Emotional wellbeing (loadings = 0.76–0.81); 4) Stigma (loadings = 0.50–0.74); 5) Social support (loadings = 0.55–0.83); 6) Cognition (loadings = 0.71–0.76); 7) Communication (loadings = 0.67–0.76); and 8) Bodily discomfort (loadings = 0.58–0.83) CFA confirmed the 8 domains (eigenvalues > 1; variance explained = 75.5%; Chi-squared = 32,02–1,885.85; GFI = 0.92; RMR = 0.04; NFI = 0.92; TLI = 0.95; IFI = 0.97; CFI = 0.96; RMSEA = 0.10): 1) Mobility (beta = 0.72); 2) ADL (beta = 0.69); 3) Emotional wellbeing (beta = 0.77); 4) Stigma (beta = 0.78); 5) Social support (beta = 0.77); 6) Cognition (beta = 0.79); 7) Communication (beta = 0.76); 8) Bodily discomfort (beta = 0.73)	The Rasch model estimates, considering the response as a logistic function, for Mobility (disordered item response threshold [n] = 6/Person strata [n] = 4.35; Logit [SD] = −0.11 [1.99]), ADL (disordered item response threshold [n] = 3; Person strata [n] = 4.33; Logit [SD] = −0.27 [1.37]), Emotional wellbeing (disordered item response threshold [n] = 0; Person strata [n] = 3.40; Logit [SD] = −1.22 [1.85]), Stigma (disordered item response threshold [n] = 1; Person strata [n] = 2.63; Logit [SD] = −0.74 [1.50]), Social support (disordered item response threshold [n] = 2; Person strata [n] = 1.31; Logit [SD] = 0.97 [1.21]), Cognition (disordered item response threshold [n] = 3; Person strata [n] = 2.23; Logit [SD] = −0.81 [1.60]), Communication (disordered item response threshold [n] = 1; Person strata [n] = 2.43; Logit [SD] = 1.21 [2.15]) and Bodily discomfort (disordered item response threshold [n] = 1; Person strata [n] = 2.07; Logit [SD] = 0.15 [1.66])	Sex, severity of symptoms (tremor, stiffness, slowness, freezing and jerking), H&YS, presence and severity of depression (assessed by MADRS or BDI), MMSE, falls, postural instability, perception of health changes compared with the previous year, motor fluctuations, dementia and use of palliative care. Differences were also found when comparing scores of patients with PD with healthy controls	severity of symptoms (tremor, stiffness, slowness, freezing, jerking, postural instability and gait disturbances), H&YS, Columbia scale, UPDRS (Parts I to IV and total score), S&E, SPMSQ, GDS, HADS-A, HADS-D, SF-36, MADRS, EQ-5D, EQ-VAS, duration of PD (years), BDI, MMSE, NHP, 15D, duration of the treatment with levodopa (years), daily dose of levodopa (mg), PDQL, SCOPA/SPES (parts A, B C and total), Pfeiffer, PIMS, patient’s age, ESS, PDQ-8, NMSS, PROMIS-29, McGill QOL, ESAS-PD, PDSQ, MoCA, GDS, CDR, Postural Instabilities and Gait Difficulty (PIGD) and Timed Up and Go (TUG)
PDQ-8	EFA identified a single factor: total variance explained = 37.16–60.00%; eigenvalue = 3.9–4.35; loadings = 0.27–0.89 CFA confirmed a single factor (variance explained = 44.12–54.51%; Chi-squared = 1.56–34.05; GFI = 0.91–0.95; RMR = 0.07–0.09; NFI = 0.88–0.91; TLI = 0.91–0.97 IFI = 0.94–98; CFI = 0.94–98; RMSEA = 0.06–0.10), and beta for Mobility (0.67–0.68), ADL (0.78–0.79), Emotional wellbeing (0.51–0.62), Social support (0.62–0.77), Cognition (0.49–0.52), Communication (0.73), Bodily discomfort (0.62–0.67) and Stigma (0.83–0.84)	The Rasch model (DIF = [−0.19]−0.27) difficulty estimates (Measure/SE/Infit MnSq/Outfit MnSq/Lower Threshold/Upper Threshold) for Mobility (−1.29/0.14/0.98/0.96/−2.97/0.39), ADL (−0.69/0.14/1.21/1.19/−2.37/0.99), Emotional wellbeing (−0.57/0.14/0.81/0.85/−2.25/1.11), Stigma (0.02/0.14/01.15/1.13/−1.66/1.70), Social support (2.26/0.19/1.16/1.06/0.58/3.94), Cognition (0.43/0.14/0.88/0.92/−1.25/2.11), Communication (0.99/0.15/0.84/0.76/−0.69/2.67) and Bodily discomfort (−0.74/0.14/0.99/1.02/−2.42/0.94) Model fit statistics: Item Separation Index = 6.99/Item Separation Reliability = 0.98/Person Separation Index = 1.53/Person Separation Reliability = 0.70	H&YS, gender, level of studies, work status, treatment with levodopa, duration of PD (years), EQ-VAS scores, presence of dyskinesia, wearing off, freezing, postural instability, cognitive impairment and depression. Differences were also found when comparing scores of patients with PD with healthy controls	PDQ-39, Columbia scale, EQ-5D, EQ-VAS, SF-36, age (years), duration of PD (years), UPDRS, ADL, BDI, H&YS, IPA-I, UPDRS (Pa Parts I to IV and total score), S&E, daily dose of levodopa (mg), duration of the treatment with levodopa (years), pain, TDQ, PSQI, MMSE, MoCA, GDS, CDR, Zarit scale, carer EQ-5D, carer EQ-VAS, PD type, motor symptoms, presence of cognitive impairment, depression, Disability-Off Index, Disability-LID Index and Non-Motor Questionnaire
PDQ-DAT	EFA identified 3 dimensions (estimates not available)	–	–	–
PDQL	–	–	Severity of PD and H&YS	MOS-24, CES-D, PDQ-39, H&YS, S&E, UPDRS (Pa Parts I to IV and total score), HADS-A, HADS-D, duration of PD (years), daily dose of levodopa (mg), duration of the treatment with levodopa (years), age, SCOPA/SPES-A, PIMS, BDI and SF-36
PDQoL7	EFA identified 3 factors (variance explained = 76%)	–	–	PD diagnosis (years), H&YS, cognitive impairment, Disability-Off Index, Disability -LID Index, Non-Motor Questionnaire, Depressive status and EQ-5D-5L
PDQUALIF	EFA identified 7 factors (variance explained = 55.6%; loadings = 0.43–0.82)	–	H&YS	UPDRS (total and parts 1, 2 and 3), SF-36 (physical and mental components), SIP (total, physical and psychosocial), age (years), years of education and time since PD diagnosis (years)
PIMS	EFA identified 4 factors (variance explained = 78%)	–	On/Off, dose of tolcapone, H&YS and presence of fluctuations	UPDRS (parts 1 to 4 and total), S&E, age (years), duration of PD (years), duration of the treatment with levodopa (years), daily dose of levodopa (mg), Pfeiffer, HADS-A, HADS-D, SCOPA-SPES (parts A, B, C and total), PDQ-39 and PDQL
QLPD	EFA identified 68 items grouped in 8 factors (variance explained = 80.3%; eigenvalue > 1), and after Varimax Orthogonal Rotation, 41 items were grouped in 7 factors (variance explained): Motor (46%), Finances (10%), Fear and Social (6%), Psychological (5%), Nonmotor symptoms (4%), Treatment (3%) and Family (3%) CFA confirmed that 45 items were grouped in 9 domains	–	Reported QoL in the visual analogue scale (included in QLPD) and H&YS	H&YS, age at diagnosis of PD, duration of the PD symptoms (years), monthly earnings, equivalent daily dose of levodopa (mg), PDQ-39 and SF-36
QLSM-DBS	EFA identified 5 factors (range of factor loadings): Reliability of the neurostimulator (0.70–0.85); Inconspicuousness of the neurostimulator (0.96–0.97); Independent handling/manipulation of the neurostimulator (0.72–0.93); Medical care (0.63–0.91); and Absence of bodily symptoms/side effects of the neurostimulation (0.59–0.82)	–	–	SF-36 (Physical and Mental components), EQ-5D utility values, EQ-VAS, QLSM-A (general satisfaction), QLSM-G (health satisfaction) and QLSM-MD
QLSM-MD	EFA identified 12 factors (range of factor loadings): Controllability/fluidity of movement (0.73–0.89); Absence of dizziness/steadiness when standing and walking (0.65–0.81); Hand dexterity throughout the day (0.73–0.87); Articulation/fluency of speech (0.93–0.94); Ability to swallow (single item); Absence of false bodily sensations (0.81); Bladder/intestinal function (0.76–0.77); Sexual excitability (0.54–0.89); Undisturbed sleep (0.86); Memory/clear thinking (0.60–0.73); Independence from help (0.54–0.94); Inconspicuousness of illness (0.75–0.87)	–	–	SF-36 (Physical and Mental components), EQ-5D utility values, EQ-VAS, QLSM-A (general satisfaction), QLSM-G (health satisfaction) and QLSM-DBS
QoLQ-PwP	EFA identified 10 factors	–	–	PDQ-39 and PDQ-8
QOLSQ	–	–	Patients with PD vs. healthy controls	–
Unspecific PROMs validated for PD				
15D	–	–	Patients with PD vs. healthy participants and patients with PD grouped by H&YS, presence of motor fluctuations, dementia and depression	PDQ-39, PDQ-8; EQ-VAS, UPDRS-Motor, UPDRS-ADL, tremor, stiffness, hypokinesia, postural instability and gait disturbances
EQ-5D-3L	–	–	Presence of depression, falls, postural instability, dyskinesia, perception of health state changes compared with the previous year, MMSE (cutoff score of 25 point) and H&YS	H&YS, S&E, UPDRS (Parts I to IV), duration of PD (years), BDI, MMSE, PDQ-39, PDQ-8, SF-36, Carer EQ-5D-3L, Carer EQ-VAS and Zarit Scale (burden of caregivers)
EQ-5D-5L	–	–	H&YS, gender, level of studies, work status, treatment with levodopa, duration of PD (years), EQ-VAS scores, presence of dyskinesia, wearing off, freezing, postural instability, cognitive impairment and depression	PDQ-8, EQ-VAS, UPDRS (Parts I to IV and total score), duration of PD (years) and H&YS
EQ-VAS	–	–	Presence of dyskinesia, wearing-off, freezing, postural instability, cognitive impairment, depression, falls, perception of health state changes compared with the previous year, MMSE (cutoff score of 25 point) and H&YS	PDQ-39, PDQ-8, EQ-VAS, UPDRS (Parts I to IV and total score), duration of PD (years), H&YS, SF-36, S&E, BDI and MMSE
McGill QOL	Factor analysis with oblique (Promax) rotation identified 3 factors (variance explained = 54%)	–	Patients receiving palliative care vs. patients without palliative care	PDQ-39, PROMIS-29, QOL-AD, ESAS-PD and HADS-D
Neuro-QOL	–	–	H&YS (Stages I/II vs. Stages III/IV)	PDQ-39, UPDRS (Parts I to III and Total), MoCA, PHQ-9, Barthel Index, Lawton IADL scale, symbol search (raw score), oral symbol digit modalities, digit symbol coding (Correct number), PROMIS (global physical and global mental), EQ-5D (utility) and global HRQoL
PGI	–	–	–	EQ-5D-5L, SF-6D, HUI and PDQ-8
PROMIS-29	Factor analysis with oblique (Promax) rotation identified 2 (variance explained = 47%) and 6 factors (variance explained = 73%)	–	Patients receiving palliative care vs. patients without palliative care	PDQ-39, McGill QOL, QOL-AD, ESAS-PD and HADS-D
QOL-AD	Factor analysis with oblique (Promax) rotation identified 3 factors (variance explained = 57%)	–	Patients receiving palliative care vs. patients without palliative care	PDQ-39, PROMIS-29, McGill QOL, ESAS-PD and HADS-D
SF-12	–	Rasch model exhibited good fit statistics for items (residuals of fit), participants (residuals of fit) and item-treat interaction (Chi-squared), and revealed good reliability (Person Separation Index, PSI), targeting (location of participants) and one-dimensionality (eigenvalues = 1.57–2.67; variance explained = 22.2%−36.0%; and proportion of different person location significative)	–	–
SF-36	CFA confirmed 8 domains (eigenvalues > 1; variance explained = 68.43%; Chi-squared = 155.89 [p < 0.0001]; GFI = 0.87 [adjusted GFI = 0.72]; CFI = 0.82; RMSEA = 0.21 [95CI = 0.18–0.24]): 1) Physical functioning (eigenvalue = 0.72); 2) Role physical (eigenvalue = 0.80); 3) Pain (eigenvalue = 0.67); 4) General health (eigenvalue = 0.77); 5) Energy (eigenvalue = 0.87); 6) Social functioning (eigenvalue = 0.79); 7) Role emotional (eigenvalue = 0.72); and 8) Mental health (eigenvalue = 0.86)	–	Presence of depression (BDI ≥ 9), cognitive impairment (MMSE < 25), self-declared falls, postural instability and perception of health changes compared with the previous year	H&YS, S&E, UPDRS (Motor), duration of PD (years), BDI, MMSE, EQ-5D-3L and EQ-VAS
SF-6D	–	–	H&YS	PDQ-8 and EQ-VAS
WHO-5	–	–	–	BDI, UPDRS (Total, Parts 3 and 4) and H&YS
WHOQOL-BREF	–	–	Patients with PD vs. healthy controls	Age, years of education, duration of PD (years), daily dose of levodopa (mg), UPDRS (Motor), MMSE, DEX, BDI, LSAS, AES, PDFS, daily physical activities and cognitive ADL

^A^ List of abbreviations is provided in Online Resource 11

With respect to the reliability of the 29 PROMs resulting from the SLR (Table 4), data on internal consistency (Cronbach’s alpha), temporal stability (ICC), and ME were available for 23 (79.3%, Online Resource 8), 11 (37.9%, Online Resource 9), and 4 (13.8%, Online Resource 10) instruments, respectively. Complete evidence for reliability, including internal consistency, time stability, and ME, was available for 4 PROMs (13.8%): PDQ-39, PDQ-8, Parkinson’s Disease Quality of Life Questionnaire (PDQL) and Neuro-QOL.

**Table 4:** Reliability of PROMs for measuring QoL in PwPD

PROMs ^A^	Internal consistency (Online Resource 8) [Cronbach’s alpha]		Test–retest (Online Resource 9) [ICC]		ME (Online Resource 10)	
	Scale	Subscales	Scale	Subscales	Scale	Subscales
Specific PROMs for PD						
Bela-P-K (Bb)	0.90–0.91	0.61–0.83	Test–retest scores differ for 10 items	–	–	–
Bela-P-K (Nfh)	0.90–0.93	0.73—0.88	Test–retest scores differ for 10 items	–	–	–
Indo-PDQOL	0.95	0.61–0.91	–	–	–	–
OFFELIA	–	0.89–0.94	–	–	–	–
PDQ-39	0.78–0.97	0.13–0.98	0.74–0.92	0.47–0.96	3.63–7.80	6.02–32.96
PDQ-39 (Proxy)	0.94	0.71–0.92	–	–	–	–
PDQ-8	0.72–0.88	–	0.72–0.97	–	1.96 (SD = 3.96)	–
PDQ-DAT	–	0.84–0.86	–	–	–	–
PDQL	0.92–0.98	0.65–0.98	0.95	0.65–0.96	6.31 (SD = 22.85)	–
PDQoL7	0.80	–	–	–	–	–
PDQUALIF	0.89	0.58–0.85	0.88	0.70–0.86	–	–
PIMS	0.90–0.87		0.83–0.98	0.91–0.98 ^B^	–	–
QLPD	–	0.42–0.83	–	–	–	–
QLSM-DBS	0.69–0.73	0.34–0.61	0.97	–	–	–
QLSM-MD	0.83–0.92	0.36–0.74	0.87	–	–	–
QoLQ-PwP	0.95	–	–	–	–	–
QOLSQ	0.90	0.69–0.95	0.99	0.98–0.99	–	–
Unspecific PROMs validated for PD						
15D	–	–	–	–	–	–
EQ-5D-3L	–	–	–	–	–	–
EQ-5D-5L	0.828	–	–	–	–	–
EQ-VAS	–	–	–	–	–	–
McGill QOL	0.88	–	–	–	–	–
Neuro-QOL	–	0.81–0.94	–	0.66–0.80	–	2.07–4.42
PGI	–	–	–	–	–	–
PROMIS-29	0.93	–	–	–	–	–
QOL-AD	0.83	–	–	–	–	–
SF-12	–	–	–	–	–	–
SF-36	–	0.67–0.95	–	0.71–0.89	–	–
SF-6D	–	–	–	–	–	–
WHO-5	0.83	–	–	–	–	–
WHOQOL-BREF	–	0.64–0.85	–	–	–	–

^A^ List of abbreviations is provided in Online Resource 11

^B^ Psychometric property assessed for items

## Discussion

In a healthcare paradigm that is shifting from a disease-centered approach to a person-based approach, PROMs are key instruments used to empower patients with respect to their disease, and to understand their own perceptions of health and well-being. However, these questionnaires need to be feasible, valid, and reliable [22]. Thus, the present study provides an exhaustive review of QoL measures for PD, whose psychometric properties were critically evaluated via a standardized method based on COSMIN statements [25–27].

A total of 29 different PROMs were identified, 15 of which were specific to PD. The remaining PROMs were generic or specific for other diseases but were validated for PwPD. After the COSMIN-based critical assessment, 6 tools had the potential to be the most suitable PROM for measuring QoL in PwPD (evidence-based recommendation: A). These questionnaires included the PDQ-39, PDQ-8, PDQL, PDQUALIF, PIMS, and Neuro-QOL. The remaining instruments proved to be feasible, valid, and reliable, thus being susceptible to being recommended in PD but requiring further studies to ensure their good metric properties (evidence-based recommendation: B).

The PDQ-39 represents the PROM whose psychometric properties have been most evaluated [46–87]. This 39-item instrument was first developed in 1995 [46] and measures the frequency of both PD-specific and generic symptoms during the previous month [46–87]. Although the impacts of work, financial conditions, treatment, and facing the disease are not measured in the PDQ-39, the tool also asks for several emotional and psychological disturbances, as well as for the patient-perceived stigma in relation to PD [46–87]. According to these items, the PDQ-39 is an appropriate measure of SWB that measures the patient satisfaction with life, expectations for the future and a sense of fulfillment in life [10]. Nonetheless, two main constraints were observed in relation to the PDQ-39. First, there is a lack of items measuring both the impact of the treatments and work impairment on patients’ QoL [46–87]. Additionally, internal consistency was poor when measured by the PROM’s domains (Cronbach’s alpha for social support decreased to 0.13) [57].

Given the extension of the PDQ-39, a reduced version was developed in 1997 containing 8 items (one per dimension), which was named as the PDQ-8 [47]. The psychometric properties of this short version have been proven in numerous studies [48, 59, 64, 73, 77, 78, 86, 88–96], and given its length, it could represent a more feasible tool for some patients. Nonetheless, QoL is a complex construct with several dimensions. Thus, when the PDQ-8 is compared with the PDQ-39, a decrease in sensitivity when detecting PD-related changes could be expected, and other properties such as content validity or internal consistency, could also be affected. Given that the PDQ-8 is the reduced version of the PDQ-39, it has the same limitations described for the 39-item version in terms of content.

The PDQL is another alternative for measuring QoL in PwPD. Although few studies assessed its psychometric properties, this PROM has proven to be feasible, valid, and reliable [65, 69, 81, 98–101]. The PDQL, which was first published in 1996 [98], is a 37-item instrument measuring the frequency of symptoms in PwPD during the last 3 months. Although the questionnaire includes four domains, its dimensionality has not been studied. The lack of evidence on its structural validity relies on the results of the Kaiser–Meyer–Olkin (KMO) test, which determines the suitability of data for conducting factorial analyses (KMO estimate = 0.461; *p* < 0.05; KMO < 0.5 is indicative of poor shared variance indicating that EFA/CFA is inappropriate) [101]. As was observed in the PDQ-39 and PDQ-8, the PDQL represents an adequate measure for SWB, as it measures the impact of emotional disturbances and fears when facing PD. However, the main limitation of this tool is its known-group validity, which was assessed comparing scores by disease severity and the Hoehn & Yahr scale (H&YS) [65, 69, 81, 98–101]. Although these groups are clinically relevant, additional evidence for the ability of PDQL scores to differentiate between interest groups would be desirable (e.g., sex, levodopa dose, presence of symptoms, etc.). Moreover, as was observed for the PDQ-39, the PDQL lacks items measuring how the treatment of PD and work impairment impacts quality of life [65, 69, 81, 98–101].

Another PROM measuring SWB in PwPD is the PDQUALIF, a 33-item questionnaire developed in 2003 [102]. In contrast with the previously described tools, the PDQUALIF asks for the score that best describes the patient’s situation in relation to the PD [102]. In the present SLR, only one psychometric study was identified, which revealed that PDQUALIF is feasible, valid, and reliable [102]. However, its use should be restricted to the context in which it was developed (the United States and Canada), until further studies and cross-cultural adaptations of the instrument are conducted. Additionally, know-group validity was assessed only for groups determined by the H&YS [102], making it necessary to demonstrate this property by considering other groups. Finally, the PDQUALIF lacks items measuring cognitive decline, treatment of PD and work impairment [102], which means that its use is inappropriate if there is interest in knowing how these factors impact the patient’s QoL.

The PIMS provides an alternative for measuring SWB in PD, with a focus on the patients’ social life, relationships, work, and self-esteem [69, 103, 104]. This instrument was first developed for a clinical study purposes [103], and includes ten items measuring the patient perceived impact of each one [69, 103, 104]. As was the case for PDQUALIF, further psychometric studies are needed to ensure the adequacy of using the PIMS in other contexts. Although the PIMS was found to be feasible, valid, and reliable [69, 103, 104], its design relies on healthcare professionals’ experience without involving patients during the items selection [103]. Thus, no items were included in the PIMS measuring ADL, cognition, symptoms of PD, treatment of PD, or emotional and psychological disturbances, impairing its content validity [103].

According to the findings of the present SLR, the Neuro-QOL is another adequate tool for measuring QoL in PwPD. Although it is a generic PROM designed for multiple neurological conditions, it has proven to be feasible, valid, and reliable when administered in patients with PD [114, 115]. Strictly speaking, the evidence for the use of Neuro-QOL in patients with PD comes from a single study in the United States [114, 115]. Although the questionnaire has been adapted for use in different languages, additional studies in patients with PD would be desirable. Moreover, the Neuro-QOL is a generic tool; therefore, it does not include some specific aspects of PD that impair patients’ QoL (e.g., some movement-related symptoms such as freezing and dysphagia) [114, 115].

Among these 6 PROMs, the PDQ-39 is the questionnaire whose psychometric properties have been studied most frequently. Although it could be thought to be the most appropriate PROM to use at present, its extension impairs the feasibility of administering the tool in some contexts (e.g., elderly patients or those with cognitive impairment who may find it difficult to answer so many questions, limited consultation time, etc.). In these circumstances, the PDQ-8, PDQL, PDQUALIF, PIMS, and Neuro-QOL represent adequate alternatives in psychometric terms.

In addition to these 6 instruments, another 23 PROMs were identified for measuring QoL in PD. All these instruments have proven to be feasible, valid, and reliable tools for measuring QoL in PwPD in several contexts. Thus, the 23 remaining PROMs have the potential to be recommended for use in PwPD, which is subject to further psychometric studies. Specifically, it would be advisable to study the ME in depth, as only 4 out of 29 PROMs had information available on this subject. Likewise, although some studies compared the validity and reliability estimates with those of other studies, no formal assessments of cross-cultural validity were found, highlighting the need to study this property.

Most of these QoL questionnaires were designed for measuring SWB, as these PROMs include items measuring disease-related fears, stigma, or other aspects related to the patients’ satisfaction with life [10], with the Parkinson’s Disease Quality of Life 7-item (PDQoL7) being the unique identified specific tool measuring HRQoL in PwPD [96]. Although HRQoL considers that quality of life depends only on the absence of pathology, which may be a simplistic approach to the concept of QoL compared with SWB [10], it has several advantages. First, while satisfaction with life is a highly heterogeneous construct, because each patient has their own concept of what this means, the presence or absence of symptoms is a more ‘objective’ measure across patients [10]. Additionally, the use of preference-based instruments that measure HRQoL allows the estimation of utility values, which can then be used in cost-effectiveness analyses [10]. Currently, SWB measures are inappropriate for estimating utility values, mainly because of the high interpatient variability previously described [10].

Another constraint of the identified QoL measures for PD identified in the present study is the date when most PROMs were developed. For example, the PDQ-39, PDQ-8, and PDQL were developed almost 30 years ago [46, 47, 98]. Changes in both therapeutics and patients’ unmet needs have occurred since the 1990s, but these QoL measures maintain the same items and structure as their original publication did [46, 47, 98]. Even though content validity was only assessed qualitatively due to the lack of any index of this property in the psychometric studies identified, it was observed that none of the PROMs included all the domains described by the patients. This suggests the potential need to develop new instruments or, alternatively, to combine existing PROMs to assess all aspects that matter to patients. Additionally, measuring patient’s experience using validated patient-reported experience measures (PREMs) will be key to optimizing the care of PwPD.

In this context, it should be noted that QoL is a multidimensional concept [10]; thus, the currently available QoL measures for PD combine items assessing most of the aspects impacting QoL. However, given that PD is a complex disease, it is recommended that QoL measures be administered with the other questionnaires to obtain a precise view of the disease’s impact. These complementary instruments may include measures of motor [123, 124] and/or nonmotor symptoms [125–130], impact on ADL [131, 132], or cognitive impairment [133].

The last limitation of the available QoL measures for PwPD was the lack of normative values or population norms published for any instrument. These normative values are key to ensure a proper interpretation of the PROMs’ scores, and allow the comparison of results in a clinical setting [134]. However, this limitation should be interpreted with caution as this type of publication could have been excluded from the search strategy used. Additionally, according to the COSMIN guidelines, the lack of normative data does not represent a reason to discourage the use of any PROM [25–27].

Previous SLRs of QoL measures in PwPD were conducted before the present study [12–21]. However, to the authors’ knowledge none of these summarized psychometric properties of the identified PROMs or used the COSMIN approach to critically assess validity and reliability [25–27]. Other strengths that should be highlighted are the wide search strategy, which allowed the identification of all PROMs in PD, and the design of the literature search strategy based on both the PRISMA [23] and the COSMIN [24] statements. Moreover, previously conducted SLRs of PROMs in PwPD were reviewed to retrieve additional studies that could not be identified based on the search strategy. The number of studies gathered from these SLRs was residual, revealing the robustness of the designed strategy.

Nonetheless, this study has several limitations. First, the critical evaluation of psychometric properties was constrained to those described in the COSMIN guidelines [24]; thus, other measures that could provide additional evidence were discarded (e.g., McDonald’s Omega). Additionally, psychometric properties requiring longitudinal studies for their assessment were not considered. The rationale for this decision relies on the fact that evidence in this regard is usually presented as a secondary outcome of clinical studies. Thus, a search strategy considering each PROM’s name, which was uncertain before conducting the screening of reference and eligibility, would be needed. Further research will be needed to synthesize the available evidence concerning the instruments’ sensitivity to change or predict validity, among other factors.

Another constraint of the study was the synthesis of psychometric properties relying on descriptive information instead of providing meta-analyzed estimates. Although the COSMIN guidelines consider estimating pooled effects, this is not a mandatory requirement; rather, providing the range of values identified in the different studies is also recommended [24]. Moreover, when cross-cultural adaptations of existing questionnaires are conducted, both the translation and the cultural context could impact on the properties of the tool [135]. Thus, meta-analysis techniques were considered more appropriate for studies limited to a single language version of the PROMs. In contrast, given that the present SLR identified studies involving patients from different regions worldwide, providing ranges of values from literature was preferable.

The evaluation of certain psychometric properties, mainly content validity, without involving patients in the research would represent another limitation. This approach could be helpful to understanding whether PROMs capture those aspects that most affect patients’ QoL. Nonetheless, patient participation is especially important during the design of the PROM’s conceptual framework to understand how PD affects patients’ quality of life and not only for measuring the PROM’s performance [136, 137]. In the present SLR, 12 out of the 15 PD-specific QoL measures involved a group of patients during the item generation phase of development [43, 45–47, 96–98, 102, 105, 106, 108, 109], ensuring that the items included in the PROMs reflect those aspects most relevant for them.

Finally, although several databases were considered for retrieving potential references of interest (PubMed, Embase, Scopus, WoS, etc.), the search was restricted to reports in English and Spanish, and some cross-cultural adaptations of PROMs may be missing. Even though the present study provides an exhaustive synthesis of the feasibility, validity, and reliability of the currently available QoL measures for PD, it is recommended that context-specific reviews be conducted before the use of any of these instruments to guarantee their adequacy for use.

## Conclusion

In conclusion, QoL measures are key to moving toward a person-centered approach in health sciences. In the present SLR, up to 29 PROMs were identified to be currently available, feasible, valid, and reliable for measuring QoL in PwPD, including the PDQ-39, PDQ-8, or PDQL. However, further research will be needed to ensure their good psychometric properties for the use of them in both clinical practice and biomedical research, as well as additional cross-cultural adaptations of existing tools to use them in several languages and contexts.

## Supplementary Information

Below is the link to the electronic supplementary material.

Supplementary file1 (DOCX 121 KB)

Supplementary file2 (DOCX 61 KB)

Supplementary file3 (DOCX 62 KB)

Supplementary file4 (DOCX 46 KB)

Supplementary file5 (DOCX 183 KB)

Supplementary file6 (DOCX 116 KB)

Supplementary file7 (DOCX 134 KB)

Supplementary file8 (DOCX 152 KB)

Supplementary file9 (DOCX 174 KB)

Supplementary file10 (DOCX 125 KB)

Supplementary file11 (DOCX 122 KB)

## Data Availability

All data used for conducting the present study are publicly available or presented in the Online Resources.
