# Supplementary material for: Quality of life measures in Parkinson’s disease: a systematic literature review of patient-reported outcomes measures (PROMs) and their psychometric properties
Source: J Neurol. 2025 Aug 28;272(9):598. doi: 10.1007/s00415-025-13348-x (PMC12394374; doi:10.1007/s00415-025-13348-x)
Supplement: Supplementary file 1 — Supplementary file1 (DOCX 121 KB) [file 415_2025_13348_MOESM1_ESM.docx]

**Quality of Life Measures in Parkinson’s Disease: A Systematic Literature Review of Patient-Reported Outcomes Measures (PROMs) and their Psychometric Properties**

**– ONLINE RESOURCE 10 –**

Table S16. Descriptions of the studies’ findings in relation to the ME of the PROMs.

| Eligible study | Sample size | Findings of the study in relation to ME | COSMIN assessment | |
| --- | --- | --- | --- | --- |
|  |  |  | **RoB** | **Good property** |
| Spliethoff-Kamminga (2003) [1] | – | – | – | – |
| Ortelli (2017) [2] | – | – | – | – |
| Bayen (2021) [3] | – | – | – | – |
| Aggarwal (2013) [4] | – | – | – | – |
| Kuharic (2022) [5] | – | – | – | – |
| Kuharic (2024) [6] | – | – | – | – |
| Peto (1995) [7] | – | – | – | – |
|  | – | – | – | – |
| Jenkinson (1997) [8] | – | – | – | – |
|  | – | – | – | – |
| Jenkinson (1997) [9] | – | **PDQ-39:**  – | – | – |
|  | – | **PDQ-8:**  – | – | – |
| Martínez-Martín (1998) [10] | – | – | – | – |
| Bushnell (1999) [11] | – | – | – | – |
| Andreu (2000) [12] | – | – | – | – |
| Schrag (2000) [13] | – | **PDQ-39:**  – | – | – |
|  | – | **EQ-5D-3L:**  – | – | – |
|  | – | **EQ-VAS:**  – | – | – |
|  | – | **SF-36:**  – | – | – |
| Katsarou (2001) [14] | – | – | – | – |
| Peto (2001) [15] | – | – | – | – |
| Tsang (2002) [16] | – | – | – | – |
| Hagell (2003) [17] | – | – | – | – |
| Jenkinson (2003) [18] | – | – | – | – |
|  | – | – | – | – |
|  | – | – | – | – |
|  | – | – | – | – |
|  | – | – | – | – |
| Park (2004) [19] | – | – | – | – |
| Tan (2004) [20] | – | – | – | – |
| Fitzpatrick (2004) [21] | 726 | SEM = 1 x SEM: T1 / T2 / S_diff_ = √ (SEM1^2^ + SEM2^2^)   - Mobility: 6.17 / 6.02 / 8.55 - ADL: 8.68 / 8.33 / 12.03 - Emotional wellbeing: 7.58 / 7.46 / 10.64 - Stigma: 10.62 / 10.71 / 15.08 - Social support: 14.36 / 16.82 / 22.11 - Cognition: 11.46 / 11.37 / 16.15 - Communication: 10.81 / 11.05 / 15.46 - Bodily discomfort: 13.17 / 12.57 / 18.20 - SI: 3.98 / 3.63 / 5.39   SEM = 1.96*SEM: T1 / T2 / S_diff_ = √ (SEM1^2^ + SEM2^2^)   - Movilidad: 11.89 / 11.80 / 16.75 - Mobility. Cotidianas: 17.01 / 16.32 / 23.57 - Emotional wellbeing: 14.86 / 14.62 / 20.85 - Stigma: 20.82 / 21.00 / 29.57 - Social support: 28.15 / 32.96 / 43.34 - Cognition: 22.47 / 22.29 / 31.65 - Communication: 21.19 / 21.65 / 30.30 - Bodily discomfort: 25.82 / 24.63 / 35.68 - SI: 7.80 / 7.11 / 10.56 | Very good | (+) |
|  | 132 | SEM = 1 x SEM: T1 / T2 / S_diff_ = √ (SEM1^2^ + SEM2^2^)   - Mobility: 6.79 / 6.10 / 9.13 - ADL: 8.82 / 8.32 / 12.12 - Emotional wellbeing: 7.24 / 6.75 / 9.89 - Stigma: 10.99 / 10.95 / 15.51 - Social support: 9.31 / 9.82 / 13.31 - Cognition: 10.63 / 11.68 / 15.79 - Communication: 10.28 / 10.80 / 14.91 - Bodily discomfort: 12.24 / 11.98 / 17.13 - SI: 3.80 / 3.71 / 5.31   SEM = 1 x SEM: T1 / T2 / S_diff_ = √ (SEM1^2^ + SEM2^2^)   - Mobility: 13.30 / 11.96 / 17.89 - ADL: 17.28 / 16.29 / 23.75 - Emotional wellbeing: 14.18 / 13.22 / 19.39 - Stigma: 21.54 / 21.46 / 30.40 - Social support: 18.24 / 18.65 / 26.09 - Cognition: 20.83 / 22.89 / 30.95 - Communication: 20.14 / 21.17 / 29.22 - Bodily discomfort: 24.00 / 23.48 / 33.58 - SI: 7.45 / 7.27 / 10.41 | Very good | (+) |
| Haapaniemi (2004) [22] | – | – | – | – |
| Martínez-Martín (2004) [23] | – | – | – | – |
| Ma (2005) [24] | – | – | – | – |
| Luo (2005) [25] | – | – | – | – |
| Martínez-Martín (2007) [26] | – | **PDQ-39:**  – | – | – |
|  | – | **PDQL:**  – | – | – |
| Hagell (2007) [27] | – | – | – | – |
| Krikmann (2008) [28] | – | – | – | – |
| Marinus (2008) [29] | – | – | – | – |
| Serrano-Dueñas (2008) [30] | – | **PDQ-39:**  – | – | – |
|  | – | **PDQL:**  – | – | – |
| Žiropađa (2009) [31] | – | – | – | – |
| Nojomi (2010) [32] | – | – | – | – |
| Luo (2010) [33] | – | – | – | – |
| Huang (2010) [34] | – | **PDQ:39:**  – | – | – |
|  | – | **PDQ-8:**  – | – | – |
| Zhang (2011) [35] | – | – | – | – |
| Kwon (2013) [36] | – | – | – | – |
| Park (2014) [37] | – | – | – | – |
| Fereshtehnejad (2014) [38] | – | **PDQ:39:**  – | – | – |
|  | – | **PDQ-8:**  – | – | – |
| Krygowska-Wajs (2015) [39] | – | **PDQ:39:**  – | – | – |
|  | – | **PDQ-8:**  – | – | – |
| Morley (2015, a) [40] | – | – | – | – |
| Morley (2015, b) [41] | – | – | – | – |
| Jesus-Ribeiro (2017) [42] | – | **PDQ-39:**  – | – | – |
|  | – | **PDQL:**  – | – | – |
| Galeoto (2018) [43] | – | – | – | – |
| Suratos (2018) [44] | – | – | – | – |
| Holden (2019) [45] | – | **PDQ-39:**  – | – | – |
|  | – | **McGill QOL:**  – | – | – |
|  | – | **PROMIS-29:**  – | – | – |
|  | – | **QOL-AD:**  – | – | – |
| Nelson (2020) [46] | – | – | – | – |
| Kim (2020) [47] | – | **PDQ:39:**  – | – | – |
|  | – | **PDQ-8:**  – | – | – |
| Hanff (2023) [48] | – | – | – | – |
| Katsarou (2004) [49] | – | – | – | – |
| Tan (2007) [50] | – | – | – | – |
|  | – | – | – | – |
|  | – | – | – | – |
| Jenkinson (2007) [51] | – | – | – | – |
|  | – | – | – | – |
|  | – | – | – | – |
|  | – | – | – | – |
|  | – | – | – | – |
| Franchignoni (2008) [52] | – | – | – | – |
|  | – | – | – | – |
|  | – | – | – | – |
| Dal Bello-Haas (2009) [53] | 24 | Estimated SEM (SD) for PDQ-8 = 1.96 (3.84) | Very good | (+) |
| Alvarado-Bolaños (2015) [54] | – | **EQ-5D-5L**  – | – | – |
|  | – | **EQ-VAS**  – | – | – |
| Kahraman (2018) [55] | – | – | – | – |
| Ramadhan (2022) [56] | – | **PDQ-8:**  – | – | – |
|  | – | **EQ-5D-3L:**  – | – | – |
| Stathis (2022) [57] | – | **PDQ-8:**  – | – | – |
|  | – | **PDQoL-7:**  – | – | – |
| Kawaguchi (2021) [58] | – | – | – | – |
| De Boer (1996) [59] | – | – | – | – |
| Serrano-Dueñas (2004) [60] | 137 | Estimated SEM for PDQL (based on Cronbach’s alpha = 0.92) = 6.31 (SD = 22.85) | Very good | (?) |
| Campos (2011) [61] | – | – | – | – |
| Dereli (2015) [62] | – | – | – | – |
| Welsh (2003) [63] | – | – | – | – |
| Calne (1996) [64] | – | – | – | – |
| Schulzer (2003) [65] | – | – | – | – |
| Aggarwal (2020) [66] | – | – | – | – |
| Kuehler (2003) [67] | – | **QLSM-DSB:**  – | – | – |
|  | – | **QLSM-MD:**  – | – | – |
| Krygowska-Wajs (2015) [68] | – | **QLSM-DSB:**  – | – | – |
|  | – | **QLSM-MD:**  – | – | – |
| Bose (2018) [69] | – | – | – | – |
| Diniz (2018) [70] | – | – | – | – |
| García-Gordillo (2013) [71] | – | **15D:**  – | – | – |
|  | – | **EQ-5D-5L:**  – | – | – |
| Del Pozo-Cruz (2018) [72] | – | **15D:**  – | – | – |
|  | – | **SF-6D:**  – | – | – |
| Luo (2009) [73] | – | **EQ-5D-3L:**  – | – | – |
|  |  | **EQ-VAS:**  – | – | – |
|  | – | **EQ-5D-3L:**  – | – | – |
|  |  | **EQ-VAS:**  – | – | – |
|  | – | **EQ-5D-3L:**  – | – | – |
|  |  | **EQ-VAS:**  – | – | – |
| Garcia-Gordillo (2015) [74] | – | **EQ-5D-3L:**  – | – | – |
|  | – | **SF-6D:**  – | – | – |
| Nowinski (2010) [75] | – | – | – | – |
| Nowinski (2016) [76] | 120 | Estimated SEM for Neuro-QOL domains:   - Positive Affect and Well-Being = 3.69 - Applied Cognition–General Concerns = 4.03 - Applied Cognition–Executive Function = 3.95 - Lower Extremity Function–Mobility = 3.50 - Upper Extremity Function–Fine Motor, ADL = 4.42 - Ability to Participate in Social Roles and Activities = 3.66 - Satisfaction with Social Roles and Activities = 3.26 - Depression = 3.86 - Anxiety = 3.25 - Stigma = 2.07 - Fatigue = 3.63 - Sleep Disturbance = 3.62   Emotional and Behavioral Dyscontrol = 4.35 | Doubtful | (?) |
| Kuspinar (2019) [77] | – | – | – | – |
| Kuspinar (2020) [78] | – | – | – | – |
| Hagell (2011) [79] | – | – | – | – |
| Steffen (2008) [80] | – | – | – | – |
| Hagell (2008) [81] | – | – | – | – |
| Schneider (2010) [82] | – | – | – | – |
| Hendred (2016) [83] | – | – | – | – |
