# Supplementary material for: Quality of life measures in Parkinson’s disease: a systematic literature review of patient-reported outcomes measures (PROMs) and their psychometric properties
Source: J Neurol. 2025 Aug 28;272(9):598. doi: 10.1007/s00415-025-13348-x (PMC12394374; doi:10.1007/s00415-025-13348-x)
Supplement: Supplementary file 2 — Supplementary file2 (DOCX 61 KB) [file 415_2025_13348_MOESM2_ESM.docx]

**Quality of Life Measures in Parkinson’s Disease: A Systematic Literature Review of Patient-Reported Outcomes Measures (PROMs) and their Psychometric Properties**

**– ONLINE RESOURCE 11 –**

**LIST OF ABBREVIATIONS**

| **15D** | Fifteen Dimensions Questionnaire |
| --- | --- |
| **95CI** | 95% Confidence Interval |
| **AAN** | American Academy of Neurology |
| **ADL** | Activities of Daily Living |
| **AES** | Apathy Evaluation Scale |
| **AMN** | *Academia Mexicana de Neurología* |
| **ANS** | Australasian Neuroscience Society |
| **Bb** | Bothered by |
| **BDI** | Beck Depression Inventory |
| **Bela-P-K** | *Belastungfragebogen Parkinson Kurzversion* |
| **BMI** | Body Mass Index |
| **CDR** | Clinical Dementia Rating |
| **CES-D** | Center for Epidemiologic Studies – Depression scale |
| **CFA** | Confirmatory Factor Analysis |
| **CFI** | Comparative Fit Index |
| **ClinRO** | Clinician-Reported Outcome |
| **CNS** | Canadian Neurological Society |
| **COOP** | Dartmouth Primary Care Cooperative Information Project |
| **COSMIN** | COnsensus-based Standards for the selection of health Measurement Instruments |
| **CTT** | Classical Test Theory |
| **DA** | Dopamine |
| **DEX** | Dysexecutive Questionnaire |
| **DIF** | Different Item Functioning |
| **EAN** | European Society of Neurology |
| **EFA** | Exploratory Factor Analysis |
| **EQ-5D-3L** | EuroQol 5 Dimensions 3 Levels |
| **EQ-5D-5L** | EuroQol 5 Dimensions 5 Levels |
| **EQ-VAS** | EuroQol Visual Analogue Scale |
| **ESAS-PD** | Edmonton Symptom Assessment System Scale for Parkinson’s Disease |
| **ESS** | Epworth Sleepiness Scale |
| **FAB** | Fullerton Advanced Balance |
| **GDS** | Geriatric Depression Scale |
| **GFI** | Goodness of Fit |
| **HADS-A** | Hospital Anxiety and Depression Scale – Anxiety |
| **HADS-D** | Hospital Anxiety and Depression Scale – Depression |
| **HRQoL** | Health-Related Quality of Life |
| **HUI** | Health Utilities Index |
| **H&YS** | Hoehn & Yahr Scale |
| **IADL** | Instrumental Activities of Daily Living |
| **ICC** | Intraclass Correlation Coefficient |
| **IEXP** | *Instituto de Experiencia del Paciente* |
| **IFI** | Incremental Fit Index |
| **Indo-PDQOL** | Health-related quality of life instrument for Hindi speaking Parkinson's disease patients |
| **IPA-I** | Impact on Participation and Autonomy questionnaire |
| **IPMDS** | International Parkinson and Movement Disorder Society |
| **IQR** | Interquartile Range |
| **IRT** | Items Response Theroy |
| **ISPOR** | International Society of Pharmacoeconomics and Outcome Research |
| **KMO** | Kaiser-Meyer-Orkin |
| **LoA** | Limit of Agreement |
| **LSAS** | Liebowitz Social Anxiety Scale |
| **MA** | Meta-Analysis |
| **MADRS** | Montgomery-Asberg Depression Rating Scale |
| **McGill QOL** | McGill Quality Of Life Questionnaire |
| **MCS** | Mental Component Summary |
| **MDC** | Minimal Detectable Change |
| **MDS** | Movement Disease Society |
| **ME** | Measurement Error |
| **MIC** | Minimally Important Change |
| **MMSE** | Minimental State Examination |
| **MoCA** | Montreal Cognitive Assessment |
| **MOS-24** | Mean Opinion Scale 24-items |
| **Neuro-QOL** | Neurology Quality of Life |
| **Nfh** | Need for help |
| **NFI** | Normed Fit Index |
| **NHP** | Nottingham Health Profile |
| **NMSQ** | Non-Motor Symptoms Questionnaire |
| **NMSS** | Non-Motor Symptoms Scale |
| **ObsRO** | Observer-Reported Outcome |
| **OFFELIA** | Off Episode Quality of Life Impact Scale |
| **PCS** | Physical Component Summary |
| **PD** | Parkinson’s Disease |
| **PDAQ-15** | Parkinson’s Daily Activities Questionnaire 15-items |
| **PDFS** | Parkinson’s Disease Fatigue Scale |
| **PDQ-39** | Parkinson's Disease Questionnaire 39-item |
| **PDQ-8** | Parkinson's Disease Questionnaire 8-item |
| **PDQ-DAT** | Parkinson's Disease Questionnaire for Device-Aided Therapy |
| **PDQL** | Parkinson's Disease Quality of Life Questionnaire |
| **PDQoL7** | Parkinson's Disease Quality of Life 7-items |
| **PDQUALIF** | Parkinson's Disease Quality of Life Scale |
| **PDSQ** | Parkinson's Disease Screening Questionnaire |
| **PGI** | Patient Generated Index |
| **PHQ-9** | Patient Health Questionnaire 9-items |
| **PIMS** | Parkinson's Impact Scale |
| **PREM** | Patient-Reported Experience Measure |
| **PRISMA** | Preferred Reporting Items in Systematic Reviews and Meta-Analyses |
| **PROM** | Patient-Reported Outcomes Measure |
| **PROMIS-29** | Patient-Reported Outcomes Measurement Information System 29-items |
| **PSQI** | Pittsburgh Sleep Quality Index |
| **PwPD** | People with Parkinson’s Disease |
| **QLPD** | Quality of Life in Parkinson's Disease |
| **QLSM-DBS** | Questions on Life Satisfaction Module - Deep Brain Stimulation |
| **QLSM-MD** | Questions on Life Satisfaction Module – Movement Disorders |
| **QOL** | Quality of Life |
| **QOL-AD** | Quality Of Life in Alzheimer's Disease Scale |
| **QoLQ-PWP** | Quality of Life Questionnaire for People with Parkinson |
| **QOLSQ** | Quality of Life in relation to Swallowing Questionnaire |
| **RMR** | Root Mean square Residual |
| **RMSEA** | Root Mean Square Error of Approximation |
| **RoB** | Risk of Bias |
| **SCOPA** | Scales for Outcomes in Parkinson’s Disease – Motor Function |
| **SD** | Standard Deviation |
| **SDC** | Smallest Detectable Change |
| **SEN** | *Sociedad Española de Neurología* |
| **SEM** | Standard Error of Measurement |
| **SF-12** | Short-Form 12-items |
| **SF-36** | Short-Form 36-items |
| **SF-6D** | Short Form 6 Dimensions |
| **SI** | Summary Index |
| **SIP** | Sickness Impact Profile |
| **SLR** | Systematic Literature Review |
| **SNA** | *Sociedad Neurológica Argentina* |
| **SNU** | *Sociedad de Neurociencias del Uruguay* |
| **SONEPSYN** | *Sociedad de Neurología, Psiquiatría y Neurocirugía de Chile* |
| **SPES** | Short Parkinson's Evaluation Scale |
| **SPMSQ** | Short Portable Mental Status Questionnaire |
| **SRM** | Standardized Response Mean |
| **SRMR** | Standarized Root Mean Residuals |
| **SWB** | Subjective Well-Being |
| **S&E** | Schwab & England Scale |
| **TDQ** | Taiwanese Depression Questionnaire |
| **TLI** | Tucker-Lewis Index |
| **UPDRS** | Unified Parkinson’s Disease Rating Scale |
| **WFN** | World Federation of Neurology |
| **WHO-5** | World Health Organization - Five Well-being Index |
| **WHOQOL-BREF** | World Health Organization Quality of Life - Short Version |
| **WONCA** | World Organization of Family Doctors |
| **WoS** | Web of Science |

**ONLINE RESOURCES REFERENCES**

1. Spliethoff-Kamminga NGA, Zwinderman AH, Springer MP, Roos RAC (2003) Psychosocial problems in Parkinson’s disease: Evaluation of a disease-specific questionnaire. Movement Disorders 18:

2. Ortelli P, Maestri R, Zarucchi M, et al (2017) Italian validation of the Belastungsfragebogen Parkinson kurzversion (BELA-P-k): a disease-specific questionnaire for evaluation of the subjective perception of quality of life in parkinson’s disease. J Clin Mov Disord 4:12. https://doi.org/10.1186/s40734-017-0059-x

3. Bayen S, Heutte J, Vanderbecken J-C, et al (2021) Translation and validation of two Parkinson’s disease-specific burden questionnaires, for patients and caregivers, from German into French. EurJGenPract 27:364–5. https://doi.org/10.1080/13814788.2021.2008352

4. Aggarwal R, Kumar N, Goyal V, et al (2013) Development of health related quality of life instrument for Hindi speaking Parkinson’s disease patients (indo-PDQOL) and initial psychometric testing. Movement Disorders 28:. https://doi.org/10.1002/mds.25605

5. Kuharic M, Shah K, Hanson K, et al (2022) PCR71 Off Episode Quality of Life Impact Scale (OFFELIA): Overview of the Development of a Quality of Life and Functional Impact Measure of Off Episodes in Parkinson’s Disease. Value in Health 25:S403–S404. https://doi.org/10.1016/j.jval.2022.09.2006

6. Kuharic M, Kulbokas V, Hanson K, et al (2024) OFF episode quality of life impact scale (OFFELIA): A new measure of quality of life for off episodes in Parkinson’s disease. Parkinsonism & Related Disorders 123:106070. https://doi.org/10.1016/j.parkreldis.2024.106070

7. Peto V, Jenkinson C, Fitzpatrick R, Greenhall R (1995) The development and validation of a short measure of functioning and well being for individuals with Parkinson’s disease. Qual Life Res 4:241–248. https://doi.org/10.1007/BF02260863

8. Jenkinson C, Fitzpatrick R, Peto V, et al (1997) The Parkinson’s Disease Questionnaire (PDQ-39): development and validation of a Parkinson’s disease summary index score. Age Ageing 26:353–357. https://doi.org/10.1093/ageing/26.5.353

9. Jenkinson C, Fitzpatrick R, Greenhall R, Hyman N (1997) The PDQ-8: Development and validation of a short-term Parkinson’s Disease Questionnaire. Pyschology and Health 12:805–14

10. Martínez-Martín P, Payo BF, and The Grupo Centro for Study of Movement Disorders (1998) Quality of life in Parkinson’s disease: validation study of the PDQ-39 Spanish version. J Neurol 245:S34–S38. https://doi.org/10.1007/PL00007737

11. Bushnell DM, Martin ML (1999) Quality of life and parkinson’s disease: Translation and validation of the US Parkinson’s Disease Questionnaire (PDQ-39). Quality of Life Research 8:345–50

12. Andreu N (2020) The Parkinson’s Disease Questionnaire (PDQ-39): Construct Validity, Reliability and Patient-Proxy Respondent Agreement of the French Version. Department of Epidemiology and Biostatistics, McGill University, Montreal

13. Schrag A, Selai C, Jahanshahi M, Quinn NP (2000) The EQ-5D—a generic quality of life measure—is a useful instrument to measure quality of life in patients with Parkinson’s disease. J Neurol Neurosurg Psychiatry 69:67–73

14. Katsarou Z, Bostantjopoulou S, Peto V, et al (2001) Quality of life in Parkinson’s disease: Greek translation and validation of the Parkinson’s disease questionnaire (PDQ-39). Quality of Life Research 10:159–63

15. Peto V (2001) Determining minimally important differences for the PDQ-39 Parkinson’s disease questionnaire. Age and Ageing 30:299–302. https://doi.org/10.1093/ageing/30.4.299

16. Tsang K, Chi I, Ho S, et al (2002) Translation and validation of the standard Chinese version of PDQ‐39: A quality‐of‐life measure for patients with Parkinson’s disease. Movement Disorders 17:1036–1040. https://doi.org/10.1002/mds.10249

17. Hagell P, Whalley D, McKenna SP, Lindvall O (2003) Health status measurement in Parkinson’s disease: Validity of the PDQ‐39 and Nottingham Health Profile. Movement Disorders 18:773–783. https://doi.org/10.1002/mds.10438

18. Jenkinson C, Fitzpatrick R, Norquist J, et al (2003) Cross-cultural evaluation of the Parkinson’s Disease Questionnaire: tests of data quality, score reliability, response rate, and scaling assumptions in the United States, Canada, Japan, Italy, and Spain. Journal of Clinical Epidemiology 56:843–847. https://doi.org/10.1016/S0895-4356(03)00148-3

19. Park HJ, Kim S, Song HJ, et al (2004) Korean translation and validation of Parkinson’s Disease Questionnaire (PDQ-39): A pilot test. Quality of Life Research 13:1527

20. Tan LCS, Luo N, Nazri M, et al (2004) Validity and reliability of the PDQ-39 and the PDQ-8 in English-speaking Parkinson’s disease patients in Singapore. Parkinsonism & Related Disorders 10:493–499. https://doi.org/10.1016/j.parkreldis.2004.05.007

21. Fitzpatrick R, Norquist JM, Jenkinson C (2004) Distribution-based criteria for change in health-related quality of life in Parkinson’s disease. Journal of Clinical Epidemiology 57:40–44. https://doi.org/10.1016/j.jclinepi.2003.07.003

22. Haapaniemi TH (2004) The generic 15D instrument is valid and feasible for measuring health related quality of life in Parkinson’s disease. Journal of Neurology, Neurosurgery & Psychiatry 75:976–983. https://doi.org/10.1136/jnnp.2003.015693

23. Martínez-Martín P, Serrano-Dueñas M, Vaca-Baquero V (2005) Psychometric characteristics of the Parkinson’s disease questionnaire (PDQ-39)—Ecuadorian version. Parkinsonism & Related Disorders 11:297–304. https://doi.org/10.1016/j.parkreldis.2005.02.003

24. Ma H-I, Hwang W-J, Chen-Sea M-J (2005) Reliability and validity testing of a Chinese-translated version of the 39-item Parkinson?s Disease Questionnaire (PDQ-39). Qual Life Res 14:565–569. https://doi.org/10.1007/s11136-004-0687-0

25. Luo N, Tan LCS, Li SC, et al (2005) Validity and reliability of the Chinese (Singapore) version of the Parkinson’s Disease Questionnaire (PDQ-39). Qual Life Res 14:273–279. https://doi.org/10.1007/s11136-004-2654-1

26. Martinez-Martin P, Serrano-Dueñas M, Forjaz MJ, Serrano MS (2007) Two questionnaires for Parkinson’s disease: are the PDQ-39 and PDQL equivalent? Qual Life Res 16:1221–1230. https://doi.org/10.1007/s11136-007-9224-2

27. Hagell P, Nygren C (2007) The 39 item Parkinson’s disease questionnaire (PDQ-39) revisited: implications for evidence based medicine. Journal of Neurology, Neurosurgery &amp; Psychiatry 78:1191–1198. https://doi.org/10.1136/jnnp.2006.111161

28. Krikmann Ü, Taba P, Lai T, Asser T (2008) Validation of an Estonian version of the Parkinson’s Disease Questionnaire (PDQ-39). Health Qual Life Outcomes 6:23. https://doi.org/10.1186/1477-7525-6-23

29. Marinus J, Visser M, Jenkinson C, Stiggelbout AM (2008) Evaluation of the Dutch version of the Parkinson’s Disease Questionnaire 39. Parkinsonism & Related Disorders 14:24–27. https://doi.org/10.1016/j.parkreldis.2007.05.005

30. Serrano-Dueñas M, Serrano S (2008) Psychometric characteristics of PIMS—Compared to PDQ-39 and PDQL—To evaluate quality of life in Parkinson’s disease patients: Validation in Spanish (Ecuadorian style). Parkinsonism & Related Disorders 14:126–132. https://doi.org/10.1016/j.parkreldis.2007.07.006

31. Žiropađa L, Stefanova E, Potrebić A, Kostić VS (2009) Quality of life in Serbian patients with Parkinson’s disease. Qual Life Res 18:833–839. https://doi.org/10.1007/s11136-009-9500-4

32. Nojomi M, Mostafavian Z, Shahidi GA, Jenkinson C (2010) Quality of life in patients with Parkinson’s disease: translation and psychometric evaluation of the Iranian version of PDQ-39. J Res Med Sci 15:

33. Luo W, Gui X, Wang B, et al (2010) Validity and reliability testing of the Chinese (mainland) version of the 39-item Parkinson’s Disease Questionnaire (PDQ-39). J Zhejiang Univ Sci B 11:531–538. https://doi.org/10.1631/jzus.B0900380

34. Huang T-T, Hsu H-Y, Wang B-H, Chen K-H (2011) Quality of life in Parkinson’s disease patients: validation of the Short-Form Eight-item Parkinson’s Disease Questionnaire (PDQ-8) in Taiwan. Qual Life Res 20:499–505. https://doi.org/10.1007/s11136-010-9777-3

35. Zhang J-L, Chan P (2012) Reliability and validity of PDQ-39: a quality-of-life measure for patients with PD in China. Qual Life Res 21:1217–1221. https://doi.org/10.1007/s11136-011-0026-1

36. Kwon D-Y, Kim JW, Ma H-I, et al (2013) Translation and Validation of the Korean Version of the 39-Item Parkinson’s Disease Questionnaire. J Clin Neurol 9:26. https://doi.org/10.3988/jcn.2013.9.1.26

37. Park H-J, Sohng K-Y, Kim S (2014) Validation of the Korean version of the 39-Item Parkinson’s Disease Questionnaire (PDQ-39). Asian Nursing Research 8:67–74. https://doi.org/10.1016/j.anr.2014.02.004

38. Fereshtehnejad S-M, Naderi N, Rahmani A, et al (2014) Psychometric study of the Persian short-form eight-item Parkinson’s disease questionnaire (PDQ-8) to evaluate health related quality of life (HRQoL). Health Qual Life Outcomes 12:78. https://doi.org/10.1186/1477-7525-12-78

39. Krygowska-Wajs A, Gorecka-Mazur A, Tomaszewski KA, et al (2015) Psychometric validation of the Polish version Parkinson’s disease questionnaire-39 (PDQ-39) and its short form (PDQ-8). Movement Disorders 30:. https://doi.org/10.1002/mds.26295

40. Morley D, Dummett S, Kelly L, et al (2015) Evaluating the psychometric properties of an e-based version of the 39-item Parkinson’s Disease Questionnaire. Health Qual Life Outcomes 13:5. https://doi.org/10.1186/s12955-014-0193-1

41. Morley D, Jenkinson C, Dummett S, et al (2015) The Parkinson’s disease questionnaire (Pdq-39) - Evaluating The Psychometric Properties of an Electronic Version. Value in Health 18:A286. https://doi.org/10.1016/j.jval.2015.03.1670

42. Jesus-Ribeiro J, Vieira E, Ferreira P, et al (2017) Reliability and Validity of 39-Item Parkinson’s Disease Questionnaire and Parkinson’s Disease Quality of Life Questionnaire. Acta Med Port 30:395–401. https://doi.org/10.20344/amp.8202

43. Galeoto G, Colalelli F, Massai P, et al (2018) Quality of life in Parkinson’s disease: Italian validation of the Parkinson’s Disease Questionnaire (PDQ-39-IT). Neurol Sci 39:1903–1909. https://doi.org/10.1007/s10072-018-3524-x

44. Suratos CTR, Saranza GRM, Sumalapao DEP, Jamora RDG (2018) Quality of life and Parkinson’s disease: Philippine translation and validation of the Parkinson’s disease questionnaire. Journal of Clinical Neuroscience 54:156–160. https://doi.org/10.1016/j.jocn.2018.06.013

45. Holden SK, Koljack CE, Prizer LP, et al (2019) Measuring quality of life in palliative care for Parkinson’s disease: A clinimetric comparison. Parkinsonism & Related Disorders 65:172–177. https://doi.org/10.1016/j.parkreldis.2019.06.018

46. Nelson G, Ndlovu N, Christofides N, et al (2020) Validation of Parkinson’s Disease-Related Questionnaires in South Africa. Parkinson’s Disease 2020:1–9. https://doi.org/10.1155/2020/7542138

47. Kim S, Park H-J, Park MJ, Kim HS (2020) Reliability and Validity of the Korean Short-form Eight-item Parkinson’s Disease Questionnaire (PDQ-8). J Health Info Stat 45:147–156. https://doi.org/10.21032/jhis.2020.45.2.147

48. Hanff A-M, McCrum C, Rauschenberger A, et al (2023) Validation of a Parkinson’s disease questionnaire-39-based functional mobility composite score (FMCS) in people with Parkinson’s disease. Parkinsonism & Related Disorders 112:105442. https://doi.org/10.1016/j.parkreldis.2023.105442

49. Katsarou Z, Bostantjopoulou S, Peto V, et al (2004) Assessing quality of life in Parkinson’s disease: Can a short‐form questionnaire be useful? Movement Disorders 19:308–312. https://doi.org/10.1002/mds.10678

50. Tan LCS, Lau P-N, Au W-L, Luo N (2007) Validation of PDQ-8 as an independent instrument in English and Chinese. Journal of the Neurological Sciences 255:77–80. https://doi.org/10.1016/j.jns.2007.01.072

51. Jenkinson C, Fitzpatrick R (2007) Cross-cultural evaluation of the short form 8-item Parkinson’s Disease Questionnaire (PDQ-8): Results from America, Canada, Japan, Italy and Spain. Parkinsonism & Related Disorders 13:22–28. https://doi.org/10.1016/j.parkreldis.2006.06.006

52. Franchignoni F, Giordano A, Ferriero G (2008) Rasch analysis of the short form 8-item Parkinson’s Disease Questionnaire (PDQ-8). Qual Life Res 17:541–548. https://doi.org/10.1007/s11136-008-9341-6

53. Dal Bello-Haas V, Klassen L, Sheppard MS, Metcalfe A (2011) Psychometric Properties of Activity, Self-Efficacy, and Quality-of-Life Measures in Individuals with Parkinson Disease. Physiotherapy Canada 63:47–57. https://doi.org/10.3138/ptc.2009-08

54. Alvarado-Bolaños A, Cervantes-Arriaga A, Rodríguez-Violante M, et al (2015) Convergent validation of EQ-5D-5L in patients with Parkinson’s disease. Journal of the Neurological Sciences 358:53–57. https://doi.org/10.1016/j.jns.2015.08.010

55. Kahraman T, Genc A, Soke F, et al (2017) Validity and Reliability of the Turkish Version of the 8-Item Parkinson’s Disease Questionnaire. Arch Neuropsychiatr. https://doi.org/10.5152/npa.2017.19343

56. Ramadhan M, Schrag A, and the CLaSP Consortium (2023) The Validity of Health-Related Quality of Life Instruments in Patients With Late-Stage Parkinson’s Disease. J Geriatr Psychiatry Neurol 36:225–232. https://doi.org/10.1177/08919887221119963

57. Stathis P, Papadopoulos G (2022) Evaluation and validation of a patient-reported quality-of-life questionnaire for Parkinson’s disease. J Patient Rep Outcomes 6:17. https://doi.org/10.1186/s41687-022-00427-0

58. Kawaguchi M, Miyagi Y, Kishimoto J, et al (2021) Development of Quality of Life Questionnaire for Patients with Parkinson’s Disease Undergoing STN-DBS. Neurol Med Chir(Tokyo) 61:475–483. https://doi.org/10.2176/nmc.oa.2020-0388

59. De Boer AG, Wijker W, Speelman JD, De Haes JC (1996) Quality of life in patients with Parkinson’s disease: development of a questionnaire. Journal of Neurology, Neurosurgery & Psychiatry 61:70–74. https://doi.org/10.1136/jnnp.61.1.70

60. Serrano-Dueñas M, Martı́nez-Martı́n P, Vaca-Baquero V (2004) Validation and cross-cultural adjustment of PDQL-questionnaire, Spanish version (Ecuador) (PDQL-EV). Parkinsonism & Related Disorders 10:433–437. https://doi.org/10.1016/j.parkreldis.2004.05.002

61. Campos M, Rezende CHAD, Farnese VDC, et al (2011) Translation, Cross-Cultural Adaptation, and Validation of the Parkinson’s Disease Quality of Life Questionnaire (PDQL), the “PDQL-BR”, into Brazilian Portuguese. ISRN Neurology 2011:1–5. https://doi.org/10.5402/2011/954787

62. Dereli EE, Yaliman A, Colaka TK, et al (2015) Turkish Version Study of “Parkinson’s Disease Quality of Life Questionnaire” (PDQL). Arch Neuropsychiatr 52:128–132. https://doi.org/10.5152/npa.2015.7359

63. Welsh M, McDermott MP, Holloway RG, et al (2003) Development and testing of the Parkinson’s disease quality of life scale. Movement Disorders 18:637–645. https://doi.org/10.1002/mds.10424

64. Calne S, Schulzer M, Mak E, et al (1996) Validating a quality of life rating scale for idiopathic parkinsonism: Parkinson’s Impact Scale (PIMS). Parkinsonism & Related Disorders 2:55–61. https://doi.org/10.1016/1353-8020(95)00026-7

65. Schulzer M, Mak E, Calne SM (2003) The psychometric properties of the Parkinson’s Impact Scale (PIMS) as a measure of quality of life in Parkinson’s disease. Parkinsonism & Related Disorders 9:291–294. https://doi.org/10.1016/S1353-8020(03)00019-1

66. Aggarwal R, Goyal V, Pandey R, et al (2020) Development and validation of quality of life in Parkinson’s disease instrument. Ann Indian Acad Neurol 23:59. https://doi.org/10.4103/aian.AIAN_471_18

67. Kuehler A (2003) A novel quality of life instrument for deep brain stimulation in movement disorders. Journal of Neurology, Neurosurgery & Psychiatry 74:1023–1030. https://doi.org/10.1136/jnnp.74.8.1023

68. Krygowska-Wajs A, Tomaszewski KA, Gorecka-Mazur A, et al (2015) Translation into Polish and validation of two quality of life assessment modules for patients with Parkinson’s disease-QLSMMD and QLSM-DBS. Movement Disorders 30:. https://doi.org/10.1002/mds.26295

69. Bose M, Bellare B, Parmar LD (2018) Development of health related quality of life questionnaire for people with parkinson’s disease (PWP) in India. Neurorehabil Neural Repair 32:349–50. https://doi.org/10.1177/1545968318765497

70. Diniz JG, Da Silva AC, Nóbrega AC (2018) Quality of life and swallowing questionnaire for individuals with Parkinson’s disease: development and validation. Intl J Lang & Comm Disor 53:864–874. https://doi.org/10.1111/1460-6984.12395

71. García-Gordillo MÁ, Del Pozo-Cruz B, Adsuar JC, et al (2014) Validation and comparison of 15-D and EQ-5D-5L instruments in a Spanish Parkinson’s disease population sample. Qual Life Res 23:1315–1326. https://doi.org/10.1007/s11136-013-0569-4

72. Del Pozo-Cruz B, Collado-Mateo D, Adsuar JC, et al (2018) A Psychometric Comparison of Different Health Utility Measures in Patients Affected by Parkinson’s Disease. Nursing Economic 36:233–44

73. Luo N, Low S, Lau P-N, et al (2009) Is EQ-5D a Valid Quality of Life Instrument in Patients With Parkinson’s Disease? A Study in Singapore. Ann Acad Med Singap 38:521–528. https://doi.org/10.47102/annals-acadmedsg.V38N6p521

74. Garcia-Gordillo MÁ, Del Pozo-Cruz B, Adsuar JC, et al (2015) Validation and comparison of EQ-5D-3L and SF-6D instruments in a Spanish Parkinson ́s disease population sample. Nutrición Hospitalaria 32:2808–21. https://doi.org/10.3305/nh.2015.32.6.9765

75. Nowinski CJ, Siderowf A, Victorson D, et al (2010) Clinical validation of neuro-QOL measurement tools in Parkinson’s disease. Annals of Neurology 68:. https://doi.org/10.1002/ana.22175

76. Nowinski CJ, Siderowf A, Simuni T, et al (2016) Neuro-QoL health-related quality of life measurement system: Validation in Parkinson’s disease. Movement Disorders 31:725–733. https://doi.org/10.1002/mds.26546

77. Kuspinar A, Mate K, Lafontaine A-L, Mayo N (2019) Evaluating the content validity of generic preference-based measures for use in Parkinson’s disease. Parkinsonism & Related Disorders 62:112–116. https://doi.org/10.1016/j.parkreldis.2019.01.014

78. Kuspinar A, Mate KKV, Lafontaine A-L, Mayo N (2020) Validation of an Individualized Measure of Quality of Life, Patient Generated Index, for Use with People with Parkinson’s Disease. Neurology Research International 2020:1–8. https://doi.org/10.1155/2020/6916135

79. Hagell P, Westergren A (2011) Measurement Properties of the SF-12 Health Survey in Parkinson’s Disease. Journal of Parkinson’s Disease 1:185–196. https://doi.org/10.3233/JPD-2011-11026

80. Steffen T, Seney M (2008) Test-Retest Reliability and Minimal Detectable Change on Balance and Ambulation Tests, the 36-Item Short-Form Health Survey, and the Unified Parkinson Disease Rating Scale in People With Parkinsonism. Physical Therapy 88:733–746. https://doi.org/10.2522/ptj.20070214

81. Hagell P, Törnqvist AL, Hobart J (2008) Testing the SF-36 in Parkinson’s disease: Implications for reporting rating scale data. J Neurol 255:246–254. https://doi.org/10.1007/s00415-008-0708-y

82. Schneider CB, Pilhatsch M, Rifati M, et al (2010) Utility of the WHO‐five well‐being index as a screening tool for depression in Parkinson’s disease. Movement Disorders 25:777–783. https://doi.org/10.1002/mds.22985

83. Hendred SK, Foster ER (2016) Use of the World Health Organization Quality of Life Assessment Short Version in Mild to Moderate Parkinson Disease. Archives of Physical Medicine and Rehabilitation 97:2123-2129.e1. https://doi.org/10.1016/j.apmr.2016.05.020
