# Supplementary material for: Quality of life measures in Parkinson’s disease: a systematic literature review of patient-reported outcomes measures (PROMs) and their psychometric properties
Source: J Neurol. 2025 Aug 28;272(9):598. doi: 10.1007/s00415-025-13348-x (PMC12394374; doi:10.1007/s00415-025-13348-x)
Supplement: Supplementary file 3 — Supplementary file3 (DOCX 62 KB) [file 415_2025_13348_MOESM3_ESM.docx]

**Quality of Life Measures in Parkinson’s Disease: A Systematic Literature Review of Patient-Reported Outcomes Measures (PROMs) and their Psychometric Properties**

**– ONLINE RESOURCE 1 –**

**SUPPLEMENTARY TABLES**

[Table S1. SLR inclusion and exclusion criteria. 2](#_Toc204791237)

[Table S2. Search strategy in PubMed (MEDLINE). 3](#_Toc204791238)

[Table S3. Search strategy in Embase. 5](#_Toc204791239)

[Table S4. Search strategy in Scopus. 7](#_Toc204791240)

[Table S5. Search strategy in WoS. 9](#_Toc204791241)

[Table S6. Search strategy in PSICODOC. 11](#_Toc204791242)

[Table S7. Search strategy for ‘grey literature’. 13](#_Toc204791243)

Table S1. SLR inclusion and exclusion criteria.

| Inclusion criteria | Exclusion criteria |
| --- | --- |
| - Studies presenting the development or validation of PROMs and PREMs. - Transcultural adaptation studies with previously designed PROMs and PREMs. - Studies including participants diagnosed with PD. - Manuscripts written in English and/or Spanish. | - Studies unrelated with development, validation and/or transcultural adaptation of PROMs and PREMs. - Studies including participants without diagnosis of PD. - Manuscripts written in different languages than English and/or Spanish. |

Table S2. Search strategy in PubMed (MEDLINE).

| Search terms | | Search strategy | Number of studies |
| --- | --- | --- | --- |
| **Search date:** 02/04/2024 | | | |
| **#1** | Enfermedad de Parkinson | "parkinson*"[Title/Abstract] OR "parkinson disease"[MeSH Terms] | 160,473 |
| **#2** | *Patient-Reported Outcome Measure* (PROM) | "patient reported outcome* measure*"[Title/Abstract] OR "patient reported outcome* measure*"[Title/Abstract] OR "patient reported outcome*"[Title/Abstract] OR "patient reported outcome*"[Title/Abstract] | 41,336 |
| **#3** | *Patient-Reported Experience Measure* (PREM) | "patient reported experience* measure*"[Title/Abstract] OR "patient reported experience* measure*"[Title/Abstract] OR "patient reported experience*"[Title/Abstract] OR "patient reported experience*"[Title/Abstract] | 901 |
| **#4** | Instrumento | "Instrument"[Title] | 18,372 |
| **#5** | Herramienta | "Tool"[Title] | 83,155 |
| **#6** | Cuestionario | "surveys and questionnaires"[MeSH Terms] OR "questionnaire*"[Title/Abstract] OR “survey*”[Title/Abstract] | 2,127,212 |
| **#7** | Desarrollo | "Develop*"[Title] | 740,375 |
| **#8** | Diseño | "Design"[Title] | 145,896 |
| **#9** | Validación | "Validation"[Title] | 101,475 |
| **#10** | Psicometría | "psychometric*"[Title/Abstract] OR "psychometrics"[MeSH Terms] | 119,721 |
| **#11** | #2 OR #3 OR #4 OR #5 OR #6 | (((("patient reported outcome* measure*"[Title/Abstract] OR "patient reported outcome* measure*"[Title/Abstract] OR "patient reported outcome*"[Title/Abstract] OR "patient reported outcome*"[Title/Abstract]) OR ("patient reported experience* measure*"[Title/Abstract] OR "patient reported experience* measure*"[Title/Abstract] OR "patient reported experience*"[Title/Abstract] OR "patient reported experience*"[Title/Abstract])) OR ("Instrument"[Title])) OR ("Tool"[Title])) OR ("surveys and questionnaires"[MeSH Terms] OR "questionnaire*"[Title/Abstract] OR "survey*"[Title/Abstract]) | 2,226,436 |
| **#12** | #7 OR #8 OR #9 OR #10 | ((("Develop*"[Title]) OR ("Design"[Title])) OR ("Validation"[Title])) OR ("psychometric*"[Title/Abstract] OR "psychometrics"[MeSH Terms]) | 1,053,649 |
| **#13** | #11 AND #12 | ((((("patient reported outcome* measure*"[Title/Abstract] OR "patient reported outcome* measure*"[Title/Abstract] OR "patient reported outcome*"[Title/Abstract] OR "patient reported outcome*"[Title/Abstract]) OR ("patient reported experience* measure*"[Title/Abstract] OR "patient reported experience* measure*"[Title/Abstract] OR "patient reported experience*"[Title/Abstract] OR "patient reported experience*"[Title/Abstract])) OR ("Instrument"[Title])) OR ("Tool"[Title])) OR ("surveys and questionnaires"[MeSH Terms] OR "questionnaire*"[Title/Abstract] OR "survey*"[Title/Abstract])) AND (((("Develop*"[Title]) OR ("Design"[Title])) OR ("Validation"[Title])) OR ("psychometric*"[Title/Abstract] OR "psychometrics"[MeSH Terms])) | 967 |
| **#14** | #1 AND #13 | ("parkinson*"[Title/Abstract] OR "parkinson disease"[MeSH Terms]) AND (((((("patient reported outcome* measure*"[Title/Abstract] OR "patient reported outcome* measure*"[Title/Abstract] OR "patient reported outcome*"[Title/Abstract] OR "patient reported outcome*"[Title/Abstract]) OR ("patient reported experience* measure*"[Title/Abstract] OR "patient reported experience* measure*"[Title/Abstract] OR "patient reported experience*"[Title/Abstract] OR "patient reported experience*"[Title/Abstract])) OR ("Instrument"[Title])) OR ("Tool"[Title])) OR ("surveys and questionnaires"[MeSH Terms] OR "questionnaire*"[Title/Abstract] OR "survey*"[Title/Abstract])) AND (((("Develop*"[Title]) OR ("Design"[Title])) OR ("Validation"[Title])) OR ("psychometric*"[Title/Abstract] OR "psychometrics"[MeSH Terms]))) | 761 |
| **#15** | Filtro de idioma: Inglés / Castellano | "English"[Language] OR "Spanish"[Language] | 32,541,379 |
| **#16** | #14 AND #15 | (("parkinson*"[Title/Abstract] OR "parkinson disease"[MeSH Terms]) AND (((((("patient reported outcome* measure*"[Title/Abstract] OR "patient reported outcome* measure*"[Title/Abstract] OR "patient reported outcome*"[Title/Abstract] OR "patient reported outcome*"[Title/Abstract]) OR ("patient reported experience* measure*"[Title/Abstract] OR "patient reported experience* measure*"[Title/Abstract] OR "patient reported experience*"[Title/Abstract] OR "patient reported experience*"[Title/Abstract])) OR ("Instrument"[Title])) OR ("Tool"[Title])) OR ("surveys and questionnaires"[MeSH Terms] OR "questionnaire*"[Title/Abstract] OR "survey*"[Title/Abstract])) AND (((("Develop*"[Title]) OR ("Design"[Title])) OR ("Validation"[Title])) OR ("psychometric*"[Title/Abstract] OR "psychometrics"[MeSH Terms])))) AND ("English"[Language] OR "Spanish"[Language]) | 745 |

Table S3. Search strategy in Embase.

| Search terms | | Search strategy | Number of studies |
| --- | --- | --- | --- |
| **Search date:** 02/04/2024 | | | |
| **#1** | Enfermedad de Parkinson | parkinson*:ti,ab,kw OR 'parkinson disease'/exp | 260,857 |
| **#2** | *Patient-Reported Outcome Measure* (PROM) | ('patient-reported outcome* measure*':ti,ab,kw OR 'patient reported outcome* measure*':ti,ab,kw OR 'patient-reported outcome*':ti,ab,kw) OR 'patient reported outcome*':ti,ab,kw OR 'patient-reported outcome'/exp | 83,974 |
| **#3** | *Patient-Reported Experience Measure* (PREM) | 'patient-reported experience* measure*':ti,ab,kw OR 'patient reported experience* measure*':ti,ab,kw OR 'patient-reported experience*':ti,ab,kw OR 'patient reported experience*':ti,ab,kw OR 'patient reported experience measure'/exp | 1,368 |
| **#4** | Instrumento | instrument:ti | 21,439 |
| **#5** | Herramienta | tool:ti | 110,494 |
| **#6** | Cuestionario | questionnaire*:ti,ab,kw OR survey*:ti,ab,kw OR 'questionnaire'/exp | 2,122,268 |
| **#7** | Desarrollo | develop*:ti | 906,249 |
| **#8** | Diseño | design:ti | 165,621 |
| **#9** | Validación | validation:ti | 138,735 |
| **#10** | Psicometría | psychometric*: ti,ab,kw OR 'psychometry'/exp | 117,749 |
| **#11** | #2 OR #3 OR #4 OR #5 OR #6 | (('patient-reported outcome* measure*':ti,ab,kw OR 'patient reported outcome* measure*':ti,ab,kw OR 'patient-reported outcome*':ti,ab,kw) OR 'patient reported outcome*':ti,ab,kw OR 'patient-reported outcome'/exp) OR ('patient-reported experience* measure*':ti,ab,kw OR 'patient reported experience* measure*':ti,ab,kw OR 'patient-reported experience*':ti,ab,kw OR 'patient reported experience*':ti,ab,kw OR 'patient reported experience measure'/exp) OR (instrument:ti) OR (tool:ti) OR (questionnaire*:ti,ab,kw OR survey*:ti,ab,kw OR 'questionnaire'/exp) | 2,283,663 |
| **#12** | #7 OR #8 OR #9 OR #10 | (develop*:ti) OR (design:ti) OR (validation:ti) OR (psychometric*: ti,ab,kw OR 'psychometry'/exp) | 1,269,136 |
| **#13** | #11 AND #12 | ((('patient-reported outcome* measure*':ti,ab,kw OR 'patient reported outcome* measure*':ti,ab,kw OR 'patient-reported outcome*':ti,ab,kw) OR 'patient reported outcome*':ti,ab,kw OR 'patient-reported outcome'/exp) OR ('patient-reported experience* measure*':ti,ab,kw OR 'patient reported experience* measure*':ti,ab,kw OR 'patient-reported experience*':ti,ab,kw OR 'patient reported experience*':ti,ab,kw OR 'patient reported experience measure'/exp) OR (instrument:ti) OR (tool:ti) OR (questionnaire*:ti,ab,kw OR survey*:ti,ab,kw OR 'questionnaire'/exp)) AND ((develop*:ti) OR (design:ti) OR (validation:ti) OR (psychometric*: ti,ab,kw OR 'psychometry'/exp)) | 132,906 |
| **#14** | #1 AND #13 | (parkinson*:ti,ab,kw OR 'parkinson disease'/exp) AND (((('patient-reported outcome* measure*':ti,ab,kw OR 'patient reported outcome* measure*':ti,ab,kw OR 'patient-reported outcome*':ti,ab,kw) OR 'patient reported outcome*':ti,ab,kw OR 'patient-reported outcome'/exp) OR ('patient-reported experience* measure*':ti,ab,kw OR 'patient reported experience* measure*':ti,ab,kw OR 'patient-reported experience*':ti,ab,kw OR 'patient reported experience*':ti,ab,kw OR 'patient reported experience measure'/exp) OR (instrument:ti) OR (tool:ti) OR (questionnaire*:ti,ab,kw OR survey*:ti,ab,kw OR 'questionnaire'/exp)) AND ((develop*:ti) OR (design:ti) OR (validation:ti) OR (psychometric*: ti,ab,kw OR 'psychometry'/exp))) | 915 |
| **#15** | Filtro de idioma: Inglés / Castellano | english:la OR spanish:la | 38,810,588 |
| **#16** | #14 AND #15 | ((parkinson*:ti,ab,kw OR 'parkinson disease'/exp) AND (((('patient-reported outcome* measure*':ti,ab,kw OR 'patient reported outcome* measure*':ti,ab,kw OR 'patient-reported outcome*':ti,ab,kw) OR 'patient reported outcome*':ti,ab,kw OR 'patient-reported outcome'/exp) OR ('patient-reported experience* measure*':ti,ab,kw OR 'patient reported experience* measure*':ti,ab,kw OR 'patient-reported experience*':ti,ab,kw OR 'patient reported experience*':ti,ab,kw OR 'patient reported experience measure'/exp) OR (instrument:ti) OR (tool:ti) OR (questionnaire*:ti,ab,kw OR survey*:ti,ab,kw OR 'questionnaire'/exp)) AND ((develop*:ti) OR (design:ti) OR (validation:ti) OR (psychometric*: ti,ab,kw OR 'psychometry'/exp)))) AND (english:la OR spanish:la) | 900 |

Table S4. Search strategy in Scopus.

| Search terms | | Search strategy | Number of studies |
| --- | --- | --- | --- |
| **Search date:** 02/04/2024 | | | |
| **#1** | Enfermedad de Parkinson | TITLE-ABS-KEY ( parkinson* ) | 232,431 |
| **#2** | *Patient-Reported Outcome Measure* (PROM) | ( TITLE-ABS-KEY ( patient-reported AND outcome* AND measure* ) OR TITLE-ABS-KEY ( patient AND reported AND outcome* AND measure* ) OR TITLE-ABS-KEY ( patient-reported AND outcome* ) OR TITLE-ABS-KEY ( patient AND reported AND outcome* ) ) | 389,344 |
| **#3** | *Patient-Reported Experience Measure* (PREM) | ( TITLE-ABS-KEY ( patient-reported AND experience* AND measure* ) OR TITLE-ABS-KEY ( patient AND reported AND experience* AND measure* ) OR TITLE-ABS-KEY ( patient-reported AND experience* ) OR TITLE-ABS-KEY ( patient AND reported AND experience* ) ) | 146,536 |
| **#4** | Instrumento | TITLE ( instrument ) | 96,459 |
| **#5** | Herramienta | TITLE ( tool ) | 380,676 |
| **#6** | Cuestionario | ( TITLE-ABS-KEY ( questionnaire* ) OR TITLE-ABS-KEY ( survey* ) ) | 4,183,000 |
| **#7** | Desarrollo | TITLE ( develop* ) | 2,028,451 |
| **#8** | Diseño | TITLE ( design ) | 1,364,821 |
| **#9** | Validación | TITLE ( validation ) | 211,615 |
| **#10** | Psicometría | TITLE-ABS-KEY ( psychometric* ) OR TITLE-ABS-KEY ( psychometry ) | 166,199 |
| **#11** | #2 OR #3 OR #4 OR #5 OR #6 | ( ( TITLE-ABS-KEY ( patient-reported AND outcome* AND measure* ) OR TITLE-ABS-KEY ( patient AND reported AND outcome* AND measure* ) OR TITLE-ABS-KEY ( patient-reported AND outcome* ) OR TITLE-ABS-KEY ( patient AND reported AND outcome* ) ) ) OR ( ( TITLE-ABS-KEY ( patient-reported AND experience* AND measure* ) OR TITLE-ABS-KEY ( patient AND reported AND experience* AND measure* ) OR TITLE-ABS-KEY ( patient-reported AND experience* ) OR TITLE-ABS-KEY ( patient AND reported AND experience* ) ) ) OR ( TITLE ( instrument ) ) OR ( TITLE ( tool ) ) OR ( ( TITLE-ABS-KEY ( questionnaire* ) OR TITLE-ABS-KEY ( survey* ) ) ) | 4,976,239 |
| **#12** | #7 OR #8 OR #9 OR #10 | ( TITLE ( develop* ) ) OR ( TITLE ( design ) ) OR ( TITLE ( validation ) ) OR ( TITLE-ABS-KEY ( psychometric* ) OR TITLE-ABS-KEY ( psychometry ) ) | 3,644,665 |
| **#13** | #11 AND #12 | ( ( ( TITLE-ABS-KEY ( patient-reported AND outcome* AND measure* ) OR TITLE-ABS-KEY ( patient AND reported AND outcome* AND measure* ) OR TITLE-ABS-KEY ( patient-reported AND outcome* ) OR TITLE-ABS-KEY ( patient AND reported AND outcome* ) ) ) OR ( ( TITLE-ABS-KEY ( patient-reported AND experience* AND measure* ) OR TITLE-ABS-KEY ( patient AND reported AND experience* AND measure* ) OR TITLE-ABS-KEY ( patient-reported AND experience* ) OR TITLE-ABS-KEY ( patient AND reported AND experience* ) ) ) OR ( TITLE ( instrument ) ) OR ( TITLE ( tool ) ) OR ( ( TITLE-ABS-KEY ( questionnaire* ) OR TITLE-ABS-KEY ( survey* ) ) ) ) AND ( ( TITLE ( develop* ) ) OR ( TITLE ( design ) ) OR ( TITLE ( validation ) ) OR ( TITLE-ABS-KEY ( psychometric* ) OR TITLE-ABS-KEY ( psychometry ) ) ) | 308,965 |
| **#14** | #1 AND #13 | ( TITLE-ABS-KEY ( parkinson* ) ) AND ( ( ( ( TITLE-ABS-KEY ( patient-reported AND outcome* AND measure* ) OR TITLE-ABS-KEY ( patient AND reported AND outcome* AND measure* ) OR TITLE-ABS-KEY ( patient-reported AND outcome* ) OR TITLE-ABS-KEY ( patient AND reported AND outcome* ) ) ) OR ( ( TITLE-ABS-KEY ( patient-reported AND experience* AND measure* ) OR TITLE-ABS-KEY ( patient AND reported AND experience* AND measure* ) OR TITLE-ABS-KEY ( patient-reported AND experience* ) OR TITLE-ABS-KEY ( patient AND reported AND experience* ) ) ) OR ( TITLE ( instrument ) ) OR ( TITLE ( tool ) ) OR ( ( TITLE-ABS-KEY ( questionnaire* ) OR TITLE-ABS-KEY ( survey* ) ) ) ) AND ( ( TITLE ( develop* ) ) OR ( TITLE ( design ) ) OR ( TITLE ( validation ) ) OR ( TITLE-ABS-KEY ( psychometric* ) OR TITLE-ABS-KEY ( psychometry ) ) ) ) | 1,009 |
| **#15** | Filtro de idioma: Inglés / Castellano | ( LANGUAGE ( english ) OR LANGUAGE ( spanish ) ) | 84,288,609 |
| **#16** | #14 AND #15 | ( ( TITLE-ABS-KEY ( parkinson* ) ) AND ( ( ( ( TITLE-ABS-KEY ( patient-reported AND outcome* AND measure* ) OR TITLE-ABS-KEY ( patient AND reported AND outcome* AND measure* ) OR TITLE-ABS-KEY ( patient-reported AND outcome* ) OR TITLE-ABS-KEY ( patient AND reported AND outcome* ) ) ) OR ( ( TITLE-ABS-KEY ( patient-reported AND experience* AND measure* ) OR TITLE-ABS-KEY ( patient AND reported AND experience* AND measure* ) OR TITLE-ABS-KEY ( patient-reported AND experience* ) OR TITLE-ABS-KEY ( patient AND reported AND experience* ) ) ) OR ( TITLE ( instrument ) ) OR ( TITLE ( tool ) ) OR ( ( TITLE-ABS-KEY ( questionnaire* ) OR TITLE-ABS-KEY ( survey* ) ) ) ) AND ( ( TITLE ( develop* ) ) OR ( TITLE ( design ) ) OR ( TITLE ( validation ) ) OR ( TITLE-ABS-KEY ( psychometric* ) OR TITLE-ABS-KEY ( psychometry ) ) ) ) ) AND ( ( LANGUAGE ( english ) OR LANGUAGE ( spanish ) ) ) | 966 |

Table S5. Search strategy in WoS.

| Search terms | | Search strategy | Number of studies |
| --- | --- | --- | --- |
| **Search date:** 02/04/2024 | | | |
| **#1** | Enfermedad de Parkinson | TS=(parkinson*) | 341,163 |
| **#2** | *Patient-Reported Outcome Measure* (PROM) | TS=(patient-reported outcome* measure*) OR TS=(patient reported outcome* measure*) OR TS=(patient-reported outcome*) OR TS=(patient reported outcome*) | 745,291 |
| **#3** | *Patient-Reported Experience Measure* (PREM) | TS=(patient-reported experience* measure*) OR TS=(patient reported experience* measure*) OR TS=(patient-reported experience*) OR TS=(patient reported experience*) | 348,524 |
| **#4** | Instrumento | TI=(instrument) | 197,479 |
| **#5** | Herramienta | TI=(tool) | 623,619 |
| **#6** | Cuestionario | TS=(questionnaire*) OR TS=(survey*) | 4,023,801 |
| **#7** | Desarrollo | TI=(develop*) | 2,675,763 |
| **#8** | Diseño | TI=(design*) | 1,433,065 |
| **#9** | Validación | TI=(validation) | 243,899 |
| **#10** | Psicometría | TS=(psychometric*) OR TS=(psychometry) | 205,799 |
| **#11** | #2 OR #3 OR #4 OR #5 OR #6 | (TS=(patient-reported outcome* measure*) OR TS=(patient reported outcome* measure*) OR TS=(patient-reported outcome*) OR TS=(patient reported outcome*)) OR (TS=(patient-reported experience* measure*) OR TS=(patient reported experience* measure*) OR TS=(patient-reported experience*) OR TS=(patient reported experience*)) OR (TI=(instrument)) OR (TI=(tool)) OR (TS=(questionnaire*) OR TS=(survey*)) | 5,576,802 |
| **#12** | #7 OR #8 OR #9 OR #10 | (TI=(develop*)) OR (TI=(design*)) OR (TI=(validation)) OR (TS=(psychometric*) OR TS=(psychometry)) | 4,415,938 |
| **#13** | #11 AND #12 | ((TS=(patient-reported outcome* measure*) OR TS=(patient reported outcome* measure*) OR TS=(patient-reported outcome*) OR TS=(patient reported outcome*)) OR (TS=(patient-reported experience* measure*) OR TS=(patient reported experience* measure*) OR TS=(patient-reported experience*) OR TS=(patient reported experience*)) OR (TI=(instrument)) OR (TI=(tool)) OR (TS=(questionnaire*) OR TS=(survey*))) AND ((TI=(develop*)) OR (TI=(design*)) OR (TI=(validation)) OR (TS=(psychometric*) OR TS=(psychometry))) | 373,303 |
| **#14** | #1 AND #13 | (TS=(parkinson*)) AND (((TS=(patient-reported outcome* measure*) OR TS=(patient reported outcome* measure*) OR TS=(patient-reported outcome*) OR TS=(patient reported outcome*)) OR (TS=(patient-reported experience* measure*) OR TS=(patient reported experience* measure*) OR TS=(patient-reported experience*) OR TS=(patient reported experience*)) OR (TI=(instrument)) OR (TI=(tool)) OR (TS=(questionnaire*) OR TS=(survey*))) AND ((TI=(develop*)) OR (TI=(design*)) OR (TI=(validation)) OR (TS=(psychometric*) OR TS=(psychometry)))) | 1,403 |
| **#15** | Filtro de idioma: Inglés / Castellano | ***NOTA:*** *Filtros añadidos de forma manual* | - |
| **#16** | #14 AND #15  ***NOTE:*** *Filters added manually.* | ((TS=(parkinson*)) AND (((TS=(patient-reported outcome* measure*) OR TS=(patient reported outcome* measure*) OR TS=(patient-reported outcome*) OR TS=(patient reported outcome*)) OR (TS=(patient-reported experience* measure*) OR TS=(patient reported experience* measure*) OR TS=(patient-reported experience*) OR TS=(patient reported experience*)) OR (TI=(instrument)) OR (TI=(tool)) OR (TS=(questionnaire*) OR TS=(survey*))) AND ((TI=(develop*)) OR (TI=(design*)) OR (TI=(validation)) OR (TS=(psychometric*) OR TS=(psychometry))))) AND (#Filters) | 1,377 |

WoS: Web of Science.

Table S6. Search strategy in PSICODOC.

| Search terms | | Search strategy | Number of studies |
| --- | --- | --- | --- |
| **Search date:** 02/04/2024 | | | |
| **#1** | Enfermedad de Parkinson | TX parkinson | 273 |
| **#2** | *Patient-Reported Outcome Measure* (PROM) | TX patient reported outcome measures OR TX patient reported outcome OR TX patient-reported outcome measures OR TX patient-reported outcome | 6 |
| **#3** | *Patient-Reported Experience Measure* (PREM) | TX patient reported experience measures OR TX patient reported experience OR TX patient-reported experience measures OR TX patient-reported experience | 3 |
| **#4** | Instrumento | TX Instrument OR TX Instrumento | 13,526 |
| **#5** | Herramienta | TX Tool OR TX Herramienta | 6,007 |
| **#6** | Cuestionario | TX Questionnaire* OR TX Survey* OR TX Cuestionario* | 17,321 |
| **#7** | Desarrollo | TX Develop* OR TX Desarrollo | 46,230 |
| **#8** | Diseño | TX Design OR TX Diseño | 9,784 |
| **#9** | Validación | TX Validation OR TX Validación | 3,990 |
| **#10** | Psicometría | TX Psychometric* OR TX Psychometry OR TX Psicométric* OR TX Psicometría | 6,904 |
| **#11** | #2 OR #3 OR #4 OR #5 OR #6 | (TX patient reported outcome measures OR TX patient reported outcome OR TX patient-reported outcome measures OR TX patient-reported outcome ) OR (TX patient reported experience measures OR TX patient reported experience OR TX patient-reported experience measures OR TX patient-reported experience) OR (TX Instrument OR TX Instrumento) OR (TX Tool OR TX Herramienta) OR (TX Questionnaire* OR TX Survey* OR TX Cuestionario*) | 29,883 |
| **#12** | #7 OR #8 OR #9 OR #10 | (TX Develop* OR TX Desarrollo) OR (TX Design OR TX Diseño) OR (TX Validation OR TX Validación) OR (TX Psychometric* OR TX Psychometry OR TX Psicométric* OR TX Psicometría) | 56,718 |
| **#13** | #11 AND #12 | ((TX patient reported outcome measures OR TX patient reported outcome OR TX patient-reported outcome measures OR TX patient-reported outcome ) OR (TX patient reported experience measures OR TX patient reported experience OR TX patient-reported experience measures OR TX patient-reported experience) OR (TX Instrument OR TX Instrumento) OR (TX Tool OR TX Herramienta) OR (TX Questionnaire* OR TX Survey* OR TX Cuestionario*)) AND ((TX Develop* OR TX Desarrollo) OR (TX Design OR TX Diseño) OR (TX Validation OR TX Validación) OR (TX Psychometric* OR TX Psychometry OR TX Psicométric* OR TX Psicometría)) | 16,023 |
| **#14** | #1 AND #13 | (TX parkinson ) AND (((TX patient reported outcome measures OR TX patient reported outcome OR TX patient-reported outcome measures OR TX patient-reported outcome ) OR (TX patient reported experience measures OR TX patient reported experience OR TX patient-reported experience measures OR TX patient-reported experience) OR (TX Instrument OR TX Instrumento) OR (TX Tool OR TX Herramienta) OR (TX Questionnaire* OR TX Survey* OR TX Cuestionario*)) AND ((TX Develop* OR TX Desarrollo) OR (TX Design OR TX Diseño) OR (TX Validation OR TX Validación) OR (TX Psychometric* OR TX Psychometry OR TX Psicométric* OR TX Psicometría))) | 17 |

Table S7. Search strategy for ‘grey literature’.

| Source | Link to web |
| --- | --- |
| AAN | <https://www.aan.com/> |
| AMN | <https://www.neurologia.org.mx/> |
| ANS | <https://www.ans.org.au/> |
| BiblioPRO | <https://bibliopro.org/es/> |
| CNS | <https://www.cnsf.org/cns/about-cns/> |
| Dialnet | <https://dialnet.unirioja.es/> |
| EAN | <https://www.ean.org/> |
| Google | [www.google.com](http://www.google.com) (manual review of first 200 registries searching for “Parkinson” and “Questionnaire” together) |
| IEXP | <https://iexp.es/> |
| IPMDS | <https://www.apdaparkinson.org/> |
| ISPOR | <https://www.ispor.org/> |
| SEN | <https://www.sen.es/> |
| SNA | <https://www.sna.org.ar/index.php> |
| SNU | <https://sociedadneurociencias.uy/> |
| SONEPSYN | <https://www.sonepsyn.cl/web/> |
| WFN | <https://wfneurology.org/> |
