# Supplementary material for: Quality of life measures in Parkinson’s disease: a systematic literature review of patient-reported outcomes measures (PROMs) and their psychometric properties
Source: J Neurol. 2025 Aug 28;272(9):598. doi: 10.1007/s00415-025-13348-x (PMC12394374; doi:10.1007/s00415-025-13348-x)
Supplement: Supplementary file 4 — Supplementary file4 (DOCX 46 KB) [file 415_2025_13348_MOESM4_ESM.docx]

**Quality of Life Measures in Parkinson’s Disease: A Systematic Literature Review of Patient-Reported Outcomes Measures (PROMs) and their Psychometric Properties**

**– ONLINE RESOURCE 2 –**

Table S8. COSMIN criteria for good measurement property.

| Measurement property | Rating | Criteria |
| --- | --- | --- |
| **Structural validity** | (+) | **CTT:**  CFA: CFI or TLI or comparable measure >0.95 OR RMSEA <0.06 OR SRMR <0.082.  **IRT / Rasch**:  No violation of unidimensionality3: CFI or TLI or comparable measure >0.95 OR RMSEA <0.06 OR SRMR <0.08.  *AND*  no violation of local independence: residual correlations among the items after controlling for the dominant factor < 0.20 OR Q3's < 0.37.  *AND*  no violation of monotonicity: adequate looking graphs OR item scalability >0.30.  *AND*  adequate model fit:  IRT: χ2 >0.01.  Rasch: infit and outfit mean squares ≥ 0.5 and ≤ 1.5 OR Z-standardized values > ‐2 and <2. |
|  | (?) | CTT: Not all information for ‘+’ reported.  IRT/Rasch: Model fit not reported. |
|  | (–) | Criteria for ‘+’ not met. |
| **Internal consistency** | (+) | At least low evidence for sufficient structural validity AND Cronbach's alpha(s) ≥ 0.70 for each unidimensional scale or subscale. |
|  | (?) | Criteria for “At least low evidence4 for sufficient structural validity” not met. |
|  | (–) | At least low evidence for sufficient structural validity AND Cronbach’s alpha(s) < 0.70 for each unidimensional scale or subscale. |
| **Temporal stability** | (+) | ICC or weighted Kappa ≥ 0.70. |
|  | (?) | ICC or weighted Kappa not reported. |
|  | (–) | ICC or weighted Kappa < 0.70. |
| **ME** | (+) | SDC or LoA < MIC. |
|  | (?) | MIC not defined. |
|  | (–) | SDC or LoA > MIC. |
| **Hypothesis testing for construct validity** | (+) | The result is in accordance with the hypothesis. |
|  | (?) | No hypothesis defined (by the review team). |
|  | (–) | The result is not in accordance with the hypothesis. |
| **Criterion validity** | (+) | Correlation with gold standard ≥ 0.70 OR AUC ≥ 0.70. |
|  | (?) | Not all information for ‘+’ reported. |
|  | (–) | Correlation with gold standard < 0.70 OR AUC < 0.70. |

Source (adapted): L.B. Mokkink, H.C.W. De Vet, C.A.C. Prinsen, D.L. Patrick, J. Alonso, L.M. Bouter, C.B. Terwee, COSMIN Risk of Bias checklist for systematic reviews of Patient-Reported Outcome Measures, Qual Life Res 27 (2018) 1171–1179. https://doi.org/10.1007/s11136-017-1765-4.
