# Supplementary material for: Quality of life measures in Parkinson’s disease: a systematic literature review of patient-reported outcomes measures (PROMs) and their psychometric properties
Source: J Neurol. 2025 Aug 28;272(9):598. doi: 10.1007/s00415-025-13348-x (PMC12394374; doi:10.1007/s00415-025-13348-x)
Supplement: Supplementary file 5 — Supplementary file5 (DOCX 183 KB) [file 415_2025_13348_MOESM5_ESM.docx]

Quality of Life Measures in Parkinson’s Disease: A Systematic Literature Review of Patient-Reported Outcomes Measures (PROMs) and their Psychometric Properties

**– ONLINE RESOURCE 3 –**

Table S9. Description of the eligible studies, participants characteristics and PROMs’ scores.

| Eligible study | Country | Sample size | Participants’ characteristics | PROMs’ scores |
| --- | --- | --- | --- | --- |
| Spliethoff-Kamminga (2003) [1] | Netherlands (Leiden) | 54 | Age [mean (SD)] = 71.6 y (8.3) / Range = 48-87  Women [%] = 37.0%  Level of studies [%]: Less than high school = 37% / High school = 46% / More than high school = 17%  Delay in PD diagnosis (years) [mean (SD)] = 1.6 (2.3) / Range = 0-12  Member of DPDA (Dutch Parkinson’s Disease Association) [%, Yes] = 50%  H&YS [mean (SD)] = 2.4 (0.9)  Duration of disease (years) [mean (SD)] = 6.8 (4.3) / Range = 1-15 | Bela-P-K (Bb) [mean (SD)] = 24.0 (12.8) / Bela-P-K (Bb) [median (Range)] = 25.0 (0-58)  Bela-P-K (Nfh) [mean (SD)] = 24.8 (17.2) / Bela-P-K (Nfh) [median (Range)] = 27.5 (0-57)  Bb:   - Achievement capability / Physical symptoms: Mean (SD) = 8.8 (4.7) / Median = 9.0 / Range = 0-18 - Fear / Emotional symptoms: Mean (SD) = 6.3 (3.8) / Median = 6.0 / Range = 0-13 - Social functioning: Mean (SD) = 5.0 (3.7) / Median = 4.0 / Range = 0-13 - Partner-bonding / Family: Mean (SD) = 3.6 (3.2) / Median = 4.0 / Range = 0-16   Nfh:   - Achievement capability / Physical symptoms: Mean (SD) = 9.6 (6.3) / Median = 10.0 / Range = 0-20 - Fear / Emotional symptoms: Mean (SD) = 6.9 (4.9) / Median = 8.0 / Range = 0-16 - Social functioning: Mean (SD) = 5.2 (4.6) / Median = 4.5 / Range = 0-17 - Partner-bonding: Mean (SD) = 3.4 (4.2) / Median = 2.0 / Range = 0-15 |
| Ortelli (2017) [2] | Italy (Como) | 202 | Age [mean (SD)] = 65.8 (9.0)  Women [%] = 48.0%  Years of education [mean (SD)] = 11.3 (4.2)  MMSE [mean (SD)] = 28.3 (1.6)  FAB [mean (SD)] = 14.5 (2.6)  UPDRS-Total [mean (SD)] = 39.7 (13.1)  UPDRS-Part II [mean (SD)] = 14.2 (5.7)  UPDRS-Part III [mean (SD)] = 19.3 (6.2)  UPDRS-Part IV [mean (SD)] = 4.2 (3.7)  H&YS [mean (SD)] = 2.7 (0.6) / H&YS [%]: Stage 1 = 37% / Stage 2 = 57% / Stage 3 = 6% | Bela-P-K response rate = 98.02%  Bela-P-K (Bb) [mean (SD)] = 22.1 (16.5) / Bela-P-K (Bb) [median (IQR; P25-P75)] = 19.0 (21.0; 10-31)  Bela-P-K (Nfh) [mean (SD)] = 19.7 (16.0) / Bela-P-K (Nfh) [median (IQR; P25-P75)] = 15.0 (21.0; 7-28=  Bb:   - Achievement capability / Physical symptoms: Mean (SD) = 6.6 (4.8) / Median (P25-P75) = 6.0 (3.0-9.25) - Fear / Emotional symptoms: Mean (SD) = 6.0 (4.4) / Median (P25-P75) = 5.0 (2.0-9.0) - Social functioning: Mean (SD) = 5.0 (5.2) / Median (P25-P75) = 4.0 (1.0-8.0) - Partner-bonding / Family: Mean (SD) = 4.5 (4.8) / Median (P25-P75) = 3.0 (1.0-7.0)   Nfh:   - Achievement capability / Physical symptoms: Mean (SD) = 6.0 (4.9) / Median (P25-P75) = 5.0 (2.0-9.0) - Fear / Emotional symptoms: Mean (SD) = 5.1 (4.3) / Median (P25-P75) = 4.0 (2.0-8.0) - Social functioning: Mean (SD) = 4.6 (5.0) / Median (P25-P75) = 3.0 (0.5-7.0) - Partner-bonding / Family: Mean (SD) = 4.1 (4.7) / Median (P25-P75) = 2.0 (0.0-6.0) |
| Bayen (2021) [3] | France | 18 | Women [%] = 22.2% | – |
| Aggarwal (2013) [4] | India | 277 | – | – |
| Kuharic (2022) [5] | United States | 569 | – | – |
| Kuharic (2024) [6] | United States | 569 | Age [mean (SD)] = 64.7 y (9.0) / Age [%]: <55 y = 13.0% / 55-60 = 14.2% / 61-65 = 19.2% / 66-70 y = 23.9% / >70 y = 29.7%  Women [%] = 54.0%  Race [%]: White or Caucasian = 96.8% / Black or African American = 0.4% / American Indian or Alaska Native = 1.2% / Asian = 2.8% / Native Hawaiian or Other Pacific Islander = 0.7% / Prefer not to answer = 0.5%  Ethnicity [%]: Not Hispanic, Latino/a or Spanish origin = 93.0% / Mexican, Mexican American, Chicano = 1.2% / Puerto Rican = 0.5% / Cuban = 0.4% / Another Hispanic, Latino/a or Spanish Origin = 3.2% / Prefer not to answer = 2.5%  Education [%]: Less than a high school degree = 1.2% / High school degree (GED or equivalent) = 6.7% / Above high school degree = 92.1% / Prefer not to answer = 0.4%  Employment [%]: Employed, full-time = 17.2% / Employed, part-time = 7.0% / Retired = 68.7% / Unemployed = 6.3% / Prefer not to answer = 0.7%  Patient location [%]: United States = 82.4% / Outside United States = 17.6%  Five Most Common Comorbid Conditions [%]: Arthritis (osteoarthritis, rheumatoid arthritis) = 43.2% / Anxiety = 31.4% / Depression = 25.9% / Gastric Disturbances = 21.8% / Heart disease = 10.3%  Years having PD [%]: <3 = 34.7% / 3-10 = 47.3% / 11-50 = 17.8%  EQ-5D-5L Utility [mean (SD)] = 0.66 (0.2)  EQ-VAS [mean (SD)] = 68.25 (19.9)  PDQ-8 SI [mean (SD)] = 22.5 (15.3)  GDS [mean (SD)] = 3.82 (3.4)  UPDRS-Part 2 [mean (SD)] = 25.89 (7.2)  PDAQ-15 [mean (SD)] = 50.4 (9.4)  NMSQ [mean (SD)] = 11.17 (5.0)  Diagnosis provider [%]: Primary Care Physician = 7.34% / General Neurologist = 55% / Neurologist Specialing in Movement Disorders = 46.6% / Other = 1.8%  Most Common PD-indicated Medication Use [%]: Carbidopa/Levodopa Immediate Release (Sinemet) = 84.4% / Carbidopa/Levodopa Controlled Release (Sinemet CR.) = 47.2% / Rasagiline (Azilect) = 31.4% / Carbidopa/Levodopa Extended Release (Rytary or Numient) = 29.8% / Amantadine (Symmetrel) = 29.1% / Pramipexole (Mirapex) = 20.7%  Proportion of OFF episodes that are unpredictable (Unpredictability) [%]: 0% = 16.3% / <25% = 45.5% / 25-50% = 19.3% / >50% = 13.5% / Unknown = 5.3%  Duration of each OFF period, on average (duration) [%]: <15 min = 8.6% / 15-30 mins = 28.8% / 30-45 mins = 22.0% / 45-60 mins = 6.9% / >2 horas = 6.9% / Unknown = 6.5%  Years since initial onset of OFF periods (onset) [%]: <1 = 33.4% / 1-5 = 50.1% / 6-10 = 9.7% / >10 = 3.2% / Unknown = 3.7%  Frequency of OFF periods per day in an average week (frequency): 0 = 5.5% / 1 = 33.0% / 2 = 28.8% / 3 = 20.2% / >4 = 7.0% / Prefer not to answer = 5.5% | – |
| Peto (1995) [7] | United Kingdom | 359 | Age [mean] = 71.4 y / Range = 42.2-89.4  Women [%] = 42.6%  Duration of PD (years) [mean] = 9.4 / Range = (<1)-40 | PDQ-39 response rate = 82.0%  PDQ-39 scores for dimensions:   - Mobility [mean (SD)] = 64.56 (8.72) / Range = 0-100 / Floor effect = 2.2% / Ceiling effect = 8.4% - ADL [mean (SD)] = 57.85 (26.83) / Range = 0-100 / Floor effect = 1.7% / Ceiling effect = 5.1% - Emotional wellbeing [mean (SD)] = 39.49 (21.69) / Range = 0-100 / Floor effect = 3.2% / Ceiling effect = 0.9% - Stigma [mean (SD)] = 36.14 (27.93) / Range = 0-100 / Floor effect = 13.9% / Ceiling effect = 3.2% - Social support [mean (SD)] = 20.67 (20.56) / Range = 0-100 / Floor effect = 26.9% / Ceiling effect = 0.3% - Cognition [mean (SD)] = 49.46 (21.78) / Range = 0-100 / Floor effect = 0.3% / Ceiling effect = 0.9% - Communication [mean (SD)] = 36.78 (27.08) / Range = 0-100 / Floor effect = 16.8% / Ceiling effect = 1.7% - Bodily discomfort [mean (SD)] = 54.89 (25.95) / Range = 0-100 / Floor effect = 2.6% / Ceiling effect = 3.4% |
|  |  | 227 | Age [mean] = 70.3 y / Range = 40.9-87.7  Women [%] = 42.6%  Duration of PD (years) [mean] = 8.6 / Range = (<1)-40 | PDQ-39 response rate = 57.6% |
| Jenkinson (1997) [8] | United Kingdom | 405 | Age [mean (SD)] = 70.3 y (8.97) / Range = 40.9-87.7  Women [%] = 42.6%  Duration of PD (years) [mean] = 8.6 / Range = (<1)-32 | PDQ-39 response rate = 58.2%  PDQ-39 SI [mean (SD)] = 44.63 (17.62) / Range = 5.83-87.19  PDQ-39 scores for dimensions:   - Mobility [mean (SD)] = 66.59 (28.25) / 95CI = 62.8-70.4 - ADL [mean (SD)] = 55.82 (28.23) / 95CI = 52.1-59.6 - Emotional wellbeing [mean (SD)] = 43.25 (23.88) / 95CI = 40.1-46.4 - Stigma [mean (SD)] = 34.67 (29.00) / 95CI = 30.8-38.5 - Social support [mean (SD)] = 24.25 (24.08) / 95CI = 20.9-27.6 - Cognition [mean (SD)] = 47.41 (23.08) / 95CI = 44.4-50.4 - Communication [mean (SD)] = 37.09 (24.35) / 95CI = 33.9-40.3 - Bodily discomfort [mean (SD)] = 52.18 (24.04) / 95CI = 49.0-55.4 |
|  |  | 146 | Age [mean (SD)] = 66.09 y (9.02) / Range = 42-85.2  Women [%] = 40.4%  H&YS [%]: Stage 1 = 24.21% / Stage 2 = 41.91% / Stage 3 = 15.44% / Stage 4 = 15.44%  Duration of PD (years) [mean] = 6.73 / Range = (<1)-30 | PDQ-39 response rate = 93.15%  PDQ-39 SI [mean (SD)] = 31.62 (19.03) / Range = 0.78-85.88  PDQ-39 scores for dimensions:   - Mobility [mean (SD)] = 41.71 (31.62) / 95CI = 36.4-47.0 - ADL [mean (SD)] = 40.40 (28.02) / 95CI = 35.8-45.0 - Emotional wellbeing [mean (SD)] = 31.90 (22.13) / 95CI = 28.3-35.5 - Stigma [mean (SD)] = 30.86 (26.26) / 95CI = 26.2-35.2 - Social support [mean (SD)] = 13.69 (20.05) / 95CI = 10.3-17.1 - Cognition [mean (SD)] = 33.36 (22.91) / 95CI = 29.6-37.2 - Communication [mean (SD)] = 25.69 (22.95) / 95CI = 21.9-29.5 - Bodily discomfort [mean (SD)] = 40.79 (28.14) / 95CI = 36.1-45.4 |
| Jenkinson (1997) [9] | United Kingdom | 207 | Age [mean (SD)] = 70 y (9) / Range = 41-88  Women [%] = 43%  Duration of PD (years) = 8.6 / Range = (<1)-32 | **PDQ-39:**  PDQ-39 response rate = 58.2%  PDQ-39 SI [mean (SD)] = 44.63 (17.62) / Range = 5.83-87.19  PDQ-39 scores for dimensions:   - Mobility [mean (SD)] = 66.59 (28.25) / 95CI = 62.8-70.4 - ADL [mean (SD)] = 55.82 (28.23) / 95CI = 52.1-59.6 - Emotional wellbeing [mean (SD)] = 43.25 (23.88) / 95CI = 40.1-46.4 - Stigma [mean (SD)] = 34.67 (29.00) / 95CI = 30.8-38.5 - Social support [mean (SD)] = 24.25 (24.08) / 95CI = 20.9-27.6 - Cognition [mean (SD)] = 47.41 (23.08) / 95CI = 44.4-50.4 - Communication [mean (SD)] = 37.09 / DE = 24.35 / 95CI = 33.9-40.3 - Bodily discomfort [mean (SD)] = 52.18 (24.04) / 95CI = 49.0-55.4   **PDQ-8:**  PDQ-8 response rate = 58.2%  PDQ-8 score [mean (SD)] = 47.25 (20.96) / PDQ-8 score [median (IQR, p25-P75)] = 50 (31.25, 31.25-62.5) / Range = 0-100 |
| Martínez-Martín (1998) [10] | Spain | 109 | Age [mean (SD)] = 65.85 (10.25) / Range = 40-84  Women [%] = 46.6%  UPDRS-Total [mean (SD)] = 32.64 (18.34) [95CI = 29.03-36.25] / Range = 2-85  S&E [mean (SD)] = 75.34 (17.36) [95CI = 71.82-78.85] / Range = 30-100  SPMSQ [mean (SD)] = 0.54 (1.10) [95CI = 0.32-0.76] / Range = 0-6  GDS [mean (SD)] = 5.27 (3.68) [95CI = 4.55-6.00] / Range = 0-15  HADS [mean (SD)] = 13.55 (7.58) [95CI = 12.05-15.04] / Range = 1-30  H&YS [mean (SD)] = 2.46 (0.79)  Duration of PD (years) [mean (SD)] = 7.76 (5.63) / Range = 0.5-35 | PDQ-39 response rate = 94.5%  PDQ-39 scores for dimensions:   - Mobility [mean (SD)] = 40.51 (30.36) / 95CI = 34.54-46.48 - ADL [mean (SD)] = 39.52 (27.27) / 95CI = 34.12-44.91 - Emotional wellbeing [mean (SD)] = 36.97 (26.01) / 95CI = 31.88-42.06 - Stigma [mean (SD)] = 22.63 (26.81) / 95CI = 17.38-27.88 - Social support [mean (SD)] = 16.26 (23.84) / 95CI = 11.51-20.98 - Cognition [mean (SD)] = 25.12 (20.56) / 95CI = 21.07-29.16 - Communication [mean (SD)] = 21.68 (22.12) / 95CI = 17.35-26.01 - Bodily discomfort [mean (SD)] = 37.21 (25.48) / 95CI = 32.23-42.20 |
| Bushnell (1999) [11] | United States | 139 | Age [mean] = 69.5 y / Range = 45-94  Women [%] = 48%  Race / Ethnicity [%]: Caucasic = 95%  Maritage status [%]: Married = 81%  Education level [%]: College = 75%  Annual earning [%]: >$25,000 = 75% | PDQ-39 response rate = 41% |
| Andreu (2000) [12] | Canada | 126 | Age [mean (SD)] = 63.8 y (9.3) / Range = 32-83  Women [%] = 41.3%  Work status [%]: Retired = 70.6% / Still employed = 7.1% / Unemployed = 22.2%  Living arrangement [%]: With spouse = 69.8% / With their family = 19.8% / Alone = 8.7% / In institution = 1.6%  Levodopa users = 91.3%  Daily dose of levodopa [mean (SD)] = 772 (572)  Comorbidities [%, Yes] = 51.6%  Hypotension [%, Yes] = 17.6%  UPDRS-Part 2 [mean (SD)] = 14.6 (6.1)  UPDRS-Part 3 [mean (SD)] = 16.3 (9.8)  Depression [%]: No = 64.3% / Mild = 28.6% / Severe = 7.1%  H&YS [%]: Stage 1 = 17.5% / Stage 2 = 32.5% / Stage 3 = 34.1% / Stage 4 = 12.7% / Stage 5 = 3.2%  Duration of PD (years) [mean (SD)] = 9.1 (4.9) / Range = 1-30 | PDQ-39 SI [mean (SD)]: First administration = 32.6 (14.8) / Second administration = 30.3 (15.1)  PDQ-39 scores for dimensions:   - Mobility [mean (SD)]: First administration = 34.1 (22.8) / Second administration = 32.3 (23.2) - ADL [mean (SD)]: First administration = 34.9 (23.5) / Second administration = 31.4 (22.7) - Emotional wellbeing [mean (SD)]: First administration = 36.5 (20.8) / Second administration = 33.5 (21.4) - Stigma [mean (SD)]: First administration = 37.8 (24.8) / Second administration = 36.2 (23.9) - Social support [mean (SD)]: First administration = 14.6 (18.2) / Second administration = 14.2 (18.9) - Cognition [mean (SD)]: First administration = 33.6 (17.7) / Second administration = 40.0 (21.1) - Communication [mean (SD)]: First administration = 30.9 (19.9) / Second administration = 27.6 (19.9) - Bodily discomfort [mean (SD)]: First administration = 41.8 (22.4) / Second administration = 36.7 (21.3) |
| Schrag (2000) [13] | United Kingdom | 97 | Age [mean (SD)] = 73 y (11.3) / Range = 36-94  Women [%] = 48.45%  Age at PD onset [mean (SD)] = 67.6 y (12.5) / Range = 23.7-93.5  MMSE [mean (SD)] = 27.5 (3.6)  H&YS [mean (SD)] = 2.4 (0.9)  Duration of disease (years) [mean (SD)] = 5.8 (4.9) / Range = 0.2-25 | **PDQ-39:**  PDQ-39 response rate = 78.23%  **EQ-5D-3L:**  EQ-5D-3L Response rate = 78.23%  EQ-5D-3L Utility [median (IQR)] = 0.62 (0.29) / Range = (-0.59)-1  **SF-36:**  SF-36 response rate = 78.23% |
| Katsarou (2001) [14] | Greece | 119 | Age [mean (SD)] = 60.45 y (8.28) / Range = 37-79  Women [%] = 53.78%  Education (years) [mean (SD)] = 8.9 (2.3)  ADL – ON [mean (SD)] = 83.80 (9.60) / Range = 50-100  ADL – OFF [mean (SD)] = 61.87 (33.78) / Range = 30-90  UPDRS – ON [mean (SD)] = 28.17 (10.19) / Range = 4-59  UPDRS – OFF [mean (SD)] = 36.39 (24.58) / Range = 16-75  BDI [mean (SD)] = 7.60 (5.68) / Range = 1-25  H&YS [%]: Stage 1 = 5.04% / Stage 2 = 44.54% / Stage 3 = 29.41% / Stage 4 = 21.01%  Duration of PD (years) [mean (SD)] = 6.67 (3.75) / Range = 1-19 | PDQ-39 scores for dimensions:   - Mobility [mean (SD)]= 29.68 (24.69) / Range = 0-92.50 / 95CI = 25.20-34.17 - ADL [mean (SD)] = 39.85 (22.06) / Range = 0-95.83 / 95CI = 35.84-43.85 - Emotional wellbeing [mean (SD)] = 29.73 (23.76) / Range = 0-95.83 / 95CI = 25.41-34.04 - Stigma [mean (SD)] = 23.79 (26.98) / Range = 0-93.75 / 95CI = 18.89-28.69 - Social support [mean (SD)] = 9.31 (17.90) / Range = 0-83.33 / 95CI = 6.06-12.56 - Cognition [mean (SD)] = 20.48 (17.68) / Range = 0-87.50 / 95CI = 17.27-23.69 - Communication [mean (SD)] = 21.08 (19.97) / Range = 0-66.67 / 95CI = 17.45-24.70 - Bodily discomfort [mean (SD)] = 26.40 (21.90) / Range = 0-83.33 / 95CI = 22.43-30.38 |
| Peto (2001) [15] | United Kingdom | 851 | Age [mean (SD)] = 72.8 y (70.4) / Range = 32.9-90.9  Women [%] = 41.1%  Maritage status [%]: Married = 75.6%  Living arrangement [%]: With other adult = 80.3%  Work status [%]: Active = 4.7%  Needed help to fulfill the questionnaire [%, Yes] = 29.4% | PDQ-39 response rate = 65%  PDQ-39 SI [mean (SD)]: First administration = 44.0 (15.85) / Second administration = 45.6 (15.57)  PDQ-39 scores for dimensions:   - Mobility [mean (SD)]: First administration = 63.07 (27.82) / Second administration = 66.23 (25.47) - ADL [mean (SD)]: First administration = 52.93 (24.95) / Second administration = 57.37 (24.10) - Emotional wellbeing [mean (SD)]: First administration = 46.20 (21.86) / Second administration = 50.39 (19.86) - Stigma [mean (SD)]: First administration = 42.17 (26.88) / Second administration = 47.73 (24.99) - Social support [mean (SD)]: First administration = 37.63 (26.69) / Second administration = 48.99 (21.32) - Cognition [mean (SD)]: First administration = 51.86 (21.40) / Second administration = 53.62 (20.00) - Communication [mean (SD)]: First administration = 47.04 (24.61) / Second administration = 51.27 (21.21) - Bodily discomfort [mean (SD)]: First administration = 61.16 (23.13) / Second administration = 63.27 (31.35) |
| Tsang (2002) [16] | China | 54 | Age [mean (SD)] = 66.4 y (10) / Range = 42-82  Women [%] = 42.6%  Marital status [%]: Single = 1.9% / Married = 83.3% / Widowed = 14.8%  Education level [%]: None = 1.9% / Primary School = 51.8% / Junior High School = 16.6% / Senior High School = 24.1% / College = 5.6%  Work status [%]: Still Working = 3.7% / Retired = 96.3%  H&YS [%]: Stage 1+2 = 40.7% / Stage 3+4 = 59.3% | PDQ-39 SI [mean (SD)] = 29.5 (21.5)  PDQ-39 scores for dimensions:   - Mobility [mean (SD)] = 43.9 (21.8) / Ceiling effect = 0.0% - ADL [mean (SD)] = 33.1 (23.8) / Ceiling effect = 5.6% - Emotional wellbeing [mean (SD)] = 28.9 (21.8) / Ceiling effect = 16.7% - Stigma [mean (SD)] = 28.3 (18.5) / Ceiling effect = 9.3% - Social support [mean (SD)] = 16.3 (23.0) / Ceiling effect = 40.7% - Cognition [mean (SD)] = 34.0 (19.2) / Ceiling effect = 1.9% - Communication [mean (SD)] = 24.4 (22.1) / Ceiling effect = 20.4% - Bodily discomfort [mean (SD)] = 26.9 (21.8) / Ceiling effect = 20.4% |
| Hagell (2003) [17] | Sweden | 71 | Age [mean (SD)] = 69.1 y (10.2) / Age [median] = 71 y  Women [%] = 38.0%  Daily administrations of antiparkinsonian medication [mean (SD)] = 5.6 (2.7)  Duration of PD (years) [mean (SD)] = 10.4 (9.4) | PDQ-39 scores for dimensions:   - Mobility [median (IQR)] = 36.2 (12.5-80.6) / Range = 0-100 / Floor effect = 10.6% / Ceiling effect = 3.0% - ADL [median (IQR)] = 37.5 (16.7-58.7) / Range = 0-100 / Floor effect = 10.3% / Ceiling effect = 1.5% - Emotional wellbeing [median (IQR)] = 33.3 (14.6-50) / Range = 0-93.8 / Floor effect = 8.7% / Ceiling effect = 0.0% - Stigma [median (IQR)] = 25.0 (6.2-50) / Range = 0-91.7 / Floor effect = 21.1% / Ceiling effect = 0.0% - Social support [median (IQR)] = 0.0 (0-16.7) / Range = 0-100 / Floor effect = 54.8% / Ceiling effect = 0.0% - Cognition [median (IQR)] = 31.2 (12.5-43.8) / Range = 0-100 / Floor effect = 14.1% / Ceiling effect = 1.4% - Communication [median (IQR)] = 16.7 (0-41.7) / Range = 0-83.3 / Floor effect = 28.6% / Ceiling effect = 0.0% - Bodily discomfort [median (IQR)] = 33.3 (16.7-52.1) / Range = 0-91.7 / Floor effect = 12.9% / Ceiling effect = 0.0% |
| Jenkinson (2003) [18] | United States, Canada, Italy, Spain and Japan | 812 | Age [mean (SD)] = 68.35 y (9.49) / Range = 33-94  Women [%] = 41% | PDQ-39 response rate = 82.54%  PDQ-39 observed floor effect:   - Mobility: United States = 8.6% / Canada = 5.5% / Italy = 2.9% / Spain = 8.0% / Japan = 4.0% - ADL: United States = 8.6% / Canada = 6.3% / Italy = 6.3% / Spain = 12.0% / Japan = 5.0% - Emotional wellbeing: United States = 12.4% / Canada = 6.3% / Italy = 2.0% / Spain = 6.5% / Japan = 5.9% - Stigma: United States = 31.4% / Canada = 25.0% / Italy = 17.6% / Spain = 45.0% / Japan = 31.7% - Social support: United States = 50.8% / Canada = 52.8% / Italy = 2.9% / Spain = 68.5% / Japan = 56.4% - Cognition: United States = 5.4% / Canada = 6.3% / Italy = 9.8% / Spain = 15.5% / Japan = 11.9% - Communication: United States = 5.4% / Canada = 25.8% / Italy = 19.0% / Spain = 46.5% / Japan = 39.6% - Bodily discomfort: United States = 10.3% / Canada = 3.9% / Italy = 9.8% / Spain = 10.5% / Japan = 13.9% - SI: United States = 0.0% / Canada = 0.0% / Italy = 0.0% / Spain = 0.0% / Japan = 0.0%   PDQ-39 observed ceiling effect:   - Mobility: United States = 3.2% / Canada = 3.1% / Italy = 6.8% / Spain = 3.0% / Japan = 4.0% - ADL: United States = 4.9% / Canada = 2.3% / Italy = 5.4% / Spain = 3.5% / Japan = 6.0% - Emotional wellbeing: United States = 2.2% / Canada = 0.8% / Italy = 2.0% / Spain = 1.0% / Japan = 1.0% - Stigma: United States = 1.1% / Canada = 2.3% / Italy = 1.0% / Spain = 0.5% / Japan = 0.0% - Social support: United States = 1.1% / Canada = 0.0% / Italy = 1.0% / Spain = 1.5% / Japan = 0.0% - Cognition: United States = 1.6% / Canada = 0.8% / Italy = 1.0% / Spain = 0.5% / Japan = 0.0% - Communication: United States = 1.6% / Canada = 0.8% / Italy = 0.5% / Spain = 1.0% / Japan = 0.0% - Bodily discomfort: United States = 2.7% / Canada = 3.9% / Italy = 1.5% / Spain = 0.5% / Japan = 2.0% - SI: United States = 1.1% / Canada = 0.0% / Italy = 0.5% / Spain = 0.0% / Japan = 0.0% |
| Park (2004) [19] | South Korea | 14 | Age [mean (SD] = 72.15 y (5.98)  Women [%] = 84.62% | – |
| Tan (2004) [20] | Singapore | 88 | Age [mean (SD)] = 63.1 y (9.6) / Range = 38-82  Women [%] = 29.5%  Years of education [%]: ≤6 years = 5.6% / 7-11 years = 52.3% / ≥12 years = 38.7% / Missing = 3.4%  Marital status: Unmarried = 4.5% / Married = 80.7% / Divorced/Separated = 4.5% / Widowed = 10.2%  Ethnicity: China = 84.1% / Malaysia = 3.4% / India = 10.2% / Other = 2.3%  Work status: Employed = 19.4% / Unemployed/Homeworker = 20.4% / Retired = 60.2%  Comorbidities [%, Yes] = 62.5% | **PDQ-39:**  PDQ-39 SI [mean (SD)] = 31.2 (14.9) / PDQ-39 SI [median] = 31.2 / Range = 18-40  PDQ-39 scores for dimensions:   - Mobility [mean (SD)] = 38.2 (28.0) / Median = 37.5 / Range = 0-100 / Floor effect = 8.0% / Ceiling effect = 1.1% - ADL [mean (SD)] = 31.6 (25.2) / Median = 29.2 / Range = 0-100 / Floor effect = 12.5% / Ceiling effect = XXX% - Emotional wellbeing [mean (SD)] = 29.5 (20.1) / Median = 29.2 / Range = 0-79.2 / Floor effect = 9.1% / Ceiling effect = 2.3% - Stigma [mean (SD)] = 27.3 (24.2) / Median = 18.8 / Range = 0-93.8 / Floor effect = 20.5% / Ceiling effect = 0.0% - Social support [mean (SD)] = 15.4 (19.5) / Median = 8.3 / Range = 0-75.0 / Floor effect = 46.6% / Ceiling effect = 0.0% - Cognition [mean (SD)] = 30.2 (19.7) / Median = 31.3 / Range = 0-81.3 / Floor effect = 9.1% / Ceiling effect = 0.0% - Communication [mean (SD)] = 32.0 (22.2) / Median = 29.2 / Range = 0-100 / Floor effect = 14.8% / Ceiling effect = 1.1% - Bodily dicomfort [mean (SD)] = 37.3 (20.2) / Median = 33.3 / Range = 0-91.7 / Floor effect = 5.7% / Ceiling effect = 0.0%   **PDQ-8:**  PDQ-8 score [mean (SD)] = 31.6 (16.8) / PDQ-8 [median] = 31.3 / Range = 15.6-43.8  PDQ-8 floor effect = 1.1%  PDQ-8 ceiling effect = 0.0% |
| Fitzpatrick (2004) [21] | United Kingdom | 800 | Participants from community:  Age [mean] = 70.4 y / Range = 33-91  Women [%] = 41.1%  Duration of PD (years) [mean] = 8.6 / Range = 1-40 | PDQ-39 response rate = 91.0%  PDQ-39 SI [mean (SD]: First administration = 38.48 (17.79) / Second administration = 40.96 (18.15)  PDQ-39 scores for dimensions:   - Mobility [mean (SD)]: First administration = 57.88 (30.33) / Second administration = 60.46 (30.11) - ADL [mean (SD)]: First administration = 50.33 (27.44) / Second administration = 52.73 (27.76) - Emotional wellbeing [mean (SD)]: First administration = 37.31 (22.86) / Second administration = 38.29 (23.59) - Stigma [mean (SD)]: First administration = 30.31 (25.76) / Second administration = 30.56 (25.98) - Social support [mean (SD)]: First administration = 16.21 (20.31) / Second administration = 25.35 (24.53) - Cognition [mean (SD)]: First administration = 42.66 (23.90) / Second administration = 43.47 (23.71) - Communication [mean (SD)]: First administration = 32.96 (25.48) / Second administration = 35.54 (26.04) - Bodily discomfort [mean (SD)]: First administration = 48.99 (25.35) / Second administration = 48.47 (25.13) |
|  | United Kingdom | 146 | Participants from clinical practice:  Age [mean] = 66.1 y / Range = 42-58  Women [%] = 40.4%  Duration of PD (years) [mean] = 6.7 / Range = (<1)-30 | PDQ-39 response rate = 90.4%  PDQ-39 SI [mean (SD]: First administration = 31.2 (19.08) / Second administration = 31.81 (18.55)  PDQ-39 scores for dimensions:   - Mobility [mean (SD)]: First administration = 39.29 (30.35) / Second administration = 41.81 (30.52) - ADL [mean (SD)]: First administration = 39.52 (27.88) / Second administration = 39.74 (27.71) - Emotional wellbeing [mean (SD)]: First administration = 31.33 (21.82) / Second administration = 30.76 (21.33) - Stigma [mean (SD)]: First administration = 29.78 (25.90) / Second administration = 29.36 (24.48) - Social support [mean (SD)]: First administration = 13.54 (19.84) / Second administration = 15.10 (21.28) - Cognition [mean (SD)]: First administration = 33.48 (23.19) / Second administration = 33.14 (22.48) - Communication [mean (SD)]: First administration = 25.19 (22.98) / Second administration = 26.15 (21.60) - Bodily discomfort [mean (SD)]: First administration = 40.83 (28.09) / Second administration = 41.86 (26.79) |
| Haapaniemi (2004) [22] | Finland | 259 | Age [mean (SD)] = 66.5 y (10.6)  Women [%] = 47.1%  Age at diagnosis [mean (SD)] = 60.4 y (10.8)  Presence of comorbidities [%]: Any comorbidity = 71.0% / Depression = 28.6% / Dementia = 9.7%  UPDRS-Motor [mean (SD)] = 24.1 (12.0)  UPDRS-ADL [mean (SD)] = 10.2 (6.5)  S&E [mean] = 82.6  Motor fluctuations [%] = 28.5%  H&YS [%]: Stage 0 = 0.4% / Stage 1 = 18.1% / Stage 1.5 = 15.1% / Stage 2 = 26.6% / Stage 2.5 = 15.1% / Stage 3 = 16.2% / Stage 4 = 8.5% / Stage 5 = 0.0%  Duration of disease (years) [mean (SD)] = 4.3 (4.4) | **PDQ-39:**  PDQ-39 SI [mean] = 31.6  PDQ-39 scores for dimensions:   - Mobility [mean (95CI)] = 32.9 (28.6-37.2) - ADL [mean (95CI)] = 35.7 (31.9-39.5) - Emotional wellbeing [mean (95CI)] = 33.5 (30.9-36.1) - Stigma [mean (95CI)] = 29.8 (26.6-32.9) - Social support [mean (95CI)] = 22.3 (18.7-25.9) - Cognition [mean (95CI)] = 33.2 (30.4-35.9) - Communication [mean (95CI)] = 22.7 (20.5-24.9) - Bodily discomfort [mean (95CI)] = 34.3 (31.8-36.7)   **15D:**  15D Utility [mean (SD)] = 0.70 (0.13)  Mobility [mean (SD)] = 0.78 (0.20)  Vision [mean (SD)] = 0.88 (0.18)  Hearing [mean (SD)] = 0.92 (0.15)  Breathing [mean (SD)] = 0.80 (0.23)  Sleeping [mean (SD)] = 0.77 (0.21)  Eating [mean (SD)] = 0.85 (0.19)  Speech [mean (SD)] = 0.80 (0.20)  Elimination [mean (SD)] = 0.74 (0.22)  Usual activities [mean (SD)] = 0.69 (0.22)  Mental function [mean (SD)] = 0.76 (0.21)  Discomfort and symptoms [mean (SD)] = 0.70 (0.21)  Depression [mean (SD)] = 0.82 (0.17)  Distress [mean (SD)] = 0.79 (0.18)  Vitality [mean (SD)] = 0.73 (0.17)  Sexual activity [mean (SD)] = 0.65 (0.30) |
| Martínez-Martín (2004) [23] | Ecuador (Quito) | 137 | Age [mean (SD)] = 69.4 y (10.1) / Range = 44-92  Women [%] = 32.1%  H&YS [%]: Stage 1 = 8.0% / Stage 1.5 = 19.7% / Stage 2 = 20.4% / Stage 2.5 = 26.3% / Stage 3 = 18.3% / Stage 4 = 7.3%  Duration of PD (years) [mean (SD)] = 5.9 (2.6) / Range = 1-15 | PDQ-39 SI [mean (SD)] = 41.5 (15.5) / Range = 8.8-81.7 / Floor effect = 0.7% / Ceiling effect = 0.7%  PDQ-39 scores for dimensions:   - Mobility [mean (SD)] = 48,5 (23,9) / Range = 0-100 - ADL [mean (SD)] = 39,9 (26,3) / Range = 0-91,6 - Emotional wellbeing [mean (SD)] = 49,6 (16,8) / Range = 7,8-100 - Stigma [mean (SD)] = 47,2 (29,1) / Range = 0-100 - Social support [mean (SD)] = 30,7 (21,5) / Range = 0-83,3 - Cognition [mean (SD)] = 36,2 (20,6) / Range = 0-87,5 - Communication [mean (SD)] = 35,7 (19,3) / Range = 0-83,3 - Bodily discomfort [mean (SD)] = 43,8 (22,7) / Range = 0-100 |
| Ma (2005) [24] | China (Taiwan) | 73 | Age [mean (SD)] = 69.02 y (9.56) / Range = 47-90  Women [%] = 42.5%  Marital status [%]: Single = 4.1% / Married = 89.0% / Widowed = 6.9%  Education level [%]: None = 15.1% / Primary School = 35.6% / Junior High School = 13.7% / Senior High School = 16.4% / College = 19.2%  Work status [%]: Paid Job = 21.9% / Housewife = 11.0% / Not Working = 67.1%  H&YS [%]: Stage 1 = 17.11% / Stage 2 = 50.68% / Stage 3 = 13.70% / Stages 4+5 = 17.81%  Duration of PD (years) [mean (SD)] = 7.93 (5.34) / Range = 2-34 | – |
| Luo (2005) [25] | Singapore | 63 | Age [mean (SD)] = 65 y (8.5) / Range = 41-82  Women [%] = 41.3%  Years of education [%]: ≤6 years = 39.6% / 7-11 years = 33.3% / ≥12 years = 27.0%  Marital status: Unmarried/Divorced = 3.2% / Married = 90.4% / Widowed = 6.3%  Work status [%]: Employed = 9.5% / Unemployed/Homeworker = 34.9% / Retired = 55.6%  Chronic comorbidities [%, Yes] = 66.7% | **PDQ-39:**  PDQ.39 response rate = 28%  PDQ-39 SI [mean (SD)] = 21.7 (14.7) / PDQ-39 SI [median (IQR, P25-P75)] = 17.3 (20.8, 10.9-31.7)  PDQ-39 scores for dimensions:   - Mobility [mean (SD)] = 29,9 (23,7) / Median [P25-P75] = 25,0 (12,5-42,5) / Floor effect = 4.8% - ADL [mean (SD)] = 19,8 (23,3) / Median [P25-P75] = 12,5 (0-29,2) / Floor effect = 25.4% - Emotional wellbeing [mean (SD)] = 25,0 (23,2 /) Median [P25-P75] = 18,8 (4,2-41,7) / Floor effect = 14.3% - Stigma [mean (SD)] = 19,1 (21,0) / Median [P25-P75] = 12,5 (0-31,3) / Floor effect = 38.1% - Social support [mean (SD)] = 13,4 (19,5) / Median [P25-P75] = 0 (0-25) / Floor effect = 58.7% - Cognition [mean (SD)] = 26,0 (19,1) / Median [P25-P75] = 25,0 (12,5-37,5) / Floor effect = 14.3% - Communication [mean (SD)] = 22,9 (22,6) / Median [P25-P75] = 16,7 (0-33,3) / Floor effect = 31.7% - Bodily discomfort [mean (SD)] = 24,9 (20,4) / Median [P25-P75] = 25,0 (8,3-33,3) / Floor effect = 19.0%   **PDQ-8:**  PDQ-8 response rate = 28.0%  PDQ-8 score [mean (SD)] = 21.9 (17.2) / PDQ-8 score [median (IQR, P25-P75] = 18.8 (25, 6.3-31.3)  PDQ-8 floor effect = 4.8% |
| Martínez-Martín (2007) [26] | Ecuador (Quito) | 188 | Age [mean (SD)] = 68.93 y (10.39) / Age [median] = 70 y / Range = 44-96  Women [%] = 33.2%  Time on treatment with levodopa (years) [mean (SD)] = 4.85 (2.86) / Median = 5 / Range = 0-15  H&YS [median] = 2,5 / Range = 1-4  H&YS [%]: Stage 1 = 5.9% / Stage 1.5 = 15.0% / Stage 2 = 21.4% / Stage 2.5 = 2.5% / Stage 3 = 27.3% / Stage 4 = 7.0%  Duration of PD (years) [mean (SD)] = 6.0 (3.02) / Median = 6 / Range = 1-15 | **PDQ-39:**  PDQ-39 SI [mean (SD)] = 41.82 (16.25) / PDQ-39 SI [median] = 40.42 / Range = 8.85-81.77  PDQ-39 scores for dimensions:   - Mobility [mean (SD)] = 47.90 (24.05) / Median = 50 / Range = 0-100 - ADL [mean (SD)] = 41.64 (26.10) / Median = 37.5 / Range = 0-95.83 - Emotional wellbeing [mean (SD)] = 48.4 (20.15) / Median = 45.83 / Range = 4.17-100 - Stigma [mean (SD)] = 48.13 (29.09) / Median = 50 / Range = 0-100 - Social support [mean (SD)] = 31.60 (22.61) / Median = 33.33 / Range = 0-100 - Cognition [mean (SD)] = 33.16 (20.15) / Median = 31.25 / Range = 0-87.5 - Communication [mean (SD)] = 36.81 (21.13) / Median = 33.33 / Range = 0-100 - Bodily discomfort [mean (SD)] = 46.97 (24.07) / Median = 41.67 / Range = 0-100   No ceiling or floor effect were observed.  **PDQL:**  PDQL score [mean (SD)] = 108.88 (24.63) / PDQL score [median] = 106 / Range = 50-162  PDQL scores for dimensions:   - Parkinsonian symptoms [mean (SD)] = 42.63 (10.37) / Median = 43 / Range = 15-63 - Systemic symptoms [mean (SD)] = 21.05 (5.33) / Median = 21 / Range = 8-34 - Emotional functioning [mean (SD)] = 27.24 (6.71) / Median = 26 / Range = 14-43 - Social functioning [mean (SD)] = 17.97 (6.13) / Median = 17 / Range = 7-33 |
| Hagell (2007) [27] | Sweden | 202 | Age [mean (SD)] = 69.8 y (10) / Range = 34-90  Women [%] = 46.5%  Retired [%, Yes] = 70.8%  Married or cohabitant [%, Yes] = 71.2%  Living in their own home [%, Yes] = 88.6%  Perceived severity of PD (1 = Mild / 2 = Moderate / 3 = Severe) [Mediana (P25-P75)] = 2 (2-2) / Range = 1-3  Motor fluctuations [%, Yes] = 67.8%  Diskynesia [%, Yes] = 49%  H&YS [Mediana (P25-P75)] = 3 (2-4) / Range = 1-5 | PDQ-39 response rate = 81%  PDQ-39 scores for dimensions:   - Mobility [mean (SD)] = 42.95 (28.43) / Median = 45 / IQR (P25-P75) = 20-62.5 / Floor effect = 11.4% / Ceiling effect = 2.5% - ADL [mean (SD)] = 38.94 (24.76) / Median = 37.5 / IQR (P25-P75) = 20.8-58 / Floor effect = 7.9% / Ceiling effect = 1.0% - Emotional wellbeing [mean (SD)] = 37.92 (21.05) / Median = 37.5 / IQR (P25-P75) = 20.8-54 / Floor effect = 5.4% / Ceiling effect = 0.5% - Stigma [mean (SD)] = 27.54 (23.17) / Median = 25 / IQR (P25-P75) = 6.2-43.8 / Floor effect = 20.3% / Ceiling effect = 0.5% - Social support [mean (SD)] = 14.78 (18.08) / Median = 8.3 / IQR (P25-P75) = 0-25 / Floor effect = 35.6% / Ceiling effect = 0.0% - Cognition [mean (SD)] = 33.03 (20.35) / Median = 31.2 / IQR (P25-P75) = 18.8-50 / Floor effect = 6.9% / Ceiling effect = 0.0% - Communication [mean (SD)] = 27.99 (24.19) / Median = 25 / IQR (P25-P75) = 6.2-41.7 / Floor effect = 24.3% / Ceiling effect = 0.5% - Bodily discomfort [mean (SD)] = 40.91 (24.07) / Median = 41.7 / IQR (P25-P75) = 25-58.3 / Floor effect = 7.9% / Ceiling effect = 0.5% |
| Krikmann (2008) [28] | Estonia | 81 | Age [mean (SD)] = 66.9 y (8.2) / Range = 48-85  Women [%] = 32.1%  Treatment with levodopa [%. Yes]= 91.4%  Combination of antiparkinsonian treatments [%, Yes] = 59.3%  Adverse events (dyskinesia, motor fluctuations…) [%, Yes] = 54.3%  Comoborbidities [%, Yes]: Hypertension = 24% / Coronary disease = 24% / Articular disease = 14% / Stroke = 2.5% / Cancer = 1.2%  Living in urban areas [%, Yes] = 60%  H&YS [%]: Stages 1+2 = 49.3% / Stage 3 = 35.8% / Stages 4+5 = 4.9  Duration of PD (years) [mean (SD)] = 8.9 (6.3) / Range = 1-35 | PDQ-39 response rate = 79%  PDQ-39 SI [mean (SD)] = 39.0 (17.7) / PDQ-39 SI [median] = 38.6  PDQ-39 scores for dimensions:   - Mobility [mean (SD)] = 46,7 (26,1) / Median = 47,5 - ADL [mean (SD)] = 44,1 (25,1) / Median = 41,7 - Emotional wellbeing [mean (SD)] = 39,8 (25,0) / Mediana = 37,5 - Stigma [mean (SD)] = 34,2 (28,3) / Median = 31,3 - Social support [mean (SD)] = 27,6 (24,5) / Median = 25,0 - Cognition [mean (SD)] = 35,2 (21,8) / Median = 41,7 - Communication [mean (SD)] = 30,6 (20,8) / Median = 33,3 - Bodily discomfort [mean (SD)] = 45,3 (22,5) / Median = 50,0 |
| Marinus (2008) [29] | Germany (Leiden) | 177 | Age [mean (SD)] = 65.2 y (11.1)  Women [%] = 44%  Age at diagnosis [mean (SD)] = 55.7 y (11.6)  Status of PD (H&YS): Mild (Stages 1+2) = 42% / Moderate (Stage 3) = 39% / Sever (Stages 4+5) = 19%  H&YS [%]: Stage 1 = 5.65% / Stage 2 = 36.16% / Stage 3 = 39.55% / Stage 4 = 17.51% / Stage 5 = 1.13% | PDQ-39 response rate = 86% |
| Serrano-Dueñas (2008) [30] | Ecuador (Quito) | 131 | Age [mean (SD)] = 68.8 y (11.0) / Range = 4-96  Women [%] = 29.7%  Yeras with levodopa [mean (SD)] = 5.9 (4.7) / Range = 0-22  Levodopa doce (mg/day) [mean (SD)] = 767.3 (370.4) / Range = 0-1500  Pfeiffer [mean (SD)] = 1.7 (1.6) / Range = 0-6  HADS-A [mean (SD] = 9.3 (5.2) / Range = 1-21  HADS-D [mean (SD)] = 10.1 (5.1) / Range = 0-21  SPES/SCOPA [mean (SD)] = 26.5 (10.6) / Range = 8-56  UPDRS-Total [mean (SD)] = 69.2 (21.6) / Range = 25-125  S&E [mean (SD)] = 66.7 (16.8) / Range = 20-90  Motor fluctuations [%, Yes] = 72.5%  H&YS [%]: Stage 1.5 = 4.6% / Stage 2 = 19.1% / Stage 2.5 = 14.5% / Stage 3 = 53.44% / Stage 4 = 8.4% / Stage 5 = 3.1%  Duration of PD (years) [mean (SD)] = 7.8 (5.8) / Range = 0.5-30 | **PDQ-39:**  –  **PDQL:**  –  **PIMS:**  PIMS response rate = 96.2%  PIMS score [mean (SD, Range)]: Non-Fluctuators = 18.4 (7.4, 0-34) / Fluctuators = 25.3 (5.8, 14.5-38.5)  Floor effect [%] = 1.1% / Ceiling effect = 2.8% |
| Žiropađa (2009) [31] | Serbia (Belgrado) | 102 | Age [mean (SD)] = 58.4 y (9.9)  Women [%] = 46.08%  UPDRS-Part 3 [mean (SD)]) = 33 (12.8)  MMSE [mean (SD)] = 27.3 (2.3)  BDI [mean (SD)] = 14.0 (5.9)  H&YS [%]: Stage 1 = 39.2% / Stage 2 = 45.1% / Stage 3 = 13.7% / Stage 4 = 2.0%  Duration of PD (years) [mean (SD)] = 4.76 (4.27) | PDQ-39 SI [mean (SD] = 45.83 (13.31) / Range = 22-86  PDQ-39 scores for dimensions:   - Mobility [mean (SD)] = 44.66 (17.06) / Range = 20-98 - ADL [mean (SD)] = 56.75 (17.27) / Range = 20-93 - Emotional wellbeing [mean (SD)] = 44.52 (19.64) / Range = 20-100 - Stigma [mean (SD)] = 44.00 (18.50) / Range = 20-100 - Social support [mean (SD)] = 47.56 (17.30) / Range = 20-80 - Cognition [mean (SD)] = 42.72 (13.79) / Range = 20-80 - Communication [mean (SD)] = 36.50 (14.47) / Range = 20-87 - Bodily discomfort [mean (SD)] = 49.96 (22.43) / Range = 14-100 |
| Nojomi (2010) [32] | Iran (Teheran) | 240 | Age [mean (SD)] = 57.3 y (10.5) / Range = 27-83  Women [%] = 32.5%  Education level [%]: Less than High School = 40% / High School Graduated = 28% / Academic = 21% / Unknown = 11%  Work status [%]: Housewife = 27% / Retired = 32% / Still Working = 30% / Unemployed = 11%  Marital status [%]: Married = 89% / Single = 2.5% / Other = 8.5%  Severity of disease [%]: Mild = 46.5% / Moderate = 40% / Severe = 11.5% / Unknown = 2%  Age at debut of PD [mean (SD)] = 50.6 y (11.0)  Duration of PD (years) = 6.5 (4.2) | PDQ-39 response rate = 83%  PDQ-39 SI [mean (SD)] = 35.1 (15.4) / 95CI = 23.1-36.7  PDQ-39 scores for dimensions:   - Mobility [mean (SD)] = 44.5 (23.1) / 95CI = 41.0-47.6 - ADL [mean (SD)] = 39.1 (22.3) / 95CI = 35.2-42.0 - Emotional wellbeing [mean (SD)] = 37.2 (21.0) / 95CI = 32.8-39.1 - Stigma [mean (SD)] = 33.9 (25.1) / 95CI = 29.8-37.4 - Social support [mean (SD)] = 22.9 (20.4) / 95CI = 20.0-26.0 - Cognition [mean (SD)] = 28.8 (16.8) / 95CI = 27.0-32.2 - Communication [mean (SD)] = 28.1 (21.0) / 95CI = 25.1-31.5 - Bodily discomfort [mean (SD)] = 42.0 (23.5) / 95CI = 38.5-45.9 |
| Luo (2010) [33] | China | 71 | Age [mean (SD)] = 63.66 y (13.06) / Range = 32-84  PROM’s administration: Interview = 11.3% / Self-administered = 88.7% (no significant differences in scores)  Education level [%]: None = 8.5% / Primary School = 23.9% / Junior Secondary School = 26.8% / Senior Secondary School = 22.5% / University = 18.3%  Marital status [%]: Single = 11.4% / Married = 80.0% / Separation = 2.9% / Widowed = 5.7%  Work status: Still Working = 12.3% / Retired = 87.7%  MMSE [mean (SD)] = 27.47 (3.34)  H&YS [%: Stage 1 = 23.94% / Stage 2 = 53.52% / Stage 3 = 11.27% / Stages 4+5 = 11.27%  Duration of PD (years) [mean (SD)] = 6 (4.16) / Range = 1-20 | PDQ-39 SI [mean (SD)] = 29.63 (17.35) / PDQ-39 SI [median (IQR, P25-P75)] = 29.1 (24.5, 15.1-39.6) / Floor effect = 0.0% / Ceiling effect = 0.0%  PDQ-39 scores for dimensions:   - Mobility [mean (SD)] = 33.73 (28.12) / Median = 30 / IQR (P25-P75) = 10-52.5 / Floor effect = 12.7% / Ceiling effect = 1.4% - ADL [mean (SD)] = 28.35 (27.37) / Median = 20.8 / IQR (P25-P75) = 4.2-41.7 / Floor effect = 16.9% / Ceiling effect = 4.2% - Emotional wellbeing [mean (SD)] = 31.57 (22.29) / Median = 25.0 / IQR (P25-P75) = 16.7-45.8 / Floor effect = 4.2% / Ceiling effect = 0.0% - Stigma [mean (SD)] = 24.56 (27.49) / Median = 18.8 / IQR (P25-P75) = 0-37.5 / Floor effect = 32.4% / Ceiling effect = 1.4% - Social support [mean (SD)] = 19.48 (22.67) / Median = 16.7 / IQR (P25-P75) = 0-33.3 / Floor effect = 43.7% / Ceiling effect = 0.0% - Cognition [mean (SD)] = 35.65 (21.74) / Median = 31.3 / IQR (P25-P75) = 18.8-43.8 / Floor effect = 2.8% / Ceiling effect = 2.8% - Communication [mean (SD)] = 28.64 (27.01) / Median = 16.7 / IQR (P25-P75) = 0-58.3 / Floor effect = 26.8% / Ceiling effect = 0.0% - Bodily discomfort [mean (SD)] = 35.09 (23.98) / Median = 25.0 / IQR (P25-P75) = 16.7-50.0 / Floor effect = 0.0% / Ceiling effect = 1.4% |
| Huang (2010) [34] | China (Taiwan) | 100 | Age [mean (SD)] = 62.04 y (11.33)  Women [%] = 44.0%  Daily dose of levodopa (mg) [mean (SD)] = 481.75 (356.22)  UPDRS-Total [mean (SD)] = 43.80 (23.12)  UPDRS-Mental [mean (SD)] = 3.21 (2.46)  UPDRS-ADL [mean (SD)] = 26.26 (14.43)  UPDRS-Motor [mean (SD)] = 1.83 (2.50)  Complications [%, Yes] = 1.83 (2.50)  S&E [mean (SD)] = 73.59 (19.04)  Pain [mean (SD)] = 2.07 (3.17) / Pain [%, Yes] = 43%  TDQ [mean (SD)] = 19.12 (11.92) / Depression (TDQ > 19) [%, Yes] = 44%  PSQI [mean (SD)] = 9.75 (5.15) / Sleep disturbances (PSQI > 5) [%, Yes] = 78%  H&YS [mean (SD)] = 2.21 (1.18)  Duration of PD (years) [mean (SD)] = 7.1 (5.15) | **PDQ-39:**  –  **PDQ-8:**  PDQ-8 scores for items (dimensions):   - Mobility [mean (SD)] = 1.37 (1.53) / Range = 0-4 - ADL [mean (SD)] = 0.97 (1.50) / Range = 0-4 - Emotional wellbeing [mean (SD)] = 1.51 (1.50) / Range = 0-4 - Stigma [mean (SD)] = 1.57 (1.64) / Rango = 0-4 - Social support [mean (SD)] = 0.78 (1.31) / Range = 0-4 - Cognition [mean (SD)] = 1.36 (1.42) / Range = 0-4 - Communication [mean (SD)] = 1.03 (1.37) / Range = 0-4 - Bodily discomfort [mean (SD)] = 1.27 (1.32) / Range = 0-4   PDQ-8 floor effect = 12% / PDQ-8 ceiling effect = 1%  PDQ-8 administration time = 5-12 minutes |
| Zhang (2011) [35] | China | 126 | Age [mean (SD)] = 63.9 y (10.37) / Range = 24-86  Women [%] = 45.2%  UPDRS-Total [mean (SD)] = 44,.7 (21.01) / Range = 6-145  Age at debut of PD [mean (SD)] = 57.90 y (10.87) / Range = 23-78  H&YS [mean (SD)] = 2.11 (0.82) | PDQ-39 SI [mean (SD)] = 25.94 (14.58)  PDQ-39 scores for dimensions:   - Mobility [mean (SD)] = 31.37 (25.50) - ADL [mean (SD)] = 26.62 (26.61) - Emotional wellbeing [mean (SD)] = 27.58 (23.06) - Stigma [mean (SD)] = 29.12 (25.72) - Social support [mean (SD)] = 11.24 (17.46) - Cognition [mean (SD)] = 29.46 (17.69) - Communication [mean (SD)] = 23.15 (20.76) - Bodily discomfort [mean (SD)] = 28.97 / DE = 20.65 |
| Kwon (2013) [36] | South Korea | 102 | Age [mean (SD)] = 65.3 y (9.55)  Women [%] = 49.02%  Years of educaction [mean (SD)] = 8.85 (4.73)  Dosis of levodopa (mg) [mean (SD)] = 397.19 (329.97)  UPDRS- Part 1 [mean (SD)] = 1.82 (0.70)  UPDRS-Part 2 [mean (SD)] = 1.94 (1.8)  UPDRS-Part 3 [mean (SD)] = 5.97 (5.90)  K-MMSE [mean (SD)] = 26.90 (2.71)  K-MADS [mean (SD)] = 10.37 (10.09)  H&YS [mean (SD)] = 1.82 (0.7) / H&YS [%] = Stage 1 = 31.37% / Stage 1.5 = 11.76% / Stage 2 = 30.39% / Stage 2.5 = 16.67% / Stage 3 = 8.82% / Stage 4 = 0.98%  Duration of PD (years) [mean (SD)] = 3.64 (4.01) | PDQ-39 response rate = 99.02%  PDQ-39 SI [mean (SD)] = 23.3 (18.63) / Range = 0-74.9  PDQ-39 scores for dimensions:   - Mobility [mean (SD] = 22.25 (26.5) / Range = 0-97.5 / Floor effect = 27.5% / Ceiling effect = 0.0% - ADL [mean (SD)] = 19.61 (23.92) / Range = 0-87.5 / Floor effect = 30.4% / Ceiling effect = 0.0% - Emotional wellbeing [mean (SD)] = 27.51 (24.11) / Range = 0-87.5 / Floor effect = 17.6% / Ceiling effect = 0.0% - Stimga [mean (SD)] = 18.18 (19.65) / Range = 0-94.0/ Floor effect = 33.3% / Ceiling effect = 0.0% - Social support [mean (SD)] = 14.70 (21.47) / Range = 0-91.7 / Floor effect = 51.9% / Ceiling effect = 0.0% - Cognition [mean (SD)] = 23.27 (22.78) / Range = 0-91.7/ Floor effect = 17.9% / Ceiling effect = 0.0% - Communication [mean (SD)] = 13.05 (18.49) / Range = 0-66.7 / Floor effect = 52.9% / Ceiling effect = 0.0% - Bodily discomfort [mean (SD)] = 26.43 (23.38) / Range = 0-83.3 / Floor effect = 21.6% / Ceiling effect = 0.0% |
| Park (2014) [37] | South Korea (Seoul) | 93 | Age [mean (SD)] = 65.13 y (9.84) / Age ranges [%]: <65 years = 42.9% / 65-74 years = 39.6% / ≥75 years = 17.6%  Women [%] = 58.1%  Years of education [mean (SD)] = 8.3 (5.6)  Work status: Employed = 22.6% / Unemployed = 77.4%  Marital status: Married = 67.7% / Unmarried (single, widowed, divorced) = 32.3%  H&YS [%]: Stage 1 = 4.3% / Stage 1.5 = 21.5% / Stage 2 = 37.6% / Stage 2.5 = 12.9% / Stage 3 = 16.1% / Stage 4 = 7.5%  Duration of PD (years) [mean (SD)] = 3.53 (3.08) | PDQ-39 SI [mean (SD)] = 30.19 (15.4) / PDQ-39 SI [median] = 28.4 / Range = 4.38-81.15 / Floor effect = 1.1% / Ceiling effect = 1.1%  PDQ-39 scores for dimensions:   - Mobility [mean (SD)] = 39.19 (25.34) / Median = 35 / Range = 5-100 / Floor effect = 6.5% / Ceiling effect = 1.1% - ADL [mean (SD)] = 25.27 (23.85) / Median = 12.5 / Range = 0-100 / Floor effect = 1.1% / Ceiling effect = 1.1% - Emotional wellbeing [mean (SD)] = 43.32 (24.13) / Median = 37.5 / Range = 0-100 / Floor effect = 1.1% / Ceiling effect = 1.1% - Stigma [mean (SD)] = 28.83 (17.31) / Median = 25 / Range = 0-75 / Floor effect = 7.5% / Ceiling effect = 2.2% - Social support [mean (SD)] = 22.07 (16.07) / Median = 25 / Range = 0-75 / Floor effect = 8.5% / Ceiling effect = 1.4% - Cognition [mean (SD)] = 43.21 (15.10) / Median = 37.5 / Range = 6.25-93.75 / Floor effect = 4.3% / Ceiling effect = 2.2% - Communication [mean (SD)] = 24.19 (16.76) / Median = 25 / Range = 0-75 / Floor effect = 5.4% / Ceiling effect = 1.1% - Bodily discomfort [mean (SD)] = 20.61 (17.75) / Median = 16.70 / Range = 0-91.67/ Floor effect = 15.1% / Ceiling effect = 1.1% |
| Fereshtehnejad (2014) [38] | Iran (Teheran) | 114 | Age [mean] = 31.3 / Range = 38-91  Women [%] = 21.9%  Educative level [%]: Illiterate = 3.5% / Primary and/or Secondary = 28.3% / High school/diploma = 30.1% / College and/or University = 38.1%  Comorbidities [%, Yes]: Depression = 22.3% / Cardiovascular disease = 17.1% / Hypertension = 16.1% / Diabetes = 13.5% / Ostheoarthritis = 9.0%  UPDRS-Total [mean SD)] = 31.3 (18.1)  UPDRS-Mental [mean (SD)] = 11.3 (7.7)  UPDRS-ADL [mean (SD)] = 14.7 (9.6)  UPDRS-Motor [mean (SD)] = 3.4 (2.8)  UPDRS-Complications [mean (SD)] = 31.3 (18.1)  S&E [mean (SD)] = 81.7 (17.7)  Daily dose of levodopa (mg) [mean (SD)] = 850 (495)  Duration of the administration of levodopa (years) [mean (SD)] = 4.6 (4.5)  H&YS [mean (SD)] = 1.9 (0.9) / H&YS [%, Stages 1+2] = 69.3%  Duration of PD (years) [mean (SD)] = 9.0 (5.4) | **PDQ-39:**  –  **PDQ-8:**  – |
| Krygowska-Wajs (2015) [39] | Poland | 119 | Age [mean (SD)] = 63.0 (10.5)  Women [%] = 39.5%  H&YS [mean (SD)] = 2.6 (0.73)  Duration of PD (years) [mean (SD)] = *.0 (5.4) | **PDQ-39:**  PDQ-39 SI [mean (SD)] = 34.5 (20.6)  **PDQ-8:**  PDQ-8 score [mean (SD)] = 34.6 (20.2) |
| Morley (2015, a) [40] | United Kingdom | 118 | Age [mean (SD)] = 63.48 y (8.66)  Women [%] = 44.07%  Age at diagnosis of PD [mean (SD)] = 57.69 y (9.00)  Duration of PD (years) [mean (SD)] = 5.73 (4.34) | PDQ-39 response rate = 91.4%  PDQ-39 SI [mean (SD)] = 28.67 (16.66) / Range = 2.97-91.93  Floor effect [%]: Mobility = 1.7% / ADL = 0.8% / Emotional wellbeing = 0.8% / Stigma = 0.8% / Social support = 0.8% / Cognition = 0.8% / Communication = 0.8% / Bodily discomfort = 0.8%  Ceiling effect [%]: Mobility = 4.2% / ADL = 1.7% / Emotional wellbeing = 5.9% / Stigma = 22.0% / Social support = 39.8% / Cognition = 1.7% / Communication = 21.2% / Bodily discomfort = 6.8% |
| Morley (2015, b) [41] | United Kingdom | 118 | Age [mean] = 63.48 y  Women [%] = 44.07%  Age at diagnosis of PD [mean] = 57.69 y  Duration of PD (years) [mean] = 5.73 | PDQ-39 response rate = 91.4% |
| Jesus-Ribeiro (2017) [42] | Portugal | 100 | Age [mean (SD)] = 65.8 y (9.1) / Range = 44-82 / Age ranges [%]: ≤64 years = 43% / 65-74 years = 36% / ≥75 years = 21%  Women [%] = 58%  MMSE [mean (SD)] = 24.9 (3.6) / Range = 19-30  Work status [%]: Active = 19% / Non active = 81%  Years of education [%]: <4 years = 45% / 4-6 years = 40% / 7-9 years = 5% / 10-12 years = 2% / >12 years = 8%  H&YS defined severity of PD [%]: Mild = 12% / Moderate = 77% / Advanced = 11% / H&YS [median (IQR)] = 2 (1) / Range = 1-4 | **PDQ-39:**  PDQ-39 scores for dimensions:   - Mobility [mean (SD)] = 56.5 / DE = 27.7 / Range = 0-100 / 95CI = 51.0-62.0 - ADL [mean (SD)] = 47.5 (47.5) / Range = 0-100 / 95CI = 41.4-53.6 - Emotional wellbeing [mean (SD)] = 51.2 (51.2) / Range = 0-100 / 95CI = 46.4-56.0 - Stigma [mean (SD)] = 30.0 (26.4) / Range = 0-100 / 95CI = 24.8-35.2 - Social support [mean (SD)] = 7.1 (4.7) / Range = 0-83.3 / 95CI = 4.0-10.3 - Cognition [mean (SD)] = 39.4 (22.5) / Range = 0-81.3 / 95CI = 35.0 - 43.9 - Communication [mean (SD)] = 28.1 (22.3) / Range = 0-75.0 / 95CI = 23.7-32.5 - Bodily discomfort [mean (SD)] = 48.4 (21.2) / Range = 0-91.7 / 95CI = 44.2-52.6   **PDQL:**  PDQL score [mean (SD)] = 51.7 (18.4) / Range = 8.1-100   - Parkinsonism symptoms [mean (SD)] = 52.1 (20.4) / Range = 8.9-100 / 95CI = 48.1-56.2 - Systemic symptoms [mean (SD)] = 47.5 (19.8) / Range = 3.6-100 / 95CI = 43.6-51.5 - Emotional functioning [mean (SD)] = 52.2 (17.6) / Range = 11.1-100 / 95CI = 48.7-55.7 - Social functioning [mean (SD)] = 54.4 (25.0) / Range = 0-100 / 95CI = 49.5-59.4 |
| Galeoto (2018) [43] | Italy (Rome) | 104 | Age [mean (SD)] = 65.7 y (10.2) / Range = 42-90  Women [%] = 38.5%  MMSE [mean (SD)] = 28.4 (1.8) / Range = 23-30  Education level [%]: Primary School = 7.6% / Middle School = 31.7% / High School = 43.2% / Graduated = 17.3%  Work status [%]: Employed = 27% / Unemployed = 6% / Retired = 67%  Marital status [%]: Married = 74% / Unmarried = 26%  H&YS [%]: Stage 1 = 8.6% / Stage 2 = 31.7% / Stage 3 = 43.2% / Stage 4 = 16.3%  Duration of PD (years) [mean (SD) = 7.4 (5.3) / Range = 0-26 | PDQ-39 SI [mean (SD)] = 52.38 (28.66) / Range = 8-128  PDQ-39 scores for dimensions:   - Mobility [mean] = 15.63 / Median = 10.59 / Range = 0-39 - ADL [mean] = 8.80 / Median = 6.27 / Range = 0-24 - Emotional wellbeing [mean] = 9.37 / Median = 5.36 / Range = 0-22 - Stigma [mean] = 4.43 / Median = 3.68 / Range = 0-14 - Social support [mean] = 1.65 / Median= 2.29 / Range = 0-9 - Cognition [mean] = 4.88 / Median = 3.47 / Range = 0-15 - Communication [mean] = 3.08 / Median = 2.85 / Range = 0-11 - Bodily discomfort [mean] = 3.57 / Median = 2.23 / Range = 0-8 |
| Suratos (2018) [44] | Philippines | 100 | Age [mean (SD)] = 60.7 y (9.33)  Women [%] = 40.0%  Education level [%]: High School = 30% / Elementary level = 11% / Post-graduate = 1% / Illiterate = 2%  Daily dose of levodopa (mg) [mean (SD)] = 502.12 (265.02)  H&YS [mean (SD)] = 2.34 (0.79) / H&YS [%]: Stage 1 = 10% / Stage 1.5 = 12% / Stage 2-3 = 28% / Stage 2.5 = 15% / Stage 4 = 7%  Duration of PD (years) [mean (SD)] = 4.67 (4.38) | PDQ-39 SI [mean (SD)] = 32.97 (17.3)  PDQ-39 scores for dimensions:   - Mobility [mean (SD)] = 46.98 (25.97) - ADL [mean (SD)] = 37.87 (27.29) - Emotional wellbeing [mean (SD)] = 32.13 (23.14) - Stigma [mean (SD)] = 29.31 (26.68) - Social support [mean (SD)] = 21.08 (23.82) - Cognition [mean (SD)] = 28.06 (22.32) - Communication [mean (SD)] = 25.42 (24.60) - Bodily discomfort [mean (SD)] = 42.92 (25.39) |
| Holden (2019) [45] | United States (California) and Canada (Alberta) | 210  (184 with PD) | Age [mean (SD)] = 69.8 y (8.3) / Range = 46-88  Duration of PD (years) = 9.5 (6.4) | **PDQ-39:**  PDQ-39 SI [mean (SD)] = 91.7 (26.8)  No significant floor or ceiling effects observed  **McGill QOL:**  McGill QOL total score [mean (SD)] = 117.8 (28.3)  No significant floor or ceiling effects observed  MIC = 3.9  **PROMIS-29:**  PROMIS-29 total score [mean (SD)] = 74.9 (21.1)  No significant floor or ceiling effects observed  MIC = 10.9  **QOL-AD:**  QOL-AD total score [mean (SD)] = 34.0 (6.3)  No significant floor or ceiling effects observed  MIC = 18.9 |
| Nelson (2020) [46] | South Africa | 576  (Content validity: 160 / Construct validity: 416) | Age [mean (SD, Range)]: Content sample = 50.9 y (10.0, 33-79) / Construct sample = 51.2 (9.4, 38-97)  Women [%]: Content sample = 61.5% / Construct sample = 58.9%  Education level [%,Content sample - Construct sample]: None = 1.4% - 14.8% / Primary School = 10.4% - 24.6% / Secondary School = 66.7% - 57.4% / Tertiary = 21.5% - 3.3%  Home language [%,Content sample - Construct sample]: English = 26.0% - 0.3% / Afrikaans = 24.0% - 1.1% / Sotho = 29.9% - 62.6% / isiWhosa = 5.8% - 14.7% / Setswana = 2.6% - 3.7% / isiZulu = 11.7% - 17.7%  Tobacco consumption (ever) [%, Content sample - Construct sample]: Yes = 28.9% - 35.3% / No = 71.1% - 64.7%  Alcohol consumption (ever) [%, Content sample - Construct sample]: Yes = 34.2% - 54.3% / No = 65.8% - 45.7% | PDQ-39 response rate = 99.04%  PDQ-39 SI [mean (SD)] = 33.1 (21.3) / Range = 0-115 |
| Kim (2020) [47] | Korea | 80 | Age [mean (SD)] = 67.55 y (9.31)  Women [%] = 61.2%  Age at diagnosis of PD [%]: >65 years = 63.7% / <64 years = 36.3%  Daily dose of levodopa (mg) [mean (SD)] = 370.24 (258.25)  Work status [%]: Employed = 15.0% / Unemployed = 85.0%  Marital status (Spouse) [%, Yes] = 63.75  UPDRS-Motor [mean (SD)] = 25.74 (17.10)  MMSE [mean (SD)] = 25.54 (3.16)  MoCA [mean (SD)] = 18.00 (8.22)  Global deterioration status [mean (SD)] = 2.67 (1.17)  CDR [mean (SD)] = 0.93 (0.96)  H&YS [mean (SD)] = 1.56 (1.14)  Duration of PD (years) [mean (SD)] = 1.18 (2.39) | **PDQ-39:**  PDQ-39 SI [mean (SD)] = 33.92 (24.4) / Floor effect = 1.3% / Ceiling effect = 1.3%  PDQ-39 scores for dimensions:   - Mobility [mean (SD)] = 46.53 (30.19) - ADL [mean (SD)] = 39.22 (30.86) - Emotional wellbeing [mean (SD)] = 41.61 (25.77) - Stigma [mean (SD)] = 25.52 (24.96) - Social support [mean (SD)] = 21.25 (22.26) - Cognition [mean (SD)] = 40.73 (24.77) - Communication [mean (SD)] = 23.44 (24.42) - Bodily discomfort [mean (SD)] = 33.02 (25.45)   **PDQ-8:**  PDQ-8 score [mean (SD)]: Nested (with PDQ-39) = 34.18 (22.44) / Independent (without PDQ-39) = 33.79 (23.65)  PDQ-8 scores for items (dimensions) [Nested (with PDQ-39]:   - Mobility [mean (SD)] = 46.25 (36.26) - ADL [mean (SD)] = 32.19 (37.14) - Emotional wellbeing [mean (SD)] = 42.82 (29.55) - Social support [mean (SD)] = 24.06 (29.08) - Cognition [mean (SD)] = 39.06 (32.03) - Communication [mean (SD)] = 23.13 (27.78) - Bodily discomfort [mean (SD)] = 38.44 (30.28) - Stigma [mean (SD)] = 27.50 (28.01)   PDQ-8 scores for items (dimensions) [Independent (without PDQ-39]:   - Mobility [mean (SD)] = 41.56 (34.21) - ADL [mean (SD)] = 35.63 (36.84) - Emotional wellbeing [mean (SD)] = 40.00 (28.57) - Social support [mean (SD)] = 24.38 (32.80) - Cognition [mean (SD)] = 36.88 (33.51) - Communication [mean (SD)] = 23.13 (29.97) - Bodily discomfort [mean (SD)] = 40.94 (30.87) - Stigma [mean (SD)] = 27.81 (30.55) |
| Hanff (2023) [48] | Luxembourg | 736 | Age [mean (SD)] = 67.3 y (10.9) / Age [median (IQR, P25-P75)] = 68.3 (14.5, 60.2-74.7) / Range = 22-92.9  Women [%] = 33.6%  Years of education [mean (SD)] = 12.9 (4.1) / Range = 1-30 / Median (IQR, P25-P75) = 12 (6, 10-16)  Most fluid language [%]: French = 28.8% / Luxembourgian = 42.9% / Other = 12.2%  Marital status [%]: Single = 5.3% / Married = 76.4% / Divorced or widowed = 17.9%  Retired from work [%, Yes] = 72.1%  Dementia [%, Yes] = 100%  Duration of PD (years) [mean (SD)] = 5.2 (5.1) / Duration of PD (years) [median (IQR, P25-P75)] = 3.5 (6.5, 1.2-7.7) / Range = 0-32.3 | PDQ-39 response rate = 93.8%  ODQ-39 SI [mean (SD)] = 73.8 (23.0) / PDQ-39 SI [median (IQR, P25-P75] = 79.7 (34.4, 59.4-93.8) / Range = 1.6-100) |
| Katsarou (2004) [49] | Greece | 228 | Age [mean (SD)] = 59.3 y (8.7) / Range = 37-79  Time in Off (years) [mean (SD)] = 2.2 (0.6) / Range = 1-4  ADL [%]: On = 84.7% / Off = 73.1%  UPDRS-Total On [mean (SD)] = 17.5 (8.6) / Range = 4-40]  UPDRS-Total Off [mean (SD)] = 26.5 (13.1) / Range = 4-65  BDI [mean (SD)] = 8.1 (6.1) / Range = 1-25  H&YS [%]: Stage 1 = 9.65% / Stage 2 = 45.18% / Stage 3 = 26.75% / Stage 4 = 18.42%  Duration of PD (years) [mean (SD)] = 6.7 (4.3) / Range = 1-20 | PDQ-8 response rate = 100%  PDQ-8 score [mean (SD)] = 25.86 (16.98) / PDQ-8 score [median (IQR, P25-P75)] = 21.88 (25, 12.5-37.5) / Range = 0-78.12 |
| Tan (2007) [50] | Singapore | 183 (Whole sample) | Age [mean (SD)] = 61.0 y (9.8)  Women [%] = 31.1%  Ethnicity [%]: China = 86.3% / Malaysia = 5.5% / India = 7.1% / Other = 1.1%  Years of education [%]: ≤6 years = 34.4% / 7-12 years = 42.1% / >12 years = 23.5%  Motor score [mean (SD)] = 21.9 (11.0)  H&YS [mean (SD)] = 2.3 (0.7)  Duration of PD (years) [mean (SD)] = 4.6 (3.8) | PDQ-8 score [mean (SD)] = 27.5 (19.9)  No relevant floor or ceiling effect observed |
|  |  | 104 (English sample) | Age [mean (SD)] = 59.9 y (9.8)  Women [%] = 26.9%  Ethnicity [%]: China = 76.0% / Malaysia = 9.6% / India = 12.5% / Other = 1.9%  Years of education [%]: ≤6 years = 20.2% / 7-12 years = 49.0% / >12 years = 30.8%  Motor score [mean (SD)] = 20.4 (11.1)  H&YS [mean (SD)] = 2.3 (0.7)  Duration of PD (years) [mean (SD)] = 4.6 (3.9) | PDQ-8 score [mean (SD)] = 23.4 (17.2)  No relevant floor or ceiling effects observed |
|  |  | 79 (Chinese sample) | Age [mean (SD)] = 62.5 y (9.7)  Women [%] = 36.7%  Ethnicity [%]: China = 100% / Malaysia = 0.0% / India = 0.0% / Other = 0.0%  Years of education [%]: ≤6 years = 53.2% / 7-12 years = 32.9% / >12 years = 13.9%  Motor score [mean (SD)] = 24.0 (10.5)  H&YS [mean (SD)] = 2.3 (0.7)  Duration of PD (years) [mean (SD)] = 4.7 (3.7) | PDQ-8 score [mean (SD)] = 32.8 (21.9)  No relevant floor or ceiling effects observed |
| Jenkinson (2007) [51] | United States | 185 | Age [mean (SD)] = 68.35 y (9.49) / Range = 33-94  Women [%] = 41.01%  H&YS [mean (SD)] = 2.54 (0.88)  Duration of PD (years) [mean (SD)] = 8.2 (6.72) | PDQ-8 response rate = 96.22%  PDQ-8 score [mean (SD)] = 30.72 (22.25) / PDQ-8 score [median (IQR, P25-P75)] = 25 (34.38, 12.5-46.88) / Range = 0-100 |
|  | Canada | 127 | Age [mean (SD)] = 68.35 y (9.49) / Range = 33-94  Women [%] = 41.01%  H&YS [mean (SD)] = 2.65 (1.03)  Duration of PD (years) [mean (SD)] = 7.43 (7.0) | PDQ-8 response rate = 95.28%  PDQ-8 score [mean (SD)] = 31.4 (20.58) / PDQ-8 score [median (IQR, P25-P75)] = 25 (29.68, 15.63-45.31) / Range = 0-100 |
|  | Italy | 205 | Age [mean (SD)] = 68.35 y (9.49) / Range = 33-94  Women [%] = 41.01%  H&YS [mean (SD)] = 2.45 (1.09)  Duration of PD (years) [mean (SD)] = 7.3 (5.43) | PDQ-8 response rate = 95.12%  PDQ-8 score [mean (SD)] = 39.07 (22.32) / PDQ-8 score [median (IQR, P25-P75)] = 37.5 (31.25, 21.88-53.13) / Range = 0-93.75 |
|  | Spain | 200 | Age [mean (SD)] = 68.35 y (9.49) / Range = 33-94  Women [%] = 41.01%  H&YS [mean (SD)] = 2.47 (1.09)  Duration of PD (years) [mean (SD)] = 9.03 (5.61) | PDQ-8 response rate = 97.00%  PDQ-8 score [mean (SD)] = 26.64 (20.1) / PDQ-8 score [median (IQR, P25-P75)] = 21.88 (25, 12.5-37.5) / Range = 0-84.38 |
|  | Japan | 100 | Age [mean (SD)] = 68.35 y (9.49) / Range = 33-94  Women [%] = 41.01%  H&YS [mean (SD)] = 2.85 (0.95)  Duration of PD (years) [mean (SD)] = 8.44 (6.75) | PDQ-8 response rate = 97.00%  PDQ-8 score [mean (SD)] = 27.16 (17.56) / PDQ-8 score [median (IQR, P25-P75)] = 25 (25, 12.5-37.5) / Range = 0-81.25 |
| Franchignoni (2008) [52] | Italy | 100 (with PDQ-39) | Age [mean (SD)] = 72 y (7)  Women [%] = 59%  IPA-I [mean] = 72 / Range = 58-83  UPDRS-ADL [mean] = 16 / Range = 12-20  UPDRS-Motor [mean] = 23 / Range = 18-28  S&E [mean] = 80 / Range = 70-80  H&YS [mean] = 3  Duration of PD (years) [mean (SD)] = 7 (5) | PDQ-8 score [mean] = 34 / Range = 19-50 |
|  |  | 100 (without PDQ-39) | Age [mean (SD)] = 71 y (8)  Women [%] = 56%  IPA-I [mean] = 73 / Range = 61-84  UPDRS-ADL [mean] = 15 / Range = 12-20  UPDRS-Motor [mean] = 23 / Range = 18-28  S&E [mean] = 80 / Range = 70-80  H&YS [mean] = 3  Duration of PD (years) [mean (SD)] = 7 (5) | PDQ-8 score [mean] = 36 / Range = 25-53 |
|  |  | 200 (all participants) | Age [mean (SD)] = 72 y (7)  Women [%] = 57.5%  IPA-I [mean] = 73 / Range = 61-84  UPDRS-ADL [mean] = 16 / Range = 12-20  UPDRS-Motor [mean] = 23 / Range = 18-28  S&E [mean] = 80 / Range = 70-80  H&YS [mean] = 3  Duration of PD (years) [mean (SD)] = 7 (5) | PDQ-8 score [mean] = 34 / Range = 22-50 |
| Dal Bello-Haas (2009) [53] | Canada | 24 | Age [mean (SD)] = 64.9 y (8.0) / Range = 40-80  Women [%] = 25%  Higher education level [%]: Some elementary = 8.3% / Some secondary = 12.5% / Completed secondary = 8.3% / Some college/university = 16.7% / Completed college/university = 41.7% / Graduate degree = 12.5%  Living arrangement [%]: Lives with others = 91.7% / Lives alone = 8.3%  Work status [%]: Retired = 75% / Working part time = 4.2% / Working full time = 20.8%  MMSE [mean (SD)] = 27.4 (2.5) / Range = 26-30  BDI [mean (SD)] = 9.0 (5.6) / Range = 0-19  Antiparkinsonian medication: L-Dopa = 20.8% / Sinemet = 12.5% / Amantadina = 50% / Mirapex = 29.2% / Requip = 12.5%  Falls (previous 2 weeks) [mean (SD)] = 0.25 (1.0) / Range = 0-5  Near-falls (previous 2 weeks) [mean (SD)] = 0.54 (2.0) / Range = 0-10  H&YS [%]: Stage 1 = 54.2% / Stage 2 = 25.0% / Stage 3 = 20.8%  Duration of PD (years) [mean (SD)] = 4.5 (4.3) / Range = 0-20 | PDQ-8 score [mean (SD)]: First administration = 25.9 (4.2) / Second administration = 24.9 (5.0)  PDQ-8 score [median (Range)]: First administration = 26 (16-32) / Second administration = 25.5 (14-32)  PDQ-8 estimated MIC = 5.43 |
| Alvarado-Bolaños (2015) [54] | Mexico | 585 | Age [mean (SD)] = 62.9 y (12.3)  Women [%] = 45.5%  Medication for PD (all patients received at least one treatment) [%]: Levodopa = 75.7% / Dopamine agonists = 60.3%  UPDRS- Part III [mean (SD)] = 30.5 (18.5)  PDQ-8 [mean (SD)] = 18.2 (13)  Level of studies [%]: University = 23.1% / Non university = 76.9%  Work status [%]: Employed = 28.3% / Unemployed = 71.7%  Comorbidities [%, Yes] = 51.1%  Duration of PD [%]: 0-8 years = 66.5% / >8 years = 33.5%  EQ-VAS [%]: Score 0-20 = 2.2% / Score 21-40 = 3.8% / Score 41-60 = 19.7% / Score 61-80 = 40.9% / Score 81-100 = 33.5%  Dyskinesia [%, Yes] = 30%  Wearing Off [%, Yes] = 33.1%  Freezing [%, Yes] = 40.8%  Postural instability [%, Yes] = 57.9%  Cognitive impairment [%, Yes] = 45.9%  Depression [%, Yes] = 50.6%  H&YS [%]: Stage 1+2 = 59.2% / Stage 3 = 30.6% / Stage 4+5 = 10.2  Duration of PD (years) [mean (SD)] = 7.6 (6.1) | **PDQ-8:**  PDQ-8 score [mean (SD)] = 18.2 (13.0)  **EQ-5D-5L**  EQ-5D-5L Utility value [mean (SD)] = 0.71 (0.20)  **EQ-VAS**  EQ-VAS score [mean (SD)] = 73.8 (18.7) |
| Kahraman (2018) [55] | Turkey | 83 | Age [mean (SD)] = 68.3 y (9.3)  Women [%] = 49.4%  BMI [mean (SD)] = 27.65 (3.92)  Education level [%]: Primary School = 50.6% / Secondary School = 12.0% / High School = 18.1% / University = 19.3%  Marital status [%]: Married = 83.2% / Single = 16.8%  H&YS [%, Stages 1 to 3] = 82.0%  Duration of PD (years) [mean] = 6.5 / Range = 1-29 | PDQ-8 score [mean] = 25 / Range = 0-75  No relevant floor or ceiling effects observed |
| Ramadhan (2022) [56] | Europe (United Kingdom, France, Germany, Netherlands, Portugal and Sweden) | 558 | Age [mean] = 76 y / Range = 42-94  Women [%] = 45.9%  Age at onset [mean] = 60 y / Range = 17-85  Years of education [mean (SD)] = 10.3 (3.79)  MMSE [mean (SD)] = 25.7 (3.40) | **PDQ-8:**  –  **EQ-5D-3L:**  – |
| Stathis (2022) [57] | Greece | 60 | Age [mean (SD)] = 64.52 y (9.39)  Women [%] = 45%  Affected by 1-10 motor symptoms [%, Yes] = 43.3%  Depression [%, Yes] = 95%  H&YS [%, Stages 2+3] = 73.4% | **PDQ-8:**  PDQ-8 score [mean (SD)] = 11.82 (6.04)  PDQ-8 floor effect = 1.7% / PDQ-8 ceiling effect = 1.7%  **PDQoL7:**  PDQoL7 response rate = 100%  PDQoL7 scores [mean (SD)] = 14.58 (5.55)  PDQoL7 Floor effect = 3.3% / Ceiling effect = 1.7% |
| Kawaguchi (2021) [58] | Japan (Fukuoka) | 54 | Distribution by age (Males) [%]: 30-39 years = 0.0% / 40-49 years = 3.7% / 50-59 years = 25.9% / 60-69 years = 55.6% / 70-79 years = 14.8%  Distribution by age (Females) [%]: 30-39 years = 3.7% / 40-49 years = 3.7% / 50-59 years = 7.4% / 60-69 years = 70.4% / 70-79 years = 14.8%  Distribution by age (Overall) [%]: 30-39 years = 1.9% / 40-49 years = 3.7% / 50-59 years = 16.7% / 60-69 years = 63.0% / 70-79 years = 14.8%  Women [%] = 50%  H&YS (On) [%]: Stage 1 = 0.0% / Stage 2 = 16.7% / Stage 3 = 72.2% / Stage 4 = 9.3% / Stage 5 = 1.9%  H&YS (Off) [%]: Stage 1 = 0.0% / Stage 2 = 13.0% / Stage 3 = 68.5% / Stage 4 = 16.7% / Stage 5 = 1.9% | – |
| De Boer (1996) [59] | Netherlands (Amsterdam) | 384 | Age [mean (SD)] = 67.1 y (10.8) / Range = 29-100  Women [%] = 47.4%  Marital status [%]: Married = 70.3% / Widowed-Unmarried-Divorced = 29.7%  Work status: Unfit to work = 16.4% / Pension = 56.3% / Other = 27.3%  S&E [mean (SD)] = 7.87 (6.34) / Group 1 (Independent - Activities a bit slower) = 44.5% / Group 2 (Slightly dependent - Considerably slower) = 33.1 / Group 3 (Dependent - Needing help or care) = 21.4%  Medication: Levodopa = 87.0% / Bromocriptine = 14.1% / Selegiline = 49.2% / Amantadine = 18.5% / Benzodiazepinas = 5.5% / Antidepressives = 8.9%  Duration of PD (years) [mean] = 6.34 | PDQL response rate = 85.07%  PDQL score [mean (SD)] = 122.9 (26.7)  PDQL scores for dimensions:   - Parkinsonian symptoms [mean (SD)] = 43.8 (10.7) - Systemic symptoms [mean (SD] = 22.9 (6.2) - Emotional functioning [mean (SD] = 31.5 (7.5) - Social functioning [mean (SD] = 24.2 (6.9) |
| Serrano-Dueñas (2004) [60] | Ecuador (Quito) | 137 | Age [mean (SD)] = 69.4 y (10.1)  Women [%] = 32.2%  Duration of treatment (years) [mean (SD)] = 4.7 (2.53)  Daily dose of levodopa (mg, 100% patient use levodopa) [mean (SD)] = 780.36 (251.17)  Bromocriptine (43.07%) [mean (SD)] = 10.78 (4.9)  SPMSQ [mean (SD)] = 1.45 (1.65) / Range = 0-6  Depression [%, Yes] = 24.8%  Anxiety [%, Yes]= 26.3%  S&E [mean (SD)] = 70.40 (19.38) / Range = 10-100 / 95CI = 67.12 - 73.67  UPDRS-Mental [mean (SD)] = 5.42 (2.47) / Range = 2-13 / 95CI = 5.00 - 5.84  UPDRS-ADL [mean (SD)] = 19.73 (10.00) / Range = 4-47 / 95CI = 18.04 - 21.442  UPDRS-Motor [mean (SD)] = 40.40 (14.925) / Range = 15-93 / 95CI = 27.87 - 42.91  UPDRS-Total [mean (SD)] = 65.5 (25.32 / Range = 24-153 / 95CI = 61.27 - 69.83  HADS-A [mean (SD)] = 8.73 (3.92) / Range = 0-19 / 95CI = 8.06 - 9.40  HADS-D [mean (SD)] = 10.38 (4.32) / Range = 1-18 / 95CI = 9.65 - 11.11  PDQ-39 SI [mean (SD)] = 41.48 (15.51) / Range = 8.85-81.77 / 95CI = 38.86 - 44.10  H&YS [mean (SD)] = 2.28 (0.77) / H&YS [%]: Stage 1 = 8.03% / Stage 1.5 = 19.71% / Stage 2 = 20.44% / Stage 2.5 = 26.28% / Stage 3 = 18.25% / Stage 4 = 5.84% / Stage 5 = 1.46%  Duration of PD (years) [mean (SD)] = 5.87 (2.58) | PDQL score [mean (SD)] = 43.01 (9.76) / Range = 21-63  PDQL scores for dimensions:   - Parkinsonism symptoms [mean (SD)] = 21.82 (5.02) / Range = 12-34 / 95CI = 20.97-22.67 - Systemic symptoms [mean (SD)] = 17.78 (6.00) / Range = 7-33 / 95CI = 16.76-18.80 - Emotional functioning [mean (SD)] = 27.39 (6.47) / Range = 16-40 / 95CI = 26.30-28.48 - Social functioning [mean (SD)] = 110.01 (22.85) / Range = 61-160 / 95CI = 106.15-113.87 |
| Campos (2011) [61] | Brazil | 58 | Age [mean] = 64.83 y / Range = 37-88  Women [%] = 30.8%  Ethnicity [%. Caucasic] = 86.5%  Marital status [%, Married] = 63.5%  Duration of PD (years) [mean] = 11.81 / Duration of PD (years) [%]: 0-5 years = 45.1% / 6-10 years = 25.0% / >10 years = 25.0% / Unknown = 1.9%  H&YS [%]: Stages 1+1.5+2 = 15.3% / Stage 2 = 44.2% / Stages 3+4+5 = 40.4% | – |
| Dereli (2015) [62] | Turkey | 89 | Age [mean (SD)] = 64.83 y (11.81) / Range = 40-82  Women [%] = 43.8%  Education level [%]: Elementary School = 51.7% / Secondary School = 15.7% / High School = 20.2% / Academy Graduates = 12.4%  UPDRS-Part 1 [mean (SD)] = 2.83 (2.16)  UPDRS-Part 2 [mean (SD)] = 14.10 (6.73)  UPDRS-Part 3 [mean (SD)] = 17.37 (6.8)  UPDRS-Total [mean (SD)] = 34.3 (13.73)  H&YS [%]: Stage 1 = 12.4% / Stage 2 = 28.1% / Stage 51.7% / Stage 7.9%  Duration of PD (years) [Range] = 2-18 | PDQL score [mean (SD)] = 125.28 (25.47)  PDQL scores for dimensions:   - Parkinsonism symptoms [mean (SD)] = 47.72 (9.51) - Systemic symptoms [mean (SD)] = 23.85 (5.14) - Emotional functioning [mean (SD)] = 22.92 (5.99) - Social functioning [mean (SD)] = 30.80 (6.29) |
| Welsh (2003) [63] | United States and Canada | 233 | Age [mean (SD)] = 65.4 y (10.3)  Women [%] = 37.8%  Ethnicity [%, Caucasic] = 97.8%  Years since diagnosis of PD [mean (SD)] = 7.2 (5.3)  Years of education [mean (SD)] = 14.7 (3.3)  Marital status [%, Married] = 65.4%  Work status [%, Retired or unable to work] = 79.8%  UPDRS-Total [mean (SD)] = 35.1 (17.8)  UPDRS-Part 1 [mean (SD)] = 1.8 (1.7)  UPDRS-Part 2 (ADL) [mean (SD)] = 11.3 (6.5)  UPDRS-Part 3 (Motor) [mean (SD)] = 22.2 (12.6)  Medication [%, Sinemet] = 65.5%  Motor fluctuations [%, Yes] = 58.8%  H&YS [%]: Stage 1 = 5.6% / Stage 1.5 = 8.6% / Stage 2 = 44.8% / Stage 2.5 = 16.8% / Stage 3 = 20.3% / Stage 3.9% / Stage 5 = 0.0% | PDQUALIF response rate = 95.28%  PDQUALIF score [mean (SD)] = 41.3 (13.7) / Floor effect = 0.0% / Ceiling effect = 0.0% / Administration time = 10-15 minutes  PDQUALIF scores for dimensions:   - Social / Role life [mean (SD)] = 47.7 (20.4) / Floor effect = 0.9% / Ceiling effect = 0.4 % - Self-image / Sexuality [mean (SD)] = 42.3 (21.2) / Floor effect = 0.4% / Ceiling effect = 0.0 % - Sleep [mean (SD)] = 41.2 (26.2) / Floor effect = 8.7% / Ceiling effect = 3.1 % - Outlook [mean (SD)] = 58.2 (16.5) / Floor effect = XXX% / Ceiling effect = XXX % - Physical function [mean (SD)] = 36.5 (17.9) / Floor effect = 3.0% / Ceiling effect = 0.0 % - Independence [mean (SD)] = 9.4 (22.3) / Floor effect = 78.9% / Ceiling effect = 3.0 % - Urinary function [mean (SD)] = 52.8 (23.7) / Floor effect = 3.0% / Ceiling effect = 2.6 % |
| Calne (1996) [64] | Canada | 167 | – | – |
| Schulzer (2003) [65] | Canada | 116 | – | – |
| Aggarwal (2020) [66] | India | 295 | Age [mean (SD)] = 57.0 y (10.7) / Range = 23-85  Women [%] = 27.0%  Family status [%]: Family joint = 52% / Nuclear = 48%  Socioeconomic status [%]: Upper = 15.4% / Upper-Middle = 33.0% / Lower-Middle = 38.7% / Upper-Lower = 11.2% / Lower = 1.9%  H&YS [%]: Stage 1 = 13.9% / Stage 1.5 = 18.0% / Stage 2 = 19.0% / Stage 2.4 = 13.2% / Stage 3 = 22.4% / Stage 4 = 10.5% / Stage 5 = 5.0%  Duration of PD (years) [mean (SD)] = 7.2 (5.4) / Range = 0.3-32 | QLPD response rate = 98.33%  QLPD total score [mean (SD)] = 35.3 (16.6) / Range = 1-83  QLPD scores for dimensions:   - ADL [mean (SD)] = 39.4 (19.7) / Range = 0-100 - Mobility [mean (SD)] = 31.1 (22.8) / Range = 0-100 - Pyschological [mean (SD)] = 30.3 (25.6) / Range = 0-100 - Fear [mean (SD)] = 39.3 (26.8) / Range = 0-100 - Social [mean (SD)] = 30.4 (25.3) / Range = 0-100 - Family [mean (SD)] = 36.7 (29.5) / Range = 0-100 - Treatment [mean (SD)] = 54.6 (24.1) / Range = 0-100 - Finance [mean (SD)] = 33.0 (31.8) / Range = 0-100 - Non-motor symptoms [mean (SD)] = 32.5 (17.8) / Range = 0-86   No floor or ceiling effect observed. |
| Kuehler (2003) [67] | Germany | 30 (PD = 12) | – | **QLSM-DBS:**  QLSM-DBS response rate = 89.3%  QLSM-DBS total score [mean (SD)] = 21.5 (10.7)  QLSM-DBS scores for items [mean (SD)]:   - Reliability of the neurostimulator = 8.6 (6.0) - Inconspicuosiness of the neurostimulator (casing, cabe, scars) = 3.0 (4.5) - Independent handling / manipulation of the neurostimulator = 3.8 (6.4) - Doctoral care (quality, availability) = 3.7 (7.4) - Absence of bodily symptoms / side effects of the neurostimulation = 2.4 (4.9)   Floor effect = 0.0% / Ceiling effect = 0.0%  **QLSM-MD:**  QLSM-MD response rate = 99.61%  QLSM- MD total score [mean (SD)] = 37.1 (51.5)  QLSM-MD score for items [mean (SD)]:   - Controlability / Fluidity of movement = 0.2 (5.4) - Absence of dizziness / Steadiness when standing and walking = 1.4 (7.0) - Hand dexterity throughout the day (e.g. when eating and writing) = 5.2 (6.2) - Articulation / Fluency of speech = 2.8 (8.1) - Ability to swallow = 7.1 (7.4) - Absence of false bodily sensations = 0.4 (6.6) - Bladder / Intestinal function = 3.3 (6.6) - Sexual excitability = 2.2 (5.2) - Unidsturbed sleep = 4.4 (7.6) - Memory / Clear thinking = 5.4 (5.9) - Independence from help (e.g. when dressing and getting washed) = 3.6 (6.4) - Inconspicuosness of illness = 1.2 (5.2)   Floor effect = 0.0% / Ceiling effect = 0.0% |
| Krygowska-Wajs (2015) [68] | Poland | 119 | Age [mean (SD)] = 63.0 y (10.5)  Women [%] = 39.5%  H&YS [mean (SD)] = 2.6 (0.73)  Duration of PD (years) [mean (SD)] = 9.0 (5.4) | **QLSM-DBS:**  QLSM-DBS total score [mean (SD)] = 4.7 (3.6)  **QLSM-MD:**  QLSM-MD total score [mean (SD)] = 0.06 (4.2) |
| Bose (2018) [69] | India | 120 | – | – |
| Diniz (2018) [70] | Brazil | 140 | Age [mean (SD)] = 67.0 y (9.0)  Women [%] = 40.71%  H&YS [%]: Stage 1 = 7.14% / Stage 2 = 33.57% / Stage 3 = 39.29% / Stage 4 = 16.43% / Stage 5 = 3.57% | QOLSQ total score [mean (SD)] = 44.24 (19.76) / QOLSQ total score [median (IQR)] = 42.41 (29.38) / Range = 5.26-96.49  QOLSQ scores for domains:   - Domain 1 [mean (SD)] = 46,61 (37,27) / Median (IQR) = 33,33 (75,00) / Range = 0-100 - Domain 2 [mean (SD)] = 49,05 (25,12) / Median (IQR) = 53,33 (40,00) / Range = 0-100 - Domain 3 [mean (SD)] = 48,39 (21,06) / Median (IQR) = 50,00 (25,00) / Range = 0-100 - Domain 3 [mean (SD)] = 35,91 (20,39) / Median (IQR) = 33,33 (27,78) / Range = 0-100 |
| García-Gordillo (2013) [71] | Spain | 133 | Age [mean (SD)] = 64.3 y (9.74) / Age [median (IQR)] = 65 (13) / Range = 34-86  Women [%] = 28.6%  Level of studies [%]: Primary studies = 48.9% / Secondary studies = 23.3% / University studies = 27.8%  Work status [%]: Self-employee = 2.3% / Government employee = 3.8% / Employee = 3.0% / Housewife = 8.3% / Retired = 82.7%  Household size [mean (SD)] = 2.42 (1.13) / Household size [median (IQR)] = 2.00 (1.00) / Range = 1-6  Monthly earnings (€) [mean (SD)] = 2,087.89 (1,369.13) / Monthly earnings (€) [mean (SD)= 1,700.00 (1,492.00) / Range = 400-5,000  Other medical conditions (N) [mean (SD)] = 0.54 (0.71) / Other medical conditions (N) [median (IQR)] = 0.00 (1.00) / Range = 0-3  Current medication (N) [mean (SD)] = 2.44 (1.38) / Current medication (N) [median (IQR)] = 3.00 (2.00) / Range = 0-6  PDQ-8 score [mean (SD)] = 26.69 (18.24) / PDQ-8 score [median (IQR)] = 21.87 (26.56) / Range = 0-87  EQ-VAS scores [mean (SD)] = 57.63 (19.67) / EQ-VAS scores [median (IQR)] = 60.00 (22.50) / Range = 10-100  EQ-5D-5L score [mean (SD)] = 0.59 (0.26) / EQ-5D-5L score [median (IQR)]= 0.64 (0.28) / Range = -0.25-1.00  H&YS [%]: Stage 1+2 = 36.8% / Stage 3+4 = 63.2%  Duration of disease (years) [mean (SD)] = 7.7 (6.44) / Duration of disease (years) [median (IQR)] = 6 (10) / Range = 0.5-32 | **15D:**  15D Utility [mean (SD)] = 0.74 (0.16) / 15D Utility [median (IQR] = 0.79 (0.24) / Range = 0.31-1.00  Mobility [%]: Level 1 = 46.6% / Level 2 = 32.3% / Level 3 = 11.3% / Level 4 = 9.8% / Level 5 = 0.0%  Visio [%]: Level 1 = 56.6% / Level 2 = 23.3% / Level 3 = 15.0% / Level 4 = 5.3% / Level 5 = 0.0%  Earing [%]: Level 1 = 70.7% / Level 2 = 18.0% / Level 3 = 9.0% / Level 4 = 2.3% / Level 5 = 0.0%  Breathing [%]: Level 1 = 56.4% / Level 2 = 24.8% / Level 3 = 12.8% / Level 4 = 4.5% / Level 5 = 1.5%  Sleeping [%]: Level 1 = 24.4% / Level 2 = 38.3% / Level 3 = 15.8% / Level 4 = 18.8% / Level 5 = 2.3%  Eating [%]: Level 1 = 73.7% / Level 2 = 18.0% / Level 3 = 7.5% / Level 4 = 0.8% / Level 5 = 0.0%  Habla: Level 1 = 33.1% / Level 2 = 45.9% / Level 3 = 14.3% / Level 4 = 6.0% / Level 5 = 0.8%  Elimination [%]: Level 1 = 32.3% / Level 2 = 43.6% / Level 3 = 20.3% / Level 4 = 3.0% / Level 5 = 0.8%  Usual activities [%]: Level 1 = 18.8% / Level 2 = 51.9% / Level 3 = 14.3% / Level 4 = 8.3% / Level 5 = 6.8%  Mental function [%]: Level 1 = 36.1% / Level 2 = 42.1% / Level 3 = 19.5% / Level 4 = 1.5% / Level 5 = 0.8%  Discomfort and symptoms [%]: Level 1 = 46.6% / Level 2 = 30.8% / Level 3 = 15.0% / Level 4 = 5.3% / Level 5 = 2.3%  Depression [%]: Level 1 = 36.8% / Level 2 = 33.1% / Level 3 = 25.6% / Level 4 = 4.5% / Level 5 = 0.0%  Distress [%]: Level 1 = 25.6% / Level 2 = 45.1% / Level 3 = 23.3% / Level 4 = 4.5% / Level 5 = 1.5%  Vitality [%]: Level 1 = 25.6% / Level 2 = 42.9% / Level 3 = 18.8% / Level 4 = 8.3% / Level 5 = 4.5%  Sexual activity [%]: Level 1 = 24.8% / Level 2 = 27.1% / Level 3 = 29.3% / Level 4 = 6.0% / Level 5 = 12.8%  **EQ-5D-5L:**  EQ-5D-5L Utility value [mean (SD)] = 0.59 (0.26) / EQ-5D-5L Utility value [median (IQR)] = 0.64 (0.28) / Range = (-0.25) – 1.00  Mobility: Level 1 = 24.1% / Level 2 = 34.6% / Level 3 = 28.6% / Level 4 = 12.8% / Level 5 = 0.0%  Self-care: Level 1 = 39.8% / Level 2 = 33.8% / Level 3 = 13.5% / Level 4 = 10.5% / Level 5 = 2.3%  Usual activities: Level 1 = 24.1% / Level 2 = 36.1% / Level 3 = 25.6% / Level 4 = 11.3% / Level 5 = 3.0%  Pain – Discomfort: Level 1 = 24.1% / Level 2 = 30.1% / Level 3 = 33.1% / Level 4 = 7.5% / Level 5 = 4.5%  Anxiety – Depression: Level 1 = 33.8% / Level 2 = 35.3% / Level 3 = 27.8% / Level 4 = 0.0% / Level 5 = 3.0%  **SF-6D:**  SF-6D response rate = 84.71%  SF-6D Utility value [mean (SD)] = 0.53 (0.29) / SF-6D Utility value [median (IQR)] = 0.60 (0.39) / Range = (-0.23)-0.98  SF-6D responses by level:   - Physical function [%]: Level 1 = 3.0% / Level 2 = 15.8% / Level 3 = 26.3% / Level 4 = 3.0% / Level 5 = 36.8% / Level 6 = 15.0% - Role limitation [%]: Level 1 = 45.9% / Level 2 = 9.0% / Level 3 = 9.8% / Level 4 = 35.3% / Level 5 = 0.0% / Level 6 = 0.0% - Social function [%]: Level 1 = 29.3% / Level 2 = 20.3% / Level 3 = 33.8% / Level 4 = 9.0% / Level 5 = 7.5% / Level 6 = 0.0% - Pain: Level 1 = 21.1% / Level 2 = 13.5% / Level 3 = 30.1% / Level 4 = 15.8% / Level 5 = 12.8% / Level 6 = 6.8% - Mental health [%]: Level 1 = 12.0% / Level 2 = 17.3% / Level 3 = 45.9% / Level 4 = 23.3% / Level 5 = 1.5% / Level 6 = 0.0% - Vitality [%]: Level 1 = 5.3% / Level 2 = 24.1% / Level 3 = 45.9% / Level 4 = 17.3% / Level 5 = 7.5% / Level 6 = 0.0%   Floor effect = 0.0% / Ceiling effect = 0.0% |
| Del Pozo-Cruz (2018) [72] | Spain | 229 | Age [mean (SD)] = 66 y (9.32) / Age [median (IQR)] = 66 y (13) / Range = 34-86 y  Women [%] = 33.3%  Level of studies [%]: University = 22.1% / No university = 75.8%  Work status [%]: Employed = 5.6% / Retired = 93.5%  Monthly earnings [%]: 400-1,800€ = 22.9% / >1,800€ = 14.7%  Other medical conditions [%, Yes] = 44.6%  Current medication [%, Yes] = 92.6%  PDQ-8 score [mean (SD)] = 28.9 (19.23) / PDQ-8 score [median (IQR)] = 25.0 (28.12) / Range = 0-87.5  H&YS [%]: Stage 1+2 = 64.6% / Stage 3+4 = 35.4%  Duration of disease (years) [mean (SD)] = 8.2 (7.25) / Duration of disease (years) [median (IQR)] = 6 (8) / Range = 0-41 | **15D:**  15D Utility [mean (SD)] = 0.73 (0.15) / 15D Utility [median (IQR)] = 0.77 (0.24) / Range = 0.31-1.00  Mobility [%]: Level 1 = 42.0% / Level 2 = 30.7% / Level 3 = 14.7% / Level 4 = 12.1% / Level 5 = 0.4%  Vision [%]: Level 1 = 53.2% / Level 2 = 25.5% / Level 3 = 13.9% / Level 4 = 7.4% / Level 5 = 0.0%  Earing [%]: Level 1 = 71.9% / Level 2 = 17.3% / Level 3 = 9.1% / Level 4 = 1.7% / Level 5 = 0.0%  Breathing [%]: Level 1 = 53.2% / Level 2 = 23.8% / Level 3 = 13.4% / Level 4 = 6.5% / Level 5 = 3.0%  Sleeping [%]: Level 1 = 25.5% / Level 2 = 36.4% / Level 3 = 18.2% / Level 4 = 17.3% / Level 5 = 2.6%  Eating [%]: Level 1 = 69.7% / Level 2 = 20.3% / Level 3 = 8.7% / Level 4 = 1.3% / Level 5 = 0.0%  Habla: Level 1 = 32.9% / Level 2 = 44.6% / Level 3 = 13.0% / Level 4 = 9.1% / Level 5 = 0.4%  Elimination [%]: Level 1 = 29.4% / Level 2 = 42.9% / Level 3 = 22.9% / Level 4 = 3.9% / Level 5 = 0.9%  Usual activities [%]: Level 1 = 18.2% / Level 2 = 46.3% / Level 3 = 16.5% / Level 4 = 12.5% / Level 5 = 6.5%  Mental function [%]: Level 1 = 38.1% / Level 2 = 42.0% / Level 3 = 16.9% / Level 4 = 2.2% / Level 5 = 0.9%  Discomfort and symptoms [%]: Level 1 = 39.4% / Level 2 = 33.3% / Level 3 = 18.6% / Level 4 = 5.2% / Level 5 = 3.5%  Depression [%]: Level 1 = 33.3% / Level 2 = 35.5% / Level 3 = 23.4% / Level 4 = 6.1% / Level 5 = 1.7%  Distress [%%]: Level 1 = 27.3% / Level 2 = 41.1% / Level 3 = 22.5% / Level 4 = 6.5% / Level 5 = 2.6%  Vitality [%]: Level 1 = 24.2% / Level 2 = 40.3% / Level 3 = 24.7% / Level 4 = 7.8% / Level 5 = 3.0%  Sexual activity [%]: Level 1 = 22.5% / Level 2 = 28.6% / Level 3 = 26.0% / Level 4 = 9.1% / Level 5 = 13.9%  **SF-6D:**  SF-6D Utility [mean (SD)] = 0.51 (0.28) / SF-6D Utility [median (IQR)] = 0.54 (0.392) / Range = (-0.22)-1.00  SF-6D responses by level:   - Physical functioning [%]: Level 1 = 2.6% / Level 2 = 15.2% / Level 3 = 23.4% / Level 4 = 4.3% / Level 5 = 37.2% / Level 6 = 17.3% - Role limitations [%]: Level 1 = 44.2% / Level 2 = 11.7% / Level 3 = 9.1% / Level 4 = 35.1% / Level 5 = 0.0% / Level 6 = 0.0% - Social functioning [%]: Level 1 = 25.5% / Level 2 = 22.1% / Level 3 = 35.9% / Level 4 = 10.4% / Level 5 = 6.1% / Level 6 = 0.0% - Pain [%]: Level 1 = 18.2% / Level 2 = 13.4% / Level 3 = 32.0% / Level 4 = 16.9% / Level 5 = 12.6% / Level 6 = 6.9% - Mental health [%]: Level 1 = 10.0% / Level 2 = 22.9% / Level 3 = 44.6% / Level 4 = 19.9% / Level 5 = 2.6% / Level 6 = 0.0% - Vitality [%]: Level 1 = 6.1% / Level 2 = 21.6% / Level 3 = 45.9% / Level 4 = 17.3% / Level 5 = 9.1% / Level 6 = 0.0%   Floor effect = 0.0% / Ceiling effect = 0.0% |
| Luo (2009) [73] | Singapore (all participants) | 206 | Age [mean (SD)] = 62.1 (9.0)  Women [%] = 20.9%  Ethnicity: China = 86.9% / Malasia = 6.3% / India or other = 7.8%  Level of studies: None or Primary education = 23.8% / O-Level = 42.7% / A-Level or Diploma = 15.1% / Degree = 18.5%  Work status: Employed = 28.2% / Housekeeper or Unemployed = 12.0% / Retired due to PD = 18.0% / Retired due to other reasons = 41.8%  Housing type: 1-2 or 3-4 room HDB = 48.6% / 5 room or Executive HDB = 34.0% / Private property = 17.5%  Caregiver [%, Yes] = 93.2%  S&E [mean (SD)] = 87.6 (8.9)  UPDRS-Motor [mean (SD)] = 20.9 (8.6)  MMSE [mean (SD)] = 26.5 (1.8)  PDQ-8 SI [mean (SD)] = 24.1 (18.8)  Self-reported problems: Mobility = 40.3% / Self-care = 25.7% / ADL = 37.9% / Pain or discomfort = 52.9% / Anxiety or depression = 44.7%  H&YS [mean (SD)] = 2.1 (0.5) / H&YS [%]: Stage 1+1.5 = 8.6% / Stage 2+2.5 = 80.1% / Stage 3 = 11.7% / Stage 4 = 11.7%  Duration of disease (years) [mean (SD)] = 5.2 (5.3) | **EQ-5D-3L**  EQ-5D-3L Response rate = 99.04%  EQ-5D-3L Utility [mean (SD)] = 0.73 (0.26)  **EQ-VAS**  EQ-VAS Response rate = 99.04%  EQ-VAS score [mean (SD)] = 71.6 (15.9) |
|  | Singapore (English speakers) | 135 | Age [mean (SD)] = 61.7 (9.4)  Women [%] = 19.3%  Ethnicity: China = 80.0% / Malasia = 9.6% / India or other = 10.4%  Level of studies: None or Primary education = 12.6% / O-Level = 48.2% / A-Level or Diploma = 15.6% / Degree = 23.7%  Work status: Employed = 31.9% / Housekeeper or Unemployed = 11.0% / Retired due to PD = 17.8% / Retired due to other reasons = 39.3%  Housing type: 1-2 or 3-4 room HDB = 41.5% / 5 room or Executive HDB = 37.0% / Private property = 21.5%  Caregiver [%, Yes] = 94.1%  S&E [mean (SD)] = 87.7 (9.2)  UPDRS-Motor [mean (SD)] = 20.9 (8.8)  MMSE [mean (SD)] = 26.4 (1.9)  PDQ-8 SI [mean (SD)] = 23.0 (18.7)  Self-reported problems: Mobility = 41.5% / Self-care = 28.9% / ADL = 38.5% / Pain or discomfort = 57.0% / Anxiety or depression = 46.7%  H&YS [mean (SD)] = 2.2 (0.5) / H&YS [%]: Stage 1+1.5 = 7.4% / Stage 2+2.5 = 80.7% / Stage 3 = 11.9% / Stage 4 = 11.9%  Duration of disease (years) [mean (SD)] = 5.5 (5.8) | **EQ-5D-3L**  EQ-5D-3L Utility [mean (SD)] = 0.72 (0.26)  **EQ-VAS**  EQ-VAS score [mean (SD)] = 71.1 (16.5) |
|  | Singapore (Chinese speakers) | 71 | Age [mean (SD)] = 62.8 (8.4)  Women [%] = 23.9%  Ethnicity: China = 100%  Level of studies: None or Primary education = 45.0% / O-Level = 32.4% / A-Level / Diploma = 14.1% / Degree = 8.5%  Work status: Employed = 21.1% / Housekeeper or Unemployed = 14.1% / Retired due to PD = 18.3% / Retired due to other reasons = 46.5%  Housing type: 1-2 or 3-4 room HDB = 62.0% / 5 room or Executive HDB = 28.2% / Private property = 9.9%  Caregiver [%, Yes] = 91.5%  S&E [mean (SD)] = 87.3 (8.2)  UPDRS-Motor [mean (SD)] = 21.0 (8.1)  MMSE [mean (SD)] = 26.5 (1.7)  PDQ-8 SI [mean (SD)] = 26.1 (19.0)  Self-reported problems: Mobility = 38.0% / Self-care = 19.7% / ADL = 36.6% / Pain or discomfort = 45.1% / Anxiety or depression = 40.9%  H&YS [mean (SD)] = 2.1 (0.6) / H&YS [%]: Stage 1+1.5 = 8.3% / Stage 2+2.5 = 80.1% / Stage 3 = 11.7% / Stage 4 = 11.7%  Duration of disease (years) [mean (SD)] = 4.8 (4.4) | **EQ-5D-3L**  EQ-5D-3L Utility [mean (SD)] = 0.74 (0.28)  **EQ-VAS**  EQ-VAS score [men (SD)] = 72.5 (14.6) |
| Garcia-Gordillo (2015) [74] | Spain | 133 | Age [mean (SD)] = 64.3 y (9.74) / Age [median (IQR)] = 65 y (13) / Range = 34-86  Women [%] = 28.6%  Level of studies [%]: Primary studies = 48.9% / Secondary studies = 23.3% / University studies = 27.8%  Work status: Self-employee = 2.3% / Government employee = 3.8% / Employee = 3.0% / Housewife = 8.3% / Retired = 82.7%  Family size (number) [mean (SD)] = 2.42 (1.13) / Family size (number) [median (IQR)] = 2.00 (1.00) / Range = 1-6  Monthly earning (€) [mean (SD)] = 2,087.89 (1,369.13) / Monthly earning (€) [median (IQR)] 1,700.00 (1,492.00) / Range = 400-5,000  Comorbidities (number) [mean (SD)] = 0.54 (0.71) / Comorbidities (number) [median (IQR)] = 0.00 (1.00) / Range = 0-3  PDQ-8 [median (IQR)] = 21.87 (26.56) / Range = 0-87  EQ-VAS [mean (SD)] = 57.63 (19.67) / EQ-VAS [median (IQR)] = 60.00 (22.50) / Range = 10-100  SF-6D [mean (SD)] = 0.53 (0.29) / SF-6D [median (IQR)] = 0.60 (0.39) / Range = -0.23 - 0.98  H&YS [%]: Stage 1+2 = 36.8% / Stage 3+4 = 63.2%  Duration of PD (years) [mean (SD)] = 7.7 (6.44) / Duration of PD (years) [median (IQR)] = 6 (1) / Range = 0.5-32 | EQ-5D-3L Response rate = 84.71%  EQ-5D-3L Utility [mean (SD)] = 0.64 (0.31) / EQ-5D-3L Utility [median (IQR)] = 0.72 (0.40) / Range = (-0.65)-1.00  Mobility: Level 1 = 39.1% / Level 2 = 60.2% / Level 3 = 0.8%  Self-care: Level 1 = 55.6% / Level 2 = 39.8% / Level 3 = 4.5%  Usual activities: Level 1 = 40.6% / Level 2 = 53.4% / Level 3 = 6.0%  Pain - DIscomfort: Level 1 = 30.1% / Level 2 = 61.7% / Level 3 = 8.3%  Anxiety - Depression: Level 1 = 45.9% / Level 2 = 52.6% / Level 3 = 1.5% |
| Nowinski (2010) [75] | United States | 121 | Age [mean] = 66 y  Women [%] = 38%  Race / Ethnicity [%] = 95%  H&YS [%]: Stage 1 = 16% / Stage 2 = 61% / Stage 3 = 18% / Stage 4 = 5% | – |
| Nowinski (2016) [76] | United States | 120 | Age [mean (SD)] = 65.51 y (9.28) / Range = 42-88  Women [%] = 38.3%  Race / Ethnicity [%]: Caucasian = 95.0% / African American = 3.3% / Native American = 0.8% / Asiatic = 0.8% / Spanish-Hispanic-Latino origin = 3.3%  Family history of PD [Yes, %] = 29.0%  Most affected side [%]: Rigth = 55.0% / Left = 45.0%  Antiparkinsonian medications: L-Dopa = 19.1% / DA agonists = 9.6% / L-Dopa + iCOMT = 11.3% / L-Dopa + DA agonists = 31.3% / L-Dopa + DA agonists + iCOMT = 3.5% / Other = 7.0% / Other + L-Dopa = 7.0% / Other + DA agonists = 2.6% / Other + L-Dopa + iCOMT = 1.7% / Other + L-Dopa + DA agonists = 7.0%  Motor fluctuations activity limitations [%]: Not at all = 42.5% / A little bit = 33.3% / Somewhat = 21.7% / Very much = 2.5%  H&YS [%]: Stage 1 = 16% / Stage 2 = 61% / Stage 3 = 18% / Stage 4 = 5%  Duration of PD (years) [mean (SD)] = 7.1 (4.74) / Range = 1-22.4 | Items, scores and MDC of Neuro-QOL domains:   - Positive Affect and Well-Being: N (items) = 9 / Mean (SD) = 54.40 (7.53) / MDC = 10.23 - Applied Cognition–General Concerns: N (items) = 8 / Mean (SD) = 44.35 (7.62) / MDC =11.18 - Applied Cognition–Executive Function: N (items) = 8 / Mean (SD) = 46.25 (8.38) / MDC = 10.96 - Lower Extremity Function–Mobility: N (items) = 8 / Mean (SD) = 45.80 (7.54) / MDC = 9.71 - Upper Extremity Function–Fine Motor, ADL: N (items) = 8 / Mean (SD) = 42.28 (8.34) / MDC = 12.27 - Ability to Participate in Social Roles and Activities: N (items) = 8 / Mean (SD) = 47.85 (6.83) / MDC =10.14 - Satisfaction with Social Roles and Activities: N (items) = 8 / Mean (SD) = 46.21 (5.70) / MDC = 9.05 - Depression: N (items) = 8 / Mean (SD) = 45.85 (6.86) / MDC =10.70 - Anxiety: N (items) = 8 / Mean (SD) = 50.82 (6.80) / MDC = 9.02 - Stigma: N (items) = 8 / Mean (SD) = 48.39 (6.62) / MDC = 5.75 - Fatigue: N (items) = 8 / Mean (SD) = 46.04 (7.75) / MDC = 10.06 - Sleep Disturbance: N (ítems) = 8 / Mean (SD) = 47.70 (7.98) / MDC = 10.04 - Emotional and Behavioral Dyscontrol: N (ítems) = 8 / Mean (SD) = 43.49 (8.36) / MDC = 12.07 |
| Kuspinar (2019) [77] | Canada (Montreal) | 76 | Age [mean (SD)] = 69.1 y (9.5)  Women [%] = 41%  Years since PD symptoms onset [mean (SD)] = 8.2 (5.1)  Tremor dominant [%]= 71% / Akinetic rigid = 29%  History of falls [%]: None = 63% / Rare = 25% / Monthly = 12%  Levodopa equivalent dose (mg) [mean (SD)] = 770.4 (523.7)  PDQ-8 [mean (SD)] = 27 (14.2)  SF-36 Physical Function Index [mean (SD)] = 63.9 (27.7)  GDS [mean (SD)] = 2.1 (2.4)  Perceived Deficits Questionnaire [mean (SD)] = 2.1 (2.4)  Questionnaire-20 [mean (SD)] = 25.8 (11.9)  Apathy Scale [mean (SD)] = 22.3 (3.9)  H&YS [mean (SD)] = 2.3 (0.9)  Duration of PD (years) [mean (SD)] = 6.0 (4.1) | PGI most affected areas: Fine psychomotor skills (36%) > Walking (28%) > Sleep (25%) > Fagiue (24%) > Dyskinesia (16%) > Speech (4%) > Social life (14%) > Household tasks (11%)  PGI score for reported areas [mean (SD)]:   - Fine psychomotor skills = 3.6 (1.9) - Walking = 4.9 (2.2) - Sleep = 3.7 (1.5) - Fatigue = 4.5 (1.2) - Cognition = 4.8 (2.5) - Tremor / Dyskinesia = 5.1 (2.5) - Sport = 3.8 (2.0) - Depression / Anxiety = 3.8 (1.4) - Self-care = 4.3 (1.9) - Speech = 3.8 (2.0) - Social life = 4.2 (2.0) - Household tasks = 3.8 (2.1) - Bowel and bladder = 4.0 (2.9) - Work / Employment = 4.1 (0.9) - Balance / Falls = 6.4 (2.1) |
| Kuspinar (2020) [78] | Canada (Montreal) | 76 | Age [mean (SD)] = 69.1 y (9.5)  Women [%] = 40.79 %  Years since PD symptoms onset [mean (SD)] = 8.2 (5.1)  Tremor dominant [%]= 71% / Akinetic rigid = 29%  History of falls [%]: None = 63% / Rare = 25% / Monthly = 12%  Levodopa equivalent dose (mg) [mean (SD)] = 770.4 (523.7)  PDQ-8 [mean (SD)] = 27 (14.2)  SF-36 Physical Function Index [mean (SD)] = 63.9 (27.7)  GDS [mean (SD)] = 2.1 (2.4)  Perceived Deficits Questionnaire [mean (SD)] = 2.1 (2.4)  Questionnaire-20 [mean (SD)] = 25.8 (11.9)  Apathy Scale [mean (SD)] = 22.3 (3.9)  H&YS [mean (SD)] = 2.3 (0.9)  Duration of PD (years) [mean (SD)] = 6.0 (4.1) | PGI score (0-10) [mean (SD)] = 4.2 (1.8) / Range = 0-10 |
| Hagell (2011) [79] | Sweden | 150 | Age [mean (SD)] = 70.4 y (7.9) / Range = 49-85  Women [%] = 43.8%  Marital status [%, Married or cohabitant] = 72.6%  H&YS [median (P25-P75)] = 1 (1-2) / Range = 1-4  Duration of PD (years) [mean (SD)] = 5.0 (4.9) / Range = 0.5-25 | SF-12 response rate = 88.0% |
| Steffen (2008) [80] | United States | 37 | Age [mean (SD)] = 71.0 y (12.0)  Women [%] = 29.73%  UPDRS [mean] = 33 / Range = 7-70  Ethnicity [%]: White or non-hispanic = 97.3% / Asian = 2.70%  Living arrangement [%]: Cohabitant = 86.49% / Solo = 8.11% / Institucionalized = 5.41%  Number of falls (last 6 months) [mean] = 7 / Range = 0-182 [21 participantes suffered at least one fall]  Treatment with levodopa [%, Yes] = 83.78%  Daily dose of levodopa (mg): Media (DE) = 412 / Range = 125-1.150  Number of comorbidities [mean (SD)] = 3 (2) / Range = 0-6  Comorbidities [%, Yes]: Osteoarthritis = 45.95% / Asthma = 8.11% / History of cancer = 18.92% / Hypertension = 29.73% / Low blood pressure = 13.51% / Diabetes = 2.70% / Previous fracture = 21.62% / Depression or other mental health problem = 24.32% / History of cardiac disease = 16.22% / Osteoporosis = 18.92% / Cerebrovascular disease = 2.70% / Other = 37.84%  H&YS [%]: Stage 1 = 35.14% / Stage 2 = 18.92% / Stage 3 = 24.32% / Stage 4 = 21.62% / Stage 5 = 0.0%  Duration of PD (years) [mean (SD)] = 14.0 (6.0) | SF-36 response rate = 100%  SF-36 scores for dimensions:   - Physical functioning [mean (SD)] = 57 (23) / 95CI = 49-65 / MDC = 28 - Physical role limitations [mean (SD)] = 47 (41) / 95CI = 33-61 / MDC = 45 - Pain [mean (SD)] = 68 (27) / 95CI = 59-77 / MDC = 25 - General health perceptions [mean (SD)] = 59 (26) / 95CI = 50-67 / MDC = 28 - Energy [mean (SD)] = 52 (20) / 95CI = 45-59 / MDC = 19 - Social functioning [mean (SD)] = 83 (20) / 95CI = 76-90 / MDC = 29 - Emotional role limitations [mean (SD)] = 75 (40) / 95CI = 61-89 / MDC = 45 - Mental health [mean (SD)] = 76 (16) / 95CI = 70-81 / MDC = 19 |
| Hagell (2008) [81] | Sweden | 202 | Age [mean (SD)]: First administration = 69.8 y (10.0) / Subsequent administration = 70.0 y (8.6)  Women [%]: First administration = 46.5% / Subsequent administration = 46.0%  Self-perceived severity (Theoretical range 1-5) [median (P25-P75]: First administration = 2 (2-2) / Subsequent administration = 2 (1-2)  Motor fluctuation [%, Yes]: First administration = 678% / Subsequent administration = 65.0%  Diskynesia [%, Yes]: First administration = 49.0% / Subsequent administration = 65.0%  Work status [%, Retired]: First administration = 70.8% / Subsequent administration = 70.1%  Living arrangement [%, Married or cohabitant]: First administration = 71.2% / Subsequent administration = 73.7%  Living arrangement [%, Living alone]: First administration = 88.6% / Subsequent administration = 87.6%  H&YS [median (P25-P75)] = 3 (2-4)  Duration of PD (years) [mean (SD)]: First administration = 8.7 (6.4) / Subsequent administration = 6.4 (6.6) | SF-36 response rate = 97.0%  SF-36 scores for dimensions:   - Physical functioning [mean (SD)] = 51.75 (29.79) / Range (SD [Range]) = 1.39-2.49 (0.63-0.82) / Floor effect = 5.1% / Ceiling effect = 5.6% - Physical role limitations [mean (SD)] = 35.02 (39.98) / Range (SD [Range]) = 1.27-1.51 (0.45-0.50) / Floor effect = 46.2% / Ceiling effect = 20.8% - Pain [mean (SD)] = 58.69 (27.45) / Range (SD [Range]) = 3.83-4.04 (1.41-1.44) / Floor effect = 2.0% / Ceiling effect = 19.6% - General health perceptions [mean (SD)] = 47.25 (21.34) / Range (SD [Range]) = 2.47-4.14 (1.07-1.29) / Floor effect = 10.0% / Ceiling effect = 10.0% - Energy [mean (SD)] = 51.03 (24.76) / Range (SD [Range]) = 2.96-4.62 (1.45-1.60) / Floor effect = 0.5% / Ceiling effect =1.5% - Social functioning [mean (SD)] = 70.96 (26.23) / Range (SD [Range]) = 2.00-3.67 (1.09-1.23) / Floor effect = 0.5% / Ceiling effect = 30.8% - Emotional role limitations [mean (SD)] = 50.43 (44.95) / Range (SD [Range]) = 1.42-1.55 (0.50-0.50) / Floor effect = 38.5% / Ceiling effect = 39.5% - Mental health [mean (SD)] = 68.01 (20.74) / Range (SD [Range]) = 3.48-5.14 (1.17-1.52) / Floor effect = 0.5% / Ceiling effect = 6.6% |
| Schneider (2010) [82] | Germany | 215 | Age [mean (SD)] = 68.2 y (8.9) / Range = 33-84  Women [%] = 45.0%  UPDRS-Total [mean (SD)] = 45.3 (20.5) / Range = 10-123  UPDRS Item 3 (Depression) [mean (SD)]) = 0.81 (0.88) / Range = 0-4  UPDRS Item 4 (Motivation / Initiative) [mean (SD)] = 0.84 (0.85) / Rango = 0-3  Motor fluctuations [%, Yes] = 40%  Affective fluctuations [%, Yes] = 14%  H&YS [mean (SD)] = 2.4 (0.8) / H&YS [%]: Stages 1+1.5 = 17% / Stages 2+2.5 = 55% / Stage 3 = 18% / Stage 4 = 8% / Stage 5 = 1%  Duration of PD (years) [mean (SD)] = 7.4 (6.6) / Range = 1-48 | WHO-5 response rate = 99.1%  WHO-5 total score [mean (SD)] = 14.4 (5.5) / Range = 1-25  Floor effect = 0.0% / Ceiling effect = 11.74% |
| Hendred (2016) [83] | United States | 96 | Age [mean (SD)] = 62.44 y (5.3)  Women [%] = 44.79%  Educación (años): Media (DE) = 15.3 (2.3)  Race: White or Caucasic = 93.75% / Black or African-American = 1.04% / American Indian or Native from Alaska = 2.08% / Asian = 1.04% / Declined to state = 2.08%  BDI-II [mean (SD)] = 11.4 (8.0)  MMSE: Media (DE) = 29.0 (1.2)  UPDRS-Motor (medication) [mean (SD)] = 17.7 (8.3)  Daily dose of levodopa (mg) [mean (SD)] = 966 (6,801)  H&YS [%]: Stage 1 = 5.21% / Stage 2 = 81.25% / Stage 2.5 = 11.46% / Stage 3 = 2.08%  Duration of PD (years) [mean (SD)] = 5.0 (4.3) | Floor effects:   - Physical health = 0.0% - Psychological health = 0.0% - Social relationships = 0.0% - Environment = 0.0% - Overall QoL = 0.0% - Satisfaction with health = 7.3%   Ceiling effects:   - Physical health = 0.0% - Psychological health = 1.0% - Social relationships = 4.2% - Environment = 3.1% - Overall QoL = 24.0% - Satisfaction with health = 2.1% |
