# Supplementary material for: Quality of life measures in Parkinson’s disease: a systematic literature review of patient-reported outcomes measures (PROMs) and their psychometric properties
Source: J Neurol. 2025 Aug 28;272(9):598. doi: 10.1007/s00415-025-13348-x (PMC12394374; doi:10.1007/s00415-025-13348-x)
Supplement: Supplementary file 6 — Supplementary file6 (DOCX 116 KB) [file 415_2025_13348_MOESM6_ESM.docx]

Quality of Life Measures in Parkinson’s Disease: A Systematic Literature Review of Patient-Reported Outcomes Measures (PROMs) and their Psychometric Properties

**– ONLINE RESOURCE 4 –**

Table S10. Description of the PROMs and summary of feasibility evidence.

| PROMs | [References] | Description | Feasibility evidence |
| --- | --- | --- | --- |
| Specific PROMs for PD | | | |
| Bela-P-K | [1–3] | The Bela-P-K, which was originally developed in Deutch, includes 19 items grouped in 4 subscales: 1) Achievement capability / Physical symptoms (questions 1-5); 2) Fear / Emotional symptoms (questions 6-9); 3) Social functioning (questions 10-14); and 4) Partner-bonding / Family (questions 15-19). Each question is first answered by determining whether the problem is present (“Yes” / “No”). Then, for those aspects highlighted the patient describe the self-perceived discomfort produced by the item (“Bothered by” [Bb]). Finally, the patient describes any potential loss of autonomy due to the item (“Need for help” [Nfh]). Both Bb and Nfh are rated in 5-point Likert scales. The result of the Bela-P-K consists of two sub-total scores by summing the scores of each item. | The identified validation studies for the use of Bela-P-K were conducted in Netherlands, Italy and France.  Mean Bela-P-K scores for Bb ranged from 22.1 to 24.0, and from 19.7 to 24.8 for Nfh. The reported response rate was 98.0%. No data were available regarding the floor and ceiling effects, MIC and administration time. |
| Indo-PDQOL | [4] | The Indo-PDQOL, which was originally developed in Hindi for indigenous population, includes 70 items grouped in 12 dimensions: 1) ADL; 2) Mobility; 3) Psychological; 4) Fear; 5) Social; 6) Family; 7) Treatment related; 8) Finance; 9) Pain, sleep & RLS; 10) Memory & RBD; 11) Autonomic disturbance; and 12) Sexual & General health satisfaction. | The identified validation study was for the use of Indo-PDQOL was conducted in India.  No data were available regarding the scores range, response rate, floor and ceiling effects, MIC and administration time. |
| OFFELIA | [5,6] | The OFFELIA, which was designed for measuring the impact of Off episodes on QoL and ADL, is a 18-item questionnaire evaluating: 1) Physical activity; 2) Leisure/hobbies; 3) Employment [3a) Employment (among those who were employed)]; 4) Relationship to care partner; 5) Friendship; 6) Household tasks; 7) Driving; 8) Self-care/grooming; 9) Independence; 10) Communication; 11) Freedom to leave the home; 12) Scheduled activities; 13) How much impact do the OFF periods have on your daily life?; 14) OFF periods frustrate me [Frustration]; 15) OFF periods make me anxious [Anxiety]; 16) Having OFF periods is scary [Scary]; 17) Having OFF periods has hurt my self-esteem [Self-Esteem]; and 18) OFF periods make me feel embarrassed [Embarrassed]. The items are grouped in two domains: Functioning and Emotional wellbeing. Items 1-12 are rated in a 5-points Likert scale ranging from 1 (No impact) to 5 (Severe impact). The remaining items (13-18) are answered by using a 5-points Likert scale ranging from 1 (Strongly disagree) to 5 (strongly agree). Thus, higher scores are indicative of a greater impairment of QoL**.** | The identified validation study for the use of OFFELIA was conducted in the United States.  No floor or ceiling effects were observed. No data were available regarding mean scores, response rate, MIC and administration time. |
| PDQ-39 | [7–48] | PDQ-39 includes 39 items for measuring QoL, which are grouped in 8 dimensions: 1) Mobility; 2) ADL; 3) Emotional wellbeing; 4) Stigma; 5) Social support; 6) Cognition; 7) Communication; 8) Bodily discomfort. Each item is answered according to its frequency perceived by the patient during the last month, by using a 5-point Likert scale ranging from 0 (Never) to 4 (Always). The scores of the domains of this PROM are expressed from 0 to 100, higher scores being indicative of a greater impairment of QoL. Additionally, a summary index (SI) can be calculated to obtain an overall score for all domains. | The identified validation studies for the use of PDQ-39 were conducted in United Kingdom, Spain, United States, Canada, Greece, China, Sweden, Italy, Japan, Singapore, Finland, Ecuador, Estonia, Germany, Serbia, Iran, South Korea, Poland, Portugal, Philippines, South Africa and Luxembourg.  Mean PDQ-39 SI scores ranged between 21.7-91.7. The reported response rates ranged from 28.0% to 99.0%. The observed floor effect ranged between 0.0% (SI) and 68.5% (Social support). The ceiling effect ranged between 0.0% (SI and all dimensions) and 40.7% (Social support). The estimated MIC for the PDQ-39 SI was 12.7. The estimated administration time was 30-60 minutes. |
| PDQ-8 | [9,20,25,34,38,39,  47,49–56,56,57] | PDQ-8 is the reduced version of the PDQ-39 including 1 item of the extended version per domain: 1) Mobility; 2) ADL; 3) Emotional wellbeing; 4) Stigma; 5) Social support; 6) Cognition; 7) Communication; 8) Bodily discomfort. Each item is answered according to its frequency perceived by the patient during the last month, by using a 5-point Likert scale ranging from 0 (Never) to 4 (Always). The scores of the domains of this PROM are expressed from 0 to 100, higher scores being indicative of a greater impairment of QoL. | The identified validation studies for the use of PDQ-39 were conducted in United Kingdom, Singapore, Greece, United States, Canada, Italy, Spain, Japan, China, Iran, Poland, Mexico, Turkey, Korea, France, Germany, Netherlands, Portugal and Sweden.  Mean PDQ-8 score ranged between 11.82-47.25. The reported response rates ranged from 28.0% to 100%. The observed floor effect ranged between 1.1% and 4.8%. The ceiling effect ranged between 0.0% and 1.7%. The estimated MIC for PDQ-8 was 5.43. The estimated administration time was 5-12 minutes. |
| PDQ-DAT | [58] | The PDQ-DAT is a QoL measure specific for patients with PD using device-aided therapy (DAT). The questionnaire includes 24 items grouped in three categories: 1) ADL; 2) Problems related with the therapy device; and 3) Psychological problems. The items are answered according to the patient’s perceived frequency in a 5-point Likert scale ranging from 0 (Never) to 4 (Regularly). The scores for the PROM’s categories can be transformed into a percentage and, additionally, a SI can be calculated (ranging from 0 to 100). | The identified validation study for the use of PDQ-DAT was conducted in Japan.  No data were available regarding mean PDQ-DAT scores, response rates, floor or ceiling effect, MIC/MDC and administration time. |
| PDQL | [26,30,42,59–62] | The PDQL includes 37 items for measuring QoL, which are grouped in 4 domains: 1) Parkinsonian symptoms; 2) Systemic symptoms; 3) Emotional functioning; and 4) Social functioning. The items are answered on a 5-point Likert scale according to the patient’s perceived frequency in the last 3 months, ranging from 1 (All of the time) to 5 (Never). The score ranges between 37 and 185, higher scores being indicative of worse QoL (the score can be transformed into a percentage). | The identified validation studies for the use of PDQL were conducted in Netherlands, Ecuador, Brazil, Turkey and Portugal.  Mean PDQL scores ranged between 43.0-125.3. The reported response rate was 85.1%. No significant floor or ceiling effects were observed in the studies identified. No data were available regarding the MIC/MDC and administration time. |
| PDQoL7 | [57] | The PDQoL7 includes 7 items representing each one the dimensions impacting on QoL: 1) Mobility; 2) Skills & personal care; 3) Social Life-Communication; 4) Problems with non-motor symptoms; 5) Emotional status; 6) Mental status; and 7) Sleep. The items are answered on a 5-point Likert scale ranging from 0 (Never) to 4 (Always) according to the patient’s perceived frequency during the last week. Thus, higher scores are indicative or worse QoL. | The validation study identified for the use of PDQoL7 was conducted in Greece.  Mean PDQoL7 score was 14.58. The reported response rate was 100%. The observed floor and ceiling effect were 3.3% and 1.7%, respectively. No data were available regarding the MIC/MDC and administration time. |
| PDQUALIF | [63] | PDQUALIF is a 33-items PROM for measuring QoL, including 7 dimensions: 1) Social / Role life (8 items); 2) Self-image / Sexuality (7 items); 3) Sleep (3 items); 4) Outlook (4 items); 5) Physical function (5 items); 6) Independence (2 items); and 7) Urinary function (2 items). The items are answered in a 4-points Likert scale ranging from 0 (Never) and 4 (Always), according to the score that best describes the patient’s situation (items 25-33 refer to the last 7 days). The PDQUALIF scores range between 0 to 100 (higher scores are indicative of worse QoL). | The validation study identified for the use of PDQUALIF was conducted in United States and Canada.  Mean PDQUALIF score was 41.3. The reported response rate was 95.28%. The observed floor effect ranged between 0.0% (total score) and 78.9% (Independence). The observed ceiling effect ranged between 0.0% (total score, Self-image / Sexuality and Physical function) and 3.1% (Sleep). The estimated administration time was 10-15 minutes. |
| PIMS | [30,64,65] | The PIMS includes 10 items measuring: 1) Self (positive); 2) Self (negative); 3) Family and relationships; 4) Community relationships; 5) Work; 6) Leisure; 7) Travel; 8) Safety; 9) Financial security; and 10) Sexuality. The items can be grouped in 4 dimensions. Each one is answered according to the perceived impact of PD in the patient’s life, by providing a score on a 4-point Likert scale ranging from 1 (Slight) to 4 (Severe). The score 0 is available for “No change”. All items can be answered according to whether the PD symptoms are stable (column 1) or not (column 2a for worse and 2b for best). The PIMS provides a score ranging between 0-40 (higher scores are indicative of worse QoL). | The identified validation studies for the use of PIMS were conducted in Canada and Ecuador.  Mean PIMS score ranged between 0-38.5. The reported response rate was 96.2%. The observed floor effect was 1.1%. The observed ceiling effect was 2.8%. No data were available regarding the MIC/MDC and administration time. |
| QLPD | [66] | The QLPD is a 46-items PROM including 9 dimensions: 1) ADL (6 items); 2) Mobility (4 items); 3) Psychological (4 items); 4) Fear (4 items); 5) Social (4 items); 6) Family (3 items); 7) Treatment (3 items); 8) Finance (3 items); and 9) Non-motor symptoms (14 items). The items are answered in a 5-points Likert scale ranging from 0 (Very Bad) and 4 (Very good). The total score ranges between 0 and 100, higher scores being indicative of better QoL. The PROM also includes a visual analogue scale for patients to determine the perceived overall impact of QoL, ranging from 0 (worst possible state) to 100 (happiness, joy in life, no comprise of QoL). | The identified validation study for the use of QLPD was conducted in India.  Mean QLPD score was 35.3. The reported response rate was 98.33%. No floor or ceiling effects were observed. The administration time was 15-45 minutes. No data was available regarding MIC/MDC. |
| QLSM-DBS | [67,68] | The QLSM-DBS is a module of the QLSM specific for measuring the impact on QoL in patients treated with deep brain stimulation. The PROM includes 5 duplicated items, which are answered referring to the last 4 weeks on a 5-point Likert scale anchored by “Not important” and “Extremely important” for the first 5 items, and by “Dissatisfied” and “Very satisfied” for the repeated items. Higher scores of QLSM-MD are indicative of better QoL. | The identified validation studies were conducted in Germany and Poland.  Mean QLSM-MD scores ranged from 4.7 to 21.5. The reported response rate was 89.30%. The floor effect was 0.0%. The ceiling effect was 0.0%. No data were available regarding MIC/MDC and administration time. |
| QLSM-MD | [67,68] | The QLSM-MD is a module of the QLSM specific for movement disorders. The PROM includes 12 duplicated items, which are answered referring to the last 4 weeks on a 5-point Likert scale anchored by “Not important” and “Extremely important” for the first 12 items, and by “Dissatisfied” and “Very satisfied” for the repeated items. Higher scores of QLSM-MD are indicative of better QoL. | The identified validation studies were conducted in Germany and Poland.  Mean QLSM-MD scores ranged from 0.1 to 37.1. The reported response rate was 99.6%. The floor effect was 0.0%. The ceiling effect was 0.0%. No data were available regarding MIC/MDC and administration time. |
| QoLQ-PwP | [69] | Not enough data was available for describing the QoLQ-PwP. | The identified validation study was conducted in India.  No data were available regarding the QoLQ-PwP mean score, response rate, floor and ceiling effect, MIC/MDC and administration time. |
| QOLSQ | [70] | The QOLSQ is a 19-item questionnaire reflecting 4 domains of QoL, specifically related with swallowing disturbances. Each item is answered on a 4-point Likert scale ranging from 0 (Never) to 3 (Always), according to the patient perceived frequency during the last month. The total score ranges between 0-76 (can be transformed into a percentage), higher scores being indicative of better QoL. | The identified validation study was conducted in Brazil.  Mean QLOSQ score was 44.2. No data were available regarding response rate, floor and ceiling effects, MIC/MDC and administration time. |
| Unspecific PROMs validated for PD | | | |
| 15D | [22,71,72] | Multidimensional and self-administered PROM, which includes 15 dimensions (Mobility, Vision, Hearing, Breathing, Sleeping, Eating, Speech, Elimination, Usual activities, Mental function, Discomfort and symptoms, Depression, Distress, Vitality, Sexual activity). Each dimension is answered by selecting between 5 options. The 15D provides a single index measure ranging from 0 (death) to 1 (perfect health). The PROM also provides a health profile measure. | The identified validation studies for the use of 15D in PD were conducted in Finland and Spain.  Mean 15 utility values observed in the studies ranged from 0.70 to 0.74. The floor effect was 0.0%. The ceiling effect ranged between 0.4-0.8%. No data were available regarding response rate, MIC/MDC and administration time in patients with PD. |
| EQ-5D-3L | [13,56,73,74] | Multidimensional and self-administered PROM including 5 dimensions: 1) Mobility; 2) Self-care; 3) Usual activities; 4) Pain / Discomfort; and 5) Anxiety / Depression. All dimensions are answered by selecting between 3 options. The EQ-5D-3L provides a single index measure ranging from 0 (death) to 1 (perfect health). The PROM also provides a health profile measure. | The identified validation studies for the use of EQ-5D-3L in PD were conducted in United Kingdom, Singapore (both English and Chinese speakers).  Mean EQ-5D-3L utility values observed in the studies ranged from 0.64 to 0.74. The reported response rate ranged between 78.2-99.0%. No data were available regarding MIC/MDC and administration time in patients with PD. |
| EQ-5D-5L | [54,71] | Multidimensional and self-administered PROM including 5 dimensions: 1) Mobility; 2) Self-care; 3) Usual activities; 4) Pain / Discomfort; and 5) Anxiety / Depression. All dimensions are answered by selecting between 5 options. The EQ-5D-3L provides a single index measure ranging from 0 (death) to 1 (perfect health). The PROM also provides a health profile measure. | The identified validation studies for the use of EQ-5D-3L in PD were conducted in Spain and Mexico.  Mean EQ-5D-3L utility values observed in the studies ranged from 0.59 to 0.71. The floor effect was 0.0%. The ceiling effect ranged between 5.3-8.8%. No data were available regarding response rate, MIC/MDC and administration time in patients with PD. |
| EQ-VAS | [13,54,73] | Visual analogue scale ranging from 0 (worse health state imaginable) to 100 (best health state imaginable), which is asked to be answered according to the self-perceived health state of the patient in the moment. This scale is administered together with EQ-5D-3L or EQ-5D-5L. | The identified validation studies for the use of EQ-VAS in PD were conducted in Mexico, United Kingdom and Singapore (both English and Chinese speakers).  Mean EQ-VAS score observed in the studies ranged from 71.1 to 73.8. The reported response rates ranged between 78.2-99.0%. No data were available regarding floor and ceiling effects, MIC/MDC and administration time in patients with PD. |
| McGill QOL | [45] | The McGill QOL, which was designed for measuring QoL in patients suffering life-threatening illness, includes 16 items grouped in 4 domains: 1) Symptoms and wellbeing (4 items); 2) Psychological wellbeing (4 items); 3) Existential issues (6 items); and 4) Support (2 items). These items are answered by using 11-point Likert scales, producing total scores ranging between 0-160. The questionnaire includes an additional single item referring to global QoL and allows patients to report qualitative information on the symptoms that most impact QoL. | The identified validation study for the use of McGill QOL in PD was conducted in United States (California) and Canada (Alberta).  The mean score was 117.8. No floor or ceiling effect were observed. The estimated MIC was 3.9. The administration time is 10-30 minutes in general palliative populations. No data were available regarding the response rate. |
| Neuro-QOL | [75,76] | The Neuro-QOL includes 13 item banks referring to common issues that impact the QOL of patients suffering different neurological diseases: 1) Mental wellbeing (Anxiety; Depression; Positive Affect and Well-Being; Applied Cognition–General Concerns; Applied Cognition–Executive Function; Emotional and Behavioral Dyscontrol and Stigma); 2) Physical wellbeing (Upper Extremity Function–Fine Motor, ADL; Lower Extremity Function–Mobility; Sleep Disturbance; and Fatigue); and 3) Social wellbeing (Ability to Participate in Social Roles and Activities; Satisfaction with Social Roles and Activities). | The identified validation study for the use of Neuro-QOL in PD was conducted in United States.  The mean scores for each item bank ranged between 42.28 (Upper Extremity Function–Fine Motor, ADL) and 54.40 (Positive Affect and Well-Being). The estimated MDC ranged from 5.75 (Stigma) to 12.27 (Upper Extremity Function–Fine Motor, ADL). No data were available regarding floor and ceiling effects, and administration time. |
| PGI | [77,78] | The PGI is and QoL measure administered in three steps: 1) Report of the 5 most important areas affected by the disease; 2) Rating the perceived impact on the selected areas in a Likert scale ranging from 0 (worst imaginable) to 10 (exactly as the patient’s would like to live), and rating other health and non-health areas; and 3) Distribution of 12 spending “points” across the areas (the more points allocated in a specific area, the more important the area is for the patient). The PGI provides a single score between 0-100 (higher scores are indicative of better QoL). | The identified validation studies for the use of PGI in PD were conducted in Canada.  The mean score was 4.2 (in a 0-10 scale). No data were available regarding the response rate, floor or ceiling effects, MIC/MDC and administration time. |
| PROMIS-29 | [45] | The PROMIS-29 is a 29-items PROM validated for several health conditions, which measures (4 items per domain): 1) Anxiety; 2) Depression; 3) Fatigue; 4) Sleep disturbances; 5) Pain interference; 6) Current physical condition; and 7) Ability to participate in social roles and activities. All items are answered in a 5-point Likert scale anchored by “Never” and “Always”, according to the patient perceived frequency over the prior week. Additionally, a separate 11-point Likert scale allows to measure pain intensity. The total score ranges from 32 to 160, higher scores being indicative of worse QoL. | The identified validation study for the use of PROMIS-29 in PD was conducted in United States and Canada.  The mean score was 74.9. No floor or ceiling effects were observed. The estimated MIC was 10.9. The administration time was 4-8 minutes. No data was available regarding response rate. |
| QOL-AD | [45] | The QOL-AD is a 13-item questionnaire designed originally for measuring QoL in Alzheimer’s Disease. The PROM measures the symptoms on 4-points Likert scales ranging from “Poor” to “Excellent”. The total scores ranges between 13 to 52, higher scores being indicative of better QoL. | The identified validation study for the use of QOL-AD in PD was conducted in United States and Canada.  The mean score was 34.0. No floor or ceiling effects were observed. The estimated MIC was 18.9. The administration time was 6-8 minutes. No data was available regarding response rate. |
| SF-12 | [79] | The SF-12, which is the abbreviated version of the SF-36, is a generic 12-items PROM including one or two items per domain. Although the PROM is not able to provide a health index due to its length, it was designed to capture the summary score of the physical (PCS, items 1, 2, 3, 4, 5, 8) and mental (MCS, items 6, 7, 9, 10, 11, 12) components. Each item is answered by using different Likert scales (Excellent – Poor / Limited a lot – Not limited at all / All of the time – None of the time), as well as “Yes / No” questions. Higher scores are indicative of worse status for each component. | The identified validation study for the use of SF-12 in PD was conducted in Sweden.  The reported response rate was 88.0%. No data were available regarding mean SF-12 score, floor and ceiling effects, MIC/MDC and administration time. |
| SF-36 | [13,80,81] | The SF-36 is a generic 36-items PROM for including 8 dimensions of QoL: 1) Physical functioning; 2) Social functioning, 3) Physical role limitations; 4) Social role limitations; 5) Mental health; 6) Energy; 7) Pain; and 8) General health perceptions. The items are answered on different Likert scales and “Yes / No” questions. The total score of the SF-36 ranges between 0 and 100, higher scores being indicative of worse QoL. Additionally, a physical and mental summary score can be determined. | The identified validation studies for the use of SF-36 in PD were conducted in United Kingdom, United States and Sweden.  The reported response rates ranged between 78.2-100%. The floor effect ranged between 0.5% (Energy, Social functioning and Mental health) and 46.2% (Physical role limitations). The ceiling effect ranged between 1.5% (Energy) and 39.5% (Emotional role limitations). The estimated MIC ranged between 28 (Physical function and General health perceptions) and 45 (Emotional role limitations). No data were available regarding the mean SF-36 score and administration time in patients with PD. |
| SF-6D | [71,72] | The SF-6D is a preference-based measure of QoL derived from the SF-36. This PROM includes 6 dimensions: 1) Physical functioning; 2) Role limitations; 3) Social functioning; 4) Pain; 5) Mental health; and 6) Vitality. Each dimension is answered by determining the health state among the 4, 5 or 5 levels presented in the questionnaire (18,000 potential health states). Given that the SF-6D is a preference-based measure, the result is a utility ranging from 0 (death) to 1 (perfect health). | The identified validation studies for the use of SF-6D in PD were conducted in Spain.  Mean SF-6D utility values observed in the studies ranged from 0.51 to 0.53. The reported response rate was 84.7%. The floor effect was 0.0%. The ceiling effect ranged between 0.0% and 0.4%. No data were available regarding MIC/MDC and administration time in patients with PD. |
| WHO-5 | [82] | The WHO-5 is a PROM designed for measuring emotional wellbeing during the previous 2 weeks. The WHO-5 items are: 1) “I have felt cheerful and in good spirits”; 2) “I have felt calm and relaxed”; 3) “I have felt active and vigorous”; 4) “I woke up feeling fresh and rested”; and 5) “My daily life has been filled with things that interest me”. All items are answered by using a 6-point Likert scale ranging from 0 (At no time) to 5 (All of the time). The total score ranges from 0 to 25 (can be transformed into percentage), higher scores being indicative of better wellbeing. A cutoff point has been proposed for scores under 13 (50/100) indicating the presence of potential mental health conditions. | The identified validation study for the use of WHO-5 in PD was conducted in Germany.  Mean WHO-5 score was 14.4. The reported response rate was 99.1%. The floor effect was 0.0%. The ceiling effect was 11.7%. No data were available regarding MIC/MDC and administration time in patients with PD. |
| WHOQOL-BREF | [83] | The WHOQOL-BREF includes 26 items grouped in 4 dimensions: 1) Physical health; 2) Psychological health; 3) Social relationships; and 4) Environment. All items are answered on 5-points Likert scales referring to the previous 2 weeks. Additionally, two separate questions ask for the overall rating of QoL and general health satisfaction. The total score for each domain ranges between 4-20, higher scores being indicative of better QoL. | The identified validation study for the use of WHOQOL-BREF in PD was conducted in United States.  The floor effect ranged between 0.0% (Physical health, Psychological health, Social relationships. Environment, Overall QoL) and 7.3% (Satisfaction with health). The ceiling effect ranged between 0.0% (Physical health) and 24.0% (Overall QoL). No data were available for the mean WHOQOL-BREF score, response rate, MIC/MDC and administration time. |
