# Supplementary material for: Quality of life measures in Parkinson’s disease: a systematic literature review of patient-reported outcomes measures (PROMs) and their psychometric properties
Source: J Neurol. 2025 Aug 28;272(9):598. doi: 10.1007/s00415-025-13348-x (PMC12394374; doi:10.1007/s00415-025-13348-x)
Supplement: Supplementary file 7 — Supplementary file7 (DOCX 134 KB) [file 415_2025_13348_MOESM7_ESM.docx]

**Quality of Life Measures in Parkinson’s Disease: A Systematic Literature Review of Patient-Reported Outcomes Measures (PROMs) and their Psychometric Properties**

– ONLINE RESOURCE 5 –

Table S11. Descriptions of the studies’ findings in relation to the Structural Validity of the PROMs.

| Eligible study | Sample size | Findings of the study in relation to Structural Validity | COSMIN assessment | |
| --- | --- | --- | --- | --- |
|  |  |  | **RoB** | **Good property** |
| Spliethoff-Kamminga (2003) [1] | – | – | – | – |
| Ortelli (2017) [2] | – | – | – | – |
| Bayen (2021) [3] | – | – | – | – |
| Aggarwal (2013) [4] | 277 | The assessment of structural validity was based on CTT  EFA identified 12 domains: 1) ADL; 2) Mobility; 3) Psychological; 4) Fear; 5) Social; 6) Family; 7) Treatment related; 8) Finance; 9) Pain, sleep & RLS; 10) Memory & RBD; 11) Autonomic disturbance; and 12) Sexual & General health satisfaction  The results of the EFA are not available | Doubtful | (?) |
| Kuharic (2022) [5] | 569 | The assessment of structural validity was based on CTT and IRT   - CTT: The EFA identified two factors “Functioning” (13 items) and “Emotional wellbeing” (5 items) - IRT: Response levels to most levels ordered, but there was some evidence of DIF observed for the variable sex. A total of 15 items worked well, 2 presented mixed evidence and 1 performed poorly (employment). | Very good | (?) |
| Kuharic (2024) [6] | 569 | The assessment of structural validity was based on CTT and IRT   - CTT: Monte Carlo parallel analysis grouped items into two factors. EFA (Promax rotation) identified two factors “Functioning” (13 items; r = 0.42-0.89; eigenvalue = 10.28; variance explained = 88%) and “Emotional wellbeing” (5 items; r = 0.54-0.86; eigenvalue = 1.41; variance explained = 12%). Factor loadings were observed to be greater than 0.4 for all items - IRT: Some evidence of DIF by sex and location (no relevant magnitude; R^2^ Nagelkerke < 0.035). Best fitting model according to BIC identified two domains “Functioning” (13 items) and “Emotional wellbeing” (5 items). The item “employment” had mixed evidence to support its inclusion | Very good | (?) |
| Peto (1995) [7] | 359 | The assessment of structural validity was based on CTT, which was used for the reduction of the PROM’s items (inclusion of factors with eigenvalues > 1 and omission of items with factor loading ≤ 0,5 in any factor).  EFA identified 10 factors explaining a total of 68% of the variance. | Very good | (?) |
|  | 227 | CFA identified 8 factors. | Very good | (?) |
| Jenkinson (1997) [8] | 201 | The assessment of structural validity was based on CTT.  EFA identified 8 factors (factor loadings): “Mobility” (0.7604), “ADL” (0.75891), “Emotional wellbeing” (0.76307), “Stigma” (0.69736), “Social support” (0.63362), “Cognition” (0.75811), “Communication” (0.71458) and “Bodily discomfort” (0.61076). The total variance explained, and eigenvalue was 51.1% and 4.1, respectively. | Very good | (?) |
|  | 136 | The assessment of structural validity was based on CTT.  EFA identified 8 factors (factor loadings): “Mobility” (0.80558), “ADL” (0.79596), “Emotional wellbeing” (0.80797), “Stigma” (0.70343), “Social support” (0.69838), “Cognition” (0.71051), “Communication” (0.75518) and “Bodily discomfort” (0.73992). The total variance explained was 56.8% and eigenvalue 4.5. | Very good | (?) |
| Jenkinson (1997) [9] | Postal = 227  Clinical = 146 | **PDQ-39:**  The assessment of structural validity was based on CTT.  EFA identified 8 factors (factor loadings): “Mobility” (0,76404 / 0,80558), “ADL” (0,75891 / 0,79596), “Emotional wellbeing” (0,76307 / 0,80797), “Stigma” (0,69736 / 0,70343), “Social support” (0,63362 / 0,69838), “Cognition” (0,75811 / 0,71051), “Communication” (0,71458 / 0,75518) and “Bodily discomfort” (0,61076 / 0,73992). The total variance explained was 51.1% and 56.8% for postal and clinical samples, respectively. | – | – |
|  | – | **PDQ-8:**  – | Very good | (?) |
| Martínez-Martín (1998) [10] | – | – | – | – |
| Bushnell (1999) [11] | 75 | The assessment of structural validity was based on CTT.  CFA identified 8 dimensions with eigenvalues > 1, which explained 75.5% of the total variance. | Very good | (?) |
| Andreu (2000) [12] | – | – | – | – |
| Schrag (2000) [13] | – | **PDQ-39:**  – | – | – |
|  | – | **EQ-5D-3L:**  – | – | – |
|  | – | **EQ-VAS:**  – | – | – |
|  | – | **SF-36:**  – | – | – |
| Katsarou (2001) [14] | – | – | – | – |
| Peto (2001) [15] | – | – | – | – |
| Tsang (2002) [16] | – | – | – | – |
| Hagell (2003) [17] | 71 | The assessment of structural validity was based on IRT.  The Rasch model estimates were (response as a logistic function):   - Mobility: Disordered item response threshold [n] = 6 / Person strata [n] = 4.35 / Logit (SD) = -0.11 (1.99) - ADL: Disordered item response threshold [n] = 3 / Person strata [n] = 4.33 / Logit (SD) = -0.27 (1.37) - Emotional wellbeing: Disordered item response threshold [n] = 0 / Person strata [n] = 3.40 / Logit (SD) = -1.22 (1.85) - Stigma: Disordered item response threshold [n] = 1 / Person strata [n] = 2.63 / Logit (SD) = -0.74 (1.50) - Social support: Disordered item response threshold [n] = 2 / Person strata [n] = 1.31 / Logit (SD) = -0.97 (1.21) - Cognition: Disordered item response threshold [n] = 3 / Person strata [n] = 2.23 / Logit (SD) = -0.81 (1.60) - Communication: Disordered item response threshold [n] = 1 / Person strata [n] = 2.43 / Logit (SD) = -1.21 (2.15) - Bodily discomfort: Disordered item response threshold [n] = 1 / Person strata [n] = 2.07 / Logit (SD) = -0.15 (1.66) | Adequate | (?) |
| Jenkinson (2003) [18] | 676 | The assessment of structural validity was based on CTT.  EFA identified a total of 62.41% of variance explained by a single factor. | Very good | (?) |
|  | 676 | The assessment of structural validity was based on CTT.  EFA identified a total of 55.03% of variance explained by a single factor. | Very good | (?) |
|  | 676 | The assessment of structural validity was based on CTT.  EFA identified a total of 59.47% of variance explained by a single factor. | Very good | (?) |
|  | 676 | The assessment of structural validity was based on CTT.  EFA identified a total of 48.48% of variance explained by a single factor. | Very good | (?) |
|  | 676 | The assessment of structural validity was based on CTT.  EFA identified a total of 49.05% of variance explained by a single factor. | Very good | (?) |
| Park (2004) [19] | – | – | – | – |
| Tan (2004) [20] | 88 | **PDQ-39:**  The assessment of structural validity was based on CTT.  EFA identified 8 factors (factor loadings): “Mobility” (0.74), “ADL” (0.75), “Emotional wellbeing” (0.79), “Stigma” (0.50), “Social support” (0.55), “Cognition” (0.74), “Communication” (0.67) and “Bodily discomfort” (0.58). The total variance explained, and eigenvalue was 45.3% and 3.6, respectively. | Very good | (?) |
|  | – | **PDQ-8:**  – | – | – |
| Fitzpatrick (2004) [21] | – | – | – | – |
|  | – | – | – | – |
| Haapaniemi (2004) [22] | – | – | – | – |
| Martínez-Martín (2004) [23] | – | – | – | – |
| Ma (2005) [24] | – | – | – | – |
| Luo (2005) [25] | – | **PDQ-39:**  – | – | – |
|  | – | **PDQ-8:**  The assessment of structural validity was based on CTT.  The EFA considering a single factor estimated an eigenvalue of 3.9 and explained 48.9% of the total variance. Factor loadings for the 8 items ranged between 0.53-0.89 |  |  |
| Martínez-Martín (2007) [26] | – | **PDQ-39:**  – | – | – |
|  | – | **PDQL:**  Cronbach’s alpha (α) for PDQL (Parkinsonian symptoms / Systemic symptoms / Emotional functioning / Social functioning) = 0.88 / 0.70 / 0.81 / 0.83 | Very good | (+) |
| Hagell (2007) [27] | 202 | The assessment of structural validity was based on CTT and IRT.  CFA confirmed 8 factors (Chi-squared = 1,885.85; p < 0.0001).  Rasch model estimates were (response as a logistic function):   - Item 1 (item statistics: location = -0.51; SE = 0.10 / fit statistics: residual = 2.02; x^2^ = 4.17; F statistic = 1.67). - Item 2 (item statistics: location = -0.26; SE = 0.10 / fit statistics: residual = -0.27; x^2^ = 2.81; F statistic = 1.55). - Item 3 (item statistics: location = -0.36; SE = 0.08 / fit statistics: residual = 0.76; x^2^ = 5.20; F statistic = 3.44). - Item 4 (item statistics: location = -0.43; SE = 0.08 / fit statistics: residual = -0.08; x^2^ = 1.36; F statistic = 1.05). - Item 5 (item statistics: location = 0.68; SE = 0.09 / fit statistics: residual = -0.55; x^2^ = 7.45; F statistic = 4.77). - Item 6 (item statistics: location = 0.09; SE = 0.10 / fit statistics: residual = -0.17; x^2^ = 1.58; F statistic = 1.00). - Item 7 (item statistics: location = -0.24; SE = 0.10 / fit statistics: residual = -3.06; x^2^ = 12.36; F statistic = 13.02). - Item 8 (item statistics: location = 0.15; SE = 0.08 / fit statistics: residual = -0.34; x^2^ = 1.31; F statistic = 0.04). - Item 9 (item statistics: location = 0.53; SE = 0.09 / fit statistics: residual = 2.26; x^2^ = 3.50; F statistic = 1.64). - Item 10 (item statistics: location = 0.34; SE = 0.10 / fit statistics: residual = -0.30; x^2^ = 1.30; F statistic = 0.74). - Item 11 (item statistics: location = 0.79; SE = 0.09 / fit statistics: residual = -1.53; x^2^ = 7.18; F statistic = 6.06). - Item 12 (item statistics: location = 0.22; SE = 0.09 / fit statistics: residual = -1.90; x^2^ = 8.15; F statistic = 7.20). - Item 13 (item statistics: location = -0.56; SE = 0.09 / fit statistics: residual = -0.66; x^2^ = 4.16; F statistic = 2.98). - Item 14 (item statistics: location = -0.89; SE = 0.09 / fit statistics: residual = 1.59; x^2^ = 6.82; F statistic = 3.54). - Item 15 (item statistics: location = -0.07; SE = 0.09 / fit statistics: residual = -0.09; x^2^ = 0.57; F statistic = 0.62). - Item 16 (item statistics: location = 0.51; SE = 0.09 / fit statistics: residual = 2.86; x^2^ = 12.41; F statistic = 4.87). - Item 17 (item statistics: location = -0.91; SE = 0.11 / fit statistics: residual = -1.73; x^2^ = 3.99; F statistic = 3.30). - Item 18 (item statistics: location = 0.18; SE = 0.10 / fit statistics: residual = 0.62; x^2^ = 0.68; F statistic = 0.27). - Item 19 (item statistics: location = 1.22; SE = 0.11 / fit statistics: residual = 1.98; x^2^ = 1.97; F statistic = 0.88). - Item 20 (item statistics: location = 0.49; SE = 0.11 / fit statistics: residual = 1.25; x^2^ = 0.17; F statistic = 0.08). - Item 21 (item statistics: location = -0.43; SE = 0.11/ fit statistics: residual = -0.52; x^2^ = 2.83; F statistic = 1.81). - Item 22 (item statistics: location = -0.55; SE = 0.11/ fit statistics: residual = 0.68; x^2^ = 0.44; F statistic = 0.25). - Item 23 (item statistics: location = 0.05; SE = 0.10/ fit statistics: residual = 0.56; x^2^ = 0.92; F statistic = 0.22). - Item 24 (item statistics: location = -0.31; SE = 0.10/ fit statistics: residual = 1.70; x^2^ = 2.66; F statistic = 1.20). - Item 25 (item statistics: location = -0.12; SE = 0.10/ fit statistics: residual = -1.10; x^2^ = 7.77; F statistic = 6.90). - Item 26 (item statistics: location = 0.37; SE = 0.11 / fit statistics: residual = 0.56; x^2^ = 1.67; F statistic = 0.93). - Item 27 (item statistics: location = 0.47; SE = 0.12 / fit statistics: residual = 1.87; x^2^ = 5.87; F statistic = 3.53). - Item 28 (item statistics: location = -0.40; SE = 0.12 / fit statistics: residual = -0.87; x^2^ = 7.06; F statistic = 7.13). - Item 29 (item statistics: location = -0.07; SE = 0.11 / fit statistics: residual = -0.27; x^2^ = 4.27; F statistic = 3.27). - Item 30 (item statistics: location = 0.59; SE = 0.08 / fit statistics: residual = 1.65; x^2^ = 0.75; F statistic = 0.27). - Item 31 (item statistics: location = -0.59; SE = 0.09 / fit statistics: residual = -0.96; x^2^ = 11.68; F statistic = 9.99). - Item 32 (item statistics: location = -0.61; SE = 0.09 / fit statistics: residual = 1.01; x^2^ = 0.30; F statistic = 0.07). - Item 33 (item statistics: location = 0.60; SE = 0.09 / fit statistics: residual = 1.11; x^2^ = 1.32; F statistic = 0.64). - Item 34 (item statistics: location = -1.03; SE = 0.12 / fit statistics: residual = -0.50; x^2^ = 1.06; F statistic = 0.86). - Item 35 (item statistics: location = -0.80; SE = 0.13 / fit statistics: residual = -2.25; x^2^ = 8.17; F statistic = 10.78). - Item 36 (item statistics: location = 1.82; SE = 0.14 / fit statistics: residual = 2.31; x^2^ = 7.50; F statistic = 3.29). - Item 37 (item statistics: location = 0.41; SE = 0.08 / fit statistics: residual = -0.19; x^2^ = 7.15; F statistic = 5.59). - Item 38 (item statistics: location = -0.42; SE = 0.08 / fit statistics: residual = -0.22; x^2^ = 3.60; F statistic = 3.15). - Item 39 (item statistics: location = 0.00; SE = 0.08 / fit statistics: residual = 1.34; x^2^ = 0.04; F statistic = 0.02). | Very good | (?) |
| Krikmann (2008) [28] | – | – | – | – |
| Marinus (2008) [29] | 177 | The assessment of structural validity was based on CTT.  EFA identified 10 factors with eigenvalues ≥1 explaining 70% of the variance. Eight factors explained 65% of the total variance. | Very good | (?) |
| Serrano-Dueñas (2008) [30] | – | **PDQ-39**  – | – | – |
|  | – | **PDQL:**  – | – | – |
|  | – | **PIMS:**  – | – | – |
| Žiropađa (2009) [31] | 102 | The assessment of structural validity was based on CTT.  Hierarchical factor analysis estimates (secondary factor loading / primary factor 1 / primary factor 2):   - Mobility: 0.66 / -0.01 / 0.59 - ADL: 0.63 / 0.01 / 0.54 - Emotional wellbeing: 0.63 / 0.66 / -0.11 - Stigma: 0.59 / 0.59 / -0.08 - Social support: 0.60 / 0.50 / 0.02 - Cognition: 0.70 / 0.39 / 0.22 - Communication: 0.59 / -0.07 / 0.59 - Bodily discomfort: 0.71 / 0.55 / 0.07   Two factors explained 73% of the total variance. | Very good | (?) |
| Nojomi (2010) [32] | – | – | – | – |
| Luo (2010) [33] | – | – | – | – |
| Huang (2010) [34] | – | **PDQ-39:**  – | – | – |
|  | – | **PDQ-8:**  The assessment of structural validity was based on CTT.  CFA considering a single factor explained 44.12%. Model fit statistics: CFI = 0.95; RMSEA = 0.075; GFI = 0.92; AGFI = 0.86; Chi-Squared Idf = 1.56 (p = 0.053) | Very good | (+) |
| Zhang (2011) [35] | – | – | – | – |
| Kwon (2013) [36] | – | – | – | – |
| Park (2014) [37] | 93 | The assessment of structural validity was based on CTT.  EFA identified 8 factors (total variance explained = 55.4%; eigenvalue = 4.35). | Very good | (?) |
| Fereshtehnejad (2014) [38] | – | **PDQ-39:**  – | – | – |
|  | 114 | **PDQ-8:**  The assessment of structural validity was based on CTT.  EFA estimated that a single factor explained 37.31% of variance (a second factor would explain 16.59% of the variance). | Very good | (?) |
| Krygowska-Wajs (2015) [39] | – | – | – | – |
| Morley (2015, a) [40] | – | – | – | – |
| Morley (2015, b) [41] | 118 | The assessment of structural validity was based on CTT.  EFA identified 8 factors. | Doubtful | (?) |
| Jesus-Ribeiro (2017) [42] | – | **DPQ-39:**  – | – | – |
|  | – | **PDQL:**  – | – | – |
| Galeoto (2018) [43] | – | – | – | – |
| Suratos (2018) [44] | – | – | – | – |
| Holden (2019) [45] | 176 | **PDQ-39:**  The assessment of structural validity was based on CTT.  The factor analysis with oblique (Promax) rotation identified 8 factors explaining 67% of the variance. | Very good | (?) |
|  | 166 | **McGill QOL:**  The assessment of structural validity was based on CTT.  The factor analysis with oblique (Promax) rotation identified 3 factors explaining 54% of the variance. | Very good | (?) |
|  | 201 | **PROMIS-29:**  The assessment of structural validity was based on CTT.  The factor analysis with oblique (Promax) rotation identified 2 factors explaining 47% of the variance, and 6 factors explaining 73% of the variance. | Very good | (?) |
|  | 141 | **QOL-AD:**  The assessment of structural validity was based on CTT.  The factor analysis with oblique (Promax) rotation identified 3 factors explaining 57% of the variance. | Very good | (?) |
| Nelson (2020) [46] | 160 | The assessment of structural validity was based on CTT.  EFA identified 5 factors:   - Factor 1 (Mobility. Outside home. ADL) - Eigenvalue = 14.08 / Variance explained = 65% - Factor 2 (Emotional wellbeing) - Eigenvalue = 3.30 / Variance explained = 15% - Factor 3 (Cognition, Communication, Bodily discomfort) - Eigenvalue = 1.94 / Variance explained = 9% - Factor 4 (Home ADL) - Eigenvalue = 1.42 / Variance explained = 6% - Factor 5 (Social, Non intended sleep) - Eigenvalue = 1.21 / Variance explained = 6% | Very good | (?) |
| Kim (2020) [47] | 80 | **PDQ-39:**  The assessment of structural validity was based on CTT.  EFA identified 8 factors (factor loadings): Mobility (0.813), ADL (0.810), Emotional wellbeing (0.780), Social support (0.827), Cognition (0.686), Communication (0.827), Bodily discomfort (0.768) and Stigma (0.735). Other statistics: Kelser-Meyer-Olkin = 0,87; Bartlett's Chi-Cuadrado = 378,15 [p < 0,001]; eigenvalue = 4,89; total variance explained = 61,17%.  CFA confirmed 8 factors (Beta): Mobility (0.715), ADL (0.693), Emotional wellbeing (0.770), Social support (0.765), Cognition (0.786), Communication (0.760), Bodily discomfort (0.726) and Stigma (0.782). Fit statistics – Absolut fit index: Chi-Cuadrado = 32.02 [D.F. = 18; p = 0.002; Q = 1,779]; GFI (Goodness of Fit) = 0.915; RMR (Root mean square residual) = 0.042 / Relative fit index – NFI (Normed Fit Index) = 0.924; TLI (Tucker-Lewis Index) = 0.945; IFI (Incremental Fit Index) = 0.965 / Non-centricity index: CFI (Comparative fit index) = 0.964; RMSEA (Root mean square error of approximation) = 0.099. | Very good | (+) |
|  | 80 | **PDQ-8:**  The assessment of structural validity was based on CTT.  EFA identified 8 factors (factor loadings [Nested to PDQ-39 / Independent PDQ-8]): Mobility (0.0.707 / 0.712), ADL (0.809 / 0.812), Emotional wellbeing (0.0.687 / 0.584), Social support (0.0.849 / 0.847), Cognition (0.0.653 / 0.718), Communication (0.0.674 / 0.802), Bodily discomfort (0.0.579 / 0.585) and Stigma (0.0.765 / 0.792). Other statistics [Nested to PDQ-39 / Independent PDQ-8]: Kelser-Meyer-Olkin = 0,84 / 0.81; Bartlett's Chi-Cuadrado = 378,15 [p < 0,001] / 277.52 [p < 0.001]; eigenvalue = 4,15 / 4.35; total variance explained = 51.86% / 54.51%.  CFA confirmed 8 factors (Beta [Nested to PDQ-39 / Independent PDQ-8]): Mobility (0.0.681 / 0.665), ADL (0.0.781 / 0.785), Emotional wellbeing (0.616 / 0.508), Social support (0.615 / 0.770), Cognition (0.485 / 0.515), Communication (0.729 / 0.732), Bodily discomfort (0.620 / 0.671) and Stigma (0.840 / 0.832). Fit statistics [Nested to PDQ-39 / Independent PDQ-8] – Absolut fit index: Chi-Cuadrado = 34.05 / 25.03 [D.F. = 18; p = 0.002; Q = 1,779]; GFI (Goodness of Fit) = 0.0.907 / 0.932; RMR (Root mean square residual) = 0.0.088 / 0.074 / Relative fit index – NFI (Normed Fit Index) = 0.877 / 0.914; TLI (Tucker-Lewis Index) = 0.0.911 / 0.973; IFI (Incremental Fit Index) = 0.0.942 / 0.981 / Non-centricity index: CFI (Comparative fit index) = 0.0.940 / 0.981; RMSEA (Root mean square error of approximation) = 0.0.099 / 0.056. | Very good | (+) |
| Hanff (2023) [48] | – | – | – | – |
| Katsarou (2004) [49] | – | – | – | – |
| Tan (2007) [50] | – | – | – | – |
|  | 104 | The assessment of structural validity was based on CTT.  EFA considering a single factor explained 43.1%. Factor loadings for items: Mobility = 0.68 / ADL = 0.70 / Emotional wellbeing = 0.69 / Social support = 0.60 / Cognition = 0.65 / Communication = 0.75 / Bodily discomfort = 0.55 / Stigma = 0.62 | Very good | (?) |
|  | 79 | The assessment of structural validity was based on CTT.  EFA considering a single factor explained 52.3%. Factor loadings for items: Mobility = 0.73 / ADL = 0.69 / Emotional wellbeing = 0.70 / Social support = 0.70 / Cognition = 0.73 / Communication = 0.77 / Bodily discomfort = 0.70 / Stigma = 0.76 | Very good | (?) |
| Jenkinson (2007) [51] | 185 | The assessment of structural validity was based on CTT.  EFA considering a single factor estimated an eigenvalue greater than 1.2 and explained 43.1%. Factor loadings for items: Mobility = 0.76 / ADL = 0.72 / Emotional wellbeing = 0.76 / Social support = 0.72 / Cognition = 0.73 / Communication = 0.80 / Bodily discomfort = 0.61 / Stigma = 0.76 | Very good | (?) |
|  | 127 | The assessment of structural validity was based on CTT.  EFA considering a single factor estimated an eigenvalue greater than 1.2 and explained 45.95%. Factor loadings for items: Mobility = 0.80 / ADL = 0.73 / Emotional wellbeing = 0.65 / Social support = 0.75 / Cognition = 0.71 / Communication = 0.61 / Bodily discomfort = 0.51 / Stigma = 0.62 | Very good | (?) |
|  | 205 | The assessment of structural validity was based on CTT.  EFA considering a single factor estimated an eigenvalue greater than 1.2 and explained 53.57%. Factor loadings for items: Mobility = 0.82 / ADL = 0.79 / Emotional wellbeing = 0.73 / Social support = 0.75 / Cognition = 0.71 / Communication = 0.61 / Bodily discomfort = 0.51 / Stigma = 0.62 | Very good | (?) |
|  | 200 | The assessment of structural validity was based on CTT.  EFA considering a single factor estimated an eigenvalue greater than 1.2 and explained 41.76%. Factor loadings for items: Mobility = 0.78 / ADL = 0.80 / Emotional wellbeing = 0.49 / Social support = 0.61 / Cognition = 0.75 / Communication = 0.75 / Bodily discomfort = 0.36 / Stigma = 0.46 | Very good | (?) |
|  | 100 | The assessment of structural validity was based on CTT.  EFA considering a single factor estimated an eigenvalue greater than 1.2 and explained 37.16%. Factor loadings for items: Mobility = 0.63 / ADL = 0.67 / Emotional wellbeing = 0.75 / Social support = 0.61 / Cognition = 0.66 / Communication = 0.69 / Bodily discomfort = 0.27 / Stigma = 0.46 | Very good | (?) |
| Franchignoni (2008) [52] | – | – | – | – |
|  | – | – | – | – |
|  | 200 | The assessment of structural validity was based on IRT.  The Rasch model estimates for DIF [range] = (-0.19)-0.27. Difficulty estimates [Measure / SE / Infit MnSq / Outfit MnSq / Lower Threshold / Upper Threshold]: Mobility (-1.29 / 0.14 / 0.98 / 0.96 / -2.97 / 0.39). ADL (-0.69 / 0.14 / 1.21 / 1.19 / -2.37 / 0.99). Emotional wellbeing (-0.57 / 0.14 / 0.81 / 0.85 / -2.25 / 1.11). Stigma (0.02 / 0.14 / 01.15 / 1.13 / -1.66 / 1.70). Social support (2.26 / 0.19 / 1.16 / 1.06 / 0.58 / 3.94). Cognition (0.43 / 0.14 / 0.88 / 0.92 / -1.25 / 2.11). Communication (0.99 / 0.15 / 0.84 / 0.76 / -0.69 / 2.67) and Bodily discomfort (-0.74 / 0.14 / 0.99 / 1.02 / -2.42 / 0.94). Model fit statistics: Item Separation Index = 6.99 / Item Separation Reliability = 0.98 / Person Separation Index = 1.53 / Person Separation Reliability = 0.70 | Very good | (+) |
| Dal Bello-Haas (2009) [53] | – | – | – | – |
| Alvarado-Bolaños (2015) [54] | – | **PDQ-8:**  – | – | – |
|  | – | **EQ-5D-5L:**  – | – | – |
|  | – | **EQ-VAS:**  – | – | – |
| Kahraman (2018) [55] | – | – | – | – |
| Ramadhan (2022) [56] | – | **PDQ-8:**  – | – | – |
|  | – | **EQ-5D-3L:**  – | – | – |
| Stathis (2022) [57] | 60 | **PDQ-8:**  The assessment of structural validity was based on CTT.  EFA estimated that 2 factors explained 60% of the total variance (omega: hierarchical = 0.51 / total = 0.87) | Very good | (?) |
|  | 60 | **PDQoL-7:**  The assessment of structural validity was based on CTT.  EFA estimated that 3 factors explained 76% of the total variance (omega: hierarchical = 0.51 / total = 0.87) | Very good | (?) |
| Kawaguchi (2021) [58] | 54 | The assessment of structural validity was based on CTT.  EFA grouped items in 3 dimensions (total variance explained / factor contribution): 1) ADL (4.559 / 18.994); 2) Problems related with the therapy device (4.272 / 17.799); and 3) Psychological problems (4.343 / 18.094) | Very good | (?) |
| De Boer (1996) [59] | – | – | – | – |
| Serrano-Dueñas (2004) [60] | – | – | – | – |
| Campos (2011) [61] | – | – | – | – |
| Dereli (2015) [62] | – | – | – | – |
| Welsh (2003) [63] | 222 | The assessment of structural validity was based on CTT.  EFA estimated that 7 factors explained 55.6% of the total variance. Factor loadings (>0.35) ranged between 0.43-0.82 | Very good | (?) |
| Calne (1996) [64] | 147 | The assessment of structural validity was based on CTT.  EFA identified 4 factors [total variance explained (items included and eigenvalues)]: Factor 1 = 37% (Self = 0.932 / Leisure = 0.738 / Feelings = 0.726 / Safety = 0.613); Factor 2 = 26% (Family = 0.909 / Friends = 0.752 / Sexuality = 0.612); Factor 3 = 21% (Work = 0.846 / Travel = 0.675); Factor 4 = 16% (Financial security = 0.918) | Very good | (?) |
| Schulzer (2003) [65] | 116 | The assessment of structural validity was based on CTT.  EFA identified 4 factors explaining 78% of the total variance [items included and eigenvalues)]: Factor 1 (Work = 0.775 / Financial security = 0.798 / Leisure = 0.754 / Safety = 0.714 / Travel = 0.647); Factor 2 (Self = 0.843 / Feelings = 0.776); Factor 3 (Family = 0.830 / Friends = 0.772); Factor 4 (Sexuality = 0.911) | Very good | (?) |
| Aggarwal (2020) [66] | 295 | The assessment of structural validity was based on CTT.  EFA identified 68 items grouped in 8 factors explaining 80.3% of the variance (eigenvalue > 1). After Varimax Orthogonal Rotation, 41 items were grouped in 7 factors (variance explained): Motor (46%), Finances (10%), Fear and Social (6%), Psychological (5%), Nonmotor symptoms (4%), Treatment (3%) and Family (3%)  CFA confirmed that 45 items were grouped in 9 domains | Very good | (?) |
| Kuehler (2003) [67] | – | **QLSM-DBS:**  The assessment of structural validity was based on CTT.  EFA identified 5 factors (range of factor loadings): Reliability of the neurostimulator (0.70-0.85); Inconspicuousness of the neurostimulator (0.96-0.97); Independent handling/manipulation of the neurostimulator (0.72-0.93); Medical care (0.63-0.91); and Absence of bodily symptoms/side effects of the neurostimulation (0.59-0.82). | Very good | (?) |
|  | – | **QLSM-MD:**  The assessment of structural validity was based on CTT.  EFA identified 12 factors (range of factor loadings): Controllability/fluidity of movement (0.73-0.89); Absence of dizziness/steadiness when standing and walking (0.65-0.81); Hand dexterity throughout the day (0.73-0.87); Articulation/fluency of speech (0.93-0.94); Ability to swallow (single item); Absence of false bodily sensations (0.81); Bladder/intestinal function (0.76-0.77); Sexual excitability (0.54-0.89); Undisturbed sleep (0.86); Memory/clear thinking (0.60-0.73); Independence from help (0.54-0.94); Inconspicuousness of illness (0.75-0.87). | Very good | (?) |
| Krygowska-Wajs (2015) [68] | – | – | – | – |
| Bose (2018) [69] | 120 | The assessment of structural validity was based on CTT.  EFA grouped 55 items in 10 factors. | Very good | (?) |
| Diniz (2018) [70] | – | – | – | – |
| García-Gordillo (2013) [71] | – | **15D:**  – | – | – |
|  | – | **EQ-5D-5L:**  – | – | – |
| Del Pozo-Cruz (2018) [72] | – | **15D:**  – | – | – |
|  | – | **SF-6D:**  – | – | – |
| Luo (2009) [73] | – | **EQ-5D-3L**  – | – | – |
|  |  | **EQ-VAS:**  – | – | – |
|  | – | **EQ-5D-3L**  – | – | – |
|  |  | **EQ-VAS:**  – | – | – |
|  | – | **EQ-5D-3L**  – | – | – |
|  |  | **EQ-VAS:**  – | – | – |
| Garcia-Gordillo (2015) [74] | – | **EQ-5D-3L:**  – | – | – |
|  | – | **SF-6D:**  – | – | – |
| Nowinski (2010) [75] | – | – | – | – |
| Nowinski (2016) [76] | – | – | – | – |
| Kuspinar (2019) [77] | – | – | – | – |
| Kuspinar (2020) [78] | – | – | – | – |
| Hagell (2011) [79] | 150 | The assessment of structural validity was based on IRT.  A Rasch model was designed with the original two components from the SF-12 (Physical Component Score [PCS-12] and Mental Component Score [MCS-12]) and. subsequently. a revised version was generated (SF-10). Global fit statistics of the model for items [residuals – mean (SD)]: Original (PCS-12) = -0.34 (1.43) / Original (MCS-12) = -0.06 (1.01) / Original (SF-12) = 0.05 (1.18) / Revised (PCS-12) = -0.19 (1.15) / Revised (MCS-12) = 0.12 (1.11) / Revised (SF-10) = 0.20 (0.64). Global fit statistics of the model for participants [residuals – mean (SD)]: Original (PCS-12) = -0.40 (0.83) / Original (MCS-12) = -0.29 (0.97) / Original (SF-12) = -0.25 (1.12) / Revised (PCS-12) = -0.38 (0.85) / Revised (MCS-12) = -0.28 (0.93) / Revised (SF-10) = -0.23 (1.08). Global fit statistics of the model for item-treat interaction [Chi-Squared (D.F.; p-Value)]: Original (PCS-12) = 29.57 (12; 0.003) / Original (MCS-12) = 16.32 (12; 0.177) / Original (SF-12) = 38.56 (24; 0.0304) / Revised (PCS-12) = 10.63 (10; 0.387) / Revised (MCS-12) = 9.75 (10; 0.462) / Revised (SF-10) = 19.08 (20; 0.516). Reliability [Person Separation Index (PSI)]: Original (PCS-12) = 0.85 / Original (MCS-12) = 0.85 / Original (SF-12) = 0.89 / Revised (PCS-12) = 0.82 / Revised (MCS-12) = 0.82 / Revised (SF-10) = 0.87. Targeting [participant location – mean (SD)]: Original (PCS-12) = -0.88 (2.20) / Original (MCS-12) = 0.44 (1.47) / Original (SF-12) = -0.14 (1.44) / Revised (PCS-12) = 0.78 (2.05) / Revised (MCS-12) = 0.55 (1.37) / Revised (SF-10) = -0.05 (1.37). One-dimensionality [eigenvalue (% of the total variance explained)]: Original (PCS-12) = 1.81 (30.2%) / Original (MCS-12) = 1.59 (26.6%) / Original (SF-12) = 2.67 (22.2%) / Revised (PCS-12) = 1.80 (36.0%) / Revised (MCS-12) = 1.57 (31.6%) / Revised (SF-10) = 2.39 (23.9%). One-dimensionality [% (95CI)]: Original (PCS-12) = 4.8 (1.3-8.4) / Original (MCS-12) = 4.9 (1.3-8.5) / Original (SF-12) = 11.3 (7.8-14.8) / Revised (PCS-12) = 4.8 (1.3-8.4) / Revised (MCS-12) = 2.8 (-0.8-6.4) / Revised (SF-10) = 8.0 (4.5-11.5) | Very good | (?) |
| Steffen (2008) [80] | – | – | – | – |
| Hagell (2008) [81] | 202 | The assessment of structural validity was based on CTT.  CFA confirmed 8 factors (Kaiser-Meyer-Olkin = 0,89 / Bartlett’s sfericity test: Chi-Cuadrado = 775-35 [p < 0,0001] / Total variance explained = 68.43%):   - Physical functioning: Eigenvalue* = 0.72 / Loading PCS = 0.71 / Loading MCS = 0.34 / Total Reliable Variance = 0.66 - Role physical: Eigenvalue* = 0.80 / Loading PCS = 0.82 / Loading MCS = 0.34 / Total Reliable Variance = 0.91 - Pain: Eigenvalue* = 0.67 / Loading PCS = 0.17 / Loading MCS = 0.74 / Total Reliable Variance = 0.64 - General health: Eigenvalue* = 0.77 / Loading PCS = 0.34 / Loading MCS = 0.73 / Total Reliable Variance = 0.82 - Energy: Eigenvalue* = 0.87 / Loading PCS = 0.45 / Loading MCS = 0.76 / Total Reliable Variance = 0.92 - Social functioning: Eigenvalue* = 0.79 / Loading PCS = 0.46 / Loading MCS = 0.65 / Total Reliable Variance = 0.81 - Role emotional: Eigenvalue* = 0.72 / Loading PCS = 0.81 / Loading MCS = 0.24 / Total Reliable Variance = 0.82 - Mental health: Eigenvalue* = 0.78 / Loading PCS = 0.29 / Loading MCS = 0.79 / Total Reliable Variance = 0.86   Model fit statistics: CFI = 0.82 / RMSEA = 0.21 (95CI = 0.18-0.24) / Chi-squared = 155.89 [p < 0.0001] / GFI = 0.87 / Adjusted GFI = 0.72. | Very good | (+) |
| Schneider (2010) [82] | – | – | – | – |
| Hendred (2016) [83] | – | – | – | – |
