# Supplementary material for: Quality of life measures in Parkinson’s disease: a systematic literature review of patient-reported outcomes measures (PROMs) and their psychometric properties
Source: J Neurol. 2025 Aug 28;272(9):598. doi: 10.1007/s00415-025-13348-x (PMC12394374; doi:10.1007/s00415-025-13348-x)
Supplement: Supplementary file 8 — Supplementary file8 (DOCX 152 KB) [file 415_2025_13348_MOESM8_ESM.docx]

**Quality of Life Measures in Parkinson’s Disease: A Systematic Literature Review of Patient-Reported Outcomes Measures (PROMs) and their Psychometric Properties**

**– ONLINE RESOURCE 6 –**

Table S12. Descriptions of the studies’ findings in relation to the Know-Group Validity of the PROMs.

| Eligible study | Sample size | Findings of the study in relation to Know-Group Validity | COSMIN assessment | |
| --- | --- | --- | --- | --- |
|  |  |  | **RoB** | **Good property** |
| Spliethoff-Kamminga (2003) [1] | 54 | ANOVA test comparing Bela-P-K scores of patients with PD grouped by H&YS did not find statistically significant differences | Very good | (–) |
| Ortelli (2017) [2] | 202 | ANOVA test comparing Bela-P-K Bb scores of patients with PD grouped by H&YS [mean (SD)]:   - Total: H&YS II = 21.0 (17.0) / III = 22.3 (16.1) / IV = 29.1 (18.9) [p = 0.33] - Achievement capability / Physical symptoms: H&YS II = 5.8 (4.7) / III = 6.7 (4.7) / IV = 9.4 (4.8) [p = 0.056] - Fear / Emotional symptoms: H&YS II = 6.1 (4.3) / III = 5.9 (4.5) / IV = 6.7 (4.9) [p = 0.84] - Social functioning: H&YS II = 4.6 (5.3) / III = 5.2 (5.2) / IV = 6.5 (5.5) [p = 0.55] - Partner-bonding / Family: H&YS II = 4.3 (4.8) / III = 4.6 (4.8) / IV = 6.5 (5.6) [p = 0.38]   ANOVA test comparing Bela-P-K Nfh scores of patients with PD grouped by H&YS [mean (SD)]:   - Total: H&YS II = 18.3 (17.4) / III = 19.7 (15.0) / IV = 28.1 (18.1) [p = 0.18] - Achievement capability / Physical symptoms: H&YS II = 5.2 (5.0) / III = 6.0 (4.7) / IV = 9.5 (4.5) [p = 0.022] - Fear / Emotional symptoms: H&YS II = 5.1 (4.2) / III = 5.0 (4.2) / IV = 6.9 (5.4) [p = 0.38] - Social functioning: H&YS II = 4.1 (5.3) / III = 4.6 (4.7) / IV = 5.8 (5.8) [p = 0.56] - Partner-bonding / Family: H&YS II = 3.9 (4.9) / III = 4.1 (4.6) / IV = 5.9 (5.4) [p = 0.42] | Very good | (–) |
| Bayen (2021) [3] | – | – | – | – |
| Aggarwal (2013) [4] | – | – | – | – |
| Kuharic (2022) [5] | 569 | Comparison of OFFELIA scores of patients with PD grouped by time per Off episode (hours) identified differences (no more data available) | Doubtful | (?) |
| Kuharic (2024) [6] | 569 | Comparison of OFFELIA scores of patients with PD grouped by:   - Time per Off episode (hours): PDQ-8 achieved greater size of effect for groups of 2 hours (0.08 vs. 0.07) and >4 hours (0.20 vs. 0.13) - Severity of disease: Discriminant ability of OFFELIA was greater than EQ-5D-5L and PDQ-8 for unpredictability of the Off episodes (F-Ratio = 2.44 vs 2.55), duration of the Off episode (F-Ratio = 2.49 vs. 3.33) and time since PD onset (F-Ratio = 3.18 vs. 2.04) | Very good | (+) |
| Peto (1995) [7] | – | T-Student test comparing PDQ-39 scores of patients with PD grouped by sex [mean (SD); male / female]:   - Mobility: 60.49 (30.94) / 69.69 (24.83) [p < 0.005] - Cognition: 53.38 (22.48) / 44.18 (19.66) [p < 0.001] - Communication: 43.76 (27.09) / 27.20 (24.03) [p < 0.001] - Bodily discomfort: 51.50 (27.62) / 59.46 (22.81) [p < 0.01]   No significant differences observed for the remaining dimensions | Doubtful | (?) |
|  | 227 | Kruskall-Wallis test comparing PDQ-39 scores of patients with PD grouped by severity of tremor [mean / median not specified; mild / moderate / severe]:   - Mobility: 57.9 / 67.4 / 79.6 [p < 0.001] - ADL: 45.4 / 54.8 / 74.0 [p < 0.0001] - Emotional wellbeing: 36.7 / 44.0 / 50.6 [p < 0.05] - Stigma: 24.5 / 33.4 / 52.7 [p < 0.0001] - Social support: 21.2 / 23.4 / 30.7 [p = 0.1667] - Cognition: 40.2 / 49.3 / 55.2 [p < 0.01] - Communication: 34.4 / 34.8 / 45.4 [p = 0.0773] - Bodily discomfort: 42.6 / 53.3 / 66.3 [p < 0.0001]   Kruskall-Wallis test comparing PDQ-39 scores of patients with PD grouped by severity of stiffness [mean / median not specified; mild / moderate / severe]:   - Mobility: 46.5 / 63.9 / 79.6 [p < 0.0001] - ADL: 3.8 / 56.8 / 65.8 [p < 0.0001] - Emotional wellbeing: 33.1 / 39.7 / 52.5 [p < 0.001] - Stigma: 22.3 / 30.5 / 45.1 [p < 0.001] - Social support: 16.7 / 23.1 / 29.3 [p = 0.1099] - Cognition: 35.3 / 44.1 / 57.5 [p < 0.0001] - Communication: 25.6 / 35.4 / 44.7 [p < 0.001] - Bodily discomfort: 35.0 / 49.2 / 64.9 [p < 0.0001]   Kruskall-Wallis test comparing PDQ-39 scores of patients with PD grouped by severity of slowness [mean / median not specified; mild / moderate / severe]:   - Mobility: 36.7 / 61.6 / 82.9 [p < 0.0001] - ADL: 29.0 / 53.1 / 68.9 [p < 0.0001] - Emotional wellbeing: 26.3 / 41.4 / 51.0 [p < 0.0001] - Stigma: 20.6 / 31.6 / 42.6 [p < 0.001] - Social support:16.4 / 21.6 / 29.7 [p = 0.695] - Cognition: 36.7 / 42.4 / 57.1 [p < 0.0001] - Communication: 24.5 / 33.9 / 45.1 [p < 0.001] - Bodily discomfort: 35.1 / 48.3 / 62.3 [p < 0.0001] | Doubtful | (?) |
| Jenkinson (1997) [8] | – | – | – | – |
|  | – | – | – | – |
| Jenkinson (1997) [9] | – | **PDQ-39:**  – | – | – |
|  | 135 | **PDQ-8:**  PDQ-8 scores of patients with PD grouped by H&YS [mean (SD)]: Stage 1 = 17.74 (16.27) / Stage 2 = 33.14 (18.80) / Stage 3 = 37.05 (22.05) / Stage 4 = 47.86 (16.17) | Very good | (?) |
| Martínez-Martín (1998) [10] | 103 | Kruskall-Wallis test comparing PDQ-39 scores of patients with PD grouped by H&YS:   - Moiblity: Stage 1 = 13.40 (19.40) / Stage 2 = 29.64 (27.48) / Stage 3 = 57.14 (18.14) / Stage 4 = 81.13 (10.45) [p = 0.001] - ADL: Stage 1 = 9.22 (10.86) / Stage 2 = 32.03 (24.48) / Stage 3 = 57.59 (20.32) / Stage 4 = 64.77 (13.86) [p = 0.001] - Emotional wellbeing: Stage 1 = 19.64 (20.17) / Stage 2 = 31.33 (23.88) / Stage 3 = 48.36 (24.60) / Stage 4 = 55.68 (24.81) [p = 0.001] - Stigma: Stage 1 = 5.80 (8.30) / Stage 2 = 20.75 (26.65) / Stage 3 = 31.25 (29.46) / Stage 4 = 30.68 (27.02) [p = 0.05] - Social support: Stage 1 = 3.57 (9.64) / Stage 2 = 15.27 (24.87) / Stage 3 = 20.37 (23.94) / Stage 4 = 26.51 (26.82) [p = 0.05] - Cognition: Stage 1 = 11.16 (9.23) / Stage 2 = 24.94 (18.56) / Stage 3 = 28.57 (25.08) / Stage 4 = 36.93 (18.63) [p = 0.05] - Communication: Stage 1 = 2.97 (5.27) / Stage 2 = 17.00 (17.73) / Stage 3 = 33.03 (25.01) / Stage 4 = 37.87 (21.52) [p = 0.001] - Bodily discomfort: Stage 1 = 18.45 (26.80) / Stage 2 = 37.16 (23.94) / Stage 3 = 43.75 (20.49) / Stage 4 = 44.69 (32.76) [p = 0.01] | Very good | (+) |
| Bushnell (1999) [11] | 75 | Kruskall-Wallis test comparing PDQ-39 scores of patients with PD grouped by severity of tremor [mean; mild / moderate / severe]:   - Mobility: 37 / 40 / 62 [p = 0.05] - ADL: 35 / 40 / 62 [p < 0.05] - Emotional wellbeing: 28 / 38 / 60 [p < 0.01] - Stigma: 22 / 28 / 51 [p < 0.05] - Social support: 22 / 21 / 46 [p < 0.01] - Cognition: 30 / 31 / 57 [p < 0.05] - Communication: 28 / 36 / 57 [p < 0.05] - Bodily discomfort: 36 / 38 / 63 [p < 0.01]   Kruskall-Wallis test comparing PDQ-39 scores of patients with PD grouped by severity of stiffness [mean; mild / moderate / severe]:   - Mobility: 23 / 44 / 70 [p < 0.001] - ADL: 26 / 45 / 66 [p < 0.001] - Emotional wellbeing: 27 / 37 / 56 [p < 0.01] - Stigma: 23 / 27 / 45 [p < 0.08] - Social support: 24 / 22 / 34 [p = 0.31] - Cognition: 26 / 36 / 51 [p < 0.05] - Communication: 29 / 33 / 53 [p < 0.05] - Bodily discomfort: 29 / 47 / 55 [p < 0.01]   Kruskall-Wallis test comparing PDQ-39 scores of patients with PD grouped by severity of slowness [mean; mild / moderate / severe]:   - Mobility: 21 / 46 / 81 [p < 0.0001] - ADL: 23 / 49 / 75 [p < 0.0001] - Emotional wellbeing: 27 / 38 / 61 [p < 0.0001] - Stigma: 23 / 27 / 45 [p = 0.06] - Social support: 24 / 22 / 32 [p = 0.33] - Cognition: 25 / 42 / 48 [p < 0.01] - Communication: 27 / 36 / 56 [p < 0.01] - Bodily discomfort: 30 / 46 / 60 [p < 0.001]   Kruskall-Wallis test comparing PDQ-39 scores of patients with PD grouped by severity of freezing [mean; mild / moderate / severe]:   - Mobility: 27 / 63 / 78 [p < 0.0001] - ADL: 29 / 63 / 75 [p < 0.0001] - Emotional wellbeing: 32 / 43 / 67 [p < 0.0001] - Stigma: 24 / 31 / 48 [p = 0.06] - Social support: 25 / 22 / 30 [p = 0.68] - Cognition: 27 / 44 / 62 [p < 0.01] - Communication: 27 / 49 / 61 [p < 0.0001] - Bodily discomfort: 36 / 48 / 59 [p < 0.05]   Kruskall-Wallis test comparing PDQ-39 scores of patients with PD grouped by severity of jerking [mean; mild / moderate / severe]:   - Mobility: 33 / 53 / 64 [p < 0.05] - ADL: 35 / 52 / 72 [p < 0.05] - Emotional wellbeing: 32 / 45 / 65 [p < 0.01] - Stigma: 22 / 44 / 60 [p < 0.01] - Social support: 24 / 23 / 35 [p = 0.46] - Cognition: 29 / 45 / 64 [p < 0.01] - Communication: 29 / 47 / 70 [p < 0.001] - Bodily discomfort: 38 / 42 / 68 [p < 0.05] | Very good | (+) |
| Andreu (2000) [12] | 126 | ANOVA test comparing PDQ-39 scores of patients with PD grouped by H&YS [mean (SD)]:   - Mobility: Stage 1 = 20.5 (13.9) / Stage 2 = 26.0 (19.5) / Stage 3 = 32.9 (17.9) / Stages 4+5 = 67.4 (13.6) - ADL: Stage 1 = 23.3 (17.3) / Stage 2 = 28.1 (20.4) / Stage 3 = 33.1 (19.8) / Stages 4+5 = 65.3 (18.1) - Emotional wellbeing: Stage 1 = 32.0 (19.6) / Stage 2 = 34.9 (21.2) / Stage 3 = 32.1 (17.9) / Stages 4+5 = 54.1 (19.3) - Communication: Stage 1 = 17.5 (16.1) / Stage 2 = 27.1 (18.7) / Stage 3 = 31.4 (17.1) / Stages 4+5 = 52.1 (16.6) - Bodily discomfort: Stage 1 = 25.1 (15.1) / Stage 2 = 29.4 (13.1) / Stage 3 = 31.6 (12.2) / Stages 4+5 = 49.8 (9.7) - PDQ-39 SI: Stage 1 = 25.1 (15.1) / Stage 2 = 29.4 (13.1) / Stage 3 = 31.6 (12.1) / Stages 4+5 = 49.8 (9.7)   ANOVA test comparing PDQ-39 scores of patients with PD grouped by severity of depression measured by MADRS [mean (SD)]:   - Mobilty: I (No depression) = 28.9 (22.3) / II (Mild depression) = 42.2 (19.9) / III (Severe depression) = 47.0 (27.8) - ADL: I (No depression) = 28.9 (20.4) / II (Mild depression) = 44.1 (23.9) / III (Severe depression) = 51.6 (30.3) - Emotional wellbeing: I (No depression) = 26.4 (16.1) / II (Mild depression) = 51.1 (13.6) / III (Severe depression) = 68.6 (16.3) - Stigma: I (No depression) = 30.9 (24.0) / II (Mild depression) = 45.8 (19.9) / III (Severe depression) = 67.4 (18.8) - Social support: I (No depression) = 10.2 (15.0) / II (Mild depression) = 19.7 (16.8) / III (Severe depression) = 36.5 (31.0) - Communication: I (No depression) = 23.9 (16.6) / II (Mild depression) = 42.6 (18.9) / III (Severe depression) = 49.1 (21.5) - PDQ-39 SI: I (No depression) = 26.6 (12.6) / II (Mild depression) = 41.5 (11.1) / III (Severe depression) = 50.7 (14.4)   No significant differences observed for the remaining dimensions. | Very good | (+) |
| Schrag (2000) [13] | 97 | **PDQ-39:**  U Mann-Whitney / Kruskall-Wallis test comparing PDQ-39 scores of patients with PD grouped by [mean]:   - Depression: Present = 39.0 / Absent = 16.7 [p < 0.0001] - MMSE < 25: Present = 49.4 / Absent = 24.4 [p < 0.0001] - Falls: Present = 36.2 / Absent = 21.4 [p < 0.0001] - Postural instability: Present = 33.7 / Absent = 18.7 [p = 0.0001] - Perception of health changes compared with the previous year: Improvement = 16.8 / No change = 25.1 / Worsening = 43.4 [p {Improvement vs. No change} = 0.2 / p {No change vs. Worsening} = 0.0001 / p {All} = 0.0002] - H&YS: Stage 1 = 16.6 / Stage 2 = 28.1 / Stage 3 = 40.8 / Stage 4+5 = 38.6 [p = 0.0001] | Very good | (+) |
|  | 97 | **EQ-5D-3L:**  U Mann-Whitney / Kruskall-Wallis test comparing EQ-5D-3L utility values of patients with PD grouped by [mean]:   - Depression: Present = 0.4 / Absent = 0.7 [p < 0.0001] - MMSE < 25: Present = 0.2 / Absent = 0.7 [p < 0.0001] - Falls: Present = 0.4 / Absent = 0.8 [p < 0.0001] - Postural instability: Present = 0.4 / Absent = 0.8 [p < 0.0001] - Perception of health changes compared with the previous year: Improvement = 0.6 / No change = 0.6 / Worsening = 0.3 [p {Improvement vs. No change} = 0.7 / p {No change vs. Worsening} = < 0.0001 / p {All} = 0.0002] - H&YS: Stage 1 = 0.9 / Stage 2 = 0.6 / Stage 3 = 0.3 / Stage 4+5 = 0.2 [p = 0.0001] | Very good | (+) |
|  | 97 | **EQ-VAS:**  U Mann-Whitney / Kruskall-Wallis test comparing EQ-VAS scores of patients with PD grouped by [mean]:   - Depression: Present = 54.9 / Absent = 76.7 [p < 0.0001] - MMSE < 25: Present = 51.1 / Absent = 69.0 [p = 0.007] - Falls: Present = 58.6 / Absent = 71.7 [p = 0.006] - Postural instability: Present = 57.2 / Absent = 77.6 [p < 0.0001] - Perception of health changes compared with the previous year: Improvement = 70.5 / No change = 70.9 / Worsening = 49.1 [p {Improvement vs. No change} = 0.9 / p {No change vs. Worsening} < 0.0001 / p {All} = 0.0001] - H&YS: Stage 1 = 78.5 / Stage 2 = 66.8 / Stage 3 = 53.8 / Stage 4+5 = 52.0 [p = 0.02] | Very good | (+) |
|  | 97 | **SF-36:**  U Mann-Whitney / Kruskall-Wallis test comparing SF-36 (Physical Composite Score – PCS) scores of patients with PD grouped by [mean]:   - Depression: Present = 31.3 / Absent = 40.3 [p = 0.0001] - MMSE < 25: Present = 29.7 / Absent = 37.2 [p = 0.05] - Falls: Present = 31.4 / Absent = 39.8 [p = 0.006] - Postural instability: Present = 32.2 / Absent = 42.3 [p = 0.0007] - Perception of health changes compared with the previous year: Improvement = 36.2 / No change = 37.0 / Worsening = 30.0 [p {Improvement vs. No change} = 0.8 / p {No change vs. Worsening} = 0.003 / p {All} = 0.01] - H&YS: Stage 1 = 46.2 / Stage 2 = 35.5 / Stage 3 = 28.2 / Stages 4+5 = 22.0 [p = 0.005]   U Mann-Whitney / Kruskall-Wallis test comparing SF-36 (Mental Composite Score – MCS) scores of patients with PD grouped by [mean]:   - Depression: Present = 43.6 / Absent = 52.0 [p < 0.0001] - MMSE < 25: Present = 40.9 / Absent = 49.5 [p = 0.006] - Falls: Present = 46.2 / Absent = 48.5 [p = 0.3] - Postural instability: Present = 47.8 / Absent = 46.3 [p = 0.7] - Perception of health changes compared with the previous year: Improvement = 53.4 / No change = 48.0 / Worsening = 43.3 [p {Improvement vs. No change} = 0.2 / p {No change vs. Worsening} = 0.1 / p {All} = 0.1] - H&YS: I = 49.5 / II = 48.5 / III = 43.9 / IV-V = 35.3 [p = 0.05] | Very good | (+) |
| Katsarou (2001) [14] |  | Kruskall-Wallis test comparing PDQ-39 scores of patients with PD grouped by H&YS [mean (SD)]:   - Movilidad: Stage 1= 10.42 (15.28) / Estadio II = 16.75 (18.45) / Stage 3 = 30.57 (17.03) / Stage 4 = 60.50 (18.61) [p < 0.001] - Act. Cotidianas: Stage 1= 23.61 (16.81) / Estadio II = 29.72 (16.65) / Stage 3 = 46.19 (19.68) / Stage 4 = 56.33 (23.45) [p < 0.001] - Comunicación: Stage 1= 13.89 (18.76) / Estadio II = 13.52 (15.95) / Stage 3 = 24.05 (19.15) / Stage 4 = 34.67 (21.74) [p < 0.001] - Discomfort corporal: Stage 1= 16.67 (13.94) / Estadio II = 20.91 (23.03) / Stage 3 = 30.95 (18.70) / Stage 4 = 34.00 (22.17) [p < 0.05]   No significant differences observed for the remaining dimensions. | Very good | (+) |
| Peto (2001) [15] | – | – | – | – |
| Tsang (2002) [16] | – | – | – | – |
| Hagell (2003) [17] | – | – | – | – |
| Jenkinson (2003) [18] | – | – | – | – |
|  | – | – | – | – |
|  | – | – | – | – |
|  | – | – | – | – |
|  | – | – | – | – |
| Park (2004) [19] | 24 | Differences in PDQ-39 scores were found when comparing patients with PD vs. healthy controls, for Mobility, ADL, Emotional wellbeing and Stigma | Very good | (?) |
| Tan (2004) [20] | – | **PDQ-39:**  – | – | – |
|  | – | **PDQ-8:**  – | – | – |
| Fitzpatrick (2004) [21] | – | – | – | – |
|  | – | – | – | – |
| Haapaniemi (2004) [22] | 259 | U Mann-Whitney test comparing PDQ-39 scores in patients with PD grouped by {mean (SD) [p-Value]}:   - HH&YS: <3 = 26.7 (13.7) / ≥3 = 46.2 (13.0) [p < 0.001] - Motor fluctuations: Absent = 27.3 (14.7) / Present = 41.3 (14.4) [p < 0.001] - Dementia: Absent = 29.8 (15.6) / Present = 46.3 (9.8) [p < 0.001] - Depression: Absent = 27.9 (15.6) / Present = 39.7 (13.5) [p < 0.001] | Very good | (+) |
|  | 259 | U Mann-Whitney test comparing healthy participants (control) vs. patients with PD {mean (SD) [p-Value]}:   - 15D Utility: Parkinson = 0.7 (0.13) / Control = 0.86 (0.12) [p < 0.001] - Mobility: Parkinson = 0.78 (0.20) / Control = 0.86 (0.20) [p < 0.001] - Vision: Parkinson = 0.88 (0.18) / Control = 0.90 (0.19) [p = 0.020] - Earing: Parkinson = 0.92 (0.15) / Control = 0.90 (0.17) [p = 0.451] - Breathing: Parkinson = 0.80 (0.23) / Control = 0.84 (0.23) [p < 0.001] - Sleeping: Parkinson = 0.77 (0.21) / Control = 0.84 (0.20) [p < 0.001] - Eating: Parkinson = 0.85 (0.19) / Control = 0.99 (0.08) [p < 0.001] - Speech: Parkinson = 0.80 (0.20) / Control = 0.97 (0.10) [p < 0.001] - Elimination: Parkinson = 0.74 (0.22) / Control = 0.86 (0.21) [p < 0.001] - Usual activities: Parkinson = 0.69 (0.22) / Control = 0.80 (0.24) [p < 0.001] - Mental function: Parkinson = 0.76 (0.21) / Control = 0.85 (0.19) [p < 0.001] - Discomfort and symptoms: Parkinson = 0.70 (0.21) / Control = 0.74 (0.24) [p = 0.007] - Depression: Parkinson = 0.82 (0.17) / Control = 0.88 (0.16) [p < 0.001] - Distress: Parkinson = 0.79 (0.18) / Control = 0.88 (0.16) [p < 0.001] - Vitality: Parkinson = 0.73 (0.17) / Control = 0.82 (0.18) [p < 0.001] - Sexual activity: Parkinson = 0.65 (0.30) / Control = 0.78 (0.30) [p < 0.001]   U Mann-Whitney test comparing 15D utility values in participants with PD grouped by {mean (SD) [p-Value]}:   - H&YS: <3 = 0.81 (0.1) / ≥3 = 0.65 (0.1) [p < 0.001] - Motor fluctuations: Absent = 0.80 (0.1) / Present = 0.70 (0.1) [p < 0.001] - Dementia: Absent = 0.78 (0.1) / Present = 0.65 (0.1) [p < 0.001] - Depression: Absent = 0.81 (0.1) / Present = 0.68 (0.1) [p < 0.001] | Very good | (+) |
| Martínez-Martín (2004) [23] | – | – | – | – |
| Ma (2005) [24] | 73 | ANOVA test comparing PDQ-39 scores in patients with PD grouped by H&YS [mean (SD)]:   - Mobility: Stage 1 = 7.88 (12.11) / Stage 2 = 21.01 (21.90) / Stage 3 = 65.00 (20.75) / Stages 4+5 = 82.11 (23.91) [p < 0.0001] - ADL: Stage 1 = 5.13 (6.17) / Stage 2 = 16.22 (21.17) / Stage 3 = 23.75 (12.89) / Stages 4+5 = 57.69 (31.68) [p < 0.0001] - Emotional wellbeing: Stage 1 = 15.71 (13.52) / Stage 2 = 22.41 (21.39) / Stage 3 = 35.42 (18.66) / Stages 4+5 = 55.77 (25.83) [p < 0.0001] - Stigma: Stage 1 = 10.10 (15.84) / Stage 2 = 25.51 (29.48) / Stage 3 = 40.63 (32.31) / Stages 4+5 = 50.00 (30.30) [p = 0.0029] - Social support: Stage 1 = 6.09 (10.84) / Stage 2 = 9.35 (17.61) / Stage 3 = 21.25 (23.93) / Stages 4+5 = 23.72 (22.78) [p = 0.0310] - Cognition: Stage 1 = 16.83 (12.07) / Stage 2 = 27.36 (18.06) / Stage 3 = 25.00 (25.46) / Stages 4+5 = 41.67 (28.05) [p < 0.0001] - Communication: Stage 1 = 7.05 (10.11) / Stage 2 = 14.41 (23.38) / Stage 3 = 25.00 (25.46) / Stages 4+5 = 41.67 (18.27) [p = 0.0009]   No significant differences were observed for the remaining dimensions. | Very good | (+) |
| Luo (2005) [25] | – | **PDQ-39:**  – | – | – |
|  | – | **PDQ-8:**  – | – | – |
| Martínez-Martín (2007) [26] | – | **PDQ-39:**  – | – | – |
|  | 188 | **PDQL:**  Spearman correlations of PDQL (Parkinsonian symptoms / Systemic symptoms / Emotional functioning / Social functioning) with H&YS [p < 0.05] = -0.61 / -0.61 / -0.59 / -0.29 / -0.56  Spearman correlations of PDQL with PDQ-39, duration of PD (years), duration of the treatment with levodopa (years), S&E, UPDRS-Mentation, UPDRS-ADL, UPDRS-Motor, HADS-A, HADS-D. | Very good | (+) |
| Hagell (2007) [27] | – | – | – | – |
| Krikmann (2008) [28] | – | – | – | – |
| Marinus (2008) [29] | 177 | ANOVA test comparing PDQ-39 scores in patients with PD grouped by H&YS [mean (SD)]:   - Mobility: Mild (Stage 1+2) = 31 / Moderate (Stage 3) = 48 / Severe (Stage 4+5) = 68 [p < 0.001] - ADL: Mild (Stage 1+2) = 37 / Moderate (Stage 3) = 48 / Severe (Stage 4+5) = 61 [p < 0.001] - Emotional wellbeing: Mild (Stage 1+2) = 32 / Moderate (Stage 3) = 40 / Severe (Stage 4+5) = 43 [p = 0.010] - Social support: Mild (Stage 1+2) = 16 / Moderate (Stage 3) = 21 / Severe (Stage 4+5) = 26 [p = 0.046] - Cognition: Mild (Stage 1+2) = 31 / Moderate (Stage 3) = 39 / Severe (Stage 4+5) = 44 [p = 0.007] - Communication: Mild (Stage 1+2) = 25 / Moderate (Stage 3) = 35 / Severe (Stage 4+5) = 42 [p < 0.001] - Bodily discomfort: Mild (Stage 1+2) = 41 / Moderate (Stage 3) = 49 / Severe (Stage 4+5) = 55 [p = 0.005] - PDQ-39 SI: Mild (Stage 1+2) = 31 / Moderate (Stage 3) = 41 / Severe (Stage 4+5) = 51 [p < 0.001]   No significant differences observed for Stigma. | Very good | (+) |
| Serrano-Dueñas (2008) [30] | 131 | **PDQ-39:**  Kruskall-Wallis test comparing PDQ-39 scores in patients with PD grouped by H&YS [mean (SD)]: Stage 1.5 = 32.7 (31.9) / Stage 2 = 30.5 (13.4) / Stage 2.5 = 41.7 (16.5) / Stage 3 = 60.6 (23.6) / Stages 4 + 5 = 109 (10.1) [p < 0.0001] | Very good | (+) |
|  | 131 | **PDQL:**  Kruskall-Wallis / T-Student test comparing PDQL scores in patients with PD grouped by H&YS [mean (SD)]: Stage 1.5 = 121 (33.9) / Stage 2 = 140.8 (15.9) / Stage 2.5 = 105.2 (28.5) / Stage 3 = 103 (20) / Stages 4+5 = 58.6 (10.6) [p < 0.0001] | Very good | (+) |
|  | 131 | **PIMS:**  Kruskall-Wallis / U Mann Whitney test comparing PIMS scores in patients with PD grouped by:   - H&YS: Stage 1.5 = 14.3 (11) / Stage 2 = 13 (6.2) / Stage 2.5 = 17.8 (5.1) / Stage 3 = 22.2 (5.6) / Stages 4+5 = 31.2 (4.4) [p < 0.0001] - Fluctuations: Fluctuators = 25.3 (5.8) / No fluctuators = 18.4 (7.4) [6.9; 95CI = 4.19 - 9.65] | Very good | (+) |
| Žiropađa (2009) [31] | 102 | ANOVA test comparing PDQ-39 scores in patients with PD grouped by H&YS [mean (SD)]:   - Mobility: Stage 1 = 31.7 (7.7) / Stage 2 = 48.5 (13.1) / Stages 3+4 = 66.0 (17.6) [p = 0.001] - ADL: Stage 1 = 43.8 (15.1) / Stage 2 = 63.0 (12.6) / Stages 3+4 = 71.1 (11.9) [p = 0.001] - Emotional wellbeing: Stage 1 = 39.9 (19.2) / Stage 2 = 48.1 (19.2) / Stages 3+4 = 45.6 (17.3) [p > 0.05] - Stigma: Stage 1 = 38.0 (16.9) / Stage 2 = 48.6 (19.2) / Stages 3+4 = 45.8 (17.3) [p = 0.03] - Social support: Stage 1 = 41.6 (17.2) / Stage 2 = 50.2 (17.4) / Stages 3+4 = 55.0 (12.8) [p = 0.001] - Cognition: Stage 1 = 35.6 (12.1) / Stage 2 = 47.3 (11.8) / Stages 3+4 = 47.5 (16.1) [p = 0.001] - Communication: Stage 1 = 27.9 (8.7) / Stage 2 = 39.5 (13.9) / Stages 3+4 = 49.1 (15.5) [p = 0.001] - Bodily discomfort: Stage 1 = 41.7 (20.4) / Stage 2 = 54.9 (12.4) / Stages 3+4 = 56.3 (20.1) [p = 0.001] - PDQ-39 SI: 37.6 (10.3) / Stage 2 = 49.9 (12.4) / Stages 3+4 = 54.6 (11.8) [p = 0.001] | Very good | (+) |
| Nojomi (2010) [32] | 200 | ANOVA test comparing PDQ-39 scores in patients with PD grouped by H&YS (severity of PD) [mean (SD)]:   - Mobility: Mild = 31.7 (19.2) / Moderate = 53.1 (20.7) / Severe = 65.5 (16.4) [p = 0.0005] - ADL: Mild = 28.3 (18.9) / Moderate = 46.8 (20.0) / Severe = 55.9 (22.8) [p = 0.0005] - Emotional wellbeing: Mild = 32.2 (19.2) / Moderate = 38.7 (19.8) / Severe = 53.2 (23.8) [p = 0.0005] - Stigma: Mild = 28.3 (23.1) / Moderate = 39.3 (24.6) / Severe = 42.3 (28.2) [p = 0.004] - Social support: Mild = 18.8 (17.9) / Moderate = 25.2 (19.5) / Severe = 34.4 (27.7) [p = 0.005] - Cognition: Mild = 23.1 (14.9) / Moderate = 32.8 (16.3) / Severe = 37.5 (19.4) [p = 0.005] - Communication: Mild = 22.4 (19.0) / Moderate = 34.0 (21.0) / Severe = 34.9 (22.2) [p = 0.005] - Bodily discomfort: Mild = 34.9 (23.1) / Moderate = 47.9 (20.3) / Severe = 50.0 (28.2) [p = 0.005] - PDQ-39 SI: Mild = 28.0 (14.3) / Moderate = 40.0 (12.4) / Severe = 46.7 (16.4) [p = 0.005] | Very good | (+) |
| Luo (2010) [33] | 71 | Kruskall-Wallis test comparing PDQ-39 scores in patients with PD grouped by H&YS [mean (SD)]:   - Mobility: Stage 1 = 15.74 (15.48) / Stage 2 = 32.11 (23.86) / Stage 3 = 38.44 (29.34) / Stages 4+5 = 75.00 (26.76) [p < 0.001] - ADL: Stage 1 = 17.40 (17.88) / Stage 2 = 24.78 (19.59) / Stage 3 = 16.67 (28.08) / Stages 4+5 = 80.21 (18.87) [p < 0.001] - Emotional wellbeing: Stage 1 = 19.36 (13.81) / Stage 2 = 34.43 (23.62) / Stage 3 = 35.42 (21.71) / Stages 4+5 = 40.10 (24.59) [p = 0.048] - Stigma: Stage 1 = 19.12 (26.65) / Stage 2 = 24.84 (23.94) / Stage 3 = 25.00 (24.55) / Stages 4+5 = 34.338 (46.05) [p = 0.764] - Social support: Stage 1 = 6.37 (13.98) / Stage 2 = 23.03 (21.17) / Stage 3 = 21.88 (24.37) / Stages 4+5 = 28.13 (33.91) [p = 0.026] - Cognition: Stage 1 = 29.41 (17.51) / Stage 2 = 33.72 (18.01) / Stage 3 = 46.88 (28.93) / Stages 4+5 = 46.88 (32.73) [p = 0.408] - Communication: Stage 1 = 10.78 (14.06) / Stage 2 = 30.92 (26.77) / Stage 3 = 40.63 (29.69) / Stages 4+5 = 43.75 (30.78) [p = 0.013] - Bodily discomfort: Stage 1 = 28.43 (16.94) / Stage 2 = 34.43 (25.50) / Stage 3 = 31.25 (21.71) / Stages 4+5 = 56.25 (23.46) [p = 0.064] | Very good | (+) |
| Huang (2010) [34] | 100 | **PDQ-39:**  ANOVA test comparing PDQ-39 SI [mean (SD)] between patients with PD [32.08 (22.17)] and healthy participants [13.98 (13.85)] [p < 0.001] | Very good | (+) |
|  | 100 | **PDQ-8:**  ANOVA test comparing PDQ-8 scores between patients with PD and healthy participants:   - Mobility: PD = 1.37 (1.53) / Healthy control = 0.06 (0.29) [p < 0.001] - ADL: PD = 0.97 (1.50) / Healthy control = 0.01 (0.012) [p < 0.001] - Emotional wellbeing: PD = 1.51 (1.50) / Healthy control = 0.85 (0.97) [p = 0.001] - Stigma: PD = 1.57 (1.64) / Healthy control = 0.44 (0.89) [p < 0.001] - Social support: PD = 0.78 (1.31) / Healthy control = 0.54 (0.95) [p = 0.190] - Cognition: PD = 1.36 (1.42) / Healthy control = 0.74 (0.77) [p = 0.001] - Communication: PD = 1.03 (1.37) / Healthy control = 0.51 (0.90) [p = 0.006] - Bodily discomfort: PD = 1.27 (1.32) / Healthy control = 0.61 (0.87) [p = < 0.001] - PDQ-8: PD = 30.81 (23.73) / Healthy control = 11.76 (13.26) [p < 0.001] | Very good | (+) |
| Zhang (2011) [35] | 126 | Kruskall-Wallis test comparing PDQ-39 scores in patients with PD grouped by H&YS [mean (SD); Stage 1 / Stage 2 / Stage 3]:   - Mobility = 21.64 (17.26) / 36.05 (27.65) / 27.50 (22.64) [p < 0.05] - ADL = 16.89 (20.16) / 31.63 (28.42) / 17.50 (17.03) [p < 0.05] - PDQ-39 SI = 21.33 (12.48) / 28.44 (15.21) / 19.43 (8.69) [p < 0.05] | Very good | (+) |
| Kwon (2013) [36] | – | – | – | – |
| Park (2014) [37] | – | – | – | – |
| Fereshtehnejad (2014) [38] | – | **PDQ-39:**  – | – | – |
|  | – | **PDQ-8:**  – | – | – |
|  |  | **PDQ-8:**  – | – | – |
| Krygowska-Wajs (2015) [39] | – | **PDQ-39:**  – | – | – |
|  | – | **PDQ-8:**  – | – | – |
| Morley (2015, a) [40] | 118 | T-Student test comparing PDQ-39 scores for patients with PD grouped by duration of PD [mean (SD); 1-4 years / 5-20 years]:   - Mobility = 27.03 (26.87) / 41.27 (25.47) [p < 0.01] - ADL = 28.11 (21.86) / 39.62 (24.15) [p < 0.01] - Cognition = 25.53 (18.29) / 32.84 (17.96) [p < 0.05] - Communication = 22.88 (23.14) / 33.05 (25.85) [p < 0.05] - Bodily discomfort = 35.17 (23.67) / 47.17 (24.05) [p < 0.05]   T-Student test comparing PDQ-39 scores for patients with PD grouped by sex [mean (SD); male / female]:   - Communication = 32.07 (23.03) / 22.76 (26.51) [p < 0.05] | Very good | (+) |
| Morley (2015, b) [41] | 118 | Differences in PDQ-39 scores were observed for patients grouped by duration of PD (years) and sex | Very good | (?) |
| Jesus-Ribeiro (2017) [42] | – | **PDQ-39:**  – | – | – |
|  | – | **PDQL:**  – | – | – |
| Galeoto (2018) [43] | – | – | – | – |
| Suratos (2018) [44] | 100 | ANOVA test comparing PDQ-39 scores in patients with PD grouped by H&YS [mean (SD); Stage 1 / Stage 1.5 / Stage 2 / Stage 2.5 / Stage 3 / Stage 4]:   - Mobility: 19.50 (15.67) / 37.71 (20.04) / 41.61 (27.77) / 43.33 (20.95) / 62.94 (21.03) / 67.50 (16.20) [p < 0.0001] - ADL: 10.83 (9.86) / 20.13 (17.48) / 30.50 (23.51) / 35.28 (17.46) / 53.87 (24.50) / 77.97 (22.27) [p < 0.0001] - Emotional wellbeing: 17.92 (17.79) / 20.49 (16.71) / 31.55 (25.62) / 33.33 (21.82) / 40.03 (21.91) / 40.48 (25.20) [p = 0.0458] - Cognition: 15.63 (15.66) / 19.79 (114.56) / 32.37 (25.06) / 25.00 (22.66) / 28.57 (20.30) / 47.32 (25.48) [p = 0.0412] - Communication: 25.83 (15.66) / 11.11 (15.21) / 23.21 (22.61) / 26.67 (21.18) / 26.19 (24.61) / 52.38 (34.60) [p = 0.0218] - Bodily discomfort: 21.37 (19.72) / 40.77 (26.68) / 40.77 (26.68) / 41.67 (22.71) / 41.67 (22.71) / 50.60 (21.74) [p = 0.0153] - PDQ-39 SI: 18.40 (13.25) / 24.81 (10.01) / 31.62 (19.92) / 31.60 (14.83) / 38.80 (14.10) / 52.78 (13.25) [p = 0.0002]   No significant differences were observed for the remaining dimensions. | Very good | (+) |
| Holden (2019) [45] | 201 | **PDQ-39:**  Standarized response mean (SRM) comparing patients with palliative care (-0.30 [CI95 = {-0.49}-{-0.10}]) vs. Patients without palliative care (-0.002 [CI95 = {-0.22}-0.21]) [p = 0.05] | Very good | (+) |
|  | 201 | **McGill QOL:**  Standarized response mean (SRM) comparing patients with palliative care (0.14 [CI95 = {-0.12}-0.35]) vs. Patients without palliative care (0.13 [CI95 = {-0.09}-0.34]) [p > 0.05] | Very good | (–) |
|  | 201 | **PROMIS-29:**  Standarized response mean (SRM) comparing patients with palliative care (-0.25; 95CI = -0.45-[-0.06]) vs. Patients without palliative care (-0.07; 95CI = -0.28-0.14) [p = 0.05] | Very good | (+) |
|  | 201 | **QOL-AD:**  Standarized response mean (SRM) comparing patients with palliative care (0.13 [95CI = {-0.09}-0.34]) vs. Patients without palliative care (-0.22 [95CI = {-0.42}-0.02]) [p > 0.05] | Very good | (+) |
| Nelson (2020) [46] | – | – | – | – |
| Kim (2020) [47] | – | **PDQ-39:**  – | – | – |
|  | – | **PDQ-8:**  – | – | – |
| Hanff (2023) [48] | – | – | – | – |
| Katsarou (2004) [49] | 228 | Kruskall-Wallis test comparing PDQ-8 scores of patients with PD grouped by H&YS [mean (SD)]: Stage 1 = 14.20 (12.50) / Stage 2 = 24.90 (18.10) / Stage 3 = 28.38 (16.71) / Stage 4 = 25.86 (16.98) [p < 0.001] | Very good | (+) |
| Tan (2007) [50] | – | – | – | – |
|  | – | – | – | – |
|  | – | – | – | – |
| Jenkinson (2007) [51] | – | – | – | – |
|  | – | – | – | – |
|  | – | – | – | – |
|  | – | – | – | – |
|  | – | – | – | – |
| Franchignoni (2008) [52] | – | – | – | – |
|  | – | – | – | – |
|  | – | – | – | – |
| Dal Bello-Haas (2009) [53] | – | – | – | – |
| Alvarado-Bolaños (2015) [54] | 585 | **PDQ-8:**  U Mann-Whitney / Kruskall-Wallis test comparing PDQ-8 score of patients with PD grouped by [mean (SD)]:   - Gender: Male = 17,31 (12,35) / Female = 19,28 (13,71) [p = 0,07] - Level of studies: University = 15,41 (11,09) / No universitary = 19,04 (13,43) [p < 0,05] - Work status: Employed = 13,88 (9,95) / Unemployed = 19,90 (13,67) [p < 0,001] - Comorbidities: Yes = 18,78 (13,47) / No = 17,61 (12,51) [p = 0,27] - Treatment with levodopa: Yes = 19,68 (13,18) / No = 13,61 (11,33) [p < 0,001] - Duration of PD: 0-8 years = 16,12 (12) / >8 years = 22,35 (13,9) [p < 0.001] - EQ-VAS: 0-20 = 36,92 (14,10) / 21-40 = 32,18 (12,28) / 41-60 = 25,63 (12,25) / 61-80 = 19,43 (11,49= / 81-100 = 9,54 (8,65) [p < 0,001] - H&YS: Mild (I+II) = 13,80 (10,73) / Moderate (III) = 22,76 (13,35) / Severe (IV+V) = 30,27 (11,97) [p < 0,001]   U Mann-Whitney / Kruskall-Wallis test comparing PDQ-8 score of patients with PD grouped by [median (IQR)]:   - Dyskinesia: Yes = 20 (22) / No = 14 (18) [p < 0,001] - Wearing Off: Yes = 22 (20) / No = 14 (18) ( [p < 0,001] - Freezing: Yes = 24 (18) / No = 12 (15) [p < 0,001] - Postural instability: Yes = 20 (22) / No = 10 (12) [p < 0,001] - Cognitive impairment: Yes = 20 (20) / No = 12 (16) [p < 0,001] - Depression: Yes = 24 (16) / No = 10 (12) [p < 0,001] |  |  |
|  | 585 | **EQ-5D-5L:**  U Mann-Whitney / Kruskall-Wallis test comparing EQ-5D-5L utility values of patients with PD grouped by [mean (SD)]:   - Gender: Male = 0.73 (0.17) / Female = 0.67 (0.20) [p = 0.001] - Level of studies: University = 0.73 (0.17) / Non universitary = 0.695 (0.19) [p = 0.01] - Work status: Employed = 0.75 (0.13) / Unemployed = 0.68 (0.20) [p < 0.001] - Comorbidities: Yes = 0.70 (0.19) / No = 0.70 (0.18) [p = 0.84] - Treatment with levodopa: Yes = 0.68 (0.19) / No = 0.76 (0.17) [p < 0.001] - Duration of PD: 0-8 years = 0.73 (0.17) / >8 years = 0.65 (0.21) [p < 0.05] - EQ-VAS: 0-20 = 0.33 (0.23) / 21-40 = 0.54 (0.19) / 41-60 = 0.60 (0.18) / 61-80 = 0.68 (0.16) / 81-100 = 0.82 (0.12) [p < 0.001] - H&YS: Mild (I+II) = 0.77 (0.13) / Moderate (III) = 0.65 (0.18) / Severe (IV+V) = 0.47 (0.22) [p < 0.001]   U Mann-Whitney / Kruskall-Wallis test comparing EQ-5D-5L utility values of patients with PD grouped by [median (IQR)]:   - Dyskinesia: Yes = 0.68 (0.27) / No = 0.75 (0.20) [p < 0.001] - Wearing Off: Yes = 0.67 (0.27) / No = 0.77 (0.19) [p < 0.001] - Freezing: Yes = 0.65 (0.28) / No = 0.78 (0.18) [p < 0.001] - Postural instability: Yes = 0.68 (0.26) / No = 0.79 (0.14) [p < 0.001] - Cognitive impairment: Yes = 0.70 (0.25) / No = 0.77 (0.19) [p < 0.001] - Depression: Yes = 0.64 (0.25) / No = 0.80 (0.15) [p < 0.001] | Very good | (+) |
|  | 585 | **EQ-VAS:**  U Mann-Whitney / Kruskall-Wallis test comparing EQ-5D-5L utility values of patients with PD grouped by [median (IQR)]:   - Dyskinesia: Yes = 75 (25) / No = 80 (20) [p < 0.001] - Wearing-Off: Yes = 75 (20) / No = 80 (20) [p < 0.001] - Freezing: Yes = 70 (30) / No = 80 (20) [p < 0.001] - Postural instability: Yes = 70 (35) / No = 80 (20) [p < 0.001] - Cognitive impairment: Yes (25) / No = 80 (20) [p < 0.001]   Depression: Yes = 70 (30) / No = 85 (20) [p < 0.001] | Very good | (+) |
| Kahraman (2018) [55] | – | – | – | – |
| Ramadhan (2022) [56] | – | **PDQ-8:**  – | – | – |
|  | – | **EQ-5D-3L:**  – | – | – |
| Stathis (2022) [57] | – | **PDQ-8:**  – | – | – |
|  | – | **PDQoL-7:**  – | – | – |
| Kawaguchi (2021) [58] | – | – | – | – |
| De Boer (1996) [59] | 384 | ANOVA test comparing PDQL scores of patients with PD grouped by severity of disease identified differences (↑ Severity 🡪 ↓ Score) | Very good | (?) |
| Serrano-Dueñas (2004) [60] | 137 | Kruskall-Wallis test comparing PDQL scores of patients with PD grouped by H&YS (↑ Severity 🡪 ↓ Score) | Very good | (?) |
| Campos (2011) [61] | 58 | Kruskall-Wallis test comparing PDQL scores of patients with PD grouped by H&YS (↑ Severity 🡪 ↓ Score) | Very good | (?) |
| Dereli (2015) [62] | – | – | – | – |
| Welsh (2003) [63] | 222 | ANOVA / Kruskall-Wallis (*) test comparing PDQL scores of patients with PD grouped by H&YS:   - Total: Stage 1 = 27.4 / Stage 1.5 = 37.7 / Stage 2 = 38.8 / Stage 2.5 = 40.9 / Stage 3 = 48.7 / Stage 4 = 58.5 / F (ANOVA) = 10.8 / Relative validity [F Dimension / F PDQUALIF Total] = 1.00 - Social / Role life: Stage 1 = 31.0 / Stage 1.5 = 41.1 / Stage 2 = 44.5 / Stage 2.5 = 45.0 / Stage 3 = 57.5 / Stage 4 = 79.6 / F = 11.6 / Relative validity = 1.07 - Self-image / Sexuality: Stage 1 = 27.4 / Stage 1.5 = 39.1 / Stage 2 = 41.1 / Stage 2.5 = 40.9 / Stage 3 = 47.4 / Stage 4 = 63.1 / F = 3.9 / Relative validity = 0.39 - Sleep: Stage 1 = 23.1 / Stage 1.5 = 40.8 / Stage 2 = 37.9 / Stage 2.5 = 43.6 / Stage 3 = 48.0 / Stage 4 = 58.3 / F = 3.2 / Relative validity = 0.29 - Outlook: Stage 1 = 53.9 / Stage 1.5 = 58.1 / Stage 2 = 57.3 / Stage 2.5 = 57.9 / Stage 3 = 59.8 / Stage 4 = 62.5 / F = 0.4 / Relative validity = 0.04 - Physical function: Stage 1 = 18.1 / Stage 1.5 = 35.3 / Stage 2 = 34.0 / Stage 2.5 = 36.2 / Stage 3 = 46.6 / Stage 4 = 44.7 / F = 7.4 / Relative validity = 0.68 - Independence: 1 = 0.0 / 1.5 = 0.7 / 2 = 5.3 / 2.5 = 9.0 / 3 = 18.1 / 4 = 44.4 / F = * / Validez relativa = * - Urinary function: Stage 1 = 33.7 / Stage 1.5 = 49.4 / Stage 2 = 50.6 / Stage 2.5 = 53.5 / Stage 3 = 63.0 / Stage 4 = 56.9 / F = 4.0 / Relative validity = 0.37 | Very good | (+) |
| Calne (1996) [64] | 147 | T-Student test comparing the PIMS score of patients with PD grouped by:   - Fluctuations vs. Stable [p = 0.81] - On vs. Off: Effect size = 10.4 [p < 0.0001] | Very good | (+) |
| Schulzer (2003) [65] | 116 | U Mann-Whitney test comparing the PIMS score of patients with PD grouped by dose of tolcapone: Effect size = 0.37 [p < 0.05] | Very good | (+) |
| Aggarwal (2020) [66] | 295 | ANOVA test comparing the QLPD score of patients with PD grouped by self-reported QoL in the visual analogue scale (included in QLPD) [mean (SD)]:   - Total Score: Very bad = 59.7 / Bad = 46.6 / Neither good nor bad = 34.8 / Good = 25.6 / Very Good = 18.9 [p < 0.0001] - ADL: Very bad = 63.3 / Bad = 48.5 / Neither good nor bad = 40.0 / Good = 29.1 / Very Good = 25.8 [p < 0.0001] - Mobility: Very bad = 52.3 / Bad = 41.3 / Neither good nor bad = 32.4 / Good = 20.9 / Very Good = 14.4 [p < 0.0001] - Psychological: Very bad = 56.8 / Bad = 41.4 / Neither good nor bad = 27.1 / Good = 22.9 / Very Good = 17.1 [p < 0.0001] - Fear: Very bad = 60.2 / Bad = 53.7 / Neither good nor bad = 39.4 / Good = 28.1 / Very Good = 20.2 [p < 0.0001] - Social: Very bad = 59.1 / Bad = 40.6 / Neither good nor bad = 29.5 / Good = 20.9 / Very Good = 14.2 [p < 0.0001] - Family: Very bad = 76.9 / Bad = 51.8 / Neither good nor bad = 35.3. / Good = 24.4 / Very Good = 9.9 [p < 0.0001] - Treatment: Very bad = 68.9 / Bad = 66.5 / Neither good nor bad = 55.5 / Good = 45.1 / Very Good = 38.5 [p < 0.0001] - Finances: Very bad = 67.4 / Bad = 51.9 / Neither good nor bad = 31.2 / Good = 18.6 / Very Good = 9.9 [p < 0.0001] - Nonmotor symptoms: Very bad = 53.8 / Bad = 42.1 / Neither good nor bad = 31.8 / Good = 24.5 / Very Good = 18.5 [p < 0.0001]   ANOVA test comparing the QLPD score of patients with PD grouped by H&YS [mean (SD)]: Group 1 (H&YS ≤ 2.5) = 30 (14.8) / Group 2 (H&YS > 2.5) = 44.7 (15.7) | Very good | (+) |
| Kuehler (2003) [67] | – | **QLSM-DSB:**  – | – | – |
|  | – | **QLSM-MD:**  – | – | – |
| Krygowska-Wajs (2015) [68] | – | **QLSM-DSB:**  – | – | – |
|  | – | **QLSM-MD:**  – | – | – |
| Bose (2018) [69] | – | – | – | – |
| Diniz (2018) [70] | 44 | U Mann-Whitney test comparing the QOLSQ scores of patients with PD vs. healthy controls [mean (SD)]:   - Global: Casos = 18.63 (18.63) / Controles = 1.09 (4.49) [p < 0.01] - Domain 1: Patients with PD = 45.33 (34.49) / Healthy controls = 0.18 (1.21) [p < 0.01] - Domain 2: Patients with PD = 45.45 (23.55) / Healthy controls = 2.55 (8.17) [p < 0.01] - Domain 3: Patients with PD = 48.48 (20.03) / Healthy controls = 4.79 (8.99) [p < 0.01] - Domain 4: Patients with PD = 36.24 (19.3) / Healthy controls = 0.59 (2.89) [p < 0.01] | Very good | (+) |
| García-Gordillo (2013) [71] | 133 | **15D:**  U Mann-Whitney test comparing the 15D utility values of patients with PD grouped by H&YS [p < 0.001]:   - Stage 1+2: Mean (SD) = 0.81 (0.10) / Median (IQR) = 0.83 (0.13)   Stage 3+4: Mean (SD) = 0.70 (0.17) / Median (IQR) = 0.76 (0.29) | Very good | (+) |
|  | 133 | **EQ-5D-5L:**  U Mann-Whitney test comparing the EQ-5D-5L utility values of patients with PD grouped by H&YS [p < 0.001]:   - Stage 1+2: Mean (SD) = 0.70 (0.18) / Median (IQR) = 0.72 (0.24)   Stage 3+4: Mean (SD) = 0.53 (0.28) / Median (IQR) = 0.59 (0.41) | Very good | (+) |
| Del Pozo-Cruz (2018) [72] | 229 | **15D:**  U Mann-Whitney test comparing the 15D utility values of patients with PD grouped by H&YS [p < 0.001]:   - Stage 1+2: Mean (SD) = 0.77 (0.14) / Median (IQR) = 0.81 (0.20) / Range = 0.31-1.00 - Stage 3+4: Mean (SD) = 0.64 (0.27) / Median (IQR) = 0.65 (0.22) / Range = 0.40 – 0.91 | Very good | (+) |
|  | 229 | **SF-6D:**  U Mann-Whitney test comparing the SF-6D utility values of patients with PD grouped by H&YS [p < 0.001]:   - Stage 1+2: Mean (SD) = 0.59 (0.25) / Median (IQR) = 0.66 (0.336) / Range = -0.22-1.000 - Stage 3+4: Mean (SD) = 0.35 (0.27) / Median (IQR) = 0.40 (0.39) / Range = -0.22-0.985 | Very good | (+) |
| Luo (2009) [73] | – | **EQ-5D-3L:**  – | – | – |
|  |  | **EQ-VAS:**  – | – | – |
|  | 135 | **EQ-5D-3L:**  U Mann-Whitney / Kruskall-Wallis test comparing EQ-5D-3L utility values of patients with PD grouped by [median (IQR)]:   - Perception of health changes compared with the previous year: Better = 0.80 (0.73-0.85) / Equal = 0.85 (0.73-1.00) / No change = 0.59 (0.52-0.80) [p < 0.0001] - Dyskinesia: No = 0.80 (0.65-1.00) / Yes = 0.52 (0.52-0.73) [p < 0.0001]   Wearing-Off periods: No = 0.80 (0.71-1.00) / Yes = 0.62 (0.52-0.78) [p < 0.0001] | Very good | (+) |
|  |  | **EQ-VAS:**  U Mann-Whitney / Kruskall-Wallis test comparing EQ-5D-3L utility values of patients with PD grouped by [median (IQR)]:   - Perception of health changes compared with the previous year: Better = 76.5 (70-85) / Equal = 80.0 (70-80) / Worse = 65.0 (50-74.5) [p < 0.001] - Dyskinesia: No = 75.0 (65-80) / Yes = 66.0 (50-75) [p = 0.004]   Wearing-Off periods: No = 78.0 (70-80) / Yes = 68.0 (54-75) [p < 0.001] | Very good | (+) |
|  | 71 | **EQ-5D-3L:**  U Mann-Whitney / Kruskall-Wallis test comparing EQ-5D-3L utility values of patients with PD grouped by [median (IQR)]:   - Perception of health changes compared with the previous year: Better = 0.85 (0.66-1.00) / Equal = 0.85 (0.74-1.00) / Worse = 0.73 (0.26-0.85) [p = 0.032] - Dyskinesia: No = 0.81 (0.72-1.00) / Yes = 0.52 (0.08-0.62) [p = 0.009]   Wearing-Off periods: No = 0.85 (0.77-1.00) / Yes = 0.62 (0.26-0.73) [p < 0.0001] | Very good | (+) |
|  |  | **EQ-VAS:**  U Mann-Whitney / Kruskall-Wallis test comparing EQ-5D-3L utility values of patients with PD grouped by [median (IQR)]:   - Perception of health changes compared with the previous year: Better = 70.0 (59-85) / Equal = 80.0 (70-89) / Worse = 70.0 (60-80) [p = 0.172] - Dyskinesia: No = 70.0 (60-80) / Yes = 70.0 (50-80) [p = 0.654]   Wearing-Off periods: No = 75.0 (60-85) / Yes = 69.0 (59-80) [p 0.099] | Very good | (–) |
| Garcia-Gordillo (2015) [74] | 133 | **EQ-5D-3L:**  U Mann-Whitney / Kruskall-Wallis test comparing EQ-5D-3L utility values of patients with PD grouped by H&YS [mean (SD); median (IQR)]: Stage 1+2 = 0.76 (0.21); 0.82 (0.30) / Stages 3+4 = 0.57 (0.34); 0.65 (0.31) [p = 0.001] | Very good | (+) |
|  | 133 | **SF-6D:**  U Mann-Whitney / Kruskall-Wallis test comparing EQ-5D-3L utility values of patients with PD grouped by H&YS [mean (SD); median (IQR)]: Stage 1+2 = 0.67 (0.19); 0.69 (0.24) / Stages 3+4 = 0.45 (0.31; 0.51 (0.49) [p = 0.001] | Very good | (+) |
| Nowinski (2010) [75] | – | – | – | – |
| Nowinski (2016) [76] | 120 | T-Student test comparing Neuro-QOL scores of patients with PD grouped by H&YS stages 1+2 vs. 3+4: Statistically significant differences were observed (effect size [Cohen’s D] = 0.5-1.11) | Very good | (+) |
| Kuspinar (2019) [77] | – | – | – | – |
| Kuspinar (2020) [78] | – | – | – | – |
| Hagell (2011) [79] | – | – | – | – |
| Steffen (2008) [80] | – | – | – | – |
| Hagell (2008) [81] | – | – | – | – |
| Schneider (2010) [82] | – | – | – | – |
| Hendred (2016) [83] | 96 | ANOVA test comparing scores of WHOQOL-BREF of patients with PD vs. healthy controls found differences in physical health, psychological health, social relationships and environment | Very good | (?) |
