# Supplementary material for: Quality of life measures in Parkinson’s disease: a systematic literature review of patient-reported outcomes measures (PROMs) and their psychometric properties
Source: J Neurol. 2025 Aug 28;272(9):598. doi: 10.1007/s00415-025-13348-x (PMC12394374; doi:10.1007/s00415-025-13348-x)
Supplement: Supplementary file 9 — Supplementary file9 (DOCX 174 KB) [file 415_2025_13348_MOESM9_ESM.docx]

**Quality of Life Measures in Parkinson’s Disease: A Systematic Literature Review of Patient-Reported Outcomes Measures (PROMs) and their Psychometric Properties**

**– ONLINE RESOURCE 7 –**

Table S13. Descriptions of the studies’ findings in relation to the Criterion Validity of the PROMs.

| Eligible study | Sample size | Findings of the study in relation to Criterion Validity | COSMIN assessment | |
| --- | --- | --- | --- | --- |
|  |  |  | **RoB** | **Good property** |
| Spliethoff-Kamminga (2003) [1] | 54 | Correlations of Bela-P-K Bb (Achievement capability - Physical symptoms / Fear - Emotional symptoms / Social functioning / Partner-bonding – Family / Total Bb) with:   - SIP (Total) = 0.48** / 0.37* / 0.52** / 0.44* / 0.57** - SIP (Pyshical dimension) = 0.45** / - / 0.49** / - / 0.50** - SIP (Pyshical dimension – Body care and Mobility) = 0.46** / - / 0.54** / - / 0.52** - SIP (Pyshical dimension - Mobility) = 0.45** / - / - / 0.44* / 0.49** - SIP (Psychosocial dimension) = - / 0.37* / - / - / - - SIP (Psychosocial dimension – Emotional behaviour) = - / 0.37* / - / - / - - SIP (Psychosocial dimension – Social interaction) = 0.50** / 0.37* / 0.54** / 0.39* / 0.58** - SIP (Independent categories - Home) = 0.45** / - / 0.43* / - / - - COOP/WONCA (Total) = 0.65** / 0.69** / 0.58** / 0.43* / 0.69** - Loneliness Scale (Total) = - / - / 0.47** / 0.49** / 0.49**   Statistical significance: (*) p < 0.01; (**) p < 0.001 [Two-tailored]; (-) Data not available in report | Very good | (+) |
| Ortelli (2017) [2] | 202 | Correlations of Bela-P-K Bb with PDQ-39 (Total / Mobility / Emotional wellbeing / Social support):   - Total = 0.63 / 0.49 / 0.59 / 0.51 - Achievement capability / Physical symptoms = 0.62 / 0.55 / 0.47 / 0.39 - Fear / Emotional symptoms = 0.56 / 0.40 / 0.66 / 0.44 - Social functioning = 0.52 / 0.38 / 0.50 / 0.45 - Partner-bonding / Family = 0.43 / 0.31 / 0.38 / 0.48   Correlations of Bela-P-K Bb with PDQ-39 (Total / Mobility / Emotional wellbeing / Social support):   - Total = 0.54 / 0.46 / 0.48 / 0.33 - Achievement capability / Physical symptoms = 0.58 / 0.56 / 0.40 / 0.27 - Fear / Emotional symptoms = 0.49 / 0.38 / 0.56 / 0.30 - Social functioning = 0.46 / 0.36 / 0.44 / 0.33 - Partner-bonding / Family = 0.35 / 0.26 / 0.31 / 0.36   Statistical significance: No data available. | Very good | (+) |
| Bayen (2021) [3] | – | – | – | – |
| Aggarwal (2013) [4] | 277 | Statistically significant correlations:   - Indo-PDQOL Total score with SF-36 Utility = -0.61 - Indo-PDQOL (Finance) with socioeconomic status = [Not specified / Statistically significant] - Indo-PDQOL (Treatment related) with levodopa dose = [Not specified / Statistically significant] - Indo-PDQOL (Mobility / ADL / Psychological / Pain, sleep & RLS) ~ PDQ-39 (Mobility / ADL / Emotional wellbeing / Bodily discomfort) = Range between 0.52-0.66 - Indo-PDQOL (Psychological / ADL / Pain, sleep & RLS / Social / Total) ~ SF-36 (Mental component / Physical component / Bodily pain / Social functioning / General health) = Range between 0.45-0.65   No data is available regarding the correlation method (Pearson / Spearman). | Very good | (?) |
| Kuharic (2022) [5] | – | – | – | – |
| Kuharic (2024) [6] | – | – | – | – |
| Peto (1995) [7] | – | – | – | – |
|  | 227 | Pearson correlations of PDQ-39 with severity of symptoms (tremor / stiffness / slowness):   - Mobility: 0.24 (p < 0.05) / 0.42 (p < 0.001) / 0.57 (p < 0.001) - Emotional wellbeing: 0.23 (p < 0.05) / 0.32 (p < 0.001) / 0.36 (p < 0.001) | Very good | (+) |
| Jenkinson (1997) [8] | – | – | – | – |
|  | 131 | Spearmna correlations of PDQ-39 (SI / Mobility / ADL / Emotional wellbeing / Stigma / Social support / Cognition / Communication / Bodily discomfort) with:   - H&YS: 0.43 ** / 0.63 ** / 0.58 ** / 0.27 * / 0.31 / 0.16 ** / 0.40 ** / 0.45 ** / 0.32 ** - Columbia scale: 0.51 ** / 0.54 ** / 0.56 ** / 0.22 ** / 0.29 ** / 0.08 * / 0.35 ** / 0.42 ** / 0.22 *   Statistical significance: (*) p < 0.05; (**) p < 0.001 | Very good | (+) |
| Jenkinson (1997) [9] | – | **PDQ-39:**  – | – | – |
|  | – | **PDQ-8:**  Pearson correlations of PDQ-8 with other PROMs:   - PDQ-39 SI = 0.95 - Columbia = 0.47 | Very good | (+) |
| Martínez-Martín (1998) [10] | 103 | Spearman correlations of PDQ-39 dimensions with other measures (H&YS / UPDRS / S&E / SPMSQ / GDS / HADS-A / HADS-D):   - Mobility: 0.69 / 0.63 / -0.73 / - / 0.47 / 0.49 / 0.60 - ADL: 0.67 / 0.66 / -0.67 / - / 0.43 / 0.43 / 0.52 - Emotional wellbeing: 0.44 / 0.42 / -0.47 / - / 0.69 / 0.74 / 0.70 - Stigma: 0.30 / 0.31 / -0.33 / - / 0.37 /0.39 /0.41 - Social support: 0.32 / 0.39 / -0.34 / - / 0.28 / 0.26 / 0.39 - Cognition: 0.27 / 0.32 / -0.25 / 0.27 / 0.31 / 0.43 / 0.48 - Communication: 0.54 / 0.49 / -0.54 / - / 0.47 / 0.59 / 0.51 - Bodily discomfort: 0.28 / 0.28 /-0.29 / - / 0.20 / 0.41 / 0.41   Statistical significance: (-) No significant; (Rho > 0.20) p < 0.05; (Rho > 0.25) p < 0.01; (Rho = 0.33) p < 0.001 | Very good | (+) |
| Bushnell (1999) [11] | 75 | Spearman correlations of PDQ-39 dimensions with SF-36 dimensions:   - PDQ-39 (Mobility) and SF-36 (Physical functioning) = -0.88 (p < 0.001) - PDQ-39 (ADL) and SF-36 (Physical role) = -0.59 (p < 0.001) - PDQ-39 (Emotional wellbeing) and SF-36 (Mental health) = -0.78 (p < 0.001) - PDQ-39 (Social support) and SF-36 (Social functioning) = -0.22 (p = 0.08) - PDQ-39 (Bodily discomfort) and SF-36 (Bodily pain) = -0.73 (p < 0.001)   Spearman correlations of PDQ-39 with severity of tremor. stiffness. slowness. freezing and jerking. | Very good | (+) |
| Andreu (2000) [12] | 126 | Pearson correlations of PDQ-39 dimensions (Mobility / ADL / Emotional wellbeing / Stigma / Social support / Cognition / Communication / Bodily discomfort / SI) with H&YS: 0.57 / 0.49 / 0.24 / 0.21 / 0.07 / 0.11 / 0.48 / 0.23 / 0.46  Pearson correlations of PDQ-39 with SF-36, UPDRS and MADRS. | Very good | (+) |
| Schrag (2000) [13] | 97 | **PDQ-39:**  Spearman correlations of PDQ-39 (ADL / Bodily discomfort / Cognition / Communication / Emotional wellbeing / Mobility / Social support / Stigma / SI) with other PROMs:   - H&YS = 0.63* / 0.36 / 0.43* / 0.49* / 0.40* / 0.69* / 0.22 / 0.26 / 0.60* - S&E = -0.55* / -0.49* / -0.44* / -0.50* / -0.51* / -0.68* / -0.36 / -0.36 / -0.65* - UPDRS-Motor = 0.52* / 0.28 / 0.26 / 0.31 / 0.20 / 0.45* / 0.10 / 0.39/ * 0.41* - Duration of PD (years) = 0.30 / 0.16 / 0.15 / 0.19 / 0.04 / 0.18 / -0.01 / 0.10 / 0.18 - BDI = 0.53* / 0.53* / 0.67* / 0.47* / 0.57* / 0.54* / 0.32 / 0.44* / 0.68* - MMSE = -0.23 / -0.31 / -0.15 / -0.23 / -0.36 / -0.39* / -0.09 / -0.10 / -0.32   Spearman correlations of EQ-5D-3L (Mobility / Usual activities / Self-care / Pain – Discomfort / Anxiety – Depression) with PDQ-39:   - Mobility = 0.73* / 0.59* / 0.67* / 0.54* / 0.47* / -0.74* / -0.56* - ADL = 0.65* / 0.72* / 0.65* / 0.37 / 0.38 / -0.63* / -0.41* - Stigma = 0.54* / 0.42* / 0.46* / 0.37 / 0.50* / -0.54* / -0.43* - Social support = 0.26 / 0.28 / 0.28 / 0.31 / 0.26 / -0.35 / -0.30 - Cognition = 0.49* / 0.51* / 0.58* / 0.35 / 0.45* / -0.55* / -0.47* - Communication = 0.53* / 0.58* / 0.57* / 0.34 / 0.28 / -0.56* / -0.42* - Bodily discomfort = 0.40 / 0.35 / 0.45* / 0.47* / 0.40 / -0.55* / -0.51* - SI = 0.69* / 0.63* / 0.68* / 0.54* / 0.55* / -0.75* / -0.60*   Statistical significance: (*) p < 0.0005 | Very good | (+) |
|  | 97 | **EQ-5D-3L:**  Spearman correlations of EQ-5D-3L (Mobility / Usual activities / Self-care / Pain – Discomfort / Anxiety – Depression) with other PROMs:   - H&YS = 0.70* / 0.51* / 0.65* / 0.49* / 0.41* / -0.68* - S&E = -0.65* / -0.50* / -0.65* / -0.47* / -0.37 / 0.66* - UPDRS-Motor = 0.40* / 0.36 / 0.40* / 0.31 / 0.26 / -0.40* - Duration of PD (years) = 0.35 / 0.25 / 0.29 / 0.12 / 0.04 / -0.21 - BDI = 0.45* / 0.54* / 0.59* / 0.45* / 0.55* / -0.63* - MMSE = -0.37 / -0.17 / -0.37 / -0.24 / -0.26 / 0.35   Spearman correlations of EQ-5D-3L (Mobility / Usual activities / Self-care / Pain – Discomfort / Anxiety – Depression) with PDQ-39:   - Mobility = 0.73* / 0.59* / 0.67* / 0.54* / 0.47* / -0.74* / -0.56* - ADL = 0.65* / 0.72* / 0.65* / 0.37 / 0.38 / -0.63* / -0.41* - Stigma = 0.54* / 0.42* / 0.46* / 0.37 / 0.50* / -0.54* / -0.43* - Social support = 0.26 / 0.28 / 0.28 / 0.31 / 0.26 / -0.35 / -0.30 - Cognition = 0.49* / 0.51* / 0.58* / 0.35 / 0.45* / -0.55* / -0.47* - Communication = 0.53* / 0.58* / 0.57* / 0.34 / 0.28 / -0.56* / -0.42* - Bodily discomfort = 0.40 / 0.35 / 0.45* / 0.47* / 0.40 / -0.55* / -0.51* - SI = 0.69* / 0.63* / 0.68* / 0.54* / 0.55* / -0.75* / -0.60*   Spearman correlations of EQ-5D-3L (Mobility / Usual activities / Self-care / Pain – Discomfort / Anxiety – Depression) with SF-36:   - Physical functioning = -0.58* / -0.44* / -0.52* / -0.42* / -0.36 / -0.57* - Physical role = -0.42* / -0.18 / -0.40 / -0.40 / -0.27 / 0.43* - Bodily pain = -0.35 / -0.33 / -0.36 / -0.59* / -0.32 / 0.56* - General health = -0.37 / -0.40 / -0.48* / -0.36 / -0.35 / 0.47* - Vitality = -0.39 / -0.46* / -0.48* / -0.34 / -0.49* / 0.52* - Social functioning = -0.56* / -0.52* / -0.52* / -0.46* / -0.46* 0.59* - Emotional role = -0.19 / -0.13 / -0.19 / -0.11 / -0.21 / 0.20 - Mental health = -0.32 / -0.43* / -0.45* / -0.36 / -0.53* / 0.50* - Physical Score = -0.52* / -0.41* / -0.51* / -0.53* / -0.31 / 0.61* - Mental Score = -0.25 / -0.33 / -0.34 / -0.20 / -0.41* / 0.35   Statistical significance: (*) p < 0.0005 | Very good | (+) |
|  | 97 | **EQ-VAS:**  Spearman correlations of EQ-VAS with:   - H&YS = -0.32 - S&E = 0.42* - UPDRS-Motor = -0.20 - Duration of PD (years) = -0.03 - BDI = -0.63* - MMSE = 0.23 - PDQ-39 (Mobility / ADL / Stigma / Social support / Cognition / Communication / Bodily discomfort / SI) = -0.56* / -0.41* / -0.43* / -0.30 / -0.47* / -0.42* / -0.51* / -0.60* - SF-36 (Physical functioning / Physical role / Bodily pain / General health / Vitality / Social functioning / Emotional role / Mental health / Physical Score / Mental Score) = 0.47* / 0.43* / 0.54* / 0.62* / 0.49* / 0.53* / 0.25 / 0.49* / 0.55* / 0.40   Statistical significance: (*) p < 0.0005 | Very good | (+) |
|  | 97 | **SF-36:**  Spearman correlations of SF-36 (Physical component / Mental component) with other PROMs:   - H&YS = -0.50* / -0.25 - S&E = 0.49* / 0.27 - UPDRS-Motor = -0.41* / -0.16 - Duration of PD (years) = -0.21 / 0.06 - BDI = -0.42* / -0.49* - MMSE = 0.08 / 0.35   Spearman correlations of SF-36 domains with EQ-5D-3L (Mobility / Self-care / ADL / Pain / Anxiety-Depression / Utility value):   - Physical functioning = -0.58* / -0.44* / -0.52* / -0.42* / -0.36 / -0.57* - Role physical = -0.42* / -0.18 / -0.40 / -0.40 / -0.27 / 0.43* - Pain = -0.35 / -0.33 / -0.36 / -0.59* / -0.32 / 0.56* - General health = -0.37 / -0.40 / -0.48* / -0.36 / -0.35 / 0.47* - Energy = -0.39 / -0.46* / -0.48* / -0.34 / -0.49* / 0.52* - Social functioning = -0.56* / -0.52* / -0.52* / -0.46* / -0.46* 0.59* - Role emotional = -0.19 / -0.13 / -0.19 / -0.11 / -0.21 / 0.20 - Mental health = -0.32 / -0.43* / -0.45* / -0.36 / -0.53* / 0.50* - PCS (Physical Composite Score) = -0.52* / -0.41* / -0.51* / -0.53* / -0.31 / 0.61* - MCS (Mental Composite Score) = -0.25 / -0.33 / -0.34 / -0.20 / -0.41* / 0.35   Spearman correlations of SF-36 (Physical functioning / Role physical / Pain / General health / Energy / Social functioning / Role emotional / Mental health / PCS / MCS) with EQ-VAS = 0.47* / 0.43* / 0.54* / 0.62* / 0.49* / 0.53* / 0.25 / 0.49* / 0.55* / 0.40  Statistical significance: (*) p < 0.0005 | Very good | (+) |
| Katsarou (2001) [14] | 119 | - Spearman correlations of PDQ-39 dimensions with other measures (Duration of PD / ADL-ON / ADL-OFF / UPDRS-ON / UPDRS-OFF / BDI): - Movilidad: 0.55 / -0.28 / -0.30 / 0.26 / 0.23 / 0.37 - Act. Cotidianas: 0.24 / -0.51 / -0.48 / 0.51 / 0.42 / 0.25 - Bienestar emocional: - / - / - / - / - / 0.60 - Estigma: - / - / - / - / - / 0.48 - Apoyo Social: - / - / - / - / - / 0.46 - Cognición: 0.27 / - / - / - / - / - - Comunicación: 0.25 / -0.35 / - 0.36 / 0.35 / 0.31 / 0.29 - Discomfort Corporal: 0.23 / - / - / - / - / 0.34   Statistical significance: (-) No significance; (Rho ≥ 0.23) p < 0.05; (Rho ≥ 0.24) p < 0.01; (Rho ≥ 0.29) p < 0.001 | Very good | (+) |
| Peto (2001) [15] | – | – | – | – |
| Tsang (2002) [16] | – | – | – | – |
| Hagell (2003) [17] | 71 | Spearman correlations of PDQ-39 dimensions with NHP dimensions:   - PDQ-39 (Mobility) with NHP (Physical mobility): 0.885 [p < 0.01] - PDQ-39 (Emotional wellbeing) with NHP (Emotional reactions): 0.831 [p < 0.01] - PDQ-39 (Bodily discomfort) with NHP (Pain): 0.704 [p < 0.01] - PDQ-39 (Social support) with NHP (Social loneliness): 0.319 [p < 0.05] | Very good | (+) |
| Jenkinson (2003) [18] | – | – | – | – |
|  | – | – | – | – |
|  | – | – | – | – |
|  | – | – | – | – |
|  | – | – | – | – |
| Park (2004) [19] | – | – | – | – |
| Tan (2004) [20] | 88 | **PDQ-39:**  Spearman correlations of PDQ-39 dimensions with EQ-5D (Mobility / Self-care / ADL / Pain / Anxiety-Depression / Utility value / EQ-VAS):   - Mobility: 0.71 / 0.66 / 0.71 / - / - / - / - - ADL: 0-58 / 0.71 / 0.65 / - / - / - / - - Emotional wellbeing: - / - / - / - / 0.65 / - / - - Bodily discomfort: - / - / - / 0.53 / - / - / - - SI: - / - / - / - / - / -0.66 / -0.45   Statistical significance: (-) No significant. all other values are statistically significant | Very good | (+) |
|  | 88 | **PDQ-8:**  Spearman correlations of PDQ-8 with other PROMs:   - EQ-5D utility value = -0.69 - EQ-VAS = -0.50 | Very good | (+) |
| Fitzpatrick (2004) [21] | – | – | – | – |
|  | – | – | – | – |
| Haapaniemi (2004) [22] | 259 | **PDQ-39:**  Lineal regression model: 15D (Utility) = -0.0064 x (PDQ-39 SI) + 0.9489 [R^2^ = 0.6716]  Spearman correlation between every dimension of 15D and PDQ-39 (SI / Mobility / ADL / Emotional wellbeing / Stigma / Social support / Cognition / Communication / Bodily discomfort):   - 15D Utility = -0.825* / -0.808* / -0.697* / -0.687* / -0.427* / -0.436* / -0.656* / -0.682* / -0.558* - Mobility = -0.623* / -0.752* / -0.598* / -0.370* / -0.303* / -0.314* / -0.465* / -0.496* / -0.342* - Vision = -0.527* / -0.539* / -0.453* / -0.409* / -0.253 / -0.307* / -0.453* / -0.440* / -0.289* - Earing = -0.311* / -0.261* / -0.214* / -0.273* / -0.188 / -0.230 / -0.326* / -0.237 / -0.125 - Breathing = -0.520* / -0.489* / -0.354* / -0.408* / -0.326* / -0.366* / -0.337* / -0.441* / -0.391* - Sleeping = -0.407* / -0.328* / -0.261* / -0.389* / -0.196 / -0.102 / -0.386* / -0.348* / -0.443* - Eating = -0.528* / -0.510* / -0.582* / -0.370* / -0.345* / -0.271* / -0.361* / -0.409* / -0.247 - Speech = -0.445* / -0.380* / -0.405* / -0.294* / -0.220 / -0.148 / -0.407* / -0.596* / -0.262* - Elimination = -0.442* / -0.437* / -0.314* / -0.381* / -0.146 / -0.241 / -0.448* / -0.378* / -0.285* - Usual activities = -0.675* / -0.695* / -0.693* / -0.483* / -0.373* / -0.402* / -0.464* / -0.538* / -0.397* - Mental function = -0.401* / -0.349* / -0.331* / -0.286* / -0.105 / -0.248 / -0.535* / -0.409* / -0.227 - Discomfort and symptoms = -0.429* / -0.392* / -0.283* / -0.441* / -0.106 / -0.195 / -0.339* / -0.280* / -0.553* - Depression = -0.527* / -0.439* / -0.359* / -0.729* / -0.394* / -0.291* / -0.394* / -0.350* / -0.344* - Distress = -0.441* / -0.325* / -0.280* / -0.660* / -0.324* / -0.256* / -0.373* / -0.320* / -0.332* - Vitality = -0.618* / -0.609* / -0.504* / -0.533* / -0.326* / -0.294* / -0.434* / -0.536* / -0.504* - Sexual activity = -0.429* / -0.484* / -0.411* / -0.209 / -0.151 / -0.160 / -0.397* / -0.420* / -0.236   Spearman correlation between every dimension of PDQ-39 and other measures (UPDRS-Motor / UPDRS-ADL / Tremor / Rigidity / Hypokinesia / Postural Instability / Gait disturbance):   - SI = 0.54** / 0.68** / 0.53** / 0.35** / 0.51** / 053** / 0.58** - Mobility = 0.54** / 0.69** / 0.52** / 0.31** / 0.52** / 0.59** / 0.64** - ADL = 0.57** / 0.72** / 0.56** / 0.35** / 0.51** / 0.53** / 0.60** - Emotional wellbeing = 0.42** / 0.47** / 0.40** / 0.32** / 0.40** / 0.35** / 0.42** - Stigma = 0.31** / 0.38** / 0.30** / 0.21 / 0.29** / 0.25 / 0.34** - Social support = 0.31** / 0.36** / 0.30** / 0.21 / 0.24 / 0.20 / 0.31** - Cognition = 0.43** / 0.50** / 0.41** / 0.23 / 0.38** / 0.41** / 0.47 - Communication = 0.48** / 0.61** / 0.49** / 0.34** / 0.43** / 0.53** / 0.50** - Bodily discomfort = 0.23 / 0.32** / 0.25 / 0.22 / 0.26 / 0.23 / 0.21   Statistical significance: (*) p < 0.001; (**) p < 0.0001 | Very good | (+) |
|  | 259 | **15D:**  Lineal regression model: 15D (Utility) = -0.0064 x (PDQ-39 SI) + 0.9489 [R^2^ = 0,6716]  Spearman correlation between every dimension of 15D and PDQ-39 (SI / Mobility / ADL / Emotional wellbeing / Stigma / Social support / Cognition / Communication / Bodily discomfort):   - 15D Utility = -0.825* / -0.808* / -0.697* / -0.687* / -0.427* / -0.436* / -0.656* / -0.682* / -0.558* - Moiblity = -0.623* / -0.752* / -0.598* / -0.370* / -0.303* / -0.314* / -0.465* / -0.496* / -0.342* - Vision = -0.527* / -0.539* / -0.453* / -0.409* / -0.253 / -0.307* / -0.453* / -0.440* / -0.289* - Earing = -0.311* / -0.261* / -0.214* / -0.273* / -0.188 / -0.230 / -0.326* / -0.237 / -0.125 - Breathing = -0.520* / -0.489* / -0.354* / -0.408* / -0.326* / -0.366* / -0.337* / -0.441* / -0.391* - Sleeping = -0.407* / -0.328* / -0.261* / -0.389* / -0.196 / -0.102 / -0.386* / -0.348* / -0.443* - Eating = -0.528* / -0.510* / -0.582* / -0.370* / -0.345* / -0.271* / -0.361* / -0.409* / -0.247 - Speech = -0.445* / -0.380* / -0.405* / -0.294* / -0.220 / -0.148 / -0.407* / -0.596* / -0.262* - Elimination = -0.442* / -0.437* / -0.314* / -0.381* / -0.146 / -0.241 / -0.448* / -0.378* / -0.285* - Usual activities = -0.675* / -0.695* / -0.693* / -0.483* / -0.373* / -0.402* / -0.464* / -0.538* / -0.397* - Mental function = -0.401* / -0.349* / -0.331* / -0.286* / -0.105 / -0.248 / -0.535* / -0.409* / -0.227 - Discomfort and symptoms = -0.429* / -0.392* / -0.283* / -0.441* / -0.106 / -0.195 / -0.339* / -0.280* / -0.553* - Depression = -0.527* / -0.439* / -0.359* / -0.729* / -0.394* / -0.291* / -0.394* / -0.350* / -0.344* - Distress = -0.441* / -0.325* / -0.280* / -0.660* / -0.324* / -0.256* / -0.373* / -0.320* / -0.332* - Vitality = -0.618* / -0.609* / -0.504* / -0.533* / -0.326* / -0.294* / -0.434* / -0.536* / -0.504* - Sexual activity = -0.429* / -0.484* / -0.411* / -0.209 / -0.151 / -0.160 / -0.397* / -0.420* / -0.236   Spearman correlation between every dimension of 15D and other measures (UPDRS-Motor / UPDRS-ADL / Tremor / Rigidity / Hypokinesia / Postural Instability / Gait disturbance):   - 15D Utility = -0.55** / -0.69** / -0.52** / -0.36** / -0.51** / -0.58** / -0.60** - Mobility = 0.45** / 0.55** / 0.43** / 0.20 / 0.41** / 0.49** / 0.63** - Vision = 0.34** / 0.40** / 0.34** / 0.14 / 0.30** / 0.44** / 0.43** - Easring = 0.15 / 0.28** / 0.12 / 0.04 / 0.18 / 0.17 / 0.18 - Breatinh = 0.23 / 0.28** / 0.20 / 0.21 / 0.23 / 0.20 / 0.32** - Sleeping = 0.20 / 0.25 / 0.18 / 0.21 / 0.27 / 0.31** / 0.17 - Eating = 0.38** / 0.52** / 0.36** / 0.23 / 0.36** / 0.47** / 0.50** - Speech = 0.33** / 0.37** / 0.31** / 0.25 / 0.35** / 0.27** / 0.29** - Elimination = 0.38** / 0.36** / 0.38** / 0.22 / 0.31** / 0.35** / 0.39** - Usual activities = 0.55** / 0.69** / 0.52** / 0.34** / 0.45** / 0.52** / 0.57** - Mental function = 0.21 / 0.30** / 0.21 / 0.06 / 0.19 / 0.29** / 0.26 - Dyscomfort and symptoms = 0.17 / 0.25 / 0.15 / 0.17 / 0.18 / 0.18 / 0.17 - Depression = 0.33** / 0.37** / 0.31** / 0.25 / 0.35** / 0.27** / 0.29** - Distress = 0.19 / 0.22 / 0.17 / 0.20 / 0.18 / 0.11 / 0.14 - Vitality = 0.40** / 0.50** / 0.38** / 0.33** / 0.43** / 0.38** / 0.41** - Sexual life = 0.51** / 0.57** / 0.47** / 0.35** / 0.46** / 0.49** / 0.49**   Statistical significance: (*) p < 0.001; (**) p < 0.0001 | Very good | (+) |
| Martínez-Martín (2004) [23] | 137 | Spearman correlations of PDQ-39 dimensions (SI / Mobility / ADL / Emotional wellbeing / Stigma / Social support / Cognition / Communication / Bodily discomfort) with H&YS: 0.60 / 0.74 / 0.67 / 0.32 / 0.19 / 0.30 / 0.49 / 0.37 / 0.22  Spearman correlations of PDQ-39 dimensions with UPDRS-Part 1, UPDRS-Part 2, UPDRS-Part 3, S&E, HADS-A, HADS-D | Very good | (+) |
| Ma (2005) [24] | 73 | Pearson correlations of PDQ-39 dimensions with SF-36 dimensions:   - PDQ-39 (Mobility) with SF-36 (Physical functioning) = -0.93 - PDQ-39 (Emotional wellbeing) with SF-36 (Mental health) = -0.70 - PDQ-39 (ADL) with SF-36 (Role functioning limitations) = -0.38 - PDQ-39 (Social support) with SF-36 (Social functioning) = -0.29   Pearson correlations of PDQ-39 dimensions with UPDRS parts (range = 0.42-0.86):   - PDQ-39 (Mobility) with UPDRS (ADL) = 0.82 - PDQ-39 (ADL) with UPDRS (ADL) = 0.81 - PDQ-39 (Stigma / Social support / Bodily discomfort) with UPDRS (All subscales) = 0.42-0.53 | Very good | (+) |
| Luo (2005) [25] | 63 | **PDQ-39:**  Spearman correlations of PDQ-39 dimensions with EQ-5D (Movility / Self-care / ADL / Pain 7 Anxiety-Depression / Utility value):   - Movilidad: 0.49 / 0.67 / 0.7855 / 0.46 / 0.58 / -0.75 - Actividades cotidianas: 0.38 *** / 0.57 / 0.49 / 0.21 * / 0.32 ** / -0.49 - Bienestar emocional: 0.27 ** / 0.46 / 0.44 / 0.24 * / 0.56 / -0.50 - Estigma: 0.27 ** / 0.46 / 0.44 / 0.24 * / 0.56 / -0.32 ** - Apoyo social: 0.06 * / 0.25 * / 0.25 * / 0.19 * / 0.31 ** / -0.43 - Cognición: 0.17 * / 0.36 *** / 0.37 *** / 0.32 ** / 0.42 *** / -0.47 - Comunicación: 0.24 * / 0.37 *** / 0.45 * / 0.35 *** / 0.47 / -0.52 - Discomfort corporal: 0.27 ** / 0.35 *** / 0.33 *** / 0.49 / 0.45 / -0.55 - PDQ-39SI: 0.40 *** / 0.62 7 0.61 / 0.46 / 0.69 / -0.76   Statistical significance: (*) No significance; (**) p < 0.05; (***) p < 0.01; ( ) p < 0.001 | Very good | (+) |
|  | 63 | **PDQ-8:**  Spearman correlations of PDQ-8 with EQ-5D (Mobility / Self-care / ADL / Pain / Anxiety-Depression / Utility value): 0,40 * / 0,62 ** / 0,61 ** / 0,46 ** / 0,69 ** / -0,76 **  Statistical significance: (*) p < 0.01; (**) p < 0.001 |  |  |
| Martínez-Martín (2007) [26] | 188 | Spearman correlations of PDQ-39 (SI / Mobility / ADL / Emotional wellbeing / Stigma / Social support / Cognition / Communication / Bodily discomfort) with H&YS: 0.58 / 0.67 / 0.65 / 0.27 / -0.09 / 0.20 / 0.39 / 0.06 / 0.05 [p < 0.05; all correlations]  Spearman correlations of PDFQ-39 with duration of PD (years), duration of the treatment with levodopa, S&E, UDPRS-Mentation, UPDRS-ADL, UPDRS-Motor, HADS-A, HADS-D.  Statistically significant correlations between PDQ-39 dimensions and PDQL dimensions [p < 0.05]:   - PDQ-39 (Mobility) with PDQL (Parkinsonian symptoms) = -0.72 - PDQ-39 (Mobility) with PDQL (Systemic symptoms) = -0.67 - PDQ-39 (Mobility) with PDQL (Social functioning) = -0.73 - PDQ-39 (ADL) with PDQL (Parkinsonian symptoms) = -0.81 - PDQ-39 (ADL) with PDQL (Systemic symptoms) = -0.62 - PDQ-39 (ADL) with PDQL (Social functioning) = -0.73 - PDQ-39 (Emotional wellbeing) with PDQL (Emotional wellbeing) = -0.66 - PDQ-39 (Stigma) with PDQL (Emotional wellbeing) = -0.67 - PDQ-39 (Communication) with PDQL (Parkinsonian symptoms) = -0.66 - PDQ-39 (Communication) with PDQL (Systemic symptoms) = -0.61 - PDQ-39 (Communication) with PDQL (Social functioning) = -0.60 | Very good | (+) |
| Hagell (2007) [27] | – | – | – | – |
| Krikmann (2008) [28] | – | – | – | – |
| Marinus (2008) [29] | 177 | Spearman correlations of PDQ-39 with other PROMs (SCOPA-Psychosocial / HADS / HADS-A / HADS-D / EQ-5D / EQ-VAS) = 0.82 / 0.74 / 0.69 / 0.65 / 0.63 / -0.54 | Very good | (+) |
| Serrano-Dueñas (2008) [30] | 131 | **PDQ-39:**  Spearman correlations of PDQ-39 with other measures (age / duration of PD / duration of the treatment with levodopa / daily dose of levodopa / Pfeiffer / S&E / HADS-A / HADS-D / SCOPA-SPES-A / SCOPA-SPES-B / SCOPA-SPES-C / SCOPA-SPES-Total / UDPRS-Part 1 / UPDRS-Part 2 / UPDRS-Part 3 / UPDRS-Total / PIMS) = 0.222 / 0.354 / 0.210 / 0.510 / 0.114 / -0.674 / 0.668 / 0.777 / 0.676 / 0.747 / 0.455 / 0.720 / 0.727 / 0.748 / 0.688 / 0.745 / 0.802  Statistical significance: All correlations significant, Rho > 0.59 (p < 0.0001) | Very good | (+) |
|  | 131 | **PDQL:**  Spearman correlations of PDQL with other measures (age / duration of PD / duration of the treatment with levodopa / daily dose of levodopa / Pfeiffer / S&E / HADS-A / HADS-D / SCOPA-SPES-A / SCOPA-SPES-B / SCOPA-SPES-C / SCOPA-SPES-Total / UDPRS-Part 1 / UPDRS-Part 2 / UPDRS-Part 3 / UPDRS-Total / PIMS) = -0.237 / -0.281 / -0.213 / -0.334 / -0.360 / 0.93 / -0.653 / -0.782 / -0.841 / -0.824 / -0.710 / -0.856 / -0.767 / -0.821 / -0.759 / -0.823 / -0.896 | Very good | (+) |
|  | 131 | **PIMS:**  Spearman correlations of PIMS with other measures (age / duration of PD / duration of the treatment with levodopa / daily dose of levodopa / Pfeiffer / S&E / HADS-A / HADS-D / SCOPA-SPES-A / SCOPA-SPES-B / SCOPA-SPES-C / SCOPA-SPES-Total / UDPRS-Part 1 / UPDRS-Part 2 / UPDRS-Part 3 / UPDRS-Total / PIMS) [p < 0.0001] = 0.123 / 0.325 / 0.285 / 0.427 / 0.284 / -0.740 / 0.637 / 0.760 / 0.723 / 0.780 / 0.780 / 0.577 / 0.772 / 0.683 / 0.739 / 0.667 / 0.729 / 0.802 / -0.896 | Very good | (+) |
| Žiropađa (2009) [31] | 102 | Pearson correlations of PDQ-39 with SF-36 (Physical functioning / Mental health) = 0.80 / 0.81 | Very good | (+) |
| Nojomi (2010) [32] | 200 | Pearson correlations of PDQ-39 dimensions with SF-36 dimensions:   - PDQ-39 (Mobility) with SF-36 (Physical functioning) = -0.61 [p < 0.01] - PDQ-39 (Emotional wellbeing) with SF-36 (Emotional wellbeing) = -0.55 [p < 0.01] - PDQ-39 (Bodily discomfort) with SF-36 (Pain) = -0.60 [p < 0.01] | Very good | (+) |
| Luo (2010) [33] | 71 | Spearman correlations of PDQ-39 with UPDRS (Mentation / Behavior and attitude / ADL / Motor / Treatment complications):   - Mobility: 0.53 / 0.63 / 0.43 / 0.40 - ADL: 0.34 / 0.65 / 0.44 / 0.16 † - Emotional wellbeing: 0.44 / 0.31 * / 0.24 ** / 0.33 - Stigma: 0.24 ** / 0.25 ** / 0.20 † / 0.30 * - Social support: 0.48 / 0.35 / 0.17 † / 0.21 † - Cognition: 0.36 / 0.37 / 0.10 † / 0.18 - Communication: 0.50 / 0.54 / 0.31 * / 0.21 † - Bodily discomfort: 0.45 / 0.45 / 0.30 * / 0.22 † - SI: 0.64 / 0.68 / 0.45 / 0.37   Spearman correlations of PDQ-39 with SF-36 (Physical functioning / Social functioning / Physical role limitations / Social role limitations / Mental health / Energy / Pain):   - Mobility: -0.68 / -0.69 / -0.35 / -0.43 / -0.66 / -0.43 / -0.35 * - ADL: -0.58 / -0.53 / -0.08 † / -0.28 ** / -0.57 7 -0.34 / -0.31 * - Emotional wellbeing: -0.25 ** / -0.37 / -0.24 ** / -0.25 ** / -0.43 / -0.33 / -0.50 - Stigma: -0.28 ** / -0.32 * / -0.22 † / -0.16 † / -0.43 / -0.25 ** / -0.33 - Social support: -0.35 * / -0.33 * / -0.38 / -0.28 ** / -0.46 / -0.37 / -0.31 * - Cognition: -0.32 / -0.28 ** / -0.18 † / -0.28 ** / -0.37 * / -0.23 ** / -0.17 † - Communication: -0.43 / -0.34 / -0.17 † / -0.31 * / -0.59 / -0.32 * / -0.37 † - Bodily discomfort: -0.43 / -0.34 / -0.63 / -0.37 / -0.32 * / -0.09 † / -0.13 † - SI: -0.64 / -0.58 / -0.41 / -0.43 / -0.71 / -0.45 / -0.44   Statistical significance: ( ) p < 0.001; (*) p < 0.01; (**) p < 0.05; (†) No significance | Very good | (+) |
| Huang (2010) [34] | – | **PDQ-39:**  – | – | – |
|  | 100 | **PDQ-8:**  Pearson correlations between PDQ-8 dimensions and other measures (duration of PD / UPDRS-Mental / UPDRS-ADL / UPDRS-Motor / H6YS / S&E / Daily dose of levodopa / TDQ / PSQI):   - Mobility: 0.38 ** / 0.38 ** / 0.57 ** / 0.53 ** / 0.55 ** / -0.65 ** / 0.48 ** / 0.29 ** / 0.48 ** / 0.38 ** - ADL: 0.26 ** / 0.35 ** / 0.62 ** / 0.51 ** / 0.50 ** / -0.66 ** / 0.38 ** / 0.17 / 0.31 ** / 0.24 * - Emotional wellbeing: 0.19 / 0.50 ** / 0.32 ** / 0.23 * / 0.28 ** / -0.5 ** / 0.33 ** / 0.25 * / 0.74 ** / 0.39 ** - Stigma: 0.28 ** / 0.25 * / 0.26 ** / 0.21 * / 0.23 * / -0.28 * / 0.43 ** / 0.16 / 0.43 ** / 0.14 - Social support: 0.33 ** / 0.27 ** / 0.22 * / 0.17 / 0.18 / 0.28 ** / 0.33 ** / 0.10 / 0.44 ** / 0.09 * - Cognition: 0.29 ** / 0.44 ** / 0.44 ** / 0.26 ** / 0.36 ** / -0.44 ** / 0.43 ** / 0.36 ** / 0.58 ** / 0.35 ** - Communication: 0.40 ** / 0.36 ** / 0.55 ** / 0.35 ** / 0.59 ** / -0.61 ** / 0.48 ** / 0.22 * / 0.43 ** / 0.15 - Bodily discomfort: 0.13 / 0.14 / 0.16 / 0.19 / 0.09 / -0.13 / 0.23 * / 0.19 / 0.32 ** / 0.32 ** - PDQ-8: 0.43 ** / 0.52 ** / 0.60 ** / 0.47 ** / 0.53 ** / -0.65 ** / 0.60 ** / 0.33 / 0.71 ** / 0.39 **   Statistical significance: (*) p < 0.05; (**) p < 0.01 | Very good | (+) |
| Zhang (2011) [35] | 126 | Spearman correlations of PDQ-39 dimensions with SF-36 dimensions:   - PDQ-39 (Mobility) with SF-36 (Physical functioning) = -0.759 [p < 0.001] - PDQ-39 (ADL) with SF-36 (Physical role) = -0.495 [p < 0.001] - PDQ-39 (Emotional wellbeing) with SF-36 (Mental health) = -0.732 [p < 0.001] - PDQ-39 (Social support) with SF-36 (Social functioning) = -0.124 [p = 0.168] - PDQ-39 (Bodily discomfort) with SF-36 (Pain) = -0.523 [p < 0.001] | Very good | (+) |
| Kwon (2013) [36] | 101 | Spearman correlations of PDQ-39 (Mobility / ADL / Emotional wellbeing / Stigma / Social support / Cognition / Communication / Bodily discomfort / SI) with H&YS: 0.345 * / 0.461 * / 0.140 ** / 0.188 ** / 0.198 ** / 0.372 * / 0.378 * / 0.204 ** / 0.356 *  Spearman correlations of PDQ-39 with age, duration of PD (years), daily dose of levodopa (mg), UPDRS-Part 1, UPDRS-Part 2, UPDRS-Parte 3; MADRS, MMSE and ESS  Statistical significance: (*) p < 0.01; (**) p < 0.05 | Very good | (+) |
| Park (2014) [37] | 93 | Spearman correlations of PDQ-39 (SI / Mobility / ADL / Emotional wellbeing / Stigma / Social support / Cognition / Communication / Bodily discomfort) with H&YS: 0.61 ** / 0.65 ** / 0.61 ** / 0.54 * / 0.10 † / 0.20 † / 0.53 ** / 0.50 ** / 0.24 *  Statistical significance: (*) p < 0.05; (**) p < 0.01; (†) No significance | Very good | (+) |
| Fereshtehnejad (2014) [38] | 114 | **PDQ-39:**  Spearman correlations of PDQ-39 with other measures (age / duration of PD / UPDRS-Mental / UPDRS-ADL / UPDRS-Motor / UPDRS-Complications / UPDRS-Total / H&YS / S&E / Daily dose of levodopa / duration of the treatment with levodopa): 0.78 [p = 407] / 0.396 [p < 0.001] / 0.752 [p < 0.001] / 0.653 [p < 0.001] / 0.447 [p < 0.001] / 0.275 [p = 0.003] / 0.635 [p < 0.001] / 0.442 [p < 0.001] / -0.598 [p < 0.001] / 0.313 [p = 0.001] / 0.302 [p = 0.001] | Very good | (+) |
|  | – | **PDQ-8:**  Spearman correlations of PDQ-8 with other measures:   - Age = 0.024 [p = 0.803] - Duration of PD (years) = 0.342 [p < 0.001] - UPDRS-Mental = 0.607 [p < 0.001] - UPDRS-ADL = 0.613 [p < 0.001] - UPDRS-Motor = 0.386 [p < 0.001] - UPDRS-Complications = 0.288 [p = 0.002] - UPDRS-Total = 0.591 [p < 0.001] - H&YS = 0.376 [p < 0.001] - S&E = -0.503 [p < 0.001] - Daily dose of levodopa (mg) = 0.270 [p = 0.004] - Duration of the treatment with levodopa (years) = 0.258 [p = 0.006] | – | – |
| Krygowska-Wajs (2015) [39] | 119 | Correlation between PDQ-39 and PDQ-8 = 0.72 [p < 0.0001] | Very good | (+) |
| Morley (2015, a) [40] | 118 | Pearson correlation of PDQ-39 with duration of PD / age at diagnosis of PD = 0.27 [p < 0.01] / -0.27 [p < 0.01] | Inadequate | (+) |
| Morley (2015, b) [41] | – | – | – | – |
| Jesus-Ribeiro (2017) [42] | 100 | Spearman correlations of PDQ-39 dimensions with SF-36 dimensions:   - PDQ-39 (Mobility): Physical functioning = -0.77 / Physical role = -0.56 / Pain = -0.39 / General health perception = -0.37 / Energy = -0.45 / Social functioning = -0.30 / Emotional role = -0.45 / Mental health = -0.33 - PDQ-39 (ADL): Physical functioning = -0.64 / Physical role = -0.49 / Pain = -0.29 / General health perception = -0.44 / Energy = -0.41 / Social functioning = -0.31 / Emotional role = -0.48 / Mental health = -0.33 - PDQ-39 (Emotional wellbeing): Physical functioning = -0.36 / Physical role = -0.36 / Pain = -0.42 / General health perception = -0.39 / Energy = -0.52 / Social functioning = -0.37 / Emotional role = -0.46 / Mental health = -0.74 - PDQ-39 (Stigma): Physical functioning = -0.38 / Physical role = -0.32 / Pain = -0.23 / General health perception = -0.44 / Energy = -0.31 / Social functioning = -0.30 / Emotional role = -0.39 / Mental health = -0.43 - PDQ-39 (Social support): Physical functioning = 0.03 / Physical role = -0.05 / Pain = -0.19 / General health perception = -0.06 / Energy = -0.06 / Social functioning = -0.21 / Emotional role = -0.02 / Mental health = -0.28 - PDQ-39 (Cognition): Physical functioning = -0.36 / Physical role = -0.44 / Pain = -0.13 / General health perception = -0.12 / Energy = -0.41 / Social functioning = -0.10 / Emotional role = -0.48 / Mental health = -0.34 - PDQ-39 (Communication): Physical functioning = -0.47 / Physical role = -0.41 / Pain = -0.23 / General health perception = -0.39 / Energy = -0.38 / Social functioning = -0.33 / Emotional role = -0.49 / Mental health = -0.42 - PDQ-39 (Bodily discomfort): Physical functioning = -0.33 / Physical role = -0.38 / Pain = -0.40 / General health perception = -0.31 / Energy = -0.38 / Social functioning = -0.24 / Emotional role = -0.36 / Mental health = -0.40   Spearman correlations of PDQ-39 with PDQL and H&YS. | Very good | (+) |
|  | 100 | Spearman correlations of PDQL dimensions with SF-36 dimensions:   - Parkinsonian symptoms: Physical functioning = 0.70 / Physical role = 0.61 / Pain = 0.38 / General health perception = 0.40 / Vitali Energy dad = 0.50 / Social functioning = 0.32 / Emotional role = 0.54 / Mental health = 0.38 - Systemic symptoms: Physical functioning = 0.57 / Physical role = 0.63 / Pain = 0.49 / General health perception = 0.40 / Energy = 0.59 / Social functioning = 0.37 / Emotional role = 0.56 / Mental health = 0.48 - Emotional functioning: Physical functioning = 0.37 / Physical role = 0.54 / Pain = 0.38 / General health perception = 0.45 / Energy = 0.48 / Social functioning = 0.36 / Emotional role = 0.59 / Mental health = 0.57 - Social functioning: Physical functioning = 0.69 / Physical role = 0.55 / Pain = 0.40 / General health perception 0.38 / Energy = 0.44 / Social functioning = 0.39 / Emotional role = 0.50 / Mental health = 0.31 | Very good | (+) |
| Galeoto (2018) [43] | 104 | Spearman correlation between PDQ-39 and SF-36 = -0.5 [p < 0.01] | Very good | (+) |
| Suratos (2018) [44] |  | Pearson correlations of PDQ-39 (Mobility / ADL / Emotional wellbeing / Stigma / Social support / Cognition / Communication / Bodily discomfort / SI) with H&YS: 0.5118 ** / 0.6415 ** / 0.3097 ** / 0.0267 † / 0.1961 † / 0.2496 * / 0.2553 * / 0.3490 * / 0.4630 **  Pearson correlations of PDQ-39 with age, duration of PD (years), daily dose of levodopa (mg), UPDRS-Part 1, UPDRS-Part 2, UPDRS-Part 3, HADS-A, HADS-D and NMSS.  Statistical significance: (*) p < 0.05; (**) p < 0.01; (†) No significance | Very good | (+) |
| Holden (2019) [45] | 201 | **PDQ-39:**  Spearman correlations of McGill QOL with:   - McGill QOL = -0.58 - PROMIS-29 = 0.82 - QOL-AD = -0.58 - ESAS-PD = 0.65 - HADS-D = -0.60   Statistical significance: p < 0.001 for all correlations | Very good | (+) |
|  | 201 | **McGill QOL:**  Spearman correlations of McGill QOL with:   - PDQ-39 = -0.58 - PROMIS-29 = -0.62 - QOL-AD = 0.64 - ESAS-PD = -0.65 - HADS-D = -0.64   Statistical significance: p < 0.001 for all correlations | Very good | (+) |
|  | 201 | **PROMIS-29:**  Spearman correlations of PROMIS-29 with:   - PDQ-39 = 0.82 - QOL-AD = -0.59 - McGill QOL = -0.62 - ESAS-PD = 0.69 - HADS-D = 0.64   Statistical significance: p < 0.001 for all correlations | Very good | (+) |
|  | 201 | **QOL-AD:**  Spearman correlations of PROMIS-29 with:   - PDQ-39 = -0.58 - PROMIS-29 = -0.59 - McGill QOL = 0.64 - ESAS-PD = -0.55 - HADS-D = -0.58   Statistical significance: p < 0.001 for all correlations | Very good | (+) |
| Nelson (2020) [46] | 416 | Spearman correlations of PDQ-39 with UDPRS-Part 3 and PDSQ = 0.22 [p < 0.001] / 0.64 [p < 0.001] | Very good | (+) |
| Kim (2020) [47] | 80 | **PDQ-39:**  Spearman correlations of PDQ-39 with PDQ-8 (Mobility / ADL / Emotional wellbeing / Social support / Cognition / Communication / Bodily discomfort / Stigma / SI) [p < 0.001]: 0.62 / 0.73 / 0.55 / 0.68 / 0.61 / 0.70 / 0.58 / 0.67 / 0.91  Spearman correlations of PDQ-39 with other measures (daily dose of levodopa / H&YS / UPDRS-Motor / MMSE / MoCA / GDS / CDR): 0.32 [p = 0.0111] / 0.38 [p = 0.005] / 0.50 [p = 0.003] / -0.33 [p = 0.018] / -0.50 [p = 0.004] / 0.37 [p = 0.010] / 0.49 [p = 0.001] | Very good | (+) |
|  | 80 | **PDQ-8:**  Spearman correlations of PDQ-8 (nested with PDQ-39) with other measures:   - PDQ-8 [p < 0.001]: Item 1 = 0.66 / Item 2 = 0.76 / Item 3 = 0.57 / Item 4 = 0.70 / Item 5 = 0.62 / Item 6 = 0.70 / Item 7 = 0.57 / Item 8 = 0.70 / Total score = 0.94 - Daily dose of Levodopa (mg) = 0.32 [p = 0.011] - H&YS = 0.44 [p = 0.001] - UPDRS-Motor = 0.51 [p = 0.002] - MMSE = -0.32 [p = 0.024] - MoCA = -0.49 [p = 0.005] - GDS = 0.38 [p = 0.010] - CDR = 0.46 [p = 0.002]   Spearman correlations of PDQ-8 (independent to PDQ-39) with other measures:   - PDQ-8 [p < 0.001]: Item 1 = 0.73 / Item 2 = 0.79 / Item 3 = 0.60 / Item 4 = 0.76 / Item 5 = 0.69 / Item 6 = 0.70 / Item 7 = 0.58 / Item 8 = 0.74 - Daily dose of Levodopa (mg) = 0.28 [p = 0.028] - H&YS = 0.44 [p = 0.001] - UPDRS-Motor = 0.45 [p = 0.007] - MMSE = -0.28 [p = 0.047] - MoCA = -0.49 [p = 0.006] - GDS = 0.38 [p = 0.025] - CDR = 0.46 [p = 0.007] | Very good | (+) |
| Hanff (2023) [48] | 253-677 | Spearman correlations of PDQ-39 with other measures (Postural Instabilities and Gait Difficulty [PIGD] / Timed Up and Go [TUG] / UPDRS-Part 2 / UPDRS-Part 3) [p < 0.001]: 0.69 / 0.45 / 0.77 / 0.45 | Very good | (+) |
| Katsarou (2004) [49] | 228 | Pearson correlations of PDQ-8 with SF-36 dimensions (Physical functioning / Pain / Energy) = -0495 / -0.419 / -0.464  Spearman correlations of PDQ-8 with other measures:   - Age (years) = -0.106 - Duration of PD (years) = 0.269 - UPDRS (On / Off) = 0.510 / 0.493 - ADL (On / Off) = -0.528 / -0.457 - BDI = 0.577 | Very good | (+) |
| Tan (2007) [50] | – | – | – | – |
|  | – | – | – | – |
|  | – | – | – | – |
| Jenkinson (2007) [51] | 178 | Spearman correlations of PDQ-8 with H&YS and PDQ-39 = 0.51 / 0.96 [p < 0.001] | Very good | (+) |
|  | 119 | Spearman correlations of PDQ-8 with H&YS and PDQ-39 = 0.51 / 0.96 [p < 0.001] | Very good | (+) |
|  | 195 | Spearman correlations of PDQ-8 with H&YS and PDQ-39 = 0.47 / 0.96 [p < 0.001] | Very good | (+) |
|  | 194 | Spearman correlations of PDQ-8 with H&YS and PDQ-39 = 0.61 / 0.93 [p < 0.001] | Very good | (+) |
|  | 96 | Spearman correlations of PDQ-8 with H&YS and PDQ-39 = 0.47 / 0.97 [p < 0.001] | Very good | (+) |
| Franchignoni (2008) [52] | – | – | – | – |
|  | – | – | – | – |
|  | 200 | Spearman correlations of PDQ-8 with other measures (IPA-I, H&YS, UPDRS-ADL) = 0.67 / 0.38 / 0.40 |  |  |
| Dal Bello-Haas (2009) [53] | – | – | – | – |
| Alvarado-Bolaños (2015) [54] | 585 | **PDQ-8:**  Pearson correlations of PDQ-8 (Mobility / ADL / Emotional wellbeing / Social support / Cognition / Communication / Bodily discomfort / Stigma / Total score) with other measures:   - H&YS = 0.48** / 0.44** / 0.31** / 0.21** / 0.18** / 0.22** / 0.35** / 0.17** / 0.46** - MDS-UPDRS I = 0.47** / 0.47** / 0.61** / 0.40** / 0.47** / 0.54** / 0.49** / 0.44** / 0.72** - MDS-UPDRS II = 0.65** / 0.71** / 0.48** / 0.42** / 0.41** / 0.45** / 0.27** / 0.35** / 0.76** - MDS-UPDRS III = 0.43** / 0.47** / 0.33** / 0.31** / 0.27** / 0.19** / 0.36** / 0.19** / 0.49** - MDS-UPDRS IV = 0.37** / 0.32** / 0.23** / 0.23** / 0.15** / 0.09* / 0.26** / 0.18** / 0.34** - MDS-UPDRS Total = 0.61** / 0.64** / 0.52** / 0.44** / 0.42** / 0.40** / 0.53** / 0.35** / 0.74** - Pearson correlations of PDQ-8 with EQ-5D-5L (Utility / EQ-VAS) = -0.75** / -0.56**   Pearson correlations of EQ-5D-5L dimensions with PDQ-8 (Mobility / ADL / Emotional wellbeing / Social support / Cognition / Communication / Bodily discomfort / Stigma):   - Mobility = 0.60** / 0.54** / 0.36** / 0.27** / 0.24** / 0.32** / 0.40** / 0.28** - Self-care = 0.54** / 0.78** / 0.42** / 0.35** / 0.34** / 0.36** / 0.44** / 0.26** - Usual activities = 0.58** / 0.62** / 0.41** / 0.39** / 0.36** / 0.42** / 0.46** / 0.28** - Pain - Discomfort = 0.34** / 0.25** / 0.33** / 0.30** / 0.25** / 0.32** / 0.25** / 0.54** - Anxiety - Depression = 0.37** / 0.32** / 0.71** / 0.42** / 0.37** / 0.40** / 0.34** / 0.27**   Spearman correlation of EQ-5D-5L with EQ-VAS = 0.54**  Statistical significance: (*) p < 0.05; (**) p < 0.001 | Very good | (+) |
|  | 585 | **EQ-5D-5L:**  Pearson correlations of EQ-5D-5L (Mobility / Usual activities / Self-care / Pain – Discomfort / Anxiety – Depression / Utility / EQ-VAS) with:   - Duration of PD (years) = 0.22* / 0.21** / 0.19** / 0.51 / 0.03 / -0.17** / -0.10** - H&YS = 0.52** / 0.44** / 0.41** / 0.20** / 0.28** / -0.46** / -0.36** - UPDRS-Part I = 0.50** / 0.50** / 0.52** / 0.48** / 0.59** / -0.65** / -0.52** - UPDRS-Part II = 0.66** / 0.72** / 0.70** / 0.37** / 0.44** / -0.72** / -0.52** - UPDRS-Part III = 0.43** / -0.29** / -0.32** / 0.16** / 0.17** / -0.31** / -0.25** - UPDRS-Part IV = 0.34** / 0.29** / 0.32** / 0.16** / 0.17** / -0.31** / -0.25** - UPDRS-Total = 0.62** / 0.66** / 0.65** / 0.38** / 0.48** / -0.70** / -0.54**   Pearson correlations of PDQ-8 with EQ-5D-5L (Utility / EQ-VAS) = -0.75** / -0.56**  Pearson correlations of EQ-5D-5L dimensions with PDQ-8 (Mobility / ADL / Emotional wellbeing / Social support / Cognition / Communication / Bodily discomfort / Stigma):   - Mobility = 0.60** / 0.54** / 0.36** / 0.27** / 0.24** / 0.32** / 0.40** / 0.28** - Self-care = 0.54** / 0.78** / 0.42** / 0.35** / 0.34** / 0.36** / 0.44** / 0.26** - Usual activities = 0.58** / 0.62** / 0.41** / 0.39** / 0.36** / 0.42** / 0.46** / 0.28** - Pain - Discomfort = 0.34** / 0.25** / 0.33** / 0.30** / 0.25** / 0.32** / 0.25** / 0.54** - Anxiety - Depression = 0.37** / 0.32** / 0.71** / 0.42** / 0.37** / 0.40** / 0.34** / 0.27**   Spearman correlation of EQ-5D-5L with EQ-VAS = 0.54**  Statistical significance: (*) p < 0.05; (**) p < 0.001 | Very good | (+) |
|  | 585 | **EQ-VAS:**  Pearson correlations of EQ-VAS with:   - Duration of PD (years) = -0.10** - H&YS = -0.36** - UPDRS-Part I = -0.52** - UPDRS-Part II = -0.52** - UPDRS-Part III = -0.25** - UPDRS-Part IV = -0.25** - UPDRS-Total = -0.54** - PDQ-8 SI = -0.56** - EQ-5D-5L Utility ~ EQ-VAS = 0.54**   Statistical significance: (*) p < 0.05; (**) p < 0.001 | Very good | (+) |
| Kahraman (2018) [55] | 83 | Spearman correlations of PDQ-8 with SF-36 Physical Component, Mental Component and H&YS [p < 0.001] = -0.52 / -0.64 / 0.56 | Very good | (+) |
| Ramadhan (2022) [56] | 401 | **PDQ-8:**  Correlation of EQ-5D-3L (Mobility / Usual activities / Self-care / Pain – Discomfort / Anxiety – Depression) with PRO / test:   - EQ-5D-3L = -0.43 - EQ-VAS = -0.32 - MMSE = -0.25   Correlation of EQ-5D-3L (Mobility / Usual activities / Self-care / Pain – Discomfort / Anxiety – Depression) with ClinRO:   - H&YS = 0.15 - UPDRS-1 = 0.46 - UPDRS-2 = 0.41 - UPDRS-3 = 0.33 - UPDRS-4 = 0.17 - S&E = -0.30   Correlation of EQ-5D-3L (Mobility / Usual activities / Self-care / Pain – Discomfort / Anxiety – Depression) with ObsRO:   - Zarit = 0.29 - Carer EQ-5D-3L = -0.02 - Carer EQ-VAS = -0.03 | Very good | (+) |
|  | 401 | **EQ-5D-3L:**  Correlation of EQ-5D-3L (Mobility / Usual activities / Self-care / Pain – Discomfort / Anxiety – Depression) with PRO / test:   - PDQ-8 = -0.43 [p < 0.001] - MMSE = 0.23 [p < 0.001]   Correlation of EQ-5D-3L (Mobility / Usual activities / Self-care / Pain – Discomfort / Anxiety – Depression) with ClinRO:   - H&YS = -0.36 - UPDRS-Part I = -0.28 - UPDRS-Part II = -0.48 - UPDRS-Part III = -0.42 - UPDRS-Part IV = -0.01 - S&E = 0.45   Correlation of EQ-5D-3L (Mobility / Usual activities / Self-care / Pain – Discomfort / Anxiety – Depression) with ObsRO:   - Zarit = -0.09 - Carer EQ-5D-3L = -0.01 - Carer EQ-VAS = -0.01 | Very good | (+) |
| Stathis (2022) [57] | 60 | **PDQ-8:**  Spearman correlations of PDQ-8 with other measures [p < 0.01]:   - Time since PD diagnosis (years) = 0.539 - H&YS = 0.552 - Cognitive impairment = 0.570 - Disability-Off Index = 0.669 - Disability -LID Index = 0.334 - Non-Motor Questionnaire = 0.540 - Depressive status = 0.593 - EQ-5D-5L = -0.789 | Very good | (+) |
|  | 60 | **PDQoL-7:**  Spearman correlations of PDQoL-7 with other measures [p < 0.01]:   - Time since PD diagnosis (years) = 0.528 - H&YS = 0.561 - Cognitive impairment = 0.575 - Disability-Off Index = 0.682 - Disability -LID Index = 0.278 - Non-Motor Questionnaire = 0.681 - Depressive status = 0.650 - EQ-5D-5L = -0.582 | Very good | (+) |
| Kawaguchi (2021) [58] | – | – | – | – |
| De Boer (1996) [59] | 384 | Pearson correlations of PDQL with CES-D (Physical function / Role function / Health perception / Energy-Fatigue / Bodily pain / Social function / Social support / Mental health / Depression):   - Parkinsonian symptoms: 0.50 / 0.48 / 046 / 0.50 / -0.22 / - / - / - / - - Systemic symptoms: 0.48 / 0.46 / 0.52 / 0.64 / -0.36 / - / - / - / - - Emotional functioning: - / - / - / - / - / 0.43 / 0.13 / - / - - Social functioning: - / - / - / - / - / - / - / 0.66 / -0.79   Pearson correlations of PDQL with MOS-24.  Statistical significance: p < 0.001 if r > 0.19 | Very good | (+) |
| Serrano-Dueñas (2004) [60] | 137 | Pearson correlations of PDQL with PDQ-39 SI = -0.91 [p < 0.0001]  Pearson correlations of PDQL with other measures (H&YS / S&E / UPDRS-I / UPDRS-II / UPDRS-III / UPDRS-Total / HADS-A / HADS-D):   - Parkinsonian symptoms: -0.64 / 0.67 / -0.60 / -0.77 / -0.52 / -0.70 / -0.40 / -0.53 - Systemic symptoms: -0.57 / 0.61 / -0.40 / -0.58 / -0.40 / -0.51 / -0.34 / -0.45 - Emotional functioning: -0.59 / 0.55 / -0.43 / -0.73 / -0.40 / -0.60 / -0.50 / -0.54 - Social functioning: -0.25 / 0.30 / -0.29 / -0.38 / -0.24 / -0.32 / -0.70 / -0.74 - Total: -0.62 / 0.65 / -0.55 / -0.76 / -0.48 / -0.66 / -0.55 / -0.67   Statistical significance: All p < 0.003 (most of them < 0.001) | Very good | (+) |
| Campos (2011) [61] | 58 | Spearman correlations:   - PDQL (Systemic symptoms) with UPDRS-Part 1 = 0.78 [p-Value not specified] - PDQL (Total) with UPDRS-Part 1 = 0.59 [p < 0.01] - PDQL (Total) with BDI = 0.57 [p < 0.01] | Very good | (+) |
| Dereli (2015) [62] | 89 | Pearson correlations of PDQL (Parkinsonian symptoms / Systemic symptoms / Emotional functioning / Social functioning / Total) with other measures:   - H&YS = -0.73** / -0.45* / -0.46* / -0.55** / -0.64** - UPDRS-I = -0.49** / -0.55** / -0.56** / -0.63** / -0.60** - UPDRS-II = -0.72** / -0.53** / -0.44** / -0.56** / -0.64** - UPDRS-III = -0.72** / -0.56** / -0.42* / -0.56** / -0.64** - UPDRS-Total = -0.79** / -0.63** / -0.51** / -0.65** / -0.73**   Statistical significance: (*) p < 0.05; (**) p < 0.01 [Two tailored] | Very good | (+) |
| Welsh (2003) [63] | 233 | Pearson correlations of PDQUALIF (Social-Role lie / Self-image-Sexuality / Sleep / Outlook / Physical function / Independence / Urinary function) with other measures:   - UPDRS-Total = 0.43** / 0.39** / 0.32** / 0.14** / 0.03 / 0.32** / 0.46** / 0.26** - UPDRS-Part1 = 0.39** / 0.28** / 0.37** / 0.10 / 0.16* / 0.30** / 0.28** / 0.22** - UPDRS-Part 2 = 0.31** / 0.27** / 0.18** / 0.06 / 0.02 / 0.23** / 0.37** / 0.16* - UPDRS-Part 3 = 0.55** / 0.50** / 0.44** / 0.26** / 0.03 / 0.40** / 0.47** / 0.33** - SF-36 (Mental) = -0.48** / -0.44** / -0.41** / -0.21** / -0.49** / -0.31** / -0.19** / -0.17* - SF-36 (Physical) = -0.52** / -0.60** / -0.34** / -0.24** / -0.21** / -0.37** / -0.26** / -0.29** - SIP-Total = 0.73** / 0.68** / 0.52** / 0.29** / 0.35** / 0.57** / 0.55** / 0.29** - SIP-Physical = 0.61** / 0.59** / 0.36** / 0.25** / 0.22** / 0.48** / 0.55** / 0.28** - SIP-Psychosocial = 0.70** / 0.62** / 0.61** / 0.27** / 0.43** / 0.52** / 0.42** / 0.31** - Age (years) = 0.03 / 0.03/ -0.22** / 0.02 / -0.21** / 0.10 / 0.11 / 0.21** - Education (years) = -0.15* / 0.09 / 0.03 / -0.12 / -0.12 / -0.09 / -0.17** / -0.09 - Time since PD diagnosis (years) = 0.32** / 0.35** / 0.32** / 0.18** / 0.08 / 0.20** / 0.19** / 0.07   Statistical significance: (*) p < 0.05; (**) p < 0.01 | Very good | (+) |
| Calne (1996) [64] | – | – | – | – |
| Schulzer (2003) [65] | 116 | Correlations of PIMS with other PROMs (stable / fluctuations):   - UDPRS-Part 2 = 0.55 [p < 0.0001] / 0.39 [p < 0.0001] - UPDRS-Part 3 = 0.33 [p = 0.003] / 0.55 [p < 0.0001] - UPDRS-Part 4 = 0.25 [p = 0.0071] / 0.32 [p = 0.0013] - S&E = 0.48 [p < 0.0001] / 0.47 [p < 0.0001] | Very good | (+) |
| Aggarwal (2020) [66] | 295 | Spearman correlations of QLPD with other measures:   - H&YS (Regression): β = 14.7 / 95CI = 11.09 - 18.34 * - Age at diagnosis of PD (Regression): β = -0.23 / 95CI = -0.41 – (-0.06) - Duration of the PD symptoms (Regression): β = 0.88 / 95CI = 0.53 - 1.22 - Monthly earnings (Regression): β = -0.68 / IC95 = -0.89 – (-0.47) - Equivalent daily dose of levodopa (Regression): β = 0.03 / 95CI = 0.02 - 0.04   Spearman correlations of QLPD with PDQ-39 (ADL / Emotional wellbeing / Stigma / Social support / Cognition / Communication / Bodily discomfort):   - ADL: 0.72 / 0.71 / 0.57 / 0.29 / 0.28 / 0.51 / 0.59 / 0.33 - Mobility: 0.65 / 0.50 / 0.52 / 0.26 / 0.19 / 0.47 / 0.51 / 0.26 - Psychological: 0.48 / 0.42 / 0.75 / 0.43 / 0.38 / 0.41 / 0.39 / 0.45 - Fear: 0.45 / 0.29 / 0.63 / 0.56 / 0.43 / 0.44 / 0.39 / 0.41 - Social: 0.51 / 0.40 / 0.59 / 0.65 / 0.41 / 0.49 / 0.53 / 0.32 - Family: 0.64 / 0.60 / 0.63 / 0.45 / 0.43 / 0.50 / 0.48 / 0.42 - Treatment: 0.34 / 0.17 / 0.37 / 0.28 / 0.17 / 0.30 / 0.25 / 0.37 - Finances: 0.32 / 0.24 / 0.41 / 0.35 / 0.35 / 0.36 / 0.22 / 0.31 - Nonmotor symptoms: 0.68 / 0.58 / 0.61 / 0.24 / 0.29 / 0.65 / 0.57 / 0.49 - Total: 0.76 / 0.64 / 0.78 / 0.49 / 0.43 / 0.66 / 0.62 / 0.54 - General health: -0.64 / -0.52 / -0.56 / -0.37 / -0.27 / -0.48 / -0.44 / -0.40   Spearman correlations of QLPD with SF-36 (Physical functioning / Role physical / Pain / General health / Energy / Social functioning / Role emotional / Metal health / Physical component / Mental component):   - ADL: -0.50 / -0.38 / -0.50 / -0.32 / -0.46 / -0.57 / -0.33 / -0.36 / -0.51 / -0.41 - Mobility: -0.53 / -0.40 / -0.45 / -0.25 / -0.41 / -0.57 / -0.37 / -0.36 / -0.49 / -0.42 - Psychological: -0.27 / -0.35 / -0.39 / -0.41 / -0.50 / -0.51 / -0.36 / -0.68 / -0.24 / -0.65 - Fear: -0.16 / -0.31 / -0.42 / -0.38 / -0.37 / -0.40 / -0.30 / -0.55 / -0.24 / -0.52 - Social: -0.22 / -0.20 / -0.47 / -0.34 / -0.43 / -0.53 / -0.23 / -0.46 / -0.28 / -0.46 - Family: -0.31 / -0.37 / -0.51 / -0.33 / -0.40 / -0.62 / -0.34 / -0.43 / -0.40 / -0.51 - Treatment: -0.34 / -0.25 / -0.31 / -0.27 / -0.41 / -0.38 / -0.24 / -0.27 / -0.35 / -0.32 - Finances: -0.11 / -0.20 / -0.27 / -0.23 / -0.37 / -0.33 / -0.35 / -0.33 / -0.13 / -0.44 - Nonmotor symptoms: -0.42 / -0.46 / -0.52 / -0.44 / -0.44 / -0.49 / -0.42 / -0.50 / -0.49 / -0.54 - Total: -0.48 / -0.49 / -0.64 / -0.47 / -0.59 / -0.69 / -0.48 / -0.63 / -0.54 / -0.67 - General health l: 0.41 / 0.30 / 0.33 / 0.40 / 0.51 / 0.41 / 0.32 / 0.33 / 0.41 / 0.41   Statistical significance: (*) p < 0.05; the remaining p < 0.0001 | Very good | (+) |
| Kuehler (2003) [67] | – | **QLSM-DBS:**  Spearman correlations of QLSM-DBS with other PROMs (SF-36 Physical Component / SF-36 Mental Component / EQ-5D Utility / EQ-VAS / QLSM-A [General satisfaction] / QLSM-G [Health satisfaction] / QLSM-MD) = 0.32 / 0.36 / 0.46 / 0.34 / 0.50 / 0.62 / 0.65 | Very good | (+) |
|  | – | **QLSM-MD:**  Spearman correlations of QLSM-MD with other PROMs (SF-36 Physical Component / SF-36 Mental Component / EQ-5D Utility / EQ-VAS / QLSM-A [General satisfaction] / QLSM-G [Health satisfaction] / QLSM-MD) = 0.59 / 0.63 / 0.68 / 0.49 / 0.53 / 0.75 / 0.65 | Very good | (+) |
| Krygowska-Wajs (2015) [68] | 30 | **QLSM-DBS:**  Good convergent validity was observed for QLSM-DBS | Doubtfull | (?) |
|  | 30 | **QLSM-MD:**  Good convergent validity was observed for QLSM-MD | Doubtfull | (?) |
| Bose (2018) [69] | 120 | Pearson correlations of QoLQ-PwP with PDQ-39 and PDQ-8 [p < 0.01] = 0.908 / 0.862 | Very good | (+) |
| Diniz (2018) [70] | – | – | – | – |
| García-Gordillo (2013) [71] | 133 | **15D:**  Spearman correlations between dimensions of PDQ-8 and 15D (Utility / Mobility / Vision / Earing / Breathing / Sleeping / Eating / Speech / Elimination / Usual activities / Mental function / Discomfort and symptoms / Depression / Distress / Vitality / Sexual activity):   - PDQ-8 (Mobility) = -0.428** / -0.502** / -0.374** / -0.212* / -0.135 / -0.078 / -0.448** / -0.382** / -0.322** / -0.441** / -0.211* / -0.336** / -0.790* / -0.157 / -0.380** / -0.180* - PDQ-8 (ADL) = -0.501** / -0.502** / -0.386** / -0.182* / -0.239** / -0.208* / -0.432** / -0.290** / -0.430** / -0.528** / -0.236** / -0.381** / -0.299** / -0.291** / -0.435** / -0.260** - PDQ-8 (Emotional wellbeing) = -0.539** / -0.318** / -0.237** / -0.285** / -0.283** / -0.306** / -0.401** / -0.193* / -0.366** / -0.464** / -0.334** / -0.346** / -0.749** / -0.568** / -0.509** / -0.414** - PDQ-8 (Stigma) = -0.295** / -0.136 / -0.111 / -0.136 / -0.207* / -0.117 / -0.288** / -0.266** / -0.134 / -0.238** / -0.161 / -0.184* / -0.246** / -0.351** / -0.337** / -0.096 - PDQ-8 (Social support) = -0.448** / -0.329** / -0.345** / -0.209* / -0.300** / -0.178* / -0.355** / -0.303** / -0.309** / -0.360** / -0.265** / -0.315** / -0.361** / -0.232** / -0.423** / -0.417** - PDQ-8 (Cognition) = -0.569** / -0.483** / -0.404** / -0.313** / -0.370** / -0.276** / -0.210* / -0.282** / -0.390** / -0.333** / -0.543** / -0.418** / -0.389** / -0.410** / -0.442** / -0.372** - PDQ-8 (Communication) = -0.473** / -0.355** / -0.362** / -0.392** / -0.358** / -0.193* / -0.309** / -0.543** / -0.333** / -0.319** / -0.432** / -0.200* / -0.315** / -0.265** / -0.307** / -0.262** - PDQ-8 (Bodily discomfort) = -0.498** / -0.403** / -0.337** / -0.138 / -0.267** / -0.327** / -0.303** / -0.172* / -0.365** / -0.357** / -0.265** / -0.454** / -0.400** / -0.300** / -0.470** / -0.235** - PDQ-8 (SI) = -0.710** / -0.564** / -0.458** / -0.296** / -0.371** / -0.301** / -0.492** / -0.449** / -0.496** / -0.576** / -0.431** / -0.498** / -0.564** / -0.510** / -0.623** / -0.429**   Correlations of EQ-VAS and 15D (Utility / Mobility / Vision / Earing / Breathing / Sleeping / Eating / Speech / Elimination / Usual activities / Mental function / Discomfort and symptoms / Depression / Distress / Vitality / Sexual activity) = 0.542** / -0.502** / -0.355** / -0.152 / -0.294** / -0.258** / -0.345** / -0.260** / -0.422** / -0.583** / -0.164 / -0.427** / -0.370** / -0.333** / -0.527** / -0.309**  Statistical significance: (*) p < 0.05; (**) p < 0.001 [Two-tailored] | Very good | (+) |
|  | 133 | **EQ-5D-5L:**  Spearman correlations between dimensions of PDQ-8 and EQ-5D-5L (Utility / Mobility / Self-care / Usual activities / Pain – Discomfort / Anxiety – Depression):   - PDQ-8 (Mobility) = -0.496** / 0.587** / 0.534** / 0.554** / 0.285** / 0.195* - PDQ-8 (ADL) = -0.612** / 0.573** / 0.688** / 0.594** / 0.403** / 0.338** - PDQ-8 (Emotional wellbeing) = -0.516** / 0.331** / 0.389** / 0.376** / 0.410** / 0.705** - PDQ-8 (Stigma) = -0.283** / 0.274 / 0.320** / 0.332** / 0.162 / 0.324** - PDQ-8 (Social support) = -0.344** / 0.374** / 0.385** / 0.298 / 0.280** / 0.302** - PDQ-8 (Cognition) = -0.458** / 0.411** / 0.356** / 0.394** / 0.345** / 0.397** - PDQ-8 (Communication) = -0.315** / 0.358** / 0.342** / 0.325** / 0.138 / 0.273** - PDQ-8 (Bodily discomfort) = -0.470** / 0.318** / 0.365** / 0.350** / 0.473** / 0.448** - PDQ-8 SI = -0.679** / 0.605** / 0.614** / 0.602** / 0.500** / 0.582**   Correlations of EQ-VAS and EQ-5D-5L (Mobility / Self-care / Usual activities / Pain – Discomfort / Anxiety – Depression) = 0.609** / -0.531** / -0.509** / -0.591** / -0.507** / -0.448**  Statistical significance: (*) p < 0.05; (**) p < 0.001 [Two-tailored] | Very good | (+) |
| Del Pozo-Cruz (2018) [72] | 229 | **15D:**  Spearman correlation between every dimension of 15D and PDQ-8 (SI / Mobility / ADL / Emotional wellbeing / Stigma / Social support / Cognition / Communication / Bodily discomfort):   - 15D Utility: -0.532** / -0.587** / -0.578** / -0.162* / -0.430** / -0.563** / -0.505** / -0.461** / -0.758** - Mobility: -0.427** / -0.541** / -0.340** / -0.074 / -0.343** / -0.374** / -0.382** / -0.364** / -0.596** - Vision: -0.381** / -0.390** / -0.314** / 0.024 / -0.300 / -0.409** / -0.322** / -0.287** / -0.463** - Earing: -0.231** / -0.208** / -0.203** / -0.070 / -0.157* / -0.255** / -0.255** / -0.106 / -0.271 ** - Breathing: -0.274** / 0.330** / -0.289** / -0.055 / -0.259** / -0.324** / -0.328** / -0.244** / -0.403* - Sleeping: -0.156* / -0.249** / -0.300** / -0.080 / -0.173** / -0.264** / -0.194** / -0.302** / -0.305** - Eating: -0.463** / -0.503** / -0.381** / -0.154* / -0.357** / -0.254** / -0.436** / -0.304** / -0.557** - Speech: -0.370** / -0.341** / -0.236** / -0.127 / -0.307** / -0.305** / -0.523** / -0.138* / -0.474** - Elimination: -0.267** / -0.326** / -0.238** / -0.069 / -0.241** / -0.299** / -0.302** / -0.292** / -0.408** - Usual activities: -0.484** / -0.600** / -0.405** / -0.115 / -0.345** / -0.344** / -0.373** / -0.336** / -0.612** - Mental function: -0.151* / -0.191** / -0.355** / -0.027 / -0.153* / -0.508** / -0.293** / -0.175** / -0.350** - Discomfort and symptoms: -0.371** / -0.377** / -0.316** / -0.120 / -0.262** / -0.353** / -0.221** / -0.389** / -0.481** - Depression: -0.274** / -0.278** / -0.754** / -0.244** / -0.309** / -0.438** / -0.308** / -0.325** / -0.563** - Distress: -0.275** / -0.341** / -0.635** / -0.283** / -0.273** / -0.469** / -0.281** / -0.296** / -0.560** - Vitality: -0.444** / -0.437 ** / -0.553** / -0.283** / -0.407** / -0.464** / -0.375** / -0.380** / -0.649** - Sexual activity: 0.294** / -0.293** / -0.389** / -0.045 / -0.338** / -0.310* / -0.237** / -0.233** / -0.412**   Statistical significance: (*) p < 0.05; (**) p < 0.001 [Two-tailored] | Very good | (+) |
|  | 229 | **SF-6D:**  Spearman correlations of SF-6D with PDQ-8 (Mobility / ADL / Emotional wellbeing / Stigma / Social support / Cognition / Communication / Bodily discomfort / SI):   - Utility: -0.523** / -0.628** / -0.604** / -0.219** / -0.456** / -0.522** / -0.398** / -0.393** / -0.741** - Physical functioning: 0.532** / 0.724** / 0.454** / 0.198** / 0.397** / 0.372** / 0.352** / 0.301** / 0.665** - Role limitation: 0.393** / -0.447** / 0.557** / 0.237** / 0.312** / 0.435** / 0.292** / 0.288** / 0.581** - Social functioning: 0.358** / 0.340** / 0.493** / 0.271** / 0.356** / 0.375** / 0.283** / 0.223** / 0.527** - Pain: 0.372** / 0.404** / 0.411** / 0.058 / 0.346** / 0.449** / 0.295** / 0.391** / 0.538** - Mental health: 0.237** / 0.300** / 0.588** / 0.262** / 0.257** / 0.401** / 0.254** / 0.219** / 0.476** - Energy: 0.401** / 0.391** / 0.417** / 0.181** / 0.283** / 0.404** / 0.220** / 0.237** / 0.507**   Statistical significance: (*) p < 0.05; (**) p < 0.001 [Two-tailored] | Very good | (+) |
| Luo (2009) [73] | – | **EQ-5D-3L:**  – | – | – |
|  |  | **EQ-VAS:**  – | – | – |
|  | 135 | **EQ-5D-3L:**  Spearman correlation of EQ-5D-3L (Mobility / Usual activities / Self-care / Pain – Discomfort / Anxiety – Depression) with:   - PDQ-8 (Mobility) = 0.52*** / 0.31* / 0.37* / 0.27* / 0.40*** / -0.40** - PDQ-8 (ADL) = 0.44** / 0.44** / 0.36* / 0.48*** / 0.32* / 0.53*** / -0.45** - PDQ-8 (Emotional wellbeing) = 0.23* / 0.16 / 0.36* / 0.32* / 0.53*** / -0.45** - PDQ-8 (Social support) = 0.38* / 0.31* / 0.39** / 0.24* / 0.35* / -0.39** - PDQ-8 (Cognition) = 0.31* / 0.27* / 0.40** / 0.27* / 0.26* / -0.38** - PDQ-8 (Communication) = 0.48*** / 0.39** / 0.55*** / 0.44** / 0.43** / -0.60*** - PDQ-8 (Bodily discomfort) = 0.39** / 0.44** / 0.39** / 0.42** / 0.41** / -0.54*** - PDQ-8 (Stigma) = 0.63*** / 0.39** / 0.56** / 0.38** / 0.55*** / -0.61*** - PDQ-8SI = 0.57*** / 0.45** / 0.56*** / 0.50*** / 0.53*** / -0.67*** - H&YS = 0.43*** / 0.35** / 0.15 / 0.18 / -0.05 / -0.28* - S&E = -0.60*** / -0.47*** / -0.36** / -0.10 / -0.20 / 0.36** - UPDRS-Motor = 0.22 / 0.07 / 0.03 / -0.07 / -0.16 / 0.04 - Duration of PD (years) = 0.37** / 0.30* / 0.32** / 0.28* / 0.17 / -0.40*** / -0.17 - MMSE = -0.09 / -0.20 / 0.09 / 0.03 / 0.17 / -0.01   Statistical significance: (*) p < 0.05; (**) p < 0.01; (***) p < 0.001 | Very good | (+) |
|  |  | **EQ-VAS:**  Spearman correlations of EQ-VAS with:   - PDQ-8 (Mobility) = -0.49*** - PDQ-8 (ADL) = -0.52*** - PDQ-8 (Emotional wellbeing) = -0.45*** - PDQ-8 (Social support) = -0.42*** - PDQ-8 (Cognitio) = -0.52*** - PDQ-8 (Communication) = -0.48*** - PDQ-8 (Bodily discomfort) = -0.48** - PDQ-8 (Stigma) = -0.40*** - PDQ-8 SI = -0.63*** - H&YS = -0.40*** - S&E = -0.41*** - UPDRS-Motor = -0.36*** - Duration of PD (years) = -0.31*** - MMSE = 0.14   Statistical significance: (*) p < 0.05; (**) p < 0.01; (***) p < 0.001 | Very good | (+) |
|  | 71 | **EQ-5D-3L:**  Spearman correlation of EQ-5D-3L (Mobility / Usual activities / Self-care / Pain – Discomfort / Anxiety – Depression) with:   - PDQ-8 (Mobility) = 0.52*** / 0.31* / 0.37* / 0.27* / 0.40*** / -0.40** - PDQ-8 (ADL) = 0.44** / 0.44** / 0.36* / 0.48*** / 0.32* / 0.53*** / -0.45** - PDQ-8 (Emotional wellbeing) = 0.23* / 0.16 / 0.36* / 0.32* / 0.53*** / -0.45** - PDQ-8 (Social support) = 0.38* / 0.31* / 0.39** / 0.24* / 0.35* / -0.39** - PDQ-8 (Cognition) = 0.31* / 0.27* / 0.40** / 0.27* / 0.26* / -0.38** - PDQ-8 (Communication) = 0.48*** / 0.39** / 0.55*** / 0.44** / 0.43** / -0.60*** - PDQ-8 (Bodily discomfort) = 0.39** / 0.44** / 0.39** / 0.42** / 0.41** / -0.54*** - PDQ-8 (Stigma) = 0.63*** / 0.39** / 0.56** / 0.38** / 0.55*** / -0.61*** - PDQ-8 SI = 0.57*** / 0.45** / 0.56*** / 0.50*** / 0.53*** / -0.67*** - H&YS = 0.43*** / 0.35** / 0.15 / 0.18 / -0.05 / -0.28* - S&E = -0.60*** / -0.47*** / -0.36** / -0.10 / -0.20 / 0.36** - UPDRS-Motor = 0.22 / 0.07 / 0.03 / -0.07 / -0.16 / 0.04 - Duration of PD (years) = 0.37** / 0.30* / 0.32** / 0.28* / 0.17 / -0.40*** / -0.17 - MMSE = -0.09 / -0.20 / 0.09 / 0.03 / 0.17 / -0.01   Statistical significance: (*) p < 0.05; (**) p < 0.01; (***) p < 0.001 | Very good | (+) |
|  |  | **EQ-VAS:**  Spearman correlations of EQ-VAS with:   - PDQ-8 (Mobility) = -0.20 - PDQ-8 (ADL) = -0.26* - PDQ-8 (Emotional wellbeing) = -0.28* - PDQ-8 (Social support) = -0.33* - PDQ-8 (Cognition) = -0.25* - PDQ-8 (Communication) = -0.25* - PDQ-8 (Bodily discomfort) = -0.19 - PDQ-8 (Stigma) = -0.25* - PDQ-8 SI = -0.35* - H&YS = -37** - S&E = -0.27* - UPDRS-Motor = -0.19 - Duration of PD (years) = -0.17 - MMSE = 0.11   Statistical significance: (*) p < 0.05; (**) p < 0.01; (***) p < 0.001 | Very good | (+) |
| Garcia-Gordillo (2015) [74] | 133 | **EQ-5D-3L:**  Spearman correlation of EQ-5D-3L (Mobility / Usual activities / Self-care / Pain – Discomfort / Anxiety – Depression) with:   - PDQ-8 (Mobility) = -0.564** / 0.472** / 0.55** / 0.538** / 0.286** / 0.272** - PDQ-8 (ADL) = -0.628** / 0.480** / 0.648** / 0.599** / 0.323** / 0.341** - PDQ-8 (Emotional wellbeing) = -0.548** / 0.324** / 0.429** / 0.408** / 0.378** / 0.687** - PDQ-8 (Stigma) = -352** / 0.198* /0.294** / 0.296** / 0.209* / 0.412** - PDQ-8 (Social support) = -0.376** / 0.276** / 0.339** / 0.320** / 0.262* / 0.317** - PDQ-8 (Cognition) = -0.469** / 0.366** / 0.343** / 0.385** / 0.389** / 0.338** - PDQ-8 (Communication) = -0.340** / 0.229** / 0.338** / 0.327** / 0.128 / 0.293** - PDQ-8 (Bodily discomfort) = -0.469** / 0.274** / 0.333** / 0.321** / 0.524** / 0.381** - PDQ-8 (SI) = -0.721** / 0.506** / 0.614** / 0.599** / 0.500** / 0.585** - EQ-VAS = 0.677** / -0.557** / -0.551** / -0.573** / -0.510** / -0.393**   Statistical significance: (*) p < 0.05; (**) p < 0.001 [Two-tailored] | Very good | (+) |
|  | 133 | **SF-6D:**  Spearman correlation of SF-6D () with:   - PDQ-8 (Mobility) = -0.450** / 0.237** / 0.143 / 0.060 / 0.183* / 0.089 / 0.132 - PDQ-8 (ADL) = -0.612** / 0.142 / 0.097 / 0.006 / 0.223* / 0.174* / 0.107 - PDQ-8 (Emotional wellbeing) = -0.577** / 0.007 / 0.044 / -0.032 / 0.050 / -0.040 / 0.013 - PDQ-8 (Stigma) = -0.309** / 0.118 / 0.122 / 0.078 / 0.215* / 0.023 / 0.111 - PDQ-8 (Social support) = -0.451** / 0.008 / -0.011 / 0.056 / 0.115 / -0.008 / 0.131 - PDQ-8 (Cognition) = -0.532** / 0.110 / 0.108 / 0.163 / 0.100 / 0.134 / 0.179* - PDQ-8 (Communication) = -0.328** / 0.171* / 0.100 / 0.84 / 0.120 / -0.37 / 0.180* - PDQ-8 (Bodily discomfort) = -0.435** / 0.034 / 0.015 / 0.029 / 0.055 / 0.059 / 0.057 - PDQ-8 (SI) = -0.711** / 0.162 / 0.103 / 0.046 / 0.184* / 0.080 / 0.156 - EQ-VAS = 0.535** / -0.060 / -0.007 / -0.16 / -0.137 / -0.159 / -0.255**   Statistical significance: (*) p < 0.05; (**) p < 0.001 [Two-tailored] | Very good | (+) |
| Nowinski (2010) [75] | 121 | Spearman correlations of:   - Neuro-QOL (Anxiety) with PDQ-39 (Emotional wellbeing) = 0.70 - Neuro-QOL (Depression) with PDQ-39 (Emotional wellbeing) = 0.69 - Neuro-QOL (Stigma) with PDQ-39 (Emotional wellbeing) = 0.52 - Neuro-QOL (Positive Affect and Well-Being) with PDQ-39 (Emotional wellbeing) = -0.51 - Neuro-QOL (Upper Extremity Function–Fine Motor, ADL) with PDQ-39 (Mobility / ADL) = -0.75 - Neuro-QOL (Perceived function) with PDQ-39 (Cognitive impairment) = 0.49 - Neuro-QOL (Applied Cognition–Executive Function) with PDQ-39 (Cognitive impairment) = 0.56   No data available regarding statistical significance | Very good | (+) |
| Nowinski (2016) [76] | 120 | Pearson correlations of Neuro-QOL (Positive Affect and Well-Being / Applied Cognition–General Concerns / Applied Cognition–Executive Function / Lower Extremity Function–Mobility / Upper Extremity Function–Fine Motor, ADL / Ability to Participate in Social Roles and Activities / Satisfaction with Social Roles and Activities / Depression / Anxiety / Stigma / Fatigue / Sleep Disturbance / Emotional and Behavioral Dyscontrol) with:   - PDQ-39 - Mobility: -0.47*** / -0.33*** / -0.41*** / -0.68*** / -0.44*** / -0.62*** / -0.56*** / 0.35*** / -0.36*** / 0.45*** / 0.32*** - PDQ-39 - ADL: -0.39*** / -0.38*** / -0.37*** / -0.57*** / -0.70*** / -0.43*** / -0.45*** / 0.36*** / 0.38*** / 0.42*** / 0.43*** - PDQ-39 – Emotional wellbeing: -0.53*** / -0.18 / -0.29** / -0.40*** / -0.37*** / -0.45*** / -0.49*** / 0.68*** / 0.65*** / 0.50*** / 0.50*** - PDQ-39 - Stigma: -0.21* / -0.15 / -0.05 / -0.28** / -0.29** / -0.27** / -0.30** / 0.22* / 0.43*** / 0.46*** / 0.30** - PDQ-39 – Social support: -0.39*** / -0.37*** / -0.31*** / -0.24* / -0.34*** / -0.37*** / -0.45*** / 0.36*** / 0.27** / 0.42*** / 0.42*** - PDQ-39 – Cognitive impairment: -0.42*** / -0.50*** / -0.49*** / -0.36*** / -0.39*** / -0.43*** / -0.38*** / 0.37*** / 0.41*** / 0.36*** / 0.43*** - PDQ-39 - Communication: -0.42*** / -0.39*** / -0.42*** / -0.39*** / -0.37*** / -0.52*** / -0.51*** / 0.38*** / 0.29*** / 0.49*** / 0.333*** - UPDRS-Total: -0.31*** / -0.20 / -0.33** / -0.51*** / -0.23* / -0.27** / -0.30** / 0.11 / 0.10* / 0.13 / 0.05 - UPDRS-Part I: -0.34*** / -0.23* / -0.24** / -0.24* / -0.16 / -0.36*** / -0.23* / 0.29** / 0.27** / 0.13 / 0.19* - UPDRS- Part II: -0.28** / -0.23* / -0.34*** / -0.58*** / -0.43*** / -0.37*** / -0.444 / 0.22* / 0.16 / 0.28** / 0.21* - UPDRS- Part III: -0.12 / -0.04 / -0.18 / -0.30** / -0.02 / -0.08 / -0.09 / -0.03 / -0.02 / 0.02 / -0.08 - MoCA: 0.19* / 0.22* / 0.34*** / 0.08 / 0.09 / 0.22* / 0.21** / -0.13 / -0.03 / -0.13 / -0.10 - PHQ-9: -0.50*** / -0.32*** / -0.24** / -0.33*** / -0.27** / -0.50*** / -0.55*** / 0.47*** / 0.42*** / 0.46*** / 0.33*** - Barthel Index: 0.28** / 0.25** / 0.40*** / 0.57*** / 0.37*** / 0.28** / 0.30*** / -0.32*** / -0.42*** / -0.33*** / -0.39*** / -0.31*** / -0.32*** - Lawton IADL Scale: 0.16 / 0.08 / 0.32*** / 0.10 / 0.27*** / 0.10 / 0.17 / -0.12 / -0.12 / -0.17 / -0.01 / -0.06 / -0.10 - Oral Symbol Digit Modalities (Correct number): 0.16 / 0.27** / 0.42*** / 0.10 / 0.11 / 0.18 / 0.13 / -0.16 / -0.10 / -0.04 / -0.03 / -0.06 / -0.08 - Symbol Search (Raw Score): 0.21* / 0.22* / 0.36*** / 0.04 / 0.05 / 0.23* / 0.16 / -0.10 / - 0.07 / -0.04 / -0.04 / 0.01 / 0.01 - Digit Symbol Coding (Correct number): 0.13 / 0.19* / 0.41*** / 0.10 / 0.05 / 0.17 / 0.16 / -0.08 / -0.05 / -0.04 / 0.02 / 0.01 / 0.06 - PROMIS (Global - Physical): 0.42*** / 0.29** / 0.38*** / 0.55*** / 0.41*** / 0.52*** / 0.43*** / -0.36*** / -0.43*** / -0.41*** / -0.63*** / -0.51*** / -0.37*** - PROMIS (Global - Mental): 0.73*** / 0.41*** / 0.40*** / 0.33*** / 0.37*** / 0.62*** / 0.59*** / -0.66*** / -0.60*** / -0.50*** / -0.47*** / -0.41*** / -0.41*** - EQ-5D (Utility): 0.37*** / 0.13 / 0.21* / 0.51*** / 0.36*** / 0.40*** / 0.36*** / -0.41*** / -0.44*** / - 0.34*** / -0.42*** / -0.38*** / -0.32*** - Global HRQoL: 0.62*** / 0.28** / 0.35*** / 0.33*** / 0.29*** / 0.53*** / 0.51*** / -0.46*** / -0.43*** / -0.39*** / -0.51*** / -0.31*** / -0.27***   Statistical significante: (*) p < 0.05; (**) p < 0.01; (***) p < 0.001 | Very good | (+) |
| Kuspinar (2019) [77] | – | PGI correlated with EQ-5D-5L, SF-6D, HUI-II and HUI-III. | Very good | (?) |
| Kuspinar (2020) [78] | – | Pearson correlation of PGI with PDQ-8 = -0.35 [p = 0.006] | Very good | (+) |
| Hagell (2011) [79] | – | – | – | – |
| Steffen (2008) [80] | – | – | – | – |
| Hagell (2008) [81] | – | – | – | – |
| Schneider (2010) [82] | 213 | Spearman significative (p < 0.05) correlations of WHO-5 with:   - BDI = -0,646* - UPDRS-Part 3 = -0.539* - UDPRS-Part 4 = -0.425 - UPDRS-Total = -0.402 - H&YS = -0.157   Statistical significance: (*) p < 0.01 | Very good | (+) |
| Hendred (2016) [83] | 96 | Pearson correlations of WHOQOL-BREF (Physical health / Mental health / Social relationships / Environment) with:   - Age = 0.25* / 0.19 / 0.15 / 0.29** - Years of education = 0.19 / 0.16 / 0.22* / 0.38** - Duration of PD (years) = -0.14 / 0.02 / 0.16 / -0.11 - Daily dose of levodopa (mg) = -0.31** / -0.20 / -0.05 / -0.31** - UPDRS-Motor = -0.01 / -0.01 / -0.01 / -0.01 - MMSE = -0.24 / 0.96 / 0.16 / 0.01 - DEX = -0.42** / -0.55** / -0.44** / -0.37** - BDI-II = -0.45** / -0.72** / -0.51** / -0.58** - LSAS = -0.44** / -0.57** / -0.43** / -0.50** - AES = -0.40** / -0.61** / -0.57** / -0.56** - PDFS = -0.75** / -0.56** / -0.40** / -0.47** - Physical daily activities = 0.42** / 0.23 / 0.13 / 0.23* - Cognitive ADL = 0.26* / 0.33** / 0.22* / 0.26*   Statistical significance: (*) p < 0.05; (**) p < 0.001 | Very good | (+) |
