# Supplementary material for: Quality of life measures in Parkinson’s disease: a systematic literature review of patient-reported outcomes measures (PROMs) and their psychometric properties
Source: J Neurol. 2025 Aug 28;272(9):598. doi: 10.1007/s00415-025-13348-x (PMC12394374; doi:10.1007/s00415-025-13348-x)
Supplement: Supplementary file 10 — Supplementary file10 (DOCX 125 KB) [file 415_2025_13348_MOESM10_ESM.docx]

**Quality of Life Measures in Parkinson’s Disease: A Systematic Literature Review of Patient-Reported Outcomes Measures (PROMs) and their Psychometric Properties**

**– ONLINE RESOURCE 8 –**

Table S14. Descriptions of the studies’ findings in relation to the Internal Consistency of the PROMs.

| Eligible study | Sample size | Findings of the study in relation to Internal Consistency | COSMIN assessment | |
| --- | --- | --- | --- | --- |
|  |  |  | **RoB** | **Good property** |
| Spliethoff-Kamminga (2003) [1] | 54 | Cronbach’s alpha (α) for Bela-P-K (Bb): Total = 0.90 / Achievement capability - Physical symptoms = 0.83 / Emotional symptoms = 0.71 / Social functioning = 0.70 / Partner-bonding – Family = 0.61  Cronbach’s alpha (α) for Bela-P-K (Nfh): Total = 0.93 / Achievement capability - Physical symptoms = 0.88 / Emotional symptoms = 0.82 / Social functioning = 0.76 / Partner-bonding – Family = 0.77  Spearman correlation between Bb and Nfh (Rho): Total = 0.74 / Achievement capability - Physical symptoms = 0.67 / Emotional symptoms = 0.78 / Social functioning = 0.69 / Partner-bonding – Family = 0.68 | Very good | (?) |
| Ortelli (2017) [2] | 202 | Cronbach’s alpha (α) for Bela-P-K (Bb): Total = 0.91 / Achievement capability - Physical symptoms = 0.72 / Emotional symptoms = 0.74 / Social functioning = 0.79 / Partner-bonding – Family = 0.71  Cronbach’s alpha (α) for Bela-P-K (Nfh): Total = 0.91 / Achievement capability - Physical symptoms = 0.75 / Emotional symptoms = 0.76 / Social functioning = 0.80 / Partner-bonding – Family = 0.73  Spearman correlation between Bb and Nfh (Rho): Total = 0.84 / Achievement capability - Physical symptoms = 0.78 / Emotional symptoms = 0.80 / Social functioning = 0.88 / Partner-bonding – Family = 0.88 | Very good | (+) |
| Bayen (2021) [3] | 18 | The report lacks measures for internal consistency but refers to good property (“strong internal consistency”) | Doubtful | (?) |
| Aggarwal (2013) [4] | 277 | Cronbach’s alpha (α) for Indo-PDQOL: Total = 0.95 / Dimensions [Range] = 0.61-0.91 | Very good | (+) |
| Kuharic (2022) [5] | – | – | – | – |
| Kuharic (2024) [6] | 569 | Cronbach’s alpha (α) for OFFELIA domains: Functioning = 0.94 / Emotional wellbeing = 0.89  Item-Total Spearman correlations > 0.5 for all items except “Employment” for which the Spearman Rho was 0.47 in all participants and 0.61 for those participants employed | Very good | (+) |
| Peto (1995) [7] | 359 | Cronbach’s alpha (α) for PDQ-39 dimensions: Mobility = 0.94 / ADL = 0.89 / Emotional wellbeing = 0.83 / Stigma = 0.80 / Social support = 0.69 / Cognition = 0.70 / Communication = 0.79 / Bodily discomfort = 0.75 | Very good | (?) |
|  | Test: 227 / Retest: 223 | Cronbach’s alpha (α) for PDQ-39 dimensions [Test; Retest]: Mobility = 0.95; 0.95 / ADL = 0.90; 0.90 /  Emotional wellbeing = 0.88; 0.90 / Stigma = 0.86; 0.87 / Social support = 0.66; 0.73 / Cognition = 0.74; 0.75 / Communication = 0.76; 0.78 / Bodily discomfort = 0.72; 0.76 | Very good | (?) |
| Jenkinson (1997) [8] | 201 | Cronbach’s alpha (α) for PDQ-39 = 0.84 | Very good | (+) |
|  | 136 | Cronbach’s alpha (α) for PDQ-39 = 0.89 | Very good | (+) |
| Jenkinson (1997) [9] | 207 | **PDQ-39:**  Cronbach’s alpha (α) for PDQ-39 = 0.84 | Very good | (+) |
|  | – | – | – | – |
| Martínez-Martín (1998) [10] | 103 | Cronbach’s alpha (α) for PDQ-39 dimensions: Mobility = 0.94 / ADL = 0.89 / Emotional wellbeing = 0.80 / Stigma = 0.80 / Social support = 0.69 / Cognition = 0.70 / Communication = 0.79 / Bodily discomfort = 0.75 | Very good | (?) |
| Bushnell (1999) [11] | 75 | Cronbach’s alpha (α) for PDQ-39 dimensions: Mobility = 0.96 / ADL = 0.89 / Emotional wellbeing = 0.90 / Stigma = 0.86 / Social support = 0.51 / Cognition = 0.80 / Communication = 0.81 / Bodily discomfort = 0.73 | Very good | (?) |
| Andreu (2000) [12] | 126 | Cronbach’s alpha (α) for PDQ-39 [Test; Retest; Proxy] = 0.94; 0.95; 0.95  Cronbach’s alpha (α) for PDQ-39 dimensions [Test; Retest; Proxy]: Mobility = 0.90; 0.92; 0.92 / ADL = 0.87; 0.87; 0.85 / Emotional wellbeing = 0.86; 0.88; 0.86 / Stigma = 0.81; 0.82; 0.82 / Social support = 0.74; 0.81;0.81 / Cognition = 0.56; 0.65; 0.74 / Communication = 0.71; 0.73; 0.71 / Bodily discomfort = 0.61; 0.64; 0.75 | Very good | (?) |
| Schrag (2000) [13] | – | **PDQ-39:**  – | – | – |
|  | – | **EQ-5D-3L:**  – | – | – |
|  | – | **EQ-VAS:**  – | – | – |
|  | – | **SF-36:**  – | – | – |
| Katsarou (2001) [14] | 119 | Cronbach’s alpha (α) for PDQ-39 dimensions: Mobility = 0.94 / ADL = 0.89 / Emotional wellbeing = 0.88 / Stigma = 0.89 / Social support = 0.84 / Cognition = 0.73 / Communication = 0.72 / Bodily discomfort = 0.71 | Very good | (+) |
| Peto (2001) [15] | – | – | – | – |
| Tsang (2002) [16] | 57 | Cronbach’s alpha (α) for PDQ-39 dimensions: Mobility = 0.89 / ADL = 0.90 / Emotional wellbeing = 0.90 / Stigma = 0.54 / Social support = 0.77 / Cognition = 0.75 / Communication = 0.81 / Bodily discomfort = 0.66 | Very good | (?) |
| Hagell (2003) [17] | 71 | Cronbach’s alpha (α) for PDQ-39 dimensions: Mobility = 0.96 / ADL = 0.89 / Emotional wellbeing = 0.90 / Stigma = 0.85 / Social support = 0.75 / Cognition = 0.85 / Communication = 0.82 / Bodily discomfort = 0.73 | Very good | (?) |
| Jenkinson (2003) [18] | 676 | Cronbach’s alpha (α) for PDQ-39 dimensions: Mobility = 0.95 / ADL = 0.91 / Emotional wellbeing = 0.91 / Stigma = 0.86 / Social support = 0.68 / Cognition = 0.79 / Communication = 0.87 / Bodily discomfort = 0.73 | Very good | (?) |
|  | 676 | Cronbach’s alpha (α) for PDQ-39 dimensions: Mobility = 0.94 / ADL = 0.90 / Emotional wellbeing = 0.86 / Stigma = 0.82 / Social support = 0.72 / Cognition = 0.75 / Communication = 0.80 / Bodily discomfort = 0.76 | Very good | (+) |
|  | 676 | Cronbach’s alpha (α) for PDQ-39 dimensions: Mobility = 0.96 / ADL = 0.94 / Emotional wellbeing = 0.90 / Stigma = 0.80 / Social support = 0.13 / Cognition = 0.82 / Communication = 0.76 / Bodily discomfort = 0.77 | Very good | (?) |
|  | 676 | Cronbach’s alpha (α) for PDQ-39 dimensions: Mobility = 0.93 / ADL = 0.91 / Emotional wellbeing = 0.83 / Stigma = 0.72 / Social support = 0.50 / Cognition = 0.70 / Communication = 0.73 / Bodily discomfort = 0.56 | Very good | (?) |
|  | 676 | Cronbach’s alpha (α) for PDQ-39 dimensions: Mobility = 0.93 / ADL = 0.87 / Emotional wellbeing = 0.87 / Stigma = 0.77 / Social support = 0.36 / Cognition = 0.64 / Communication = 0.73 / Bodily discomfort = 0.50 | Very good | (?) |
| Park (2004) [19] | 14 | Cronbach’s alpha (α) > 0.85 for all PDQ-39 dimensions | Very good | (+) |
| Tan (2004) [20] | 88 | **PDQ-39:**  Cronbach’s alpha (α) for PDQ-39 = 0.82  Cronbach’s alpha (α) for PDQ-39 dimensions: Mobility = 0.94 / ADL = 0.92 / Emotional wellbeing = 0.87 / Stigma = 0.85 / Social support = 0.82 / Cognition = 0.74 / Communication = 0.78 / Bodily discomfort = 0.59 | Very good | (?) |
|  | 88 | **PDQ-8:**  Cronbach’s alpha (α) for PDQ-8 = 0.75 | Very good | (+) |
| Fitzpatrick (2004) [21] | 728 | Cronbach’s alpha (α) for PDQ-39 [Test; Retest] = 0.95; 0.96  Cronbach’s alpha (α) for PDQ-39 dimensions [Test; Retest]: Mobility = 0.96; 0.96 / ADL = 0.90; 0.91 / Emotional wellbeing = 0.89; 0.90 / Stigma = 0.83; 0.83 / Social support = 0.50; 0.53 / Cognition = 0.77; 0.77 / Communication = 0.82; 0.82 / Bodily discomfort = 0.73; 0.75 | Very good | (?) |
|  | 132 | Cronbach’s alpha (α) for PDQ-39 [Test; Retest] = 0.96; 0.96  Cronbach’s alpha (α) for PDQ-39 dimensions [Test; Retest]: Mobility = 0.95; 0.96 / ADL = 0.90; 0.91 / Emotional wellbeing = 0.89; 0.90 / Stigma = 0.82; 0.80 / Social support = 0.78; 0.80 / Cognition = 0.0.79; 0.73 / Communication = 0.80; 0.75 / Bodily discomfort = 0.81; 0.80 | Very good | (?) |
| Haapaniemi (2004) [22] | – | – | – | – |
| Martínez-Martín (2004) [23] | 137 | Cronbach’s alpha (α) for PDQ-39 dimensions: Mobility = 0.92 / ADL = 0.93 / Emotional wellbeing = 0.60 / Stigma = 0.78 / Social support = 0.33 / Cognition = 0.64 / Communication = 0.40 / Bodily discomfort = 0.48 | Very good | (?) |
| Ma (2005) [24] | 73 | Cronbach’s alpha (α) for PDQ-39 dimensions: Mobility = 0.96 / ADL = 0.90 / Emotional wellbeing = 0.86 / Stigma = 0.81 / Social support = 0.58 / Cognition = 0.63 / Communication = 0.80 / Bodily discomfort = 0.60 | Very good | (?) |
| Luo (2005) [25] | 63 | **PDQ-39:**  Cronbach’s alpha (α) for PDQ-39 = 0.78  Cronbach’s alpha (α) for PDQ-39 dimensions: Mobility = 0.89 / ADL = 0.88 / Emotional wellbeing = 0.90 / Stigma = 0.78 / Social support = 0.69 / Cognition = 0.64 / Communication = 0.74 / Bodily discomfort = 0.84 | Very good | (?) |
|  | 63 | **PDQ-8:**  Cronbach’s alpha (α) for PDQ-8 = 0.78 | Very good | (+) |
| Martínez-Martín (2007) [26] | 188 | Cronbach’s alpha (α) for PDQ-39 dimensions: Mobility = 0.92 / ADL = 0.93 / Emotional wellbeing = 0.77 / Stigma = 0.81 / Social support = 0.43 / Cognition = 0.66 / Communication = 0.56 / Bodily discomfort = 0.61 | Very good | (?) |
| Hagell (2007) [27] | 202 | Cronbach’s alpha (α) for PDQ-39 dimensions: Mobility = 0.95 (95CI = 0.94-0.96) / ADL = 0.89 (95CI = 0.87-0.91) / Emotional wellbeing = 0.89 (95CI = 0.87-0.91) / Stigma = 0.85 (95CI = 0.81-0.88) / Social support = 0.74 (95CI = 0.66-0.81) / Cognition = 0.74 (95CI = 0.67-0.79) / Communication = 0.87 (95CI = 0.83-0.90) / Bodily discomfort = 0.72 (95CI = 0.65-0.78) | Very good | (+) |
| Krikmann (2008) [28] | 81 | Cronbach’s alpha (α) for PDQ-39 dimensions: Mobility = 0.85 / ADL = 0.85 / Emotional wellbeing = 0.85 / Stigma = 0.86 / Social support = 0.84 / Cognition = 0.82 / Communication = 0.83 / Bodily discomfort = 0.81 | Very good | (+) |
| Marinus (2008) [29] | 177 | Cronbach’s alpha (α) for PDQ-39 dimensions: Mobility = 0.91 / ADL = 0.84 / Emotional wellbeing = 0.86 / Stigma = 0.81 / Social support = 0.76 / Cognition = 0.71 / Communication = 0.73 / Bodily discomfort = 0.59 | Very good | (?) |
| Serrano-Dueñas (2008) [30] | 131 | **PDQ-39:**  Cronbach’s alpha (α) for PDQ-39 = 0.9684 | Very good | (+) |
|  | 131 | **PDQL:**  Cronbach’s alpha (α) for PDQL = 0.9798 | Very good | (+) |
|  | 131 | **PIMS:**  Cronbach’s alpha (α) for PIMS (first assessment / second assessment) = 0.8821 / 0.9015 | Very good | (+) |
| Žiropađa (2009) [31] | 102 | Cronbach’s alpha (α) for PDQ-39 = 0.83  Cronbach’s alpha (α) for PDQ-39 dimensions (range) = 0.59-0.91 | Very good | (?) |
| Nojomi (2010) [32] | 200 | Cronbach’s alpha (α) for PDQ-39 = 0.93  Cronbach’s alpha (α) for PDQ-39 dimensions: Mobility = 0.91 / ADL = 0.88 / Emotional wellbeing = 0.86 / Stigma = 0.87 / Social support = 0.70 / Cognition = 0.60 / Communication = 0.78 / Bodily discomfort = 0.75 | Very good | (?) |
| Luo (2010) [33] | 71 | Cronbach’s alpha (α) for PDQ-39 = 0.84  Cronbach’s alpha (α) for PDQ-39 dimensions: Mobility = 0.86 / ADL = 0.87 / Emotional wellbeing = 0.87 / Stigma = 0.88 / Social support = 0.87 / Cognition = 0.60 / Communication = 0.86 / Bodily discomfort = 0.87 | Very good | (+) |
| Huang (2010) [34] | – | **PDQ-39:**  – | – | – |
|  | 100 | Cronbach’s alpha (α) for PDQ-8 = 0.81 | Very good | (+) |
| Zhang (2011) [35] | 126 | Cronbach’s alpha (α) for PDQ-39 dimensions: Mobility = 0.919 / ADL = 0.897 / Emotional wellbeing = 0.887 / Stigma = 0.835 / Social support = 0.803 / Cognition = 0.486 / Communication = 0.584 / Bodily discomfort = 0.542 | Very good | (?) |
| Kwon (2013) [36] | 101 | Cronbach’s alpha (α) for PDQ-39 dimensions: Mobility = 0.78 / ADL = 0.80 / Emotional wellbeing = 0.73 / Stigma = 0.58 / Social support = 0.64 / Cognition = 0.77 / Communication = 0.75 / Bodily discomfort = 0.76 | Very good | (?) |
| Park (2014) [37] | 93 | Cronbach’s alpha (α) for PDQ-39 dimensions: Mobility = 0.97 / ADL = 0.96 / Emotional wellbeing = 0.93 / Stigma = 0.90 / Social support = 0.70 / Cognition = 0.89 / Communication = 0.75 / Bodily discomfort = 0.78 | Very good | (+) |
| Fereshtehnejad (2014) [38] | 114 | **PDQ-39:**  Cronbach’s alpha (α) for PDQ-39 = 0.939 | Very good | (+) |
|  | 114 | **PDQ-8:**  Cronbach’s alpha (α) for PDQ-8 = 0.74 | Very good | (+) |
| Krygowska-Wajs (2015) [39] | 119 | **PDQ-39:**  Cronbach’s alpha (α) for PDQ-39 dimensions (range) = 0.81-0.94 | Very good | (+) |
|  | 119 | **PDQ-8:**  Cronbach’s alpha (α) for PDQ-8 = 0.79 | Very good | (+) |
| Morley (2015, a) [40] | 118 | Cronbach’s alpha (α) for PDQ-39 dimensions: Mobility = 0.95 / ADL = 0.88 / Emotional wellbeing = 0.88 / Stigma = 0.83 / Social support = 0.76 / Cognition = 0.64 / Communication = 0.87 / Bodily discomfort = 0.71 | Very good | (?) |
| Morley (2015, b) [41] | 118 | Cronbach’s alpha (α) for PDQ-39 dimensions (range) = 0.64-0.95 | Very good | (?) |
| Jesus-Ribeiro (2017) [42] | 100 | **PDQ-39:**  Cronbach’s alpha (α) for PDQ-39 dimensions: Mobility = 0.951 / ADL = 0.976 / Emotional wellbeing = 0.872 / Stigma = 0.669 / Social support = 0.981 / Cognition = 0.943 / Communication = 0.947 / Bodily discomfort = 0.657 | Very good | (?) |
|  | 100 | **PDQL:**  Cronbach’s alpha (α) for PDQL (Total / Parkinsonian symptoms / Systemic symptoms / Emotional functioning / Social functioning) = 0.975 / 0.978 / 0.973 / 0.784 / 0.974 | Very good | (+) |
| Galeoto (2018) [43] | 104 | Cronbach’s alpha (α) for PDQ-39 dimensions: Mobility = 0.92 / ADL = 0.87 / Emotional wellbeing = 0.80 / Stigma = 0.70 / Social support = 0.69 / Cognition = 0.70 / Communication = 0.76 / Bodily discomfort = 0.76 | Very good | (+) |
| Suratos (2018) [44] |  | Cronbach’s alpha (α) for PDQ-39 = 0.8455  Cronbach’s alpha (α) for PDQ-39 dimensions: Mobility = 0.8716 / ADL = 0.8722 / Emotional wellbeing = 0.8563 / Stigma = 0.8821 / Social support = 0.8670 / Cognition = 0.8628 / Communication = 0.8705 / Bodily discomfort = 0.8721 | Very good | (+) |
| Holden (2019) [45] | 201 | **PDQ-39:**  Cronbach’s alpha (α) for PDQ-39 = 0.95 | Very good | (+) |
|  | 201 | **McGill QOL:**  Cronbach’s alpha (α) for McGill QOL = 0.88 | Very good | (+) |
|  | 201 | **PROMIS-29:**  Cronbach’s alpha (α) for PROMIS-29 = 0.93 | Very good | (+) |
|  |  | **QOL-AD:**  Cronbach’s alpha (α) for QOL-AD = 0.83 | Very good | (+) |
| Nelson (2020) [46] | 160 | Cronbach’s alpha (α) for PDQ-39 = 0.95 | Very good | (+) |
| Kim (2020) [47] | 80 | **PDQ-39:**  Cronbach’s alpha (α) for PDQ-39 = 0.97  Cronbach’s alpha (α) for PDQ-39 dimensions: Mobility = 0.94 / ADL = 0.92 / Emotional wellbeing = 0.93 / Stigma = 0.81 / Social support = 0.72 / Cognition = 0.82 / Communication = 0.77 / Bodily discomfort = 0.84 | Very good | (+) |
|  | 80 | **PDQ-8:**  Cronbach’s alpha (α) for PDQ-8 (Nested with PDQ-39 / Independent to PDQ-39) = 0.86 / 0.88 | Very good | (+) |
| Hanff (2023) [48] | – | – | – | – |
| Katsarou (2004) [49] | 228 | Cronbach’s alpha (α) for PDQ-8 = 0.72 | Very good | (+) |
| Tan (2007) [50] | – | – | – | – |
|  | – | – | – | – |
|  | – | – | – | – |
| Jenkinson (2007) [51] | 183 | Cronbach’s alpha (α) for PDQ-8 = 0.88 | Very good | (+) |
|  | 125 | Cronbach’s alpha (α) for PDQ-8 = 0.83 | Very good | (+) |
|  | 200 | Cronbach’s alpha (α) for PDQ-8 = 0.87 | Very good | (+) |
|  | 198 | Cronbach’s alpha (α) for PDQ-8 = 0.79 | Very good | (+) |
|  | 99 | Cronbach’s alpha (α) for PDQ-8 = 0.73 | Very good | (+) |
| Franchignoni (2008) [52] | – | – | – | – |
|  | – | – | – | – |
|  | 200 | Cronbach’s alpha (α) for PDQ-8 = 0.72 | Very good | (+) |
| Dal Bello-Haas (2009) [53] | – | – | – | – |
| Alvarado-Bolaños (2015) [54] | – | **PDQ-8:**  – | – | – |
|  | – | **EQ-5D-5L:**  – | – | – |
|  | – | **EQ-VAS:**  – | – | – |
| Kahraman (2018) [55] | 83 | Cronbach’s alpha (α) for PDQ-8 = 0.78 | Very good | (+) |
| Ramadhan (2022) [56] | – | **PDQ-8:**  – | – | – |
|  | – | **EQ-5D-3L:**  – | – | – |
| Stathis (2022) [57] | 60 | **PDQ-8:**  Cronbach’s alpha (α) for PDQ-8 = 0.779 | Very good | (+) |
|  | 60 | **PDQoL-7:**  Cronbach’s alpha (α) for PDQoL-7 was 0.804. Alpha when excluding each item: Item 1 = 0.767 / Item 2 = 0.760 / Item 3 = 0.784 / Item 4 = 0.767 / Item 5 = 0.776 / Item 6 = 0.774 / Item 7 = 0.815 | Very good | (+) |
| Kawaguchi (2021) [58] | 54 | Cronbach’s alpha (α) for PDQ-DAT domains: ADL = 0.861 / Problems related with the therapy device = 0.836 / Psychological problems = 0.836 | Very good | (+) |
| De Boer (1996) [59] | 384 | Cronbach’s alpha (α) for PDQL (Total / Parkinsonian symptoms / Systemic symptoms / Emotional functioning / Social functioning) = 0.94 / 0.86 / 0.80 / 0.87 / 0.82 | Very good | (+) |
| Serrano-Dueñas (2004) [60] | 137 | Cronbach’s alpha (α) for PDQL (Total / Parkinsonian symptoms / Systemic symptoms / Emotional functioning / Social functioning) = 0.92 / 0.85 / 0.69 / 0.81 / 0.78 | Very good | (+) |
| Campos (2011) [61] | 58 | Cronbach’s alpha (α) for PDQL (Total / Parkinsonian symptoms / Systemic symptoms / Emotional functioning / Social functioning) = 0.93 / 0.83 / 0.65 / 0.79 / 0.80 | Very good | (+) |
| Dereli (2015) [62] | 89 | Cronbach’s alpha (α) for PDQL (Total / Parkinsonian symptoms / Systemic symptoms / Emotional functioning / Social functioning) = 0.97 / 0.90 / 0.88 / 0.89 / 0.89 | Very good | (+) |
| Welsh (2003) [63] | 222 | Cronbach’s alpha (α) for PDQUALIF (Social -Role life / Self-image-Sexuality / Sleep / Outlook / Physical function / Independence / Urinary function / Total) = 0.85 / 0.79 / 0.59 / 0.58 / 0.55 / 0.72 / 0.62 / 0.89 | Very good | (+) |
| Calne (1996) [64] | 147 | Cronbach’s alpha (α) for PIMS = 0.898 | Very good | (+) |
| Schulzer (2003) [65] | 116 | Cronbach’s alpha (α) for PIMS = 0.872 | Very good | (+) |
| Aggarwal (2020) [66] | 295 | Cronbach’s alpha (α) for QLPD domains (ADL / Mobility / Psychological / Fear / Social / Family / Treatment / Finances / Nonmotor sympotms) = 0.82 / 0.83 / 0.78 / 0.80 / 0.78 / 0.74 / 0.42 / 0.91 / 0.83 | Very good | (+) |
| Kuehler (2003) [67] | – | **QLSM-DBS:**  Cronbach’s alpha (α) for QLSM-DBS (Total / Reliability of the neurostimulator / Inconspicuousness of the neurostimulator / Independent handling – manipulation of the neurostimulator / Medical care / Absence of bodily symptoms – side effects of the neurostimulation) = 0.73 / 0.61 / 0.34 / 0.57 / 0.54 / 0.46 | Very good | (+) |
|  | – | **QLSM-MD:**  Cronbach’s alpha (α) for QLSM-MD (Total / Controllability – fluidity of movement / Absence of dizziness – steadiness when standing and walking / Hand dexterity throughout the day / Articulation – fluency of speech / Ability to swallow / Absence of false bodily sensations / Bladder – intestinal function / Sexual excitability / Undisturbed sleep / Memory – clear thinking / Independence from help / Inconspicuousness of illness) = 0.83 / 0.74 / 0.60 / 0.51 / 0.53 / 0.62 / 0.59 / 0.59 / 0.38 / 0.36 / 0.61 / 0.70 / 0.66 | Very good | (+) |
| Krygowska-Wajs (2015) [68] | 30 | **QLSM-DBS:**  Cronbach’s alpha (α) for QLSM-DBS = 0.69 | Very good | (–) |
|  | 119 | **QLSM-MD:**  Cronbach’s alpha (α) for QLSM-DBS = 0.92 | Very good | (+) |
| Bose (2018) [69] | 120 | Cronbach’s alpha (α) for QoLQ-PwP = 0.954 | Very good | (+) |
| Diniz (2018) [70] | 140 | Cronbach’s alpha (α) for QOLSQ (Total / Domain 1 / Domain 2 / Domain 3 / Domain 4) [Test; Retest] = 0.90; 0.90 / 0.94; 0.95 / 0.81; 0.91 / 0.71; 0.69 / 0.75; 0.79 | Very good | (+) |
| García-Gordillo (2013) [71] | – | **15D:**  – | – | – |
|  | – | **EQ-5D-5L:**  Cronbach’s alpha (α) for EQ-5D-5L = 0.828 | Very good | (+) |
| Del Pozo-Cruz (2018) [72] | – | **15D:**  – | – | – |
|  | – | **SF-6D:**  – | – | – |
| Luo (2009) [73] | – | **EQ-5D-3L**  – | – | – |
|  |  | **EQ-VAS**  – | – | – |
|  | – | **EQ-5D-3L**  – | – | – |
|  |  | **EQ-VAS**  – | – | – |
|  | – | **EQ-5D-3L**  – | – | – |
|  |  | **EQ-VAS**  – | – | – |
| Garcia-Gordillo (2015) [74] | – | **EQ-5D-3L:**  – | – | – |
|  | – | **SF-6D:**  – | – | – |
| Nowinski (2010) [75] | – | – | – | – |
| Nowinski (2016) [76] | 120 | Cronbach’s alpha (α) for Neuro-QOL domains:   - Positive Affect and Well-Being = 0.94 - Applied Cognition–General Concerns = 0.90 - Applied Cognition–Executive Function = 0.90 - Lower Extremity Function–Mobility = 0.84 - Upper Extremity Function–Fine Motor, ADL = 0.82 - Ability to Participate in Social Roles and Activities = 0.94 - Satisfaction with Social Roles and Activities = 0.89 - Depression = 0.93 - Anxiety = 0.91 - Stigma = 0.85 - Fatigue = 0.93 - Sleep Disturbance = 0.81 - Emotional and Behavioral Dyscontrol = 0.91 | Very good | (+) |
| Kuspinar (2019) [77] | – | – | – | – |
| Kuspinar (2020) [78] | – | – | – | – |
| Hagell (2011) [79] | – | – | – | – |
| Steffen (2008) [80] | 37 | Cronbach’s alpha (α) for SF-36 (Physical functioning / Role physical / Pain / General health / Energy / Social functioning / Role emotional / Mental health) [Test; Retest] = 0.85; 0.87 / 0.85; 0.74 / 0.95; 0.91 / 0.85; 0.80 / 0.85; 0.91 / 0.67; 0.84 / 0.91; 0.89 / 0.84; 0.93 | Very good | (+) |
| Hagell (2008) [81] | 202 | Cronbach’s alpha (α) for SF-36 (Physical functioning / Role physical / Pain / General health / Energy / Social functioning / Role emotional / Mental health) = 0.94 / 0.87 / 0.92 / 0.79 / 0.84 / 0.78 / 0.88 / 0.82 | Very good | (+) |
| Schneider (2010) [82] | 213 | Cronbach’s alpha (α) for WHO-5 = 0.83 | Very good | (+) |
| Hendred (2016) [83] | 96 | Cronbach’s alpha (α) for WHOQOL-BREF (Physical health / Psychological health / Social relationships / Environment) = 0.85 / 0.85 / 0.64 / 0.84 | Very good | (+) |
