# Supplementary material for: Quality of life measures in Parkinson’s disease: a systematic literature review of patient-reported outcomes measures (PROMs) and their psychometric properties
Source: J Neurol. 2025 Aug 28;272(9):598. doi: 10.1007/s00415-025-13348-x (PMC12394374; doi:10.1007/s00415-025-13348-x)
Supplement: Supplementary file 11 — Supplementary file11 (DOCX 122 KB) [file 415_2025_13348_MOESM11_ESM.docx]

**Quality of Life Measures in Parkinson’s Disease: A Systematic Literature Review of Patient-Reported Outcomes Measures (PROMs) and their Psychometric Properties**

**– ONLINE RESOURCE 9 –**

Table S15. Descriptions of the studies’ findings in relation to the ICC (Test-Retest) of the PROMs.

| Eligible study | Sample size | Findings of the study in relation to CCI (Test-Retest) | COSMIN assessment | |
| --- | --- | --- | --- | --- |
|  |  |  | **RoB** | **Good property** |
| Spliethoff-Kamminga (2003) [1] | – | – | – | – |
| Ortelli (2017) [2] | – | – | – | – |
| Bayen (2021) [3] | 18 | Differences in test-retest scores were found for 10 items | Doubtful | (?) |
| Aggarwal (2013) [4] | – | – | – | – |
| Kuharic (2022) [5] | – | – | – | – |
| Kuharic (2024) [6] | – | – | – | – |
| Peto (1995) [7] | – | – | – | – |
|  | 223 | ICC for Test-Retest (days between administrations = 3-6) for PDQ-39 dimensions [p < 0.001]: Mobility = 0.94 / ADL = 0.93 / Emotional wellbeing = 0.90 / Stigma = 0.90 / Social support = 0.68 / Cognition = 0.86 / Communication = 0.86 / Bodily discomfort = 0.80 | Very good | (+) |
| Jenkinson (1997) [8] | – | – | – | – |
|  | – | – | – | – |
| Jenkinson (1997) [9] | – | **PDQ-39:**  – | – | – |
|  | – | **PDQ-8:**  – | – | – |
| Martínez-Martín (1998) [10] | – | – | – | – |
| Bushnell (1999) [11] | 64 | ICC for Test-Retest (days between administrations = 3) for PDQ-39 dimensions [p < 0.001]: Mobility = 0.95 / ADL = 0.96 / Emotional wellbeing = 0.95 / Stigma = 0.88 / Social support = 0.86 / Cognition = 0.93 / Communication = 0.86 / Bodily discomfort = 0.88 | Very good | (+) |
| Andreu (2000) [12] | 126 | ICC for Test-Retest (days between administrations not specified) for PDQ-39 dimensions: Mobility = 0.90 / ADL = 0.90 / Emotional wellbeing = 0.90 / Stigma = 0.86 / Social support = 0.77 / Cognition = 0.86 / Communication = 0.81 / Bodily discomfort = 0.84 / SI = 0.92 | Very good | (+) |
| Schrag (2000) [13] | – | **PDQ-39:**  – | – | – |
|  | – | **EQ-5D-3L:**  – | – | – |
|  | – | **EQ-VAS:**  – | – | – |
|  | – | **SF-36:**  – | – | – |
| Katsarou (2001) [14] | 119 | ICC (Pearson correlation coefficient) for Test-Retest (days between administrations = 5-7) for PDQ-39 dimensions [p < 0.001]: Mobility = 0.89 / ADL = 0.95 / Emotional wellbeing = 0.95 / Stigma = 0.93 / Social support = 0.95 / Cognition = 0.84 / Communication = 0.90 / Bodily discomfort = 0.91 | Very good | (+) |
| Peto (2001) [15] | – | – | – | – |
| Tsang (2002) [16] | – | – | – | – |
| Hagell (2003) [17] | – | – | – | – |
| Jenkinson (2003) [18] | – | – | – | – |
|  | – | – | – | – |
|  | – | – | – | – |
|  | – | – | – | – |
|  | – | – | – | – |
| Park (2004) [19] | – | – | – | – |
| Tan (2004) [20] | 68 | **PDQ-39:**  ICC for Test-Retest (mean days between administrations = 12 [range = 4-19]) for PDQ-39 dimensions: Mobility = 0.87 / ADL = 0.87 / Emotional wellbeing = 0.76 / Stigma = 0.77 / Social support = 0.73 / Cognition = 0.80 / Communication = 0.77 / Bodily discomfort = 0.67 / SI = 0.85 | Very good | (+) |
|  | 68 | **PDQ-8:**  ICC for Test-Retest (mean days between administrations = 12 [range = 4-19]) for PDQ-8 = 0.80 | Very good | (+) |
| Fitzpatrick (2004) [21] | – | – | – | – |
|  | – | – | – | – |
| Haapaniemi (2004) [22] | – | – | – | – |
| Martínez-Martín (2004) [23] | – | – | – | – |
| Ma (2005) [24] | 22 | ICC for Test-Retest (days between administrations = 5-7) for PDQ-39 dimensions: Mobility = 0.74 / ADL = 0.71 / Emotional wellbeing = 0.88 / Stigma = 0.89 / Social support = 0.95 / Cognition = 0.81 / Communication = 0.76 / Bodily discomfort = 0.84 | Very good | (+) |
| Luo (2005) [25] | 36 | **PDQ-39:**  ICC for Test-Retest (mean days between administrations = 12 [7-4]) for PDQ-39 dimensions: Mobility = 0.76 / ADL = 0.82 / Emotional wellbeing = 0.66 / Stigma = 0.78 / Social support = 0.75 / Cognition = 0.80 / Communication = 0.70 / Bodily discomfort = 0.68 / SI = 0.74 | Very good | (+) |
|  | 36 | **PDQ-8:**  ICC for Test-Retest (mean days between administrations = 12 [7-4]) for PDQ-8 = 0.74 | Very good | (+) |
| Martínez-Martín (2007) [26] | 188 | **PDQ-39:**  ICC for Test-Retest (days between administrations not specified) for PDQ-39 dimensions: Mobility = 0.88 / ADL = 0.81 / Emotional wellbeing = 0.83 / Stigma = 0.90 / Social support = 0.86 / Cognition = 0.76 / Communication = 0.90 / Bodily discomfort = 0.83 | Very good | (+) |
|  | 188 | **PDQL:**  ICC for Test-Retest (days between administrations not specified) for PDQL dimensions (Parkinsonian symptoms / Systemic symptoms / Emotional functioning / Social functioning) = 0.91 / 0.85 / 0.81 / 0.88 |  |  |
| Hagell (2007) [27] | 14 | ICC for Test-Retest (days between administrations = 14) for PDQ-39 dimensions: Mobility = 0.93 [95CI = 0.91-0.95] / ADL = 0.93 [95CI = 0.90-0.95] / Emotional wellbeing = 0.87 [95CI = 0.82-0.91] / Stigma = 0.85 [95CI = 0.79-0.89] / Social support = 0.76 [95CI = 0.66-0.83] / Cognition = 0.86 [95CI = 0.81-0.90] / Communication = 0.86 [95CI = 0.81-0.90] / Bodily discomfort = 0.79 [95CI = 0.72-0.85] | Very good | (+) |
| Krikmann (2008) [28] | 78 | ICC for Test-Retest (days between administrations not specified) for PDQ-39 dimensions [range] = 0.72-0.92 | Very good | (+) |
| Marinus (2008) [29] | – | – | – | – |
| Serrano-Dueñas (2008) [30] | – | **PDQ-39:**  – | – | – |
|  | – | **PDQL:**  – | – | – |
|  | 131 | **PIMS:**  ICC for Test-Retest (days between administrations = 7) for PIMS = 0.9837 (95CI = 0.9770-0.9885). ICC items: Item 1 = 0.9746 (95CI = 0.9642-0.9821) / Item 2 = 0.9514 (IC95 = 0.9313-0.9656) / Item 3 = 0.9368 (IC95 = 0.9107-0.9552) / Item 4 = 0.9196 (IC95 = 0.8865-0.9431) / Item 5 = 0.9286 (IC95 = 0.8990-0.9494) / Item 6 = 0.9584 (IC95 = 0.9412-0.9705) / Item 7 = 0.9569 (IC95 = 0.9362-0.9695) / Item 8 = 0.9515 (IC95 = 0.9315-0.9657) / Item 9 = 0.9131 (IC95 = 0.8773-0.9385) / Item 10 = 0.9764 (IC95 = 0.9666 - 0.9833) | Very good | (+) |
| Žiropađa (2009) [31] | – | – | – | – |
| Nojomi (2010) [32] | 200 | ICC for Test-Retest (days between administrations = 21) for PDQ-39 dimensions: Mobility = 0.90 / ADL = 0.47 / Emotional wellbeing = 0.78 / Stigma = 0.82 / Social support = 0.77 / Cognition = 0.84 / Communication = 0.74 / Bodily discomfort = 0.75 / SI = 0.80 | Very good | (+) |
| Luo (2010) [33] | 68 | ICC for Test-Retest (days between administrations = 7) for PDQ-39 dimensions: Mobility = 0.77 / ADL = 0.82 / Emotional wellbeing = 0.62 / Stigma = 0.67 / Social support = 0.56 / Cognition = 0.71 / Communication = 0.77 / Bodily discomfort = 0.82 / SI = 0.82 | Very good | (+) |
| Huang (2010) [34] | – | **PDQ-39:**  – | – | – |
|  | – | **PDQ-8:**  – | – |  |
| Zhang (2011) [35] | – | – | – | – |
| Kwon (2013) [36] | 102 | ICC for Test-Retest (days between administrations = 10-14) for PDQ-39 dimensions [p < 0.001]: Mobility = 0.943 / ADL = 0.932 / Emotional wellbeing = 0.870 / Stigma = 0.834 / Social support = 0.766 / Cognition = 0.835 / Communication = 0.874 / Bodily discomfort = 0.715 / SI = 0.919 | Very good | (+) |
| Park (2014) [37] | – | – | – | – |
| Fereshtehnejad (2014) [38] | – | **PDQ-39:**  – | – | – |
|  | – | **PDQ-8:**  – |  |  |
| Krygowska-Wajs (2015) [39] | – | **PDQ-39:**  – | – | – |
|  | 78 | **PDQ-8:**  ICC for Test-Retest (days between administrations = 14) for PDQ-8 = 0.84 (95CI = 0.75-0.90) | Adequate | (+) |
| Morley (2015, a) [40] | – | – | – | – |
| Morley (2015, b) [41] | – | – | – | – |
| Jesus-Ribeiro (2017) [42] | 100 | **PDQ-39:**  ICC for Test-Retest (days between administrations = 10-12) for PDQ-39 dimensions: Mobility = 0.907 / ADL = 0.953 / Emotional wellbeing = 0.774 / Stigma = 0.502 / Social support = 0.964 / Cognition = 0.892 / Communication = 0.899 / Bodily discomfort = 0.489 | Very good | (+) |
|  | 100 | **PDQL:**  ICC for Test-Retest (days between administrations = 10-12) for PDQL dimensions (Total / Parkinsonian symptoms / Systemic symptoms / Emotional functioning / Social functioning) = 0.952 / 0.957 / 0.948 / 0.645 / 0.950 |  |  |
| Galeoto (2018) [43] | 35 | ICC for Test-Retest (days between administrations = 3) for PDQ-39 dimensions [p < 0.001]: Mobility = 0.94 [95CI = 0.88-0.97] / ADL = 0.94 [95CI = 0.88-0.97] / Emotional wellbeing = 0.91 [95CI = 0.82-0.86] / Stigma = 0.91 [95CI = 0.82-0.96] / Social support = 0.85 [95CI = 0.71-0.93] / Cognition = 0.0.93 [95CI = 0.82-0.96] / Communication = 0.91 [95CI = 0.82-0.96] / Bodily discomfort = 0.96 [95CI = 0.93-0.98] | Adequate | (+) |
| Suratos (2018) [44] | – | – | – | – |
| Holden (2019) [45] | – | **PDQ-39:**  – | – | – |
|  | – | **McGill QOL:**  – | – | – |
|  | – | **PROMIS-29:**  – | – | – |
|  | – | **QOL-AD:**  – | – | – |
| Nelson (2020) [46] | – | – | – | – |
| Kim (2020) [47] | – | **PDQ-39:**  – | – | – |
|  | – | **PDQ-8:**  – | – | – |
| Hanff (2023) [48] | – | – | – | – |
| Katsarou (2004) [49] | 228 | ICC for Test-Retest (days between administrations not specified) for PDQ-8 = 0.72 (95CI = 0.87-0.92) [p < 0.001] | Very good | (+) |
| Tan (2007) [50] | – | – | – | – |
|  | – | – | – | – |
|  | – | – | – | – |
| Jenkinson (2007) [51] | – | – | – | – |
|  | – | – | – | – |
|  | – | – | – | – |
|  | – | – | – | – |
|  | – | – | – | – |
| Franchignoni (2008) [52] | – | – | – | – |
|  | – | – | – | – |
|  | – | – | – | – |
| Dal Bello-Haas (2009) [53] | 24 | ICC for Test-Retest (mean days between administrations = 12.9; SD = 5.1) for PDQ-8 = 0.82 (95CI = 0.63-0.91) | Very good | (+) |
| Alvarado-Bolaños (2015) [54] | – | **PDQ-8:**  – | – | – |
|  | – | **EQ-5D-5L**  – | – | – |
|  | – | **EQ-VAS**  – | – | – |
| Kahraman (2018) [55] | 83 | ICC for Test-Retest (days between administrations not specified) for PDQ-8 = 0.97 (95CI = 0.93-0.99) [p < 0.001] | Very good | (+) |
| Ramadhan (2022) [56] | – | **PDQ-8:**  – | – | – |
|  | – | **EQ-5D-3L:**  – | – | – |
| Stathis (2022) [57] | – | **PDQ-8:**  – | – | – |
|  | – | **PDQoL-7:**  – | – | – |
| Kawaguchi (2021) [58] | – | – | – | – |
| De Boer (1996) [59] | – | – | – | – |
| Serrano-Dueñas (2004) [60] | – | – | – | – |
| Campos (2011) [61] | 21 | No differences observed in Test-Retest scores (days between administrations not specified) | Very good | (?) |
| Dereli (2015) [62] | 46 | ICC for Test-Retest (days between administrations = 7) for PDQL dimensions (Parkinsonian symptoms / Systemic symptoms / Emotional functioning / Social functioning) = 0.82 / 0.88 / 0.85 / 0.85 |  |  |
| Welsh (2003) [63] | 222 | ICC for Test-Retest (days between administrations = 14) for PDQUALIF (Social -Role life / Self-image-Sexuality / Sleep / Outlook / Physical function / Independence / Urinary function / Total) = 0.86 / 0.82 / 0.70 / 0.68 / 0.86 / 0.69 / 0.85 / 0.88 | Very good | (+) |
| Calne (1996) [64] | 147 | ICC for Test-Retest (days between administrations = 28) for PIMS (Factors 1-3 / Factor 4) = 0.69 / 0.91 | Very good | (+) |
| Schulzer (2003) [65] | 116 | ICC for Test-Retest (days between administrations = 28) for PIMS = 0.825 (95CI = 0.755-0.877) | Very good | (+) |
| Aggarwal (2020) [66] | – | – | – | – |
| Kuehler (2003) [67] | – | **QLSM-DBS:**  – | – | – |
|  | – | **QLSM-MD:**  – | – | – |
| Krygowska-Wajs (2015) [68] | 30 | **QLSM-DBS:**  ICC for Test-Retest (days between administrations = 14) for QLSM-DBS = 0.97 (95CI = 0.95-0.98) | Very good | (+) |
|  | 78 | **QLSM-MD:**  ICC for Test-Retest (days between administrations = 14) for QLSM-MD = 0.87 (95CI = 0.77-0.89) | Very good | (+) |
| Bose (2018) [69] | – | – | – | – |
| Diniz (2018) [70] | 44 | ICC for Test-Retest (days between administrations not specified) for QOLSQ (Total / Domain 1 / Domain 2 / Domain 3 / Domain 4) = 0.99 [95CI = 0.98-0.99] / 0.99 [95CI = 0.98-0.99] / 0.99 [95CI = 0.98-0.99] / 0.99 [95CI = 0.98-0.99] / 0.98 [95CI = 0.97-0.99] / | Very good | (+) |
| García-Gordillo (2013) [71] | – | **15D:**  – | – | – |
|  | – | **EQ-5D-5L:**  – | – | – |
| Del Pozo-Cruz (2018) [72] | – | **15D:**  – | – | – |
|  | – | **SF-36:**  – | – | – |
| Luo (2009) [73] | – | **EQ-5D-3L:**  – | – | – |
|  |  | **EQ-VAS:**  – | – | – |
|  | – | **EQ-5D-3L:**  – | – | – |
|  |  | **EQ-VAS:**  – | – | – |
|  | – | **EQ-5D-3L:**  – | – | – |
|  |  | **EQ-VAS:**  – | – | – |
| Garcia-Gordillo (2015) [74] | – | **EQ-5D-3L:**  – | – | – |
|  | – | **SF-36:**  – | – | – |
| Nowinski (2010) [75] | – | – | – | – |
| Nowinski (2016) [76] | 120 | ICC for Test-Retest (days between administrations = 7) for Neuro-QOL domains:   - Positive Affect and Well-Being = 0.76 - Applied Cognition–General Concerns = 0.72 - Applied Cognition–Executive Function = 0.78 - Lower Extremity Function–Mobility = 0.78 - Upper Extremity Function–Fine Motor, ADL = 0.72 - Ability to Participate in Social Roles and Activities = 0.71 - Satisfaction with Social Roles and Activities = 0.66 - Depression = 0.68 - Anxiety = 0.76 - Stigma = 0.80 - Fatigue = 0.78 - Sleep Disturbance = 0.79   Emotional and Behavioral Dyscontrol = 0.73 | Very good | (+) |
| Kuspinar (2019) [77] | – | – | – | – |
| Kuspinar (2020) [78] | – | – | – | – |
| Hagell (2011) [79] | – | – | – | – |
| Steffen (2008) [80] | 37 | ICC for Test-Retest (days between administrations = 7) for SF-36 (Physical functioning / Role physical / Pain / General health / Energy / Social functioning / Role emotional / Mental health) = 0.80 / 0.85 / 0.89 / 0.85 / 0.88 / 0.71 / 0.84 / 0.83 | Very good | (+) |
| Hagell (2008) [81] | – | – | – | – |
| Schneider (2010) [82] | – | – | – | – |
| Hendred (2016) [83] | – | – | – | – |
